# Exploring the Scope of Functionalized *N*-Acylneuraminic Acid β-Methyl Glycosides as Inhibitors of *Neisseria meningitidis* CMP-Sialic Acid Synthetase

**DOI:** 10.3390/molecules30224329

**Published:** 2025-11-07

**Authors:** Pradeep Chopra, Jana Führing, Preston Ng, Thomas Haselhorst, Jeffrey C. Dyason, Faith J. Rose, Robin J. Thomson, Rita Gerardy-Schahn, I. Darren Grice, Michael P. Jennings, Anja K. Münster-Kühnel, Mark von Itzstein

**Affiliations:** 1 Institute for Biomedicine and Glycomics, Griffith University, Gold Coast, QLD 4222, Australia; pchopra@uga.edu (P.C.); t.haselhorst@griffith.edu.au (T.H.); r.thomson@griffith.edu.au (R.J.T.); d.grice@griffith.edu.au (I.D.G.); 2 Institut für Klinische Biochemie, Medizinische Hochschule, Carl-Neuberg-Straße 1, 30625 Hannover, Germany; fuehring.jana@mh-hannover.de (J.F.); gerardy-schahn.rita@mh-hannover.de (R.G.-S.)

**Keywords:** sialic acids, CMP-sialic acid, CMP-sialic acid synthase, inhibitors, *Neisseria meningitidis*

## Abstract

Cell surface sialylation is utilized by a number of pathogenic bacteria to evade the host immune system through molecular mimicry of host sialoglycoconjugates. Human pathogen *Neisseria meningitidis* serotype B (*Nm*B) expresses both sialylated capsule and surface lipooligosaccharides as pivotal virulence factors. An essential enzyme in the sialylation pathway of *Nm*B is CMP-sialic acid synthetase (CSS), which produces the activated nucleotide sugar necessary for sialic acid transfer. In this work, novel C-4, -5, -7, and -9 functionalized derivatives of neuraminic acid β-methyl glycoside (Neuβ2Me) were synthesized as candidate CSS inhibitors. A number of these were found to reduce the activity of *Nm*B CSS in vitro. The highest inhibition of *Nm*B CSS, in a mixed mode manner, was observed with a Neu5Acβ2Me C-9 serine carboxamide. Direct interaction with the enzyme was confirmed by Saturation Transfer Difference (STD) NMR. Supplementation of growth media with this compound reduced lipooligosaccharide (LOS) sialylation of living *N. meningitidis*, thus providing an interesting starting point for the development of specific *Nm*B CSS inhibitors as an alternative treatment strategy to fight bacterial infections.

## 1. Introduction

*Neisseria meningitidis* serogroup B (*Nm*B) is an obligate human pathogen and a leading cause of meningitis and septicemia in developed countries [1]. Vaccines are currently available for protection against *N. meningitidis* serogroups A, B, C, W, and Y [2,3]. The Men A, C, W, and Y vaccines are based on bacterial capsular polysaccharide antigens [2,3,4]. In contrast, the MenB vaccine uses subcapsular membrane proteins and outer membrane vesicle proteins [2,5] due to the similarity of its capsular polysaccharides to human neuronal glycans [6,7,8]. *Nm*B expresses sialic acid (Sia) on its surface, both in its capsule, which is composed of an α2,8-linked homopolymer of Sia (polySia), and as the terminal sugar on lipooligosaccharides (LOS) [9,10]. Sialic acids are 9-carbon acidic sugars [11], incorporated into glycoconjugates as α-linked sialosides, which are negatively charged under physiological conditions. The highly charged polySia capsule of serogroup B meningococci, as well as LOS terminated by Sia, are pivotal virulence factors [12]. Sia not only mediates interactions with the host [13] but also promotes survival and dissemination of this pathogen. Sialylated LOS has been shown to be involved in immune evasion of *Nm*B, resistance against phagocytosis by human dendritic cells [14,15], and hindrance of complement attack by interfering with the alternative pathway of complement activation [16,17,18]. Genetic interruption of the *Nm*B sialylation pathway abolishes capsule formation as well as LOS sialylation, and results in rapid bacterial killing by human serum [19]. Interference with *Nm*B cell surface sialylation may therefore provide an attractive antimicrobial strategy.

**Figure 1:**
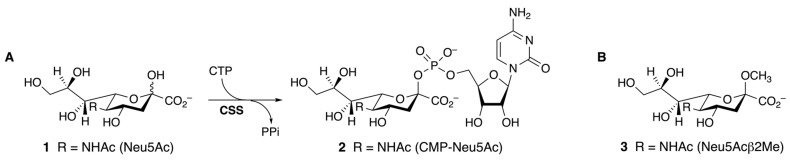
(**A**). Activation of the Sia *N*-acetylneuraminic acid (Neu5Ac) **1** to the nucleotide sugar form CMP-Neu5Ac **2**, catalyzed by CMP-sialic acid synthetase (CSS). (**B**). Neu5Acβ2Me (**3**), an inhibitor of mammalian CSS [27,28].

A prerequisite for incorporation of Sia into sialylated glycans in both pro- and eukaryotes is the enzymatic conversion of free Sia to the activated nucleotide sugar CMP-Sia. Exclusively CMP-Sia is used by sialyltransferases for the transfer of Sia onto nascent sialoglycoconjugates. The sialic acid present in *Nm*B polysaccharides is *N*-acetylneuraminic acid, Neu5Ac **1**. Activation of Neu5Ac to CMP-Neu5Ac **2** through reaction with cytidine 5′-triphosphate (CTP) [20,21] is catalyzed by CMP-sialic acid synthetase (*N*-acylneuraminate cytidylyltransferase, CSS, EC 2.7.7.43) (Figure 1A) [20,21,22,23,24,25]. Interference with CSS activity in mammalian cells abolishes cell surface sialylation [26], suggesting the enzyme as a potential target for manipulation of cell surface sialylation in *Nm*B.

Tolerance of *Nm*B CSS to structural variation on the substrate Neu5Ac at carbon atoms C-5 [29,30,31,32], C-7 [33], C-8 [34], and C-9 [30,31,35], and indeed to replacement of the glycerol side chain [36], has been reported. This substrate tolerance has been exploited for the generation of modified sialoglycans [29,37], including in multi-enzyme and chemoenzymatic syntheses [30,31,32,33,36,38,39]. In contrast, comparatively little research has to date been reported on the inhibition of CSS. Only a relatively small number of studies have examined the inhibitory effect of modified sialic acids [27,28,40,41] or nucleotide isosteres [42] on CSS activity. Removal of the C-2 hydroxyl group of Neu5Ac (the proposed nucleophile in coupling Neu5Ac **1** to CTP [43,44]) was found to abolish recognition by mammalian CSS [27]. Retaining the C-2 oxygen but blocking it with a methyl group—giving the methyl glycoside in the β-configuration to mimic the β-configuration of CMP-Neu5Ac **2**—however, led to inhibition of mammalian CSS, albeit at millimolar concentration [Neu5Acβ2Me (**3**): *K*_i_ = 2.5 mM [27]; 15 mM [28]. As part of our continuing work in sialic acid and CSS biology [13,18,24,45,46,47,48], we sought to explore the potential for inhibition of CSS from pathogenic *Nm*B using novel Neu5Ac derivatives. As the inhibitor template, we chose the above-mentioned β-methyl glycoside of Neu5Ac (Neu5Acβ2Me **3**, Figure 1B), based on its known inhibitory effect on CSS activity [27,28].

## 2. Results and Discussion

### 2.1. Structure Analysis of NmB CSS

**Figure 2:**
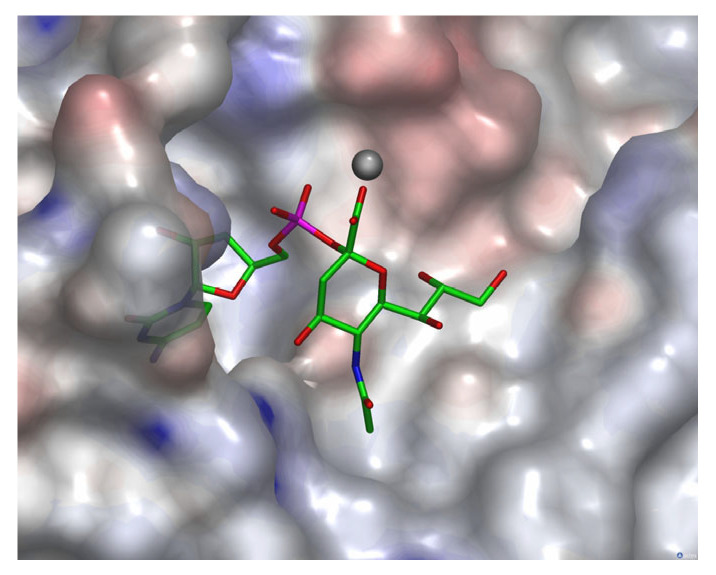
X-Ray crystal structure of CMP-Neu5Ac **2** in the active site of *Nm* CSS in an open form (PDB 6CKM) [44]. Solvent accessible surfaces are coloured according to charge (red, negative charge; blue, positive charge; grey, neutral regions). CMP-Neu5Ac is shown in stick representation (green). Bound Ca^2+^ (grey sphere). Visualized using OpenAstexViewer (Version 3.0) [50].

To decide on a rational basis which positions of Neu5Acβ2Me (**3**) would be most promising for modification, to potentially increase binding affinity and inhibitory effect, we began our study with an analysis of the available X-ray structural data for CSS. At the beginning of our studies, the X-ray crystal structure of *Nm*B CSS in complex with CDP as a substrate mimetic (PDB: 1EYR) was available [49]. This structure showed the enzyme as an asymmetric homodimer, with the active site present at the interface of the core domain of one subunit and the dimerization domain of the other subunit [49]. In 2020, X-ray crystal structures of *Nm*B CSS in complex with substrate CTP, and with the product of reaction CMP-Neu5Ac **2** (PDB: 6CKM; Figure 2) were reported [44], providing further insights into the catalytic cycle of the enzyme. From kinetic studies with active-site mutants and the X-ray crystallographic studies, it is apparent that the CSS active site switches between an open form for substrate binding (and product release), and a catalytically active ‘closed’ form [43,44,49].

It is clear that in the open form of the enzyme, there would be significant space around bound Neu5Ac. Substitutions on the Neu5Ac template, however, would no doubt have implications for the enzyme being able to close to give the correct positioning of the sialic acid and CTP for catalytic activity. The apparent extensive flexibility of the protein as it performs its function may limit a traditional molecular docking study. Instead, more complex techniques would need to be employed that would take into account protein flexibility, e.g., molecular dynamics simulations [51,52]. Our approach was therefore to systematically explore changes in spatial and functional group characteristics on the Neu5Ac template, based on analysis of contacts around Neu5Ac in the available models and crystal structures.

**Figure 3:**
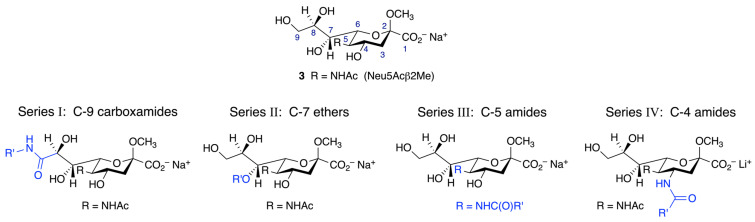
Overview of modifications incorporated onto the Neu5Acβ2Me (**3**) scaffold to explore interactions with *Nm* CSS.

In our original modelling study, and subsequently in the *Nm*B CSS–CMP-Neu5Ac (**2**) complex [44] (Figure 2), the glycerol side-chain (C-7 to C-9 hydroxyl groups) of the Neu5Ac moiety is seen to project into a spacious channel or cleft. The potential binding environment of the Neu5Ac glycerol side chain contains a number of charged amino acid residues, including positively charged residues on one side of the channel. This suggested that the introduction of a negatively charged substituent at the terminus of the glycerol side chain was of particular interest. The scope for this modification could be explored through the synthesis of a C-9 carboxylic acid derivative that could then be further elaborated with various simple neutral amines and with amino acids to generate a series of C-9 carboxamide derivatives of Neu5Acβ2Me (Figure 3, Series I).

Activation of 7-deoxy-Neu5Ac by *Nm* CSS [33] indicates that the C-7 hydroxyl group is not essential for substrate binding. Inspection of *Nm* CSS crystal structures revealed that a phenylalanine residue is in proximity to the Neu5Ac C-7 hydroxyl group, suggesting that an opportunity for possible π-π interaction exists in this region. With this in mind, we chose to synthesize a number of 7-*O*-aryl (e.g., benzyl) and unsaturated-alkyl (e.g., allyl) ethers, as well as the ethyl and propyl ethers, to explore this possible binding domain (Figure 3, Series II).

Apparent spatial constraints around C-4 and C-5 of Neu5Ac in *Nm* CSS structures seem to imply there is little potential for further functionalization at these positions. However, there are extensive protein dynamics involved in the function of CSS [44,45,53], and reports of successful activation of various *N*-acylated neuraminic acids by *Nm*B CSS [29,30,31,32], and a number of C-4 modified Neu5Ac derivatives (including 4-acetamido-4-deoxy-Neu5Ac [54]) by mammalian CSS. Together, these indicate that some structural variation at positions C-5 and C-4 can be tolerated. We therefore chose to introduce separately at each position, amides with varying functionality, ranging from small alkyl chains to large hydrophobic groups, in order to investigate structure–activity relationships around these positions (Figure 3, Series III and IV). In summary, four series of novel functionalized Neu5Ac derivatives, as the β-methyl glycosides (Figure 3), were proposed to probe interactions with *Nm* CSS, introducing carboxamides at C-9, ethers at C-7, and amides at C-5 and C-4.

### 2.2. Synthesis of Functionalized Neu5Acβ2Me Derivatives

#### 2.2.1. Synthesis of C-9 Functionalized Neu5Acβ2Me Derivatives (Series I)

**Scheme 1:**
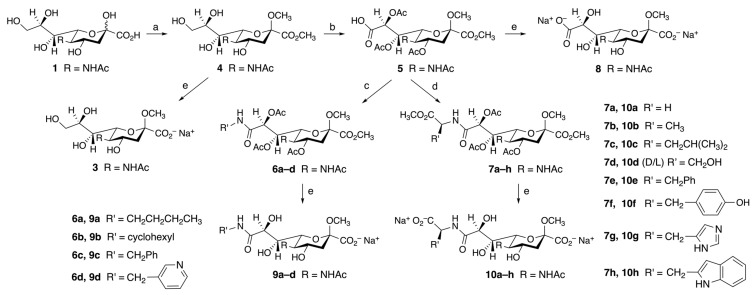
Synthesis of C-9 carboxamide derivatives of Neu5Acβ2Me. Reagents and conditions: (a) MeOH, H^+^ ion exchange resin, (i) reflux, 48 h (62%), or (ii) MW (100 W), 120 °C, 15 min (63%); (b) i. TEMPO, DCM, sat. aq. NaHCO_3_, KBr, Bu_4_NBr, aq. NaOCl, 0 °C to 5 °C, 1 h; ii. Ac_2_O, pyridine, rt, 16 h (66% over 2 steps) [59]; (c) R’NH_2_, HOBt, EDC, DIPEA, DCM/DMF (4:1), rt, 16 h (**6a** 60%, **6b** 60%, **6c** 56%, **6d** 55%); (d) R’C(NH_2_)CO_2_CH_3_, HOBt, EDC, DIPEA, DCM/DMF (4:1), rt, 16 h (**7a** 55%, **7b** 60%, **7c** 60%, **7d** 62%, **7e** 55%, **7f** 60%, **7g** 40%, **7h** 45%); (e) NaOH, MeOH/H_2_O (1:1), pH 13, 0 °C-rt, 16 h.

The starting point for the current studies was the synthesis of the β-methyl glycoside of *N*-acetylneuraminic acid methyl ester [(Neu5Ac1,β2Me_2_, **4**) from Neu5Ac **1** (Scheme 1). Methyl esterification of **1** and concomitant formation of the β-methyl glycoside can be achieved by heating **1** under reflux [55], or under microwave irradiation [56], with dry acidic ion exchange resin in anhydrous methanol. We have found [56] that while both approaches give comparable yields, microwave irradiation provides a more rapid reaction, and is more economical in terms of solvent and resin requirement than reaction under conventional heating. Saponification of **4** provided the benchmark β-methyl glycoside derivative, Neu5Acβ2Me, **3** [27,57,58].

To pursue modification at the C-9 position, we took advantage of the efficient synthesis of the C-9 carboxylic acid derivative of Neu5Ac2βMe [59], which could then be elaborated to generate a series of C-9 carboxamides. Regioselective TEMPO-mediated oxidation of the primary C-9 hydroxyl group of Neu5Ac2βMe methyl ester **4** was followed by peracetylation to assist in isolation and purification (Scheme 1). This gave the key intermediate 9-carboxy derivative **5** [59] in 66% yield over two steps. Compound **5** ultimately provides an interesting probe of CSS in its own right (see **8**), while the C-9 carboxy group of **5** provided us with the opportunity to further modify this position through coupling with amines of varying functionality. Elaboration of the carboxylic acid was carried out by reaction with neutral amines, as well as carboxy-protected amino acids. The latter would, upon deprotection, provide a negative charge at the end of the glycerol side-chain that may engage binding with positively charged protein residues.

The amide coupling was achieved using a slight modification of an established method for coupling the C-1 carboxyl group of Neu5Ac to amino acid esters [60,61], with the addition of 1-hydroxybenztriazole (HOBt) to minimize racemization and side reactions. 1-Ethyl-3-(3-dimethylaminopropyl) carbodiimide (EDC), which gives a water-soluble urea byproduct that can be easily removed by aqueous work-up, was used in place of (benzotriazol-1-yloxy)-tris(dimethylamino)phosphonium hexafluorophosphate (BOP reagent) because of the potential hazard of HMPA formation associated with coupling using the latter reagent [62]. 9-Carboxy derivative **5** was coupled with a series of aliphatic amines, and with a range of L-amino acid methyl esters (with the exception of D-/L-serine methyl ester), to give carboxamides **6a**–**d** and **7a**–**h**, respectively, in moderate yields (Scheme 1). De-*O*-acetylation followed by saponification yielded the 1,9-dicarboxylic acid derivative **8** [59], and carboxamides **9a**–**d** and **10a**–**h**. For the serine amino acid carboxamide **10d**, prepared from racemic D-/L-serine methyl ester, two isomers were separable by HPLC (denoted **10d-1** and **10d-2**); however, the relative stereochemistries at the serine a-carbon were not determined.

#### 2.2.2. Synthesis of C-7 Functionalized Neu5Acβ2Me Derivatives (Series II)

**Scheme 2:**
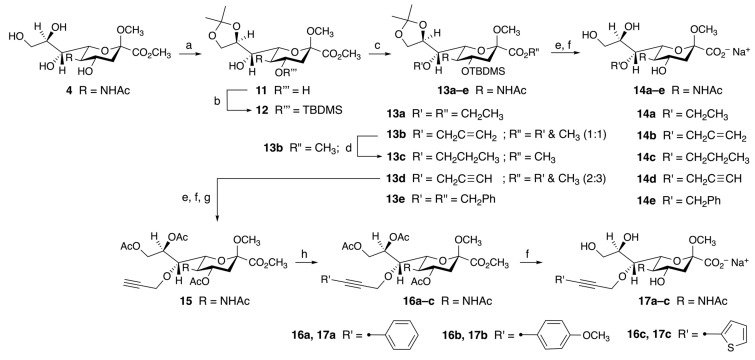
Synthesis of 7-*O*-alkylated derivatives of Neu5Acβ2Me. Reagents and conditions: (a) 2,2-dimethoxypropane, H^+^ ion exchange resin, acetone, rt, 16 h (90%); (b) *t*-BuMe_2_SiCl, pyridine, 0 °C-rt, 16 h (58%); (c) R^1^X, NaH, TBAI, DMF, 0 °C-rt, 16 h (**13a** 56%, **13b** 60%, **13d** 60%, **13e** 36%); (d) H_2_, 10% Pd/C, MeOH, rt, 16 h (**13c** 87%); (e) 80% aq. AcOH, 80 °C, 2 h; (f) NaOH, MeOH/H_2_O (1:1), pH 13, 0 °C-rt, 16 h; (g) (i) MeOH, H^+^ ion exchange resin, MW (100 W), 80 °C, 15 min; (ii) Ac_2_O, pyridine, 16 h (88% over 4 steps from **13d**); (h) R’I, PdCl_2_(PPh_3_)_2_, CuI, Et_3_N, CH_3_CN, rt, 2–6 h (**16a** 60%, **16b** 87%, **16c** 85%). In **16**,**17a–c**, the sphere indicates the point of attachment of R' to the alkyne carbon.

For the synthesis of the target Neu5Acβ2Me C-7 ethers (Series II), we utilized a route analogous to that reported by Masuda and coworkers [63,64,65] (Scheme 2). To isolate the relatively unreactive C-7 hydroxyl group, Neu5Ac1,β2Me_2_ **4** was reacted with 2,2-dimethoxypropane under acid catalysis to give the 8,9-*O*-isopropylidenated derivative **11** [66] in 90% yield. Regioselective monosilylation of the C-4 hydroxyl group then afforded 4-*O*-*t*-butyldimethylsilyl-8,9-*O*-isopropylidene derivative **12** [67]. The C-7 hydroxyl group of **12** was successfully alkylated using ethyl iodide (**13a**), allyl bromide (**13b**), propargyl bromide (**13d**), and benzyl bromide (**13e**) in the presence of NaH in DMF, to afford the corresponding alkyl ethers in moderate yield (36–60%). Use of ethyl iodide gave a comparable yield of **13a** to that reported when using diethylsulfate as the alkylating agent [64]. Higher equivalents of base were used to overcome the low reactivity of the C-7 hydroxyl group, which resulted in partial to complete transesterification in each reaction. Advantage was taken of the 7-*O*-allyl derivative **13b**, to prepare the 7-*O*-propyl derivative **13c** [63,64] by hydrogenation. Finally, simultaneous deprotection of both acid-labile groups (isopropylidene and TBDMS) of compounds **13a**–**e** by heating with 80% acetic acid at 80 °C, followed by saponification, gave the target 7-*O*-alkylated Neu5Acβ2Me derivatives **14a**–**e**.

The C-7 propyne ether derivative **13d** provided us with the opportunity to utilize Sonogashira coupling to further diversify the C-7 substituent. In order to achieve coupling selectively at the C-7 propyne ether, a sequence of deprotection and re-protection was employed on **13d** to remove the propyne ester functionality, and subsequently produce the acetylated methyl ester derivative **15** (Scheme 2). Following the method of Sato and coworkers [68], compound **15** was treated with aryl iodides in the presence of PdCl_2_(PPh_3_)_2_, CuI, and Et_3_N in anhydrous CH_3_CN to yield the disubstituted alkyne derivatives **16a**–**c** in good yield (60–87%). Upon saponification, these afforded the target compounds **17a**–**c**.

#### 2.2.3. Synthesis of C-5 Functionalized Neuβ2Me Derivatives (Series III)

**Scheme 3:**
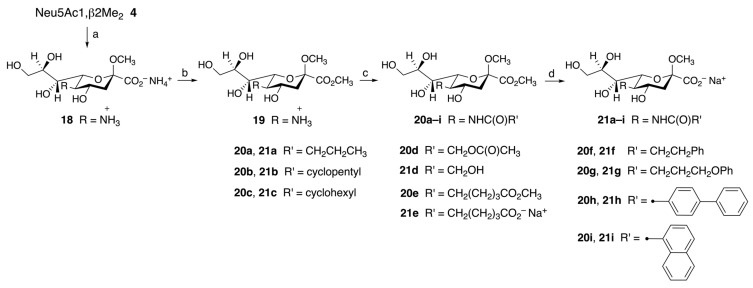
Synthesis of C-5 amide derivatives of Neuβ2Me. Reagents and conditions: (a) NaOH (2.0 M), MW (100 W), 120 °C, 15 min (80%) [71]; (b) SOCl_2_, MeOH, 0 °C-rt, 16 h (92%); (c) R’COCl, Et_3_N, 1,4-dioxane/water (5:1), 0 °C–40 °C, 2 h (**20a** 87%, **20b** 90%, **20c** 91%, **20d** 91%, **20e** 78%, **20f** 80%, **20g** 82%, **20h** 80% and **20i** 88%); d) NaOH, MeOH/H_2_O (1:1), pH 13, 0 °C-rt, 16 h. In **20**,**21h,i**, the sphere indicates the point of attachment of R' to the amide carbon.

For the synthesis of compounds of Series III, which have alternative acyl groups on the amine at C-5, basic hydrolysis of the C-5 acetamido group of **4** was utilized to obtain key 5-amino derivative **18** (Scheme 3). Instead of using conventional heating, which requires long reaction times [69,70], in this work, we took advantage of reaction under microwave irradiation to provide a method for rapid de-*N*-acetylation of **4** [71]. Under microwave irradiation (max. 100 W) at 120 °C, NaOH-promoted de-*N*-acetylation of **4** was complete in only 15 min, with the 5-amino derivative **18** obtained cleanly in 80% yield. The carboxylate group of **18** was then re-esterified under acidic conditions to give methyl ester **19**. *N*-Acylation of the C-5 amino group of **19** was performed with acid chlorides of varying functionality using triethylamine as base in water/dioxane (1:5) to provide the desired C-5 amide derivatives **20a**–**i** in high yields (78–91%). In contrast to an earlier reported method [72], no bis-acylation was observed. Finally, saponification afforded the target variously *N*-acylated Neuβ2Me derivatives **21a**–**i**.

#### 2.2.4. Synthesis of C-4 Functionalized Neu5Acβ2Me Derivatives (Series IV)

**Scheme 4:**
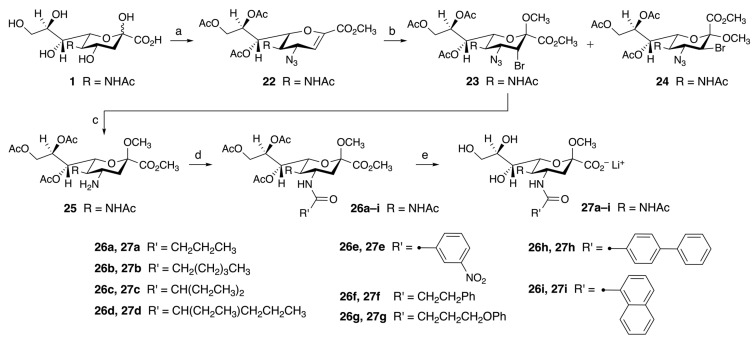
Synthesis of C-4 amide derivatives of 4-deoxy-Neu5Acβ2Me. Reagents and conditions: (a) over 4 steps according to the reported procedures: (i) MeOH, H^+^ ion exchange resin, rt, 16 h; (ii) Ac_2_O, pyridine, rt, 16 h; (iii) TMSOTf, EtOAc, 50 °C, 2 h; (iv) TMSN_3_, *t*-BuOH, reflux, 6 h [75]; (b) according to the reported procedure: NBS, MeOH, 0–5 °C, 72 h (**23 + 24**, 87%) [77]; (c) Bu_3_SnH, AIBN, dioxane, 65 °C, 16 h (77%) [77]; (d) R’COCl, Et_3_N, DCM, 0 °C-rt, 2 h (**26a** 55%, **26b** 67%, **26c** 60%, **26d** 65%, **26e** 65%, **26f** 65%, **26g** 65%, **26h** 60%, **26i** 65%); (e) LiOH, MeOH/H_2_O (1:1), pH 13, 0 °C-rt, 16 h. In **26**,**27e,h,i**, the sphere indicates the point of attachment of R' to the amide carbon.

The synthesis of the C-4 amide derivatives of Neu5Acβ2Me (Series IV) required the introduction of an amino group at C-4 in the equatorial configuration. Nitrogen functionality can be introduced at C-4 on a suitably protected Neu5Ac template via a 3-step sequence of oxidation of the equatorially substituted C-4 hydroxyl group, reduction in the 4-oxo derivative to the 4-*epi*-OH derivative, and finally introduction of azide in the equatorial configuration via a Mitsunobu reaction using HN_3_.[54] An alternative approach is to proceed via the 2,3-unsaturated sialic acid, Neu5Ac2en, scaffold, where the 4-azido derivative **22** [73,74,75] can be produced in good yield over four steps from Neu5Ac **1** [75]. Having **22** at hand, we chose the second approach (Scheme 4). Bromo-methoxylation [76] of the 2,3 double bond of 4-azido-4-deoxy-Neu5Ac2en derivative **22** gives rise to an approximately 1:1 isomeric mixture of 2,3-diaxial (**23**) and 2,3-diequatorial (**24**) products [77]. Treatment of the 2,3-di-axial bromomethoxylated isomer (the β-methyl glycoside) **23** with tributyltin hydride in the presence of AIBN [77] resulted in both debromination at C-3 and reduction in the C-4 azide, to give 4-amino Neu5Acβ2Me derivative **25**. Treatment of **25** with a range of acid chlorides afforded the C-4 amide derivatives **26a**–**i**, which, after global deprotection, gave the target 4-acylamino-4-deoxy-Neu5Acβ2Me derivatives **27a**–**i**.

### 2.3. Screening of Functionalized Neu5Acβ2Me Derivatives for Inhibition of NmB CSS

**Figure 4:**
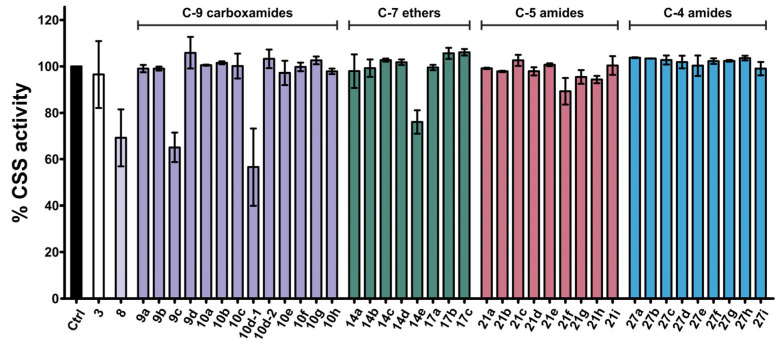
Relative activity of *Nm*B CSS in the presence of C-9, C-7, C-5, and C-4 functionalized Neu5acylβ2Me derivatives (at 100 μM) in the EnzChek^®^ pyrophosphate assay (Life Technologies, Darmstadt, Germany). Bar graphs represent the means of two individual experiments, with error bars representing the standard deviation. Activity of *Nm*B CSS in the absence of functionalized compounds was defined as 100% (Ctrl).

To analyze the effect of the compounds on *Nm*B CSS activity in vitro, we recombinantly expressed and purified the enzyme to homogeneity. The EnzChek™ pyrophosphate assay kit (Life Technologies, Darmstadt, Germany) was used to determine *Nm*B CSS activity in the forward reaction (i.e., formation of CMP-Neu5Ac and PPi), revealing *K*_m_ values of 22 and 37 µM for CTP and Neu5Ac, respectively. After confirming that the test compounds did not show any inhibitory effect on the assay components, we screened all functionalized Neu5acylβ2Me derivatives and the parent compound **3** at a concentration of 100 μM (Figure 4). Interestingly, the benchmark parent compound, unsubstituted β-methyl glycoside **3**, previously reported [27] to be a modest inhibitor of mammalian CSS (*K*_i_ = 2.5 mM), showed no significant inhibitory effect on *Nm*B CSS in this in vitro assay (Figure 4). This difference might be attributed to differences in the employed CSS species and/or in assay conditions: while Zbiral and coworkers [27] utilized the Warren assay [78] under CTP-limiting conditions, we provided both substrates in saturating concentrations of 1 mM each. Three of the C-9 modified Neu5Acβ2Me derivatives—parent 9-carboxy-Neu5Acβ2Me **8** [59], benzamide **9c**, and serine carboxamide **10d-1**—and one of the C-7 ethers—7-*O*-benzyl-Neu5Acβ2Me **14e**, however, showed inhibition of *Nm*B CSS activity by more than 20% in this screen. Detailed kinetic analysis of these four compounds suggests a mixed-mode inhibition that most closely resembles the non-competitive mode, as indicated by α-values close to 1 and supported by the appearance of Lineweaver-Burk plots (Supplementary Figure S1). As all compounds were designed to target the active site and compete with the natural substrate Neu5Ac **1** for binding, the finding that none of the four characterized inhibitors display competitive kinetics may be somewhat unexpected, but does not necessarily indicate binding outside of the catalytic center. Indeed, multiple mechanisms exist that may account for non-competitive or “mixed-mode mimicking” inhibition patterns of inhibitors that in fact bind to the active site [79,80]. One of these mechanisms applies to multisubstrate/-product enzymes that follow a sequential ordered mechanism, which is the case for CSS, where CTP binds prior to Neu5Ac [44]. With inhibitory constants (*K*_i_) in the range between 100 and 500 μM, these compounds appear to be stronger inhibitors than the previously reported sulfone-based nucleotide isosteres, which inhibited *Nm*B CSS activity up to 55% at a concentration of 1 mM [42].

### 2.4. Interaction of Neu5Acβ2Me C-9 Serine Carboxamide 10d-1 with NmB CSS as Determined by STD NMR

To confirm direct binding and to investigate the interactions of the most promising C9 carboxamide derivative **10d-1** with CSS, we used Saturation Transfer Difference (STD) NMR spectroscopy [46,81,82]. This powerful tool allows evaluation of ligand–biomolecule interactions by mapping the binding epitope of the ligand, essentially through screening the protons of the ligand for their proximity to the protein upon binding.

**Figure 5:**
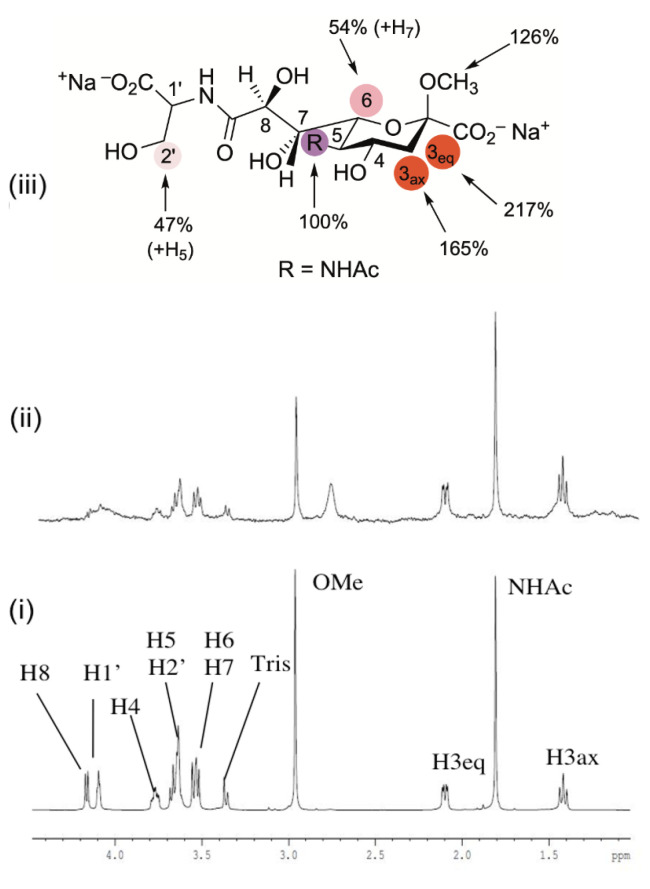
^1^H NMR and STD NMR spectra of Neu5Acβ2Me C-9 serine carboxamide **10d-1** with *Nm*B CSS. (**i**) ^1^H NMR spectrum of **10d-1**; (**ii**) STD NMR of **10d-1** with *Nm*B CSS (ligand to protein ratio of 100:1); (**iii**) epitope map of **10d-1**.

The binding epitope of **10d-1** in complex with *Nm*B CSS (Figure 5iii) was determined from the relative strength of transferred saturation (with the effect at the C-5 NHAc methyl group set to 100%). It is evident from the STD NMR spectrum (Figure 5ii) that the carbohydrate ring of **10d-1** is in close contact with the enzyme. Relatively strong STD NMR signals were observed for the equatorial proton at C-3 (H-3eq 217%), with more moderate signal intensities observed for the axial C-3 proton (H-3ax 165%) and the glycoside methyl group (126%). Interaction of the serine ‘tail’ could not be unequivocally determined due to the signal overlap, for example, of H-2′ with H-5; however, any interaction would appear to be only weak.

### 2.5. Neu5Acβ2Me C-9 Serine Carboxamide **10d-1** as a Modulator of NmB LOS Sialylation

**Figure 6:**
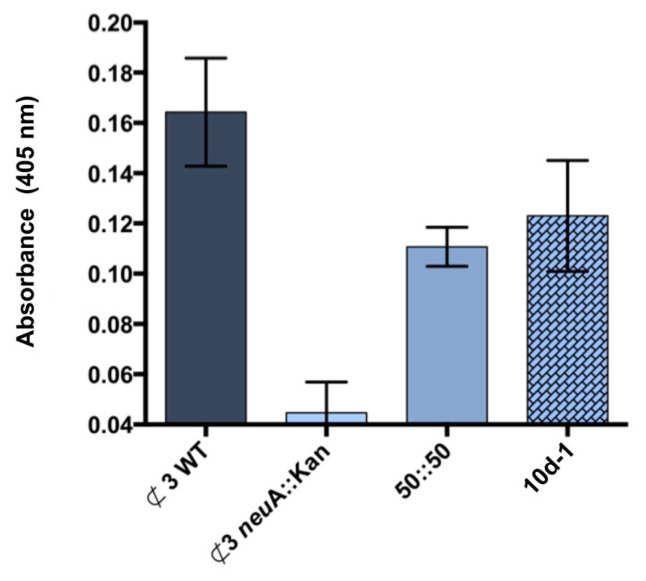
ELISA to measure the effect of **10d-1** on *N. meningitidis* LOS sialylation. ¢3 WT (capsule–deficient *N. meningitidis* wild type), ¢3*neuA::kan*: (*CSS-deficient* ¢3), and a 1:1 ratio of ¢3 WT and ¢3*neuA::kan* (50::50) were coated on microtiter plates and surface sialic acid detected photometrically (405 nm) with the sialic-acid-specific lectin MAA-AP conjugate. Similarly, ¢3 wild type was incubated with **10d-1** at 1 mM final concentration for 4 h, after which surface sialic acid was detected. Absorbance at 405 nM was measured to detect *p*-nitrophenol released from the alkaline phosphatase substrate *p*-nitrophenyl phosphate (PNPP). Experiments were performed in triplicate.

To determine whether Neu5Acβ2Me C-9 serine carboxamide **10d-1** was capable of inhibiting CSS (encoded by the gene *neuA*) in living bacteria, we established an ELISA-based assay utilizing a sialic acid-recognizing lectin to detect LOS sialylation on the cell surface of *Nm*B. The capsule-deficient *N. meningitidis* strain ¢3 (¢3 WT) [13] expressed sialylated LOS at high levels (defined as 100%, Figure 6). To define the background signal in the absence of *neuA* activity, we generated a corresponding *neuA* knockout strain (¢3*neuA::kan*) and observed a reduction in LOS sialylation to about 2%. Complementation of the *neuA* knockout mutant by reintroducing the *neu*A gene (¢3*neuA::kan*, NeuA+) confirmed that loss of signal in the assay was due to inactivity of CSS and thus, loss of sialylation in this mutant (Supplementary Figure S2). To evaluate whether the results of this assay can be interpreted in a quantitative manner, we mixed ¢3 wild type strain and ¢3*neuA::kan* knockout mutant in a 1:1 ratio. This resulted in a ~44% ± 6% reduction in signal intensity, indicating a corresponding reduction in LOS sialylation of wild-type *Nm,* which was close to ~50%. Given that not all ¢3 wild-type LOS molecules are sialylated, a value of under 50% is reasonable (Supplementary Figure S2). We then tested the effect of C-9 serine carboxamide **10d-1** on LOS sialylation by supplementing the growth medium with 1 mM of the compound. The ELISA results indicated that bacterial growth in the presence of **10d-1** resulted in a reduction in *Nm*B LOS sialylation to ~66% of ¢3 wild wild-type level (Figure 6). This suggests that inhibitor **10d-1** is able to cross the cell membranes and act on CSS, leading to a decrease in cell surface sialylation of whole cells.

## 3. Materials and Methods

### 3.1. Modelling of Neu5Ac 3 in the Active Site of NmB CSS

The PyMOL Molecular Graphics System (Version 1.7.x Schrödinger, LLC, New York, NY, USA) was used for modelling.

### 3.2. Chemical Synthesis—Materials and Methods

*N*-Acetylneuraminic acid (5-acetamido-3,5-dideoxy-d-*glycero*-d-*galacto*-non-2-ulosonic acid) (**1**) was obtained from Jülich Chiral Solutions GmbH (Jülich, Germany) and Carbosynth Ltd. (Compton, UK). Reagents and dry solvents purchased from commercial sources were used without further purification. Anhydrous reactions were carried out under an atmosphere of nitrogen or argon, using oven-dried glassware. Microwave reactions were conducted using a CEM Discover^®^ SP Explorer Hybrid-12 (CEM Corporation, Matthews, NC, USA) microwave system with a single-mode cavity, and were carried out in a 10 mL pressure tube, sealed with a Teflon septum.

Reactions were monitored using thin-layer chromatography (TLC) on aluminum plates precoated with Silica Gel 60 F254 (E. Merck, Darmstadt, Germany). Developed plates were observed under UV light at 254 nm and then visualized after application of a solution of H_2_SO_4_ in EtOH (5% *v*/*v*) or ninhydrin in EtOH (0.2% *v*/*v*), as appropriate, and heating. Flash chromatography was performed on silica gel 60 (0.040–0.063 mm) or using a Reveleris^®^ flash chromatography system (Grace Davison, Columbia, MD, USA) (as indicated) using distilled solvents.

^1^H and ^13^C NMR spectra were recorded either at 600 or 300 MHz and 150 or 75.5 MHz, respectively, on a Bruker Avance 600 or 300 MHz spectrometer (Bruker, Rheinstetten, Germany; as indicated). Low-resolution mass spectra (LRMS) were recorded, in electrospray ionization mode, on a Bruker Daltonics Esquire 3000 ESI spectrometer (Bruker, Bremen, Germany), using positive or negative mode (as indicated). High-resolution mass spectrometry (HRMS) was carried out by the Griffith University FTMS Facility on a Bruker Daltonics Apex III 4.7e Fourier Transform MS, fitted with an Apollo ESI source, or by the University of Queensland MS Facility on a Bruker MicrOTOF-Q with a Bruker ESI source (Bruker, Bremen, Germany).

HPLC purification was performed on an Agilent HP1100 instrument (Agilent, Santa Clara, CA, USA) using a Phenomenex Aqua 5 μ C18 124 Å column (250 × 10 mm) (Phenomenex, Torrance, CA, USA) at a flow rate of 3 mL/min and column temperature of 40 °C using isocratic elution with solvents as indicated. The purities of all synthetic intermediates after chromatographic purification were judged to be >90% by ^1^H and ^13^C NMR. The purity of tested compounds was ≥95% by HPLC analysis or by ^1^H and ^13^C NMR.

Detailed synthetic methods for all test compounds and intermediates [83] are provided in the Supporting Information—Part 1. NMR spectra (^1^H and ^13^C) of all test compounds and intermediates [83] are provided in the Supporting Information—Part 2.

### 3.3. Protein Expression and Purification (For Screening Assay and STD NMR)

A modified pET22b-Strep vector (IBA Lifesciences, Göttingen, Germany) allowing the prokaryotic expression of *Nm*B CSS (accession No. M95053) was kindly provided by PD Dr. Martina Mühlenhoff, MHH, Germany. Recombinant *Nm*B CSS with an N-terminal StrepII-tag was expressed in *E. coli* BL21(DE3) (Novagen, registered trademark of Merck, Darmstadt, Germany) at 15 °C and purified by affinity chromatography utilizing the StrepII-Tag. Peak fractions were desalted (HiPrep 26/10, GE Healthcare/now Cytiva, Freiburg, Germany) and concentrated in buffer containing 50 mM Tris-HCl, pH 8, 20 mM MgCl_2_, 150 mM NaCl, and 1 mM DTT to 2.5 mg/mL. Protein concentrations were determined by measuring the absorption at 280 nm under consideration of the specific extinction coefficients calculated at https://web.expasy.org/protparam/ (accessed on 15 January 2020). Purified protein samples were flash-frozen in liquid nitrogen and stored at −80 °C until required.

### 3.4. In Vitro Inhibition Assay and Kinetic Characterization

For initial screening of the synthesized compounds, CSS in vitro activity in the absence or presence of synthesized compounds was measured in 96-well half-area plates (Greiner Bio-One, Frickenhausen, Germany) in a reaction volume of 100 μL, utilizing the EnzChek pyrophosphate assay kit (Life Technologies, Darmstadt, Germany). Briefly, the inorganic pyrophosphate (PPi), produced by CSS as a byproduct, is cleaved by inorganic pyrophosphatase (IPP), yielding two molecules of phosphate (Pi), which subsequently react with 2-amino-6-mercapto-7-methylpurine ribonucleoside (MESG) in a reaction catalyzed by purine nucleoside phosphorylase (PNP). Detection of the reaction product 2-amino-6-mercapto-7-methylpurine at 360 nm allows quantitation of the initially generated Pi. The assay reaction mixture contained 50 mM Tris-HCl, pH 8.0, 25 mM MgCl_2_, 1 mM Neu5Ac (Nacalai Tesque, Kyoto, Japan), 1 mM CTP (Sigma-Aldrich, Taufkirchen, Germany), as well as 0.22 mM MESG, 1 U/mL PNP, and 0.03 U/mL IPP (all from Life Technologies), and 0.1 mM of the compounds to be tested. To exclude an effect of the compounds on the coupling enzymes PNP and IPP, a control assay without Neu5Ac, CTP, and CSS was performed, and the reaction was instead initiated by the addition of inorganic pyrophosphate (Sigma-Aldrich) at 20 μM final concentration. For CSS activity measurements, the reaction was initiated by the addition of purified *Nm*B CSS at a final concentration of 157.5 ng/mL. The enzyme was diluted in 50 mM Tris-HCl, pH 8.0, 25 mM MgCl_2,_ and briefly pre-incubated with the compounds before transferring the CSS/compound mixture to the reaction mixture with a 96-well pipette. Product formation was monitored at 360 nm in a 96-well plate reader at 25 °C for 4 min. As a reference, CSS activity without compounds was measured and defined as 100% activity. Activity in the presence of each compound was calculated as a percentage of the reference activity.

For kinetic characterization, the CSS in vitro activity assay was carried out as described above, but with Neu5Ac and effector compound concentrations varied between 0.01 and 1.0 mM and 0–0.4 mM, respectively, while CTP concentration was maintained at 1 mM. All measurements were performed in duplicate. Uninhibited CSS activity was used to calculate kinetic parameters for the *Nm*B enzyme by nonlinear regression according to the Michaelis–Menten model. Inhibitory constants were determined by plotting CSS activity in the presence of different compound concentrations against Neu5Ac concentration, and performing nonlinear regression curve fitting according to different inhibition modes (competitive, non-competitive, uncompetitive, and mixed-mode). Lineweaver–Burk plots were generated by plotting the reciprocal values of enzyme activity (1/v) against substrate concentration (1/[s]). The displayed linear regression lines with x- and y-intercepts at −1/*K*_m_ and 1/*V*_max_, respectively, were constructed based on the corresponding kinetic parameters obtained from nonlinear regression analysis of the original data. All kinetic analyses were carried out using GraphPad Prism version 5.01 for Windows (GraphPad Software, Boston, MA, USA, www.graphpad.com).

### 3.5. STD NMR Spectroscopy

Saturation Transfer Difference (STD) NMR experiments were performed on a Bruker Avance 600 MHz NMR spectrometer, equipped with a 5 mm TXI probe with triple-axis gradient at 298 K [83]. The protein was saturated on-resonance at −1 ppm and off-resonance at 33 ppm with a cascade of 40 selective Gaussian-shaped pulses, of 50 ms duration with a 100 μs delay between each pulse in all STD NMR experiments. The total duration of the saturation time was set to 2 s. For the STD NMR experiments, 23.3 μM *Nm*B CSS in deuterated 50 mM TRIS_D5_, 50 mM NaCl, and 5 mM MgCl_2_ at pH 8.0 was used. The test compound was added in a molecular protein:ligand ratio of 1:100. A total of 1024 scans per STD NMR experiment were acquired, and a WATERGATE sequence was used to suppress the residual HDO signal. A spin-lock filter with a strength of 5 kHz and a duration of 10 ms was applied to suppress protein background.

### 3.6. ELISA-Based Assay to Monitor Sialylation of NmB LOS

Bacterial strains, plasmids, media, and growth conditions: *Neisseria* strains were grown on brain heart infusion (BHI, Oxoid; Thermo Scientific, Scoresby, Australia) agar plates made with 1% agar and supplemented with 10% Levinthal base [84] at 37 °C with 5% CO_2_. *E. coli* strain DH5α was grown on Luria–Bertani agar plate or broth [85]. Antibiotics ampicillin (amp) and kanamycin (kan) were used at a final concentration of 100 μg/mL where appropriate.

Construction of mutant strains: A PCR product encompassing 1 kb upstream and downstream of the sequence flanking the CMP-Neu5Ac synthase gene (*neuA*, NMB0069) was amplified from *Neisseria* strain MC58 using primers containing *Neisseria* uptake sequence; NeuAMC58_FOR (5′-GCCGTCTGAACAGAACCTACAAGGAAGTAAC-3′) and NeuAMC58_REV (5′- GACGCT GAAGTCTCCATTG-3′) and cloned into pGEM-T-Easy (Promega, Madison, WI, USA). Expression of *neuA* was disrupted through the insertion of a kanamycin resistance cassette (Kan^R^) from the pUC4Kan plasmid (Cytiva (formerly GE Healthcare, Amersham Biosciences), USA) into a *Bam*HI restriction site. The resulting mutant construct named pGEM-T::*neuA*::Kan^R^ was linearized with *Nco*I-HF and transformed into *Neisseria* strain MC58 ¢3 as described previously [86]. The new construct was named (¢3*neuA::kan*). Orientation of the insert into the vector was verified through sequencing and the generation of a unique PCR product with primers NeuABMC58_REV and KanUP_OUT (5′-AGACGTTTCCCGTTGAATATGGTCAT-3′), a primer located within the Kan^R^ cassette.

Complementation of the siaB gene: The *neuA* mutant was complemented as described previously [87]. A PCR product of *siaB* with 300 bp upstream and downstream flanking region was amplified using primers with added *random bases* and restriction enzyme sequence; NeuA_COMP_FOR (5′-*ACATCG***CTTAAG** GCAACTCAAGTGCAGGTATTAG-3′) (*Afl*II restriction enzyme sequence) and NeuA_COMP_REV (5′-*GTCGTA***CCCGGG**CTTCTTCATTCAGGGCGCAAC-3′) (*Sma*I restriction enzyme sequence). This insert was cloned into the corresponding *Afl*II and *Sma*I digested pCTS32 vector under the control of the vector promoter. The subsequent construct, named ¢3*neuA::kan*, NeuA+, was linearized with *Sal*I and transformed into ¢3*neuA::kan.* Clones were selected on BHI plates supplemented with 10% Levinthal base and antibiotics ampicillin (100 μg/mL) and spectinomycin (50 μg/mL), respectively. Correct recombination of *siaB* into the chromosome was verified by PCR.

ELISA-based LOS sialylation assay: *Neisseria* strains were plated out on BHI plates (supplemented with antibiotics where necessary) and incubated at 37 °C + 5% CO_2_ overnight. Cell suspension was prepared from overnight growth in 2X BHI supplemented with levinthal, and normalized to an OD600 of 1.0.

Test compound was suspended in 300 μL of sterile dH_2_O to give a final concentration of 1 mM and filter sterilized. Standing cultures were grown with the test compound at a starting OD of 0.1 for 4 h at 37 °C, shaken at 200 rpm. After incubation, cells were washed and resuspended in PBS and normalized to an OD600 of 0.2. Cells were heat-killed for 1 h at 60 °C before being used for ELISA. A total of 50 μL of normalized heat-killed cells was added per well (Nunc, Thermo Scientific, Waltham, MA, USA), and the plates were dried overnight at room temperature. Wells were blocked with 5% BSA + 1× TBST overnight, followed by 1 h incubation with 100 μL of *Maackia amurensis* lectin–alkaline phosphatase conjugate (MAA-AP; EY Laboratories, San Mateo, CA, USA) in 1/1400 dilution. Wells were washed at 5 min intervals with 1× TBST, repeated 4 times, and developed with substrate *p*-nitrophenyl phosphate (PNPP, Thermo Scientific) for 30 min. Reaction was stopped with 50 μL of 1N NaOH, and absorbance was read at 405 nm. Experiments were performed in triplicate.

## 4. Conclusions

We have functionalized the C-9, -7, and -4 positions of Neu5Acβ2Me and varied the C-5 *N*-acyl substituent on Neuβ2Me to produce a series of compounds with varied substitution around the sialic acid framework to probe interaction with *N. meningitidis* CSS. Evaluation of the compounds for inhibition of *Nm*B CSS activity in vitro identified four compounds that showed an increased inhibition of the recombinant enzyme (*K*_i_ range between 100 and 500 μM), compared to the unsubstituted parent compound **3**. Importantly, the Neu5Acβ2Me C-9 serine carboxamide **10d-1** was shown to directly interact with *Nm*B CSS, to exhibit mixed mode inhibition of the enzyme, and to reduce the level of sialylation of *N. meningitidis* LOS. This data provides an intriguing starting point for further development of *Nm*B CSS inhibitors as novel and specific anti-microbial agents for the treatment of bacterial meningitis.

## Data Availability

The data presented in this study are included in the article and Supplementary Material. Further inquiries can be directed to the corresponding authors.

## Supplementary Materials

The following supporting information can be downloaded at: https://www.mdpi.com/article/10.3390/molecules30224329/s1: Supporting Information-S1, which includes: Figure S1, Kinetic analysis of test compounds with *Nm*B CSS; Figure S2, ELISA to measure *N. meningitidis* LOS sialylation; Detailed experimental procedures for synthesis of the reported functionalized sialic acids and their characterization, including NMR data. Supporting Information-S2: NMR spectra.

## Abbreviations

The following abbreviations are used in this manuscript:

CSS	CMP-sialic acid synthetase
LOS	lipooligosaccharide
*Nm*B	*Neisseria meningitidis* serotype B
STD	Saturation Transfer Difference (STD) NMR
