# Supplementary material for: Exploring the Scope of Functionalized N-Acylneuraminic Acid β-Methyl Glycosides as Inhibitors of Neisseria meningitidis CMP-Sialic Acid Synthetase
Source: Molecules. 2025 Nov 7;30(22):4329. doi: 10.3390/molecules30224329 (PMC12654856; doi:10.3390/molecules30224329)
Supplement: Supplementary file 1 [file molecules-30-04329-s001.zip › Supporting information-1.pdf]

Supporting Information – 1

Article

Exploring the Scope of Functionalized *N*-Acylneuraminic Acid β-Methyl Glycosides as Inhibitors of *Neisseria meningitidis* CMP-Sialic Acid Synthetase

Pradeep Chopra [1], Jana Führing [2], Preston Ng [1], Thomas Haselhorst [1], Jeffrey C. Dyason [1], Faith J. Rose [1], Robin J. Thomson [1], Rita Gerardy-Schahn [2], I. Darren Grice [1], Michael P. Jennings [1*], Anja K. Münster-Kühnel [2*] and Mark von Itzstein [1*]

1 Institute for Biomedicine and Glycomics, Griffith University, Gold Coast, Queensland 4222, Australia; pchopra@uga.edu (P.C.); t.haselhorst@griffith.edu.au (T.H.); r.thomson@griffith.edu.au (R.J.T.); d.grice@griffith.edu.au (I.D.G.)

2 Institut für Klinische Biochemie, Medizinische Hochschule, Carl-Neuberg-Straße 1, 30625 Hannover, Germany; fuehring.jana@mh-hannover.de (J.F.); gerardy-schahn.rita@mh-hannover.de (R.G.-S.)

* Correspondence: m.jennings@griffith.edu.au (M.P.J.); muenster.anja@mh-hannover.de (A.K.M.-K.); m.vonitzstein@griffith.edu.au (M.v.I.)

Table of Contents

Figure S1 – Kinetic analysis of test compounds with *Nm*B CSS S3
Figure S2 – ELISA to measure *N. meningitidis* LOS sialylation S4

Synthetic chemistry – Materials and methods. S5
Synthesis of C-9 carboxamide derivatives of Neu5Acβ2Me S6
Synthesis of C-7 ether derivatives of Neu5Acβ2Me S13
Synthesis of C-5 amide derivatives of Neu5acylβ2Me S18
Synthesis of C-4 amide derivatives of Neu5Acβ2Me S23
References S28

NMR Spectra (^{1}H and ^{13}C) of synthesized compounds: see Supporting Information – 2

Supplementary Figure 1

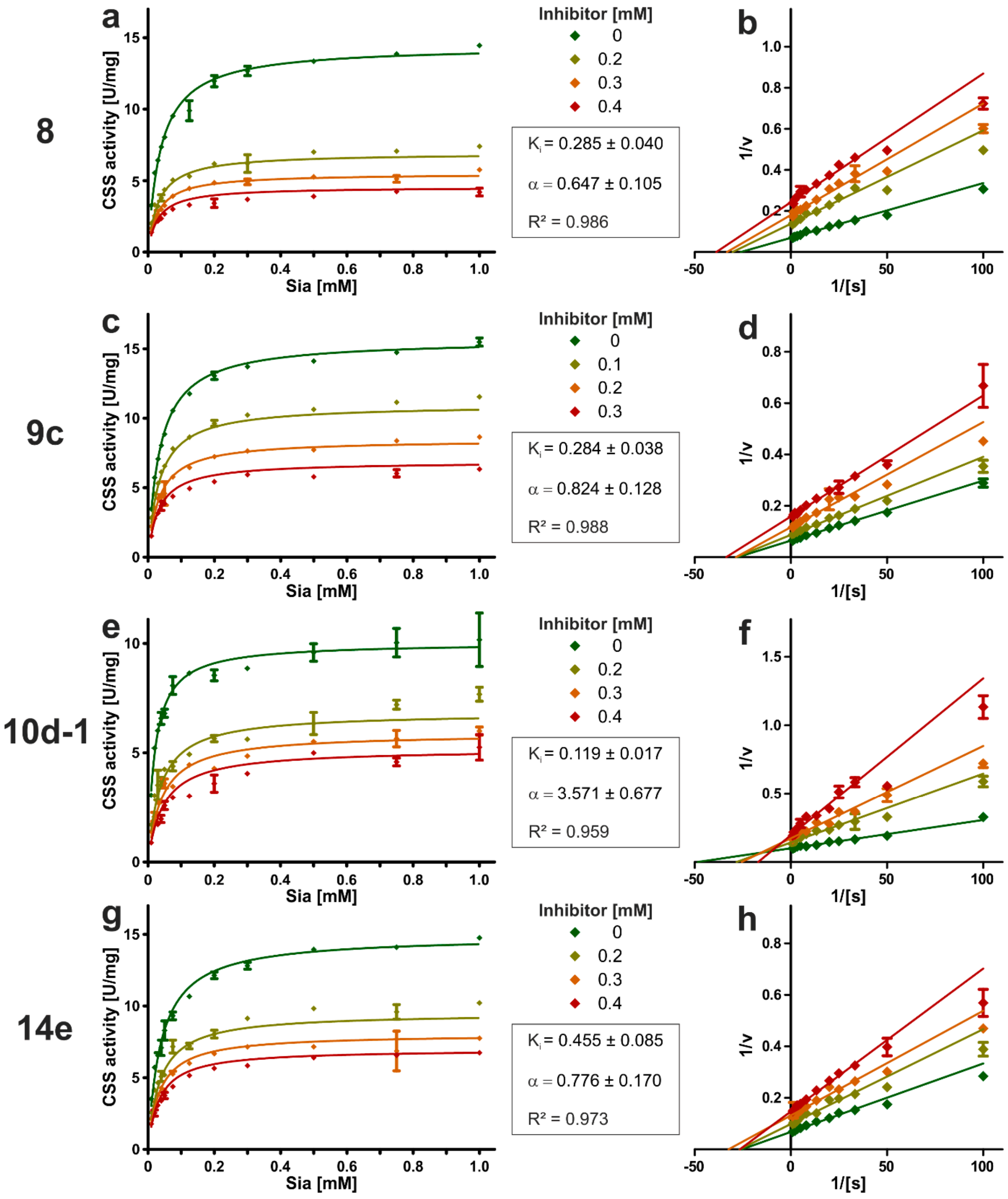

Figure S1. Kinetic analysis of test compounds with *Nm*B CSS

Figure S1. Analysis of the inhibitory effect of test compounds **8**, **9c**, **10d-1** and **14e** on *Nm*B CSS *in vitro* activity. Datapoints represent means of two replicates ± SEM. (a, c, e, g) CSS activity measured in the absence or presence of varying concentrations of the respective test compound was analyzed by nonlinear regression. The inhibitory constant (K_i), alpha value (α) and coefficient of determination (R^2), derived from nonlinear regression using the mixed model of inhibition, are given as insets. Values of α >>1, α = 1 or 0 < α << 1 indicate competitive, non-competitive or uncompetitive mechanisms of inhibition, respectively. (b, d, f, h) Graphical representation of the respective inhibitor kinetics as Lineweaver-Burk plots. Data were analyzed and visualized using GraphPad Prism version 5.01 for Windows (GraphPad Software, Boston, MA, USA, www.graphpad.com).

Supplementary Figure 2

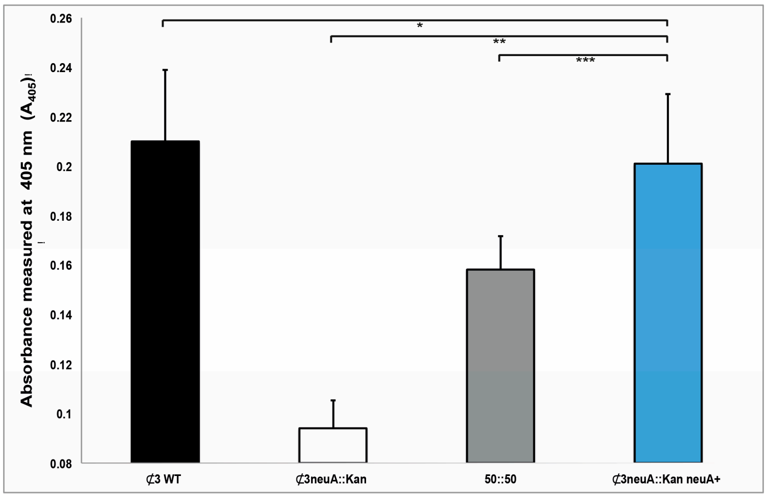

Figure S2. ELISA to measure *N. meningitidis* LOS sialylation. ¢3 wild type; ¢3*neuA::kan*; a 1:1 ratio of ¢3 wild type and ¢3*neuA::kan*; and a NeuA complemented strain, ¢3*neuA::kan,* NeuA+ were coated on microtitre plates and surface sialic acid detected photometrically with MAA-AP lectin conjugate. Absorbance at 405 nM was measured to detect *p*-nitrophenol released from the alkaline phosphatase substrate *p*-nitrophenyl phosphate (PNPP). Experiments were performed in triplicate. A T-test was used to calculated statistical significant of difference between ¢3 wild type and derived strains, indicated with asterisks above the samples; (*)(not significant), (**)(P = 0.0039) and (***)(P = 0.0758).

Experimental Procedures [1]

Synthetic chemistry – Materials and methods. *N*-Acetylneuraminic acid (5-acetamido-3,5-dideoxy-D-*glycero*-D-*galacto*-non-2-ulosonic acid) (**1**) was obtained from Jülich Chiral Solutions GmbH (Jülich, Germany) and Carbosynth Ltd (Compton, UK). Reagents and dry solvents purchased from commercial sources were used without further purification. Anhydrous reactions were carried out under an atmosphere of nitrogen or argon, using oven-dried glassware. Microwave reactions were conducted using a CEM Discover® SP Explorer Hybrid-12 microwave system with single mode cavity (CEM Corporation, Matthews, NC, USA). Microwave reactions were carried-out in a 10 mL pressure tube, sealed with a Teflon septum.

Reactions were monitored using thin layer chromatography (TLC) on aluminium plates precoated with Silica Gel 60 F254 (E. Merck, Darmstadt, Germany). Developed plates were observed under UV light at 254 nm and then visualized after application of a solution of H_2SO_4 in EtOH (5% v/v) or ninhydrin in EtOH (0.2% v/v), as appropriate, and heating. Flash chromatography was performed on silica gel 60 (0.040-0.063mm) or using a Reveleris® flash chromatography system (Grace Davison, Columbia, MD, USA) (as indicated) using distilled solvents.

^{1}H and ^{13}C NMR spectra were recorded either at 600 or 300 MHz and 150 or 75.5 MHz respectively on a Bruker Avance 600 or 300 MHz spectrometer (Bruker, Rheinstetten, Germany) (as indicated). Multiplicities are described using the abbreviation: s, singlet; d, doublet, t, triplet; q, quartet; m, multiplet; and app, apparent. Chemical shifts (δ) are reported in parts per million, relative to the residual solvent peak as internal reference [$CDCl_3$: 7.24 (s) for ^{1}H, 77.0 (t) for ^{13}C; CD_3OD: 3.30 (pent) for ^{1}H, 49.0 (sept) for ^{13}C; D_2O: 4.79 (s) for ^{1}H]. 2D COSY and HSQC experiments were run to support assignments. An asterisk (*) denotes assignments which are tentative. Substituents are assigned with the point of attachment designated as 'a'. Low-resolution mass spectra (LRMS) were recorded, in electrospray ionization mode, on a Bruker Daltonics Esquire 3000 ESI spectrometer (Bruker, Bremen, Germany), using positive or negative mode (as indicated). High-resolution mass spectrometry (HRMS) was carried out by the Griffith University FTMS Facility on a Bruker Daltonics Apex III 4.7e Fourier Transform MS, fitted with an Apollo ESI source or by the University of Queensland MS Facility on a Bruker MicrOTOF-Q with a Bruker ESI source. HPLC purification was performed on an Agilent HP1100 instrument (Santa Clara, CA, USA) using a Phenomenex Aqua 5 µ C18 124 Å column (250 × 10 mm) (Torrance, CA, USA) at a flow rate of 3mL/min and column temperature of 40 °C using isocratic elution with solvents as indicated. The purities of all synthetic intermediates after chromatographic purification were judged to be >90% by ^{1}H and ^{13}C NMR. The purity of tested compounds was ≥95% by HPLC analysis or by ^{1}H and ^{13}C NMR.

Methyl 5-acetamido-7,8,9-tri-*O*-acetyl-2,6-anhydro-4-azido-3,4,5-trideoxy-D-*glycero*-D-*galacto*-non-2-enonate (**22**) [2] and methyl (methyl 5-acetamido-7,8,9-tri-*O*-acetyl-4-azido-3-bromo-3,4,5-trideoxy-β-D-*erythro*-L-*manno*-non-2-ulopyranosid)onate (**23**) [3] were prepared according to literature procedures.

General procedure for base catalyzed de-*O*-acetylation and de-esterification. To a solution of *O*-acetylated methyl ester derivative in MeOH:H_2O (1:1) at 0 °C, was added aq NaOH (2.0 M) to give pH 13. The reaction mixture was stirred at rt and monitored by TLC (hexane/EtOAc, 1:5). After 16 h, the reaction mixture was acidified to pH 8 with Amberlite® IR-120 (H^+) resin, the resin was filtered-off and filtrate evaporated under reduced pressure to afford yellow syrup. The crude product was purified by column chromatography (EtOAc/MeOH/H_2O, 7:2:1) to give a solid residue, which was further purified by RP-HPLC and then lyophilized. Where TFA was used in the eluent during RP-HPLC purification, isolated sodium salts were converted to the free acid form.

Synthesis of C-9 carboxamide derivatives of Neu5Acβ2Me

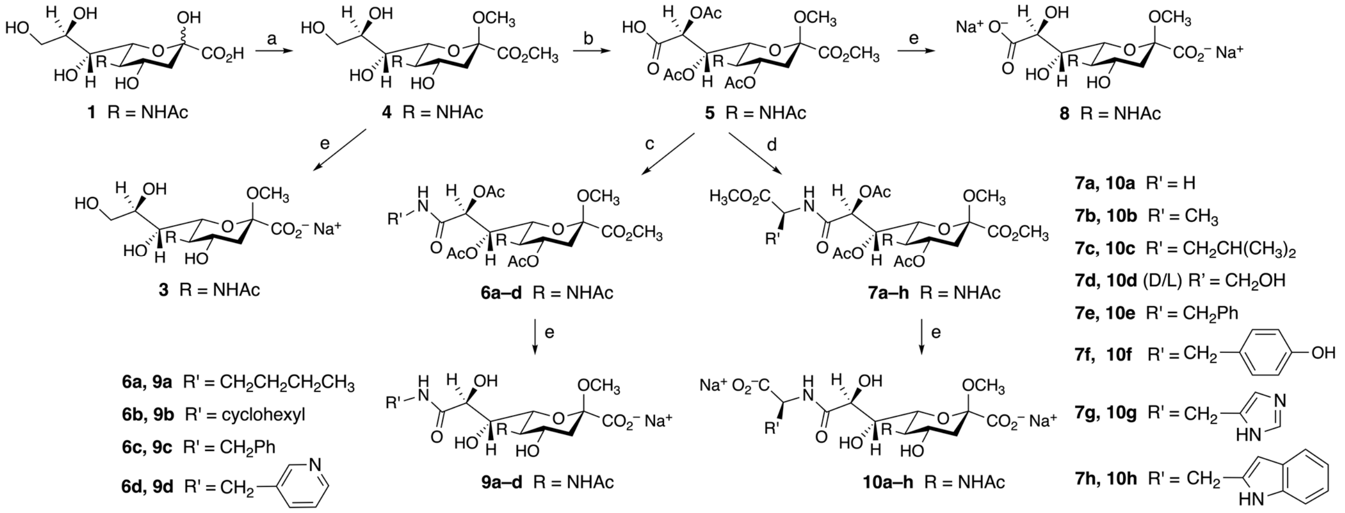

Scheme S1. Synthesis of C-9 carboxamide derivatives of Neu5Acβ2Me. *Reagents and conditions:* (a) CH_3OH, H^+ ion exchange resin, (i) reflux, 48 h (62%), or (ii) MW (100 W), 120 °C, 15 min (63%); (b) (i) TEMPO, DCM, sat. aq. $NaHCO_3$, KBr, Bu_4NBr, aq. NaOCl, 0 °C to 5 °C, 1 h; (ii) Ac_2O, pyridine, rt, 16 h (66% over 2 steps); (c) $R'NH_2$, HOBt, EDC, DIPEA, DCM/DMF (4:1), rt, 16 h (**6a** 60%, **6b** 60%, **6c** 56%, **6d** 55%); (d) $R'C(NH_2)CO_2CH_3$, HOBt, EDC, DIPEA, DCM/DMF (4:1), rt, 16 h (**7a** 55%, **7b** 60%, **7c** 60%, **7d** 62%, **7e** 55%, **7f** 60%, **7g** 40%, **7h** 45%); (e) NaOH, MeOH/H_2O (1:1), pH 13, 0 °C to rt, 16 h.

Methyl (methyl 5-acetamido-3,5-dideoxy-D-*glycero*-β-D-*galacto*-non-2-ulopyranosid)onate (4).

Method 1: A mixture of *N*-acetylneuraminic acid (**1**) (5.0 g, 16.18 mmol) and dry Dowex® 50Wx8 (H^+) resin (12.5 g) in anhydrous MeOH (250 mL) was refluxed for 48 h, then cooled to room temperature. The resin was then removed by filtration and the filtrate concentrated under reduced pressure to give a yellow syrup which was dissolved in a small volume of hot ethyl acetate-methanol (3:1, v/v). On standing in the cold, crystals were deposited which were separated and further washed with ethyl acetate to give **4** [4] as a white crystals (3.4 g, 62%).

Method 2: A mixture of *N*-acetylneuraminic acid (**1**) (1.0 g, 3.24 mmol) and dry Dowex® 50x8 (H+) resin (1.0 g) in anhydrous MeOH (25 mL) was microwave irradiated (max. power 100 W) for 15 minutes at 120 °C [5]. After completion of the holding time, the reaction mixture was cooled to room temperature, the resin was removed by filtration over Celite, and the filtrate was concentrated under reduced pressure to give a syrup. The crude product mixture was dissolved in a small volume of EtOAc–MeOH, adsorbed onto silica, and purified using a Reveleris® flash chromatography system [40 g column; flow rate 30 mL/min; eluent: 100% EtOAc to 4:1 EtOAc–MeOH] to provide **4** (0.68 g, 63%) as a white solid. R_f 0.2 (EtOAc/MeOH 5:1); ^1H NMR (300 MHz, CD_3OD): δ 1.71 (1 H, dd, $J_{3ax,3eq}$ 13.2 Hz, $J_{3ax,4}$ 11.4 Hz, H-3ax), 1.97 (3 H, s, NHCOCH_3), 2.32 (1 H, dd, $J_{3eq,3ax}$ 13.2 Hz, $J_{3eq,4}$ 4.8 Hz, H-3eq), 3.20 (3 H, s, OCH_3), 3.51 (1 H, d, J 9.6 Hz, H-6), 3.59 (1 H, dd, $J_{9A,9B}$ 12.0 Hz, $J_{9A,8}$ 5.7 Hz, H-9A), 3.74-3.88 (7 H, m, H-5, H-7, H-8, H-9B and CO_2CH_3), 3.94-4.01 (1 H, ddd, $J_{4,3ax}$ 11.4, $J_{4,5}$ 10.8 Hz, $J_{4,3eq}$ 4.8 Hz, H-4); ^{13}C NMR (75.5 MHz, CD_3OD): δ 21.9 (NHCO*C*H_3), 39.0 (C-3), 50.8 (O*C*H_3), 51.5 (C-5), 53.4 (CO_2*C*H_3), 63.2 (C-9), 66.3 (C-4), 67.8, 69.6 and 70.4 (C-6, C-7 and C-8), 99.0 (C-2), 170.3 and 174.6 (C-1 and NH*C*OCH_3); LRMS (ESI): *m/z* 360.3 [$(M+Na)^+$ 100%].

Methyl 5-acetamido-3,5-dideoxy-D-*glycero*-β-D-*galacto*-non-2-ulopyranosidonic acid (3).

Compound **3** was prepared from **4** (0.10 g, 0.29 mmol), according to the general procedure for de-esterification (90 mg, 95% yield). HPLC: 0.05% TFA in 0.3:99.7 CH_3CN/H_2O; retention time 5.5-6.7 min; ^1H NMR (600 MHz, D_2O): δ 1.71 (1 H, dd, $J_{3ax,3eq}$ 13.2 Hz, $J_{3ax,4}$ 12.0 Hz, H-3ax), 2.06 (3 H, s, NHCOCH_3), 2.36 (1 H, dd, $J_{3eq,3ax}$ 13.2 Hz, $J_{3eq,4}$ 4.8 Hz, H-3eq), 3.24 (3 H, s, OCH_3), 3.56 (1 H, d, J 9.6 Hz, H-7), 3.65 (1 H, dd, $J_{9A,9B}$ 12.0 Hz, $J_{9A,8}$ 6.0 Hz, H-9A), 3.81-3.94 (4 H, m, H-5, H-6, H-8 and H-9B), 4.01-4.07 (1 H, m, H-4); ^{13}C NMR (150 MHz, D_2O): δ 22.0 (NHCO*C*H_3), 39.5 (C-3), 50.6 (O*C*H_3), 51.8 (C-5), 63.4 (C-9), 66.8 (C-4), 68.2 (C-7), 69.9, 70.2 (C-8, C-6), 100.0 (C-2), 174.1, 174.7 (NH*C*OCH_3, C-1); LRMS (ESI): *m/z* 322.2 [$(M-H)^-$ 100%]. ^1H [6, 7] and ^{13}C [6, 8] NMR data are consistent with those reported.

Methyl (methyl 5-acetamido-4,7,8-tri-*O*-acetyl-8-carboxy-3,5-dideoxy-D-*glycero*-β-D-*galacto*-oct-2-ulopyranosid)onate (5).

To a stirring solution of **4** (5.0 g, 10.8 mmol) and TEMPO (0.02 g, 0.18 mmol) in CH_2Cl_2 (100 mL) was added a solution of saturated aq. $NaHCO_3$ (30 mL) containing KBr (0.15 g, 0.3 mmol) and Bu_4NBr (0.15 g, 0.4 mmol). The biphasic mixture was stirred vigorously at 0 °C, while a solution of aq. NaOCl (10-15%, 35 mL), containing saturated aq. $NaHCO_3$ (18 mL) and saturated NaCl (30 mL), was added over 15 min. After 45 min, a further portion of aq. NaOCl (10-15%, 35 mL) was added. After an additional 15 min, the pH was adjusted to pH 2 using HCl (4 M) and the mixture concentrated under vacuum. The residue was then taken up in pyridine (50 mL) and acetic anhydride (25 mL), and stirred for 16 h at room temperature, concentrated to a yellow syrup, and purified by column chromatography on silica gel to give **5** [9] (3.4 g, 66%) as a white solid. R_f 0.2 (EtOAc/MeOH 3:1); ^{1}H NMR (300 MHz, CD_3OD): δ 1.78 (1 H, dd, $J_{3ax,3eq}$ 12.9 Hz, $J_{3ax,4}$ 11.7 Hz, H-3ax), 1.85 (3 H, s, NHCOC*H*$_3$), 1.95, 2.07 and 2.08 (3 x 3 H, 3 x s, 3 x OCOC*H*$_3$), 2.39 (1 H, dd, $J_{3eq,3ax}$ 12.9 Hz, $J_{3eq,4}$ 5.1 Hz, H-3eq), 3.20 (3 H, s, OC*H*$_3$), 3.78 (3 H, s, CO_2C*H*$_3$), 3.96-4.12 (2 H, m, H-5 and H-6), 5.04 (1 H, d, $J_{8,7}$ 9.0 Hz, H-8), 5.13-5.26 (1 H, m, H-4), 5.41 (1 H, dd, $J_{7,8}$ 9.0 Hz, $J_{7,6}$ 1.5 Hz, H-7); ^{13}C NMR (75.5 MHz, CD_3OD): δ 20.0, 20.1 and 21.6 (3 x OCO*C*H$_3$, NHCO*C*H$_3$), 36.4 (C-3), 48.8 (C-5), 50.9 (O*C*H$_3$), 53.5 (CO_2*C*H$_3$), 68.0 (C-7), 69.0 (C-4), 69.5 (C-6), 71.4 (C-8), 99.0 (C-2), 169.1 (C-1), 172.0, 172.7, 173.1, 173.5 and 174.2 (3 x O*C*OCH$_3$, NH*C*OCH$_3$ and C-9); LRMS (ESI): *m/z* 476 [$(M-H)^-$ 100%]; HRMS (ESI): calcd for $C_{19}H_{26}N_1O_{13}$ $[M-H]^-$ 476.1409, found 476.1423.

Methyl 5-acetamido-8-carboxy-3,5-dideoxy-D-*glycero*-β-D-*galacto*-oct-2-ulopyranosidonic acid (8).

Compound **8** [9] was prepared from **5** (0.1 g, 0.20 mmol), according to the general procedure for de-esterification (64 mg, 90% yield). HPLC: 0.05% TFA in 0.3:99.7 CH_3CN/H_2O; retention time 5.5-7.0 min; ^{1}H NMR (300 MHz, D_2O): δ 1.76 (1 H, dd, $J_{3ax,3eq}$ 12.9 Hz, $J_{3ax,4}$ 11.7 Hz, H-3ax), 2.01 (3 H, s, NHCOC*H*$_3$), 2.36 (1 H, dd, $J_{3eq,3ax}$ 13.2 Hz, $J_{3eq,4}$ 4.8 Hz, H-3eq), 3.22 (3 H, s, OC*H*$_3$), 3.75-3.84 (2 H, m, H-6 and H-7), 3.92 (1 H, app.t, $J_{5,4} \approx J_{5,6}$ 10.2 Hz, H-5), 3.90-4.10 (1 H, m, H-4), 4.35 (1 H, d, $J_{7,8}$ 9.0 Hz, H-8); ^{13}C NMR (75.5 MHz, D_2O): δ 22.0 (NHCO*C*H$_3$), 39.3 (C-3), 50.7 (O*C*H$_3$), 51.5 (C-5), 66.5 (C-4), 69.1 (C-6), 70.0 (C-7) and 70.1 (C-8), 99.7 (C-2), 172.8 (C-1), 174.7 (C-9), 176.5 (NH*C*OCH$_3$); LRMS (ESI): *m/z* 359.6 [$(M+Na)^+$ 100%]; HRMS (ESI): calcd for $C_{12}H_{19}N_1NaO_{10}$ $[M+Na]^+$ 360.0901, found 360.0915.

Methyl [methyl 5-acetamido-4,7,8-tri-*O*-acetyl-8-(*N*-butyl)carboxamido-3,5-dideoxy-D-*glycero*-β-D-*galacto*-oct-2-ulopyranosid]onate (6a).

To a solution of **5** (0.2 g, 0.41 mmol) in CH_2Cl_2/DMF (4:1) (5 mL) were added EDC (0.07 g, 0.45 mmol) and HOBt (0.06 g, 0.46 mmol) under N_2, and the reaction mixture was stirred for 15 min at rt. Butylamine (0.08 mL, 0.82 mmol) and DIPEA (0.28 mL, 1.64 mmol) were then added, and the reaction was stirred at rt. The progress of the reaction was monitored by TLC (hexane/EtOAc, 1:5). After 16 h, the reaction mixture was diluted with EtOAc (30 mL) and subsequently washed with water (20 mL), satd $NaHCO_3$ (20 mL) and satd NaCl (20 mL), dried (Na_2SO_4), filtered, and concentrated under reduced pressure. The crude product was purified by flash chromatography (hexane/EtOAc 1:1 1:5) to afford **6a** as a white solid (135 mg, 60%). R_f 0.30 (hexane/EtOAc 1:5); ^{1}H NMR (300 MHz, $CDCl_3$): δ 0.89 (3 H, t, $J_{d,c}$ 7.3 Hz, H-d), 1.26-1.49 (4 H, m, H-b and H-c), 1.87 (3 H, s, NHCOC*H*$_3$), [H-3ax obscured], 1.99, 2.07 and 2.14 (3 x 3 H, 3 x s, 3 x OCOC*H*$_3$), 2.44 (1 H, dd, $J_{3eq,3ax}$ 12.6 Hz, $J_{3eq,4}$ 4.8 Hz, H-3eq), 3.14-3.35 (5 H, m, OC*H*$_3$ and H-a), 3.80 (3 H, s, CO_2C*H*$_3$), 3.99 (1 H, dd, $J_{6,5}$ 10.5 Hz, $J_{6,7}$ 2.1 Hz, 6-H), 4.15 (1 H, q, $J_{5,4} \approx J_{5,6} \approx J_{5,NH}$ 10.2 Hz, H-5), 5.24 (1 H, ddd, $J_{4,3ax} \approx J_{4,5}$ 10.8 Hz, $J_{4,3eq}$ 4.8 Hz, H-4), 5.32 (1 H, d, $J_{8,7}$ 4.8 Hz, H-8), 5.36-5.41 (2 H, m, N*H*COCH$_3$ and H-7), 6.65 (1H, app. t, *J* 6.0 Hz, N*H*CO); ^{13}C NMR (75.5 MHz, $CDCl_3$): δ 13.7 (C-d), 20.0 (C-c), 20.7, 20.8 and 20.9 (3 x OCO*C*H$_3$), 23.2 (NHCO*C*H$_3$), 31.4, 33.9 and 39.3 (C-a, C-b and C-c), 37.2 (C-3), 49.3 (C-5), 51.2 (O*C*H$_3$), 52.9 (CO_2*C*H$_3$), 68.7 (C-4), 69.0 (C-7), 71.4 (C-6), 72.2 (C-8), 98.7 (C-2), 162.3 (*C*ONH), 165.8 (C-1), 169.5, 170.1, 170.2, and 171.0 (3 x O*C*OCH$_3$ and NH*C*OCH$_3$); LRMS (ESI): *m/z* 555.1 [$(M+Na)^+$ 100%]; HRMS (ESI): calcd for $C_{23}H_{37}N_2O_{12}$ $[M+H]^+$ 533.2341, found 533.2341.

Methyl [methyl 5-acetamido-4,7,8-tri-*O*-acetyl-8-(*N*-cyclohexyl)carboxamido-3,5-dideoxy-D-*glycero*-β-D-*galacto*-oct-2-ulopyranosid]onate (6b).

Compound **6b** was prepared by coupling of **5** (0.4 g, 0.83 mmol) with cyclohexylamine (0.14 mL, 1.25 mmol) in a similar manner to that described for the synthesis of **6a** from **5**. The crude reaction product was purified by flash chromatography (hexane/EtOAc, 1:1 EtOAc) to give **6b** (210 mg, 60%) as a white solid. R_f 0.26 (hexane/EtOAc, 1:5); ^{1}H NMR (300 MHz, $CDCl_3$): δ 0.99-1.31 (6 H, m, H-c and H-c', H-d), 1.55-1.85 (5 H, m, H-b and H-b', H-3ax), 1.85 (3 H, s, NHCOC*H*$_3$), 1.97, 2.06 and 2.10 (3 x 3 H, 3 x s, 3 x OCOC*H*$_3$), 2.41 (1 H, dd, $J_{3eq,3ax}$ 12.6 Hz, $J_{3eq,4}$ 4.8 Hz, H-3eq), 3.15 (3 H, s, OC*H*$_3$), 3.61-3.73 (1 H, m, H-a), 3.78 (3 H, s, CO_2C*H*$_3$), 3.94 (1 H, dd, $J_{6,5}$ 10.8 Hz, $J_{6,7}$ 2.4 Hz, H-6), 4.15 (1 H, app.q, $J_{5,4} \approx J_{5,6} \approx J_{5,NH}$ 10.2 Hz, H-5), 5.16-5.26 (2 H, m, H-4 and H-8), 5.39 (1 H, d, $J_{7,8}$ 5.7 Hz, H-7), 5.96 (1 H, br.m, N*H*COCH$_3$), 6.27 (1 H, q, *J* 8.1 Hz, CON*H*); ^{13}C NMR (75.5 MHz,

CDCl$_3$): δ 20.7, 20.8 and 20.9 (3 x OCOCH_3), 23.1 (NHCOCH_3), 24.8 (C-d), 25.4 (C-c and C-c'), 32.8 (C-b and C-b'), 37.2 (C-3), 48.5 (C-a), 49.2 (C-5), 51.1 (OCH_3), 52.8 (CO$_2$CH_3), 68.5 (C-7), 68.9 (C-4), 71.1 (C-6), 71.5 (C-8), 98.7 (C-2), 165.4 (C-1), 169.5 (*C*ONH), 169.8, 170.3, 170.4 and 171.0 (3 x O*C*OCH$_3$ and NH*C*OCH$_3$); LRMS (ESI): *m/z* 581.0 [(M+Na)$^+$ 100%]; HRMS (ESI): calcd for $C_{25}H_{38}N_2NaO_{12}$ [M+Na]$^+$ 581.2316, found 581.2292.

Methyl [methyl 5-acetamido-4,7,8-tri-*O*-acetyl-8-(*N*-benzyl)carboxamido-3,5-dideoxy-D-*glycero*-β-D-*galacto*-oct-2-ulopyranosid]onate (6c).

Compound **6c** was prepared by coupling of **5** (0.2 g, 0.41 mmol) with benzylamine (0.06 mL, 0.61 mmol) in a similar manner to that described for the synthesis of **6a** from **5**. The crude reaction product was purified by flash chromatography (hexane/EtOAc, 1:1 EtOAc) to give **6c** (133 mg, 56%) as a white solid. R_f 0.65 (EtOAc/MeOH 5:1); ^{1}H NMR (300 MHz, CDCl$_3$): δ [H-3ax obscured],1.85 (3 H, s, NHCOCH_3), 1.86, 1.99 and 2.14 (3 x 3 H, 3 x s, 3 x OCOCH_3), 2.41 (1 H, dd, $J_{3eq,3ax}$ 12.9 Hz, $J_{3eq,4}$ 4.8 Hz, H-3eq), 3.20 (3 H, s, OCH_3), 3.73 (3 H, s, CO$_2$CH_3), 3.99 (1 H, dd, $J_{6,5}$ 10.5 Hz, $J_{6,7}$ 2.7 Hz, H-6), 4.15 (1 H, app.q, $J_{5,4}$ ≈ $J_{5,6}$ ≈ $J_{5,NH}$ 10.2 Hz, H-5), 4.31 (1 H, dd, $J_{a,b}$ 14.7 Hz, $J_{a,NH}$ 5.1 Hz, H-a), 4.56 (1 H, dd, $J_{b,a}$ 14.7 Hz, $J_{b,NH}$ 6.9 Hz, H-b), 5.24 (1 H, ddd, $J_{4,3ax}$ ≈ $J_{4,5}$ 11.1 Hz, $J_{4,3eq}$ 4.8 Hz, H-4), 5.34 (1 H, d, $J_{8,7}$ 4.5 Hz, H-8), 5.39 (1 H, m, H-7), 7.14 (1 H, app.t, $J_{NH,a}$ ≈ $J_{NH,b}$ 6.3 Hz, CON*H*), 7.23-7.30 (5 H, m, Ar*H*); ^{13}C NMR (75.5 MHz, CDCl$_3$): δ 20.4, 20.8 and 20.9 (3 x OCOCH_3), 23.1 (NHCOCH_3), 37.2 (C-3), 43.5 (C-a), 49.1 (C-5), 51.2 (OCH_3), 52.9 (CO$_2$CH_3), 68.7 (C-7), 68.8 (C-4), 71.5 (C-6), 72.2 (C-8), 98.8 (C-2), 127.9, 128.0, 128.5, 128.7 and 128.8 (5 x Ar*C*), 138.7 (*ipso*-Ar), 165.9 (C-1), 167.5 (*C*ONH), 169.6, 170.2, 170.5 and 171.0 (3 x O*C*OCH$_3$ and NH*C*OCH$_3$); LRMS (ESI): *m/z* 588.9 [(M+Na)$^+$ 100%]; HRMS (ESI): calcd for $C_{26}H_{34}N_2NaO_{12}$ [M+Na]$^+$ 589.2003, found 589.2002.

Methyl {methyl 5-acetamido-4,7,8-tri-*O*-acetyl-3,5-dideoxy-8-[*N*-(3-pyridyl-methyl)]carboxamido-D-*glycero*-β-D-*galacto*-oct-2-ulopyranosid}onate (6d).

Compound **6d** was prepared by coupling of **5** (0.4 g, 0.83 mmol) with 3-(aminomethyl)pyridine (0.12 mL, 1.25 mmol) in a similar manner to that described for the synthesis of **6a** from **5**. The crude reaction product was purified by flash chromatography (hexane/EtOAc, 1:1 EtOAc) to give **6d** (190 mg, 55%) as a white solid. R_f 0.48 (EtOAc/MeOH 5:1); ^{1}H NMR (300 MHz, CDCl$_3$): δ ([H-3ax not assigned], 1.82 (3H, s, NHCOCH_3), 1.85, 1.99 and 2.14 (3 x 3 H, 3 x s, 3 x OCOCH_3), 2.47 (1 H, dd, $J_{3eq,3ax}$ 12.9 Hz, $J_{3eq,4}$ 4.8 Hz, H-3eq), 3.22 (3 H, s, OCH_3), 3.77 (3 H, s, CO$_2$CH_3), 4.02 (1 H, dd, $J_{6,5}$ 10.2 Hz, $J_{6,7}$ 2.7 Hz, H-6), 4.13 (1 H, q, $J_{5,4}$ ≈ $J_{5,6}$ ≈ $J_{5,NH}$ 10.2 Hz, H-5), 4.33 (1 H, dd, $J_{a,b}$ 15.3 Hz, $J_{a,NH}$ 5.7 Hz, H-a), 4.59 (1 H, dd, $J_{b,a}$ 15.3 Hz, $J_{b,NH}$ 6.6 Hz, H-b), 5.26 (1 H, ddd, $J_{4,3ax}$ 11.1 Hz, $J_{4,5}$ 10.2 Hz, $J_{4,3eq}$ 4.8 Hz, H-4), 5.33-5.40 (3 H, m, H-7, H-8 and N*H*COCH$_3$), 7.61-7.70 (2 H, m, Ar*H*, CON*H*), 8.47-8.57 (3 H, m, 3 x Ar*H*); ^{13}C NMR (75.5 MHz, CDCl$_3$): δ 20.4, 20.8 and 20.8 (3 x OCOCH_3), 23.2 (NHCOCH_3), 37.1 (C-3), 41.0 (C-a), 49.2 (C-5), 51.5 (OCH_3), 53.1 (CO$_2$CH_3), 68.4 (C-7), 69.3 (C-4), 72.4 (C-6), 73.2 (C-8), 98.9 (C-2), 123.4 (Ar*C*), 134.2 (ArC_q), 135.9, 148.6 and 149.5 (3 x Ar*C*), 165.5 (C-1), 170.0, 170.2, 170.6 and 171.0 (3 x O*C*OCH$_3$ and NH*C*OCH$_3$); LRMS (ESI): *m/z* 590.1 [(M+Na)$^+$ 100%]; HRMS (ESI): calcd for $C_{25}H_{32}N_3O_{12}$ [M-H]$^-$ 568.2137, found 568.2134.

Methyl [methyl 5-acetamido-4,7,8-tri-*O*-acetyl-3,5-dideoxy-8-(glycine methyl ester)carboxamido-D-*glycero*-β-D-*galacto*-oct-2-ulopyranosid]onate (7a).

Compound **7a** was prepared by coupling of **5** (0.3 g, 0.62 mmol) with glycine methyl ester (0.11 g, 0.93 mmol) in a similar manner to that described for the synthesis of **6a** from **5**. The crude reaction product was purified by flash chromatography (hexane/EtOAc, 1:1 EtOAc) to give **7a** (190 mg, 55%) as a white solid. R_f 0.5 (EtOAc/MeOH 5:1); ^{1}H NMR (300 MHz, CDCl$_3$): δ [H-3ax not assigned], 1.85 (3 H, s, NHCOCH_3), 1.98, 2.09 and 2.12 (3 x 3 H, 3 x s, 3 x OCOCH_3), 2.42 (1 H, dd, $J_{3eq,3ax}$ 12.9 Hz, $J_{3eq,4}$ 4.8 Hz, H-3eq), 3.20 (3 H, s, OCH_3), 3.71 and 3.78 (2 x 3 H, 2 x s, 2 x CO$_2$CH_3), 3.95-3.98 (3 H, m, H-a and H-6), 4.16 (1 H, app.q, $J_{5,4}$ ≈ $J_{5,6}$ ≈ $J_{5,NH}$ 10.2 Hz, H-5), 5.22 (1 H, ddd, $J_{4,3ax}$ ≈ $J_{4,5}$ 11.1 Hz, $J_{4,3eq}$ 4.8 Hz, H-4), 5.34-5.37 (2 H, m, H-7 and H-8), 5.64 (1 H, br.s, N*H*COCH$_3$), 7.13 (1 H, br.s, CON*H*); ^{13}C NMR (75.5 MHz, CDCl$_3$): δ 20.7, 20.7 and 20.9 (3 x OCOCH_3), 23.1 (NHCOCH_3), 37.2 (C-3), 41.2 (C-a), 49.2 (C-5), 51.2 (OCH_3), 52.3 and 52.9 (2 x *C*O$_2$*C*H$_3$), 68.6 (C-4), 68.7 (C-6), 71.1 (C-7), 72.0 (C-8), 98.8 (C-2), 166.6 (C-1), 167.5 (*C*O$_2$CH$_3$), 169.9 (*C*ONH), 169.9, 170.0, 170.4 and 171.0 (NH*C*OCH$_3$, 3 x O*C*OCH$_3$); LRMS (ESI): *m/z* 571.0 [(M+Na)$^+$ 100%]; HRMS (ESI): calcd for $C_{22}H_{32}N_2NaO_{14}$ [M+Na]$^+$ 571.1745, found 571.1736.

Methyl [methyl 5-acetamido-4,7,8-tri-*O*-acetyl-8-(L-alanine methyl ester)carboxamido-3,5-dideoxy-D-*glycero*-β-D-*galacto*-oct-2-ulopyranosid]onate (7b).

Compound **7b** was prepared by coupling of **5** (0.23 g, 0.48 mmol) with L-alanine methyl ester (0.10 g, 0.72 mmol) in a similar manner to that described for the synthesis of **6a** from **5**. The crude reaction product was purified by flash chromatography (hexane/EtOAc, 1:1 EtOAc) to give **7b** (135 mg, 60%) as a white solid. R_f 0.58 (EtOAc/MeOH 5:1); ^{1}H NMR (300 MHz, CDCl$_3$): δ 1.40 (1 H, d, $J_{b,a}$ 7.2 Hz, H-b), [H-3ax not assigned], 1.87 (3 H, s, NHCOCH_3), 2.00, 2.09 and 2.13 (3 x 3 H, 3 x s, 3 x OCOCH_3),

2.42 (1 H, dd, $J_{3eq,3ax}$ 12.6 Hz, $J_{3eq,4}$ 4.8 Hz, H-3eq), 3.21 (3 H, s, OCH_3), 3.72 and 3.80 (2 x 3 H, 2 x s, 2 x CO_2CH_3), 3.95 (1 H, dd, $J_{6,7}$ 2.4 Hz, $J_{6,5}$ 10.2 Hz, H-6), 4.17 (1 H, app.q, $J_{5,4} \approx J_{5,6} \approx J_{5,NH}$ 10.2 Hz, H-5), 4.49-4.58 (1 H, m, H-a), 5.20-5.38 (4 H, m, H-4, H-7, H-8 and N*H*$COCH_3$), 6.73 (1H, d, *J* 7.2 Hz, CON*H*); ^{13}C NMR (75.5 MHz, $CDCl_3$): δ 18.0 (C-b), 20.6, 20.7 and 20.9 (3 x $OCOCH_3$), 23.0 ($NHCOCH_3$), 37.2 (C-3), 48.1 (C-a), 51.0 (C-5), 52.4 (OCH_3), 52.5 and 52.8 (2 x CO_2CH_3), 68.2 (C-4), 68.9 (C-6), 70.6 (C-7), 71.3 (C-8), 98.7 (C-2), 166.2 (C-1), 167.4 (CO_2CH_3), 169.6 (*C*ONH), 170.1, 170.4, 171.0 and 173.0 ($NHCOCH_3$, 3 x $OCOCH_3$); LRMS (ESI): *m/z* 584.8 [$(M+Na)^+$ 100%]; HRMS (ESI): calcd for $C_{23}H_{34}N_2NaO_{14}$ $[M+Na]^+$ 585.1902, found 585.1874.

Methyl [methyl 5-acetamido-4,7,8-tri-*O*-acetyl-3,5-dideoxy-8-(L-leucine methyl ester)carboxamido-D-*glycero*-β-D-*galacto*-oct-2-ulopyranosid]onate (7c).

Compound **7c** was prepared by coupling of **5** (0.3 g, 0.62 mmol) with L-leucine methyl ester (0.16 g, 0.93 mmol) in a similar manner to that described for the synthesis of **6a** from **5**. The crude reaction product was purified by flash chromatography (hexane/EtOAc, 1:1 1:5) to give **7c** (230 mg, 60%) as a white solid. R_f 0.29 (EtOAc); ^{1}H NMR (300 MHz, $CDCl_3$): δ 0.89-0.92 (6 H, m, H-d and H-d'), 1.57-1.66 (3 H, m, H-c and H-b), [H3-ax obscured], 1.87 (3 H, s, $NHCOCH_3$), 2.00, 2.09 and 2.12 (3 x 3 H, 3 x s, 3 x $OCOCH_3$), 2.42 (1 H, dd, $J_{3eq,3ax}$ 12.9 Hz, $J_{3eq,4}$ 4.8 Hz, H-3eq), 3.22 (3 H, s, OCH_3), 3.70 and 3.79 (2 x 3 H, 2 x s, 2 x CO_2CH_3), 3.94 (1 H, dd, $J_{6,7}$ 2.7 Hz, $J_{6,5}$ 10.5 Hz, H-6), 4.15 (1 H, app.q, $J_{5,4} \approx J_{5,6} \approx J_{5,NH}$ 10.5 Hz, H-5), 4.54-4.61 (1 H, m, H-a), 5.20-5.25 (1 H, m, H-4), 5.27 (1 H, d, $J_{8,7}$ 6.3 Hz, H-8), 5.37 (1 H, dd, $J_{7,8}$ 6.3 Hz, $J_{7,6}$ 2.7 Hz, H-7), 5.45 (1 H, d, $J_{NH,5}$ 9.9 Hz, N*H*$COCH_3$), 6.54 (1 H, d, *J* 8.4 Hz, CON*H*); ^{13}C NMR (75.5 MHz, $CDCl_3$): δ 20.6, 20.7 and 20.9 (3 x $OCOCH_3$), 22.7, 22.9 (C-d and C-d'), 23.0 ($NHCOCH_3$), 24.5 (C-c), 37.2 (C-3), 41.2 (C-b), 49.0 (C-5), 50.8 (C-a), 51.0 (OCH_3), 52.2 and 52.8 (2 x CO_2CH_3), 68.3 (C-4), 69.0 (C-6), 70.5 (C-7), 71.3 (C-8), 98.6 (C-2), 166.4 and 167.4 (2 x CO_2CH_3), 169.6 (*C*ONH), 170.0, 170.5, 170.9 and 173.0 ($NHCOCH_3$, 3 x $OCOCH_3$); LRMS (ESI): *m/z* 627.0 [$(M+Na)^+$ 100%]; HRMS (ESI): calcd for $C_{26}H_{40}N_2NaO_{14}$ $[M+Na]^+$ 627.2371, found 627.2380.

Methyl [methyl 5-acetamido-4,7,8-tri-*O*-acetyl-3,5,-dideoxy-8-(D/L-serine methyl ester)carboxamido-D-*glycero*-β-D-*galacto*-oct-2-ulopyranosid]onate (**7d**) .

Compound **7d** was prepared by coupling of **5** (0.3 g, 0.62 mmol) with D/L-serine methyl ester hydrochloride (0.14 g, 0.93 mmol) in a similar manner to that described for the synthesis of **6a** from **5**. The crude reaction product was purified by flash chromatography (hexane/EtOAc, 1:1 EtOAc) to give **7d** (225 mg, 62%) as a white solid. R_f 0.42 (EtOAc/MeOH 5:1); ^{1}H NMR (300 MHz, $CDCl_3$): δ [H3-ax not assigned], 1.86 (3 H, s, $NHCOCH_3$), 1.99, 2.12 and 2.13 (3 x 3 H, 3 x s, 3 x $OCOCH_3$), 2.40 (1 H, dd, $J_{3eq,3ax}$ 12.9 Hz, $J_{3eq,4}$ 4.8 Hz, H-3eq), 3.17 (3 H, s, OCH_3), 3.77 and 3.79 (2 x 3 H, 2 x s, 2 x CO_2CH_3), 3.85-3.98 (3 H, m, H-6 and CH_2OH), 4.16 (1 H, q, $J_{5,4} \approx J_{5,6} \approx J_{5,NH}$ 10.8 Hz, H-5), 4.44-4.56 (1 H, m, H-a), 5.24-5.40 (3 H, m, H-4, H-7 and H-8), 5.56 (1 H, d, $J_{NH,5}$ 10.2 Hz, N*H*$COCH_3$) 7.09 (1 H, app.t, *J* 6.9 Hz, CON*H*); ^{13}C NMR (75.5 MHz, $CDCl_3$): δ 20.6, 20.8 and 20.9 (3 x $OCOCH_3$), 23.1 ($NHCOCH_3$), 37.1 (C-3), 49.3 (C-5), 51.0 (OCH_3), 52.8 and 52.9 (2 x CO_2CH_3), 55.1 (C-a), 61.6 (C-b), 68.3 (C-4), 68.7 (C-6), 70.3 (C-7), 71.1 (C-8), 98.8 (C-2), 166.8 (*C*ONH), 170.4, 170.8, 171.1 and 171.3 (3 x $OCOCH_3$, $NHCOCH_3$), [C-1 and CO_2CH_3-serine not assigned]; LRMS (ESI): *m/z* 600.9 [$(M+Na)^+$ 100%]; HRMS (ESI): calcd for $C_{23}H_{34}N_2NaO_{15}$ $[M+Na]^+$ 601.1851, found 601.1831.

Methyl [methyl 5-acetamido-4,7,8-tri-*O*-acetyl-3,5-dideoxy-8-(L-phenylalanine methyl ester)carboxamido-D-*glycero*-β-D-*galacto*-oct-2-ulopyranosid]onate (**7e**).

Compound **7e** was prepared by coupling of **5** (0.3 g, 0.62 mmol) with L-phenylalanine methyl ester hydrochloride (0.20 g, 0.93 mmol) in a similar manner to that described for the synthesis of **6a** from **5**. The crude reaction product was purified by flash chromatography (hexane/EtOAc, 1:1 EtOAc) to give **7e** (220 mg, 55%) as a white solid. R_f 0.45 (EtOAc); ^{1}H NMR (300 MHz, $CDCl_3$): δ 1.84 (3 H, s, $NHCOCH_3$), [H3-ax obscured], 1.99, 2.03 and 2.11 (3 x 3 H, 3 x s, 3 x $OCOCH_3$), 2.39 (1 H, dd, $J_{3eq,3ax}$ 12.9 Hz, $J_{3eq,4}$ 4.8 Hz, H-3eq), 3.10 (1 H, d, *J* 5.4 Hz, H-b), 3.18 (3 H, s, OCH_3), 3.68 and 3.78 (2 x 3 H, 2 x s, 2 x CO_2CH_3), 3.91 (1 H, d, $J_{6,5}$ 10.2 Hz, $J_{6,7}$ 2.4 Hz, H-6), 4.14 (1 H, q, $J_{5,4} \approx J_{5,6} \approx J_{5,NH}$ 10.2 Hz, H-5), 4.78-4.84 (1 H, m, H-a), 5.14 (1 H, d, *J* 7.5 Hz, H-8), 5.18-5.36 (3 H, m, H-4, N*H*$COCH_3$ and H-7), 6.56 (1 H, d, *J* 7.5 Hz, CON*H*), 7.14-7.30 (5 H, m, 5 x Ar*H*); ^{13}C NMR (75.5 MHz, $CDCl_3$): δ 20.6, 20.7 and 20.9 (3 x $OCOCH_3$), 23.1 ($NHCOCH_3$), 37.3 (C-b), 37.6 (C-3), 49.1 (C-5), 51.0 (OCH_3), 52.3 and 52.8 (2 x CO_2CH_3), 53.3 (*C*-a), 68.0 (C-4), 68.8 (C-6), 70.4 (C-7), 71.0 (C-8), 98.7 (C-2), 127.1, 128.5 and 129.5 (5 x Ar*C*), 135.6 (*ipso*-Ar*C*), 166.5 (C-1), 167.4, 169.5, 170.1, 170.4, 171.0 and 171.4 (*C*ONH, 2 x CO_2CH_3, 3 x $OCOCH_3$ and $NHCOCH_3$); LRMS (ESI): *m/z* 660.9 [$(M+Na)^+$ 100%]; HRMS (ESI): calcd for $C_{29}H_{38}N_2NaO_{14}$ $[M+Na]^+$ 661.2215, found 661.2189.

Methyl [methyl 5-acetamido-4,7,8-tri-*O*-acetyl-3,5-dideoxy-8-(L-tyrosine methyl ester)carboxamido-D-*glycero*-β-D-*galacto*-oct-2-ulopyranosid]onate (**7f**).

Compound **7f** was prepared by coupling of **5** (0.2 g, 0.41 mmol) with L-tyrosine methyl ester hydrochloride (0.14 g, 0.62 mmol) in a similar manner to that described for the synthesis of **6a** from **5**. The crude reaction product was purified by flash chromatography (hexane/EtOAc, 1:1   EtOAc) to give **7f** (160 mg, 60%) as a white solid. R_f 0.57 (EtOAc); ^{1}H NMR (300 MHz, CD_3OD): δ 1.80 (1 H, m, H-3ax),1.83 (3 H, s, NHCOCH_3), 1.96, 2.01 and 2.09 (3 x 3 H, 3 x s, 3 x OCOCH_3), 2.43 (1 H, dd, $J_{3eq,3ax}$ 12.9 Hz, $J_{3eq,4}$ 4.8 Hz, H-3eq), 2.94-2.97 (2 H, m, H-b), 3.14 (3 H, s, OCH_3), 3.64 and 3.81 (2 x 3 H, 2 x s, 2 x CO_2CH_3), 4.00-4.02 (2 H, m, H-5 and H-6), 4.62 (1 H, app.t, *J* 6.0 Hz, H-a), 5.12-5.17 (1 H, m, H-4), 5.20 (1 H, d, $J_{8,7}$ 9.6 Hz, H-8), 5.30 (1 H, d, *J* 9.3 Hz, H-7), 6.68 (2 H, d, *J* 8.4 Hz, Ar*H*), 7.00 (2 H, d, *J* 8.4 Hz, Ar*H*); ^{13}C NMR (75.5 MHz, CD_3OD): δ 19.6, 19.9 and 21.7 (3 x OCOCH_3, NHCOCH_3), 36.6 (C-b), 37.0 (C-3), 48.6 (C-5), 50.4 (OCH_3), 51.5 and 52.4 (2 x CO_2CH_3), 54.1(C-a), 67.4 (C-4), 69.1 (C-6), 69.6 (C-7), 69.7 (C-8), 98.8 (C-2), 114.9, 126.6, 130.3 and 155.8 (6 x Ar*C*), 168.0 (C-1), 168.1, 169.6, 170.0, 170.7, 171.2 and 171.9 (*C*ONH, 2 x CO_2CH_3, 3 x O*C*OCH_3, NH*C*OCH_3); LRMS (ESI): *m/z* 677.0 [$(M+Na)^+$ 100%]; HRMS (ESI): calcd for $C_{29}H_{38}N_2NaO_{15}$ $[M+Na]^+$ 677.2164, found 677.2152.

Methyl [methyl 5-acetamido-4,7,8-tri-*O*-acetyl-3,5-dideoxy-8-(L-histidine methyl ester)carboxamido-D-*glycero*-β-D-*galacto*-oct-2-ulopyranosid]onate (**7g**).

Compound **7g** was prepared by coupling of **5** (0.3 g, 0.62 mmol) with L-histidine methyl ester hydrochloride (0.22 g, 0.93 mmol) in a similar manner to that described for the synthesis of **6a** from **5**. The crude reaction product was purified by flash chromatography (hexane/acetone, 4:1   1:4) to give **7g** (160 mg, 40%) as a white solid. A small amount of unreacted histidine methyl ester co-eluted with the product. R_f 0.70 (hexane/acetone 1:3); ^{1}H NMR (300 MHz, $CDCl_3$): δ 1.83 (4 H, m, NHCOCH_3 and H-3ax), 1.98, 2.04 and 2.11 (3 x 3 H, 3 x s, 3 x OCOCH_3), 2.40 (1 H, dd, $J_{3eq,3ax}$ 12.9 Hz, $J_{3eq,4}$ 4.8 Hz, H-3eq), 3.13-3.16 (5 H, m, H-b and OCH_3), 3.69 and 3.77 (2 x 3 H, 2 x s, 2 x CO_2CH_3), 3.97 (1 H, d, $J_{6,5}$ 10.5 Hz, $J_{6,7}$ 1.8 Hz, H-6), 4.13 (1 H, q, $J_{5,4} \approx J_{5,6} \approx J_{5,NH}$ 10.5 Hz, H-5), 4.66-4.71 (1 H, m, H-a), 5.18-5.23 (1 H, m, H-4), 5.26 (1 H, d, $J_{8,7}$ 7.2 Hz, H-8), 5.40 (1 H, dd, $J_{7,8}$ 7.2 Hz, $J_{7,6}$ 1.8 Hz, H-7), 6.30 (1 H, d, $J_{NH,5}$ 9.6 Hz, N*H*COCH$_3$), 7.61-7.67 (2 H, m, 2 x Ar*H*); ^{13}C NMR (75.5 MHz, $CDCl_3$): δ 20.6, 20.7 and 20.9 (3 x OCOCH_3), 22.9 (NHCOCH_3), 27.8 (C-b), 37.6 (C-3), 49.2 (C-5), 51.0 (OCH_3), 52.3 and 52.8 (2 x CO_2CH_3), 52.9 (*C*-a), 68.2 (C-4), 68.9 (C-6), 70.1 (C-7), 71.4 (C-8), 98.8 (C-2), 118.4, 130.4 and 135.2 (3 x Ar*C*), 167.1 (C-1), 167.5, 169.8, 170.5, 170.7, 170.9 and 171.1 (*C*ONH, 2 x CO_2CH_3, 3 x O*C*OCH_3, NH*C*OCH_3),; LRMS (ESI): *m/z* 650.8 [$(M+Na)^+$ 100%]; HRMS (ESI): calcd for $C_{26}H_{36}N_4NaO_{14}$ $[M+Na]^+$ 651.2120, found 651.2105.

Methyl [methyl 5-acetamido-4,7,8-tri-*O*-acetyl-3,5-dideoxy-8-(L-tryptophan methyl ester)carboxamido-D-*glycero*-β-D-*galacto*-oct-2-ulopyranosid]onate (7h).

Compound **7h** was prepared by coupling of **5** (0.3 g, 0.62 mmol) with L-tryptophan methyl ester hydrochloride (0.23 g, 0.93 mmol) in a similar manner to that described for the synthesis of **6a** from **5**. The crude reaction product was purified by flash chromatography (hexane/EtOAc, 1:1   1:3) to give **7h** (180 mg, 45%) as a white solid. Some unreacted tryptophan methyl ester co-eluted with the product. R_f 0.23 (hexane/EtOAc 1:3); ^{1}H NMR (300 MHz, $CDCl_3$): δ 1.73 (3 H, s, NHCOCH_3), 1.86 (1 H, app.t, $J_{3ax,3eq} \approx J_{3ax,4}$ 12.9 Hz, H-3ax), 1.99, 2.00 and 2.09 (3 x 3 H, 3 x s, 3 x OCOCH_3), 2.39 (1 H, dd, $J_{3eq,3ax}$ 12.9 Hz, $J_{3eq,4}$ 4.8 Hz, H-3eq), 3.18 (3 H, s, OCH_3), 3.20-3.30 (2 H, m, H-b), 3.68 and 3.78 (2 x 3 H, 2 x s, 2 x CO_2CH_3), 3.91 (1 H, d, $J_{6,5}$ 10.2 Hz, $J_{6,7}$ 2.4 Hz, H-6), 4.17 (1 H, q, $J_{5,4} \approx J_{5,6} \approx J_{5,NH}$ 10.2 Hz, H-5), 4.82-4.88 (1 H, m, H-a), 5.13-5.36 (4 H, m, H-4, H-7, H-8 and N*H*COCH$_3$), 6.62 (1 H, d, *J* 7.5 Hz, CON*H*), 7.14-7.30 (3 H, m, 3 x Ar*H*), 7.35 (1 H, Ar*H*), 7.55 (1 H Ar*H*); ^{13}C NMR (75.5 MHz, $CDCl_3$): δ 20.6 and 20.9 (3 x OCOCH_3), 23.0 (NHCOCH_3), 27.5 (C-b), 37.3 (C-3), 49.3 (C-5), 51.0 (OCH_3), 52.0 and 52.4 (2 x CO_2CH_3), 52.7 (C-a), 68.2 (C-4), 68.7 (C-6), 70.4 (C-7), 71.4 (C-8), 98.8 (C-2), 109.4 (ArC_q), 111.2, 118.5, 119.5, 122.1, and 123.6 (5 x Ar*C*), 127.3 and 136.0 (2 x ArC_q), 166.4 (C-1), 167.3, 169.6, 170.2, 170.3, 171.0 and 171.8 (*C*ONH, 2 x CO_2CH_3, 3 x O*C*OCH_3, NH*C*OCH_3); LRMS (ESI): *m/z* 699.8 [$(M+Na)^+$ 100%]; HRMS (ESI): calcd for $C_{31}H_{39}N_3NaO_{14}$ $[M+Na]^+$ 700.2324, found 700.2293.

Methyl 5-acetamido-8-(*N*-butyl)carboxamido-3,5-dideoxy-D-*glycero*-β-D-*galacto*-oct-2-ulopyranosidonic acid (9a).

Prepared from **6a** (0.1 g, 0.18 mmol), according to the general procedure for deprotection (60 mg, 82% yield). HPLC: 0.05% TFA in 14:86 CH_3CN/H_2O; retention time 7.2-11.5 min; ^{1}H NMR (300 MHz, D_2O): δ 0.77 (3 H, t, $J_{d,c}$ 7.5 Hz, H-d), 1.14-1.26 (2 H, m, H-c), 1.33-1.43 (2 H, m, H-b), 1.66 (1 H, dd, $J_{3ax,3eq}$ 13.2 Hz, $J_{3ax,4}$ 11.4 Hz, H-3ax), 1.93 (3 H, s, NHCOCH_3), 2.26 (1 H, dd, $J_{3eq,3ax}$ 12.9 Hz, $J_{3eq,4}$ 5.1 Hz, H-3eq), 3.05-3.17 (5 H, m, H-a and OCH_3), 3.64 (1 H, dd, $J_{7,8}$ 9.0 Hz, $J_{7,6}$ 0.9 Hz, H-7), 3.70 (1 H, d, *J* 10.5 Hz, H-6), 3.79 (1 H, q, $J_{5,4} \approx J_{5,6}$ 10.5 Hz, H-5), 5.24 (1 H, ddd, $J_{4,3ax}$ 11.4 Hz, $J_{4,5}$ 10.5 Hz, $J_{4,3eq}$ 5.1 Hz, H-4), 4.11 (1 H, d, $J_{8,7}$ 9.0 Hz, H-8); ^{13}C NMR (75.5 MHz, D_2O): δ 12.8 (C-d), 19.2 (C-c), 22.0 (NHCOCH_3), 30.3 (C-b), 38.9 (C-3),

39.1 (C-a), 50.8 (C-5), 51.6 (O*C*H_3), 66.3 (C-4), 68.9 (C-6), 70.3 (C-7), 70.8 (C-8), 99.2 (C-2), 172.0 (C-1), 174.2 (*C*ONH), 174.8 (NH*C*OCH_3); LRMS (ESI): *m/z* 391.0 [(M-H)$^-$ 100%]; HRMS (ESI): calcd for $C_{16}H_{27}N_2O_9$ [M-H]$^-$ 391.1722, found 391.1704.

Methyl 5-acetamido-8-(*N*-cyclohexyl)carboxamido-3,5-dideoxy-D-*glycero*-β-D-*galacto*-oct-2-ulopyranosidonic acid (9b).
Prepared from **6b** (0.15 g, 0.27 mmol), according to the general procedure for deprotection (90 mg, 82% yield). HPLC: 0.05% TFA in 18:82 CH_3CN/H_2O; retention time 10.5-12.0 min; ^{1}H NMR (300 MHz, D_2O): δ 1.00-1.30 (5 H, m, 5 x H-cyclohexyl), 1.46-1.72 (6 H, m, 5 x H-cyclohexyl and H-3ax), 1.94 (3 H, s, NHCOC*H*$_3$), 2.27 (1 H, dd, $J_{3eq,3ax}$ 13.2 Hz, $J_{3eq,4}$ 4.8 Hz, H-3eq), 3.15 (3 H, s, OC*H*$_3$), 3.49-3.56 (1 H, m, H-a), 3.62-3.71 (2 H, m, H-7 and H-6), 3.79 (1 H, q, $J_{5,4} \approx J_{5,6}$ 10.5 Hz, H-5), 3.94 (1 H, ddd, $J_{4,3ax}$ 13.2 Hz, $J_{4,5}$ 10.5 Hz, $J_{4,3eq}$ 4.8 Hz, H-4), 4.10 (1 H, d, $J_{8,7}$ 9.0 Hz, H-8); ^{13}C NMR (75.5 MHz, D_2O): δ 22.0 (NHCO*C*H_3), 24.3 (C-d), 24.8 (C-c), 31.8 (C-b), 39.1 (C-3), 48.7 (C-a), 50.8 (O*C*H_3), 51.6 (C-5), 66.4 (C-4), 68.9 (C-6), 70.2 (C-7), 70.8 (C-8), 99.3 (C-2), 172.1 (C-1), 173.1 (*C*ONH), 174.8 (NH*C*OCH_3); LRMS (ESI): *m/z* 417 [(M-H)$^-$ 100%]; HRMS (ESI): calcd for $C_{18}H_{29}N_2O_9$ [M-H]$^-$ 417.1878, found 417.1857.

Methyl 5-acetamido-8-(*N*-benzyl)carboxamido-3,5-dideoxy-D-*glycero*-β-D-*galacto*-oct-2-ulopyranosidonic acid (9c).
Prepared from **6c** (0.05 g, 0.08 mmol), according to the general procedure for deprotection (22 mg, 80% yield). HPLC: 0.05% TFA in 12:88 CH_3CN/H_2O; retention time 17.5-18.5 min; ^{1}H NMR (300 MHz, CD_3OD): δ 1.60 (1 H, dd, $J_{3ax,3eq}$ 13.2 Hz, $J_{3ax,4}$ 11.4 Hz, H-3ax), 2.02 (3 H, s, NHCOC*H*$_3$), 2.33 (1 H, dd, $J_{3eq,3ax}$ 13.2 Hz, $J_{3eq,4}$ 4.8 Hz, H-3eq), 3.15 (3 H, s, OC*H*$_3$), 3.72 - 3.80 (2 H, m, H-7 and H-6), 3.85 (1 H, app.t, $J_{5,4} \approx J_{5,6}$ 10.2 Hz, H-5), 3.95 (1 H, m, H-4), 4.26 (1 H, d, $J_{8,7}$ 9.0 Hz, H-8), 4.37-4.48 (2 H, m, H-a), 7.23-7.33 (5 H, m, 5 x Ar*H*); ^{13}C NMR (75.5 MHz, D_2O): δ 22.0 (NHCO*C*H_3), 39.6 (C-3), 42.6 (C-a), 50.3 (O*C*H_3), 51.7 (C-5), 66.8 (C-4), 69.2 (C-6), 69.8 (C-7), 71.1 (C-8), 100.4 (C-2), 127.0, 127.3 and 128.6 (5 x Ar*C*), 137.6 (*ipso*-Ar*C*), 174.6 (C-1), 174.7 (*C*ONH), 175.1 (NH*C*OCH_3); LRMS: *m/z* 424.8 [(M-H)$^-$ 100%]; HRMS (ESI): calcd for $C_{19}H_{25}N_2O_9$ [M-H]$^-$ 425.1565, found 425.1575.

Methyl 5-acetamido-3,5-dideoxy-8-[*N*-(3-pyridyl-methyl)]carboxamido-D-*glycero*-β-D-*galacto*-oct-2-ulopyranosidonic acid (9d).
Prepared from **6d** (0.15 g, 0.26 mmol), according to the general procedure for deprotection (96 mg, 85% yield). HPLC: 0.05% TFA in 1:99 CH_3CN/H_2O; retention time 7.1-8.1 min; ^{1}H NMR (300 MHz, D_2O): δ 1.63 (1 H, dd, $J_{3ax,3eq}$ 13.2 Hz, $J_{3ax,4}$ 11.4 Hz, H-3ax), 1.94 (3 H, s, NHCOC*H*$_3$), 2.27 (1 H, dd, $J_{3eq,3ax}$ 13.2 Hz, $J_{3eq,4}$ 4.8 Hz, H-3eq), 3.13 (3 H, s, OC*H*$_3$), 3.64 (1 H, dd, $J_{7,8}$ 9.0 Hz, $J_{7,6}$ 0.9 Hz, H-7), 3.70 (1 H, dd, $J_{6,5}$ 10.5 Hz, $J_{6,7}$ 0.9 Hz, H-6), 3.79 (1H, q, $J_{5,4} \approx J_{5,6}$ 10.5 Hz, H-5), 3.95 (1H, ddd, $J_{4,3ax}$ 11.4 Hz, $J_{4,5}$ 10.5 Hz, $J_{4,3eq}$ 4.8 Hz, H-4), 4.27 (1 H, d, *J* 9.0 Hz, H-8), 4.47-4.67 (2 H, m, H-a), 7.93-7.98 (1 H, m, Ar*H*), 8.43-8.46 (1H, m, Ar*H*), 8.59-8.61 (2 H, m, 2 x Ar*H*); ^{13}C NMR (75.5 MHz, D_2O): δ 22.0 (NHCO*C*H_3), 39.4 (C-3), 39.8 (C-a), 50.6 (O*C*H_3), 51.6 (C-5), 66.5 (C-4), 69.5 (C-6), 70.1 (C-7), 70.5 (C-8), 99.7 (C-2), 127.2 (Ar*C*), 138.7 (Ar*C*$_q$), 139.4, 139.9 and 145.6 (3 x Ar*C*), 173.3 (C-1), 174.9 (*C*ONH), 175.4 (NH*C*OCH_3); LRMS (ESI): *m/z* 426.0 [(M-H)$^-$ 100%]. HRMS calcd for $C_{18}H_{24}N_3O_9$ [M-H]$^-$ 426.1518, found 426.1498.

Methyl 5-acetamido-3,5-dideoxy-8-(glycine)carboxamido-D-*glycero*-β-D-*galacto*-oct-2-ulopyranosidonic acid (10a).
Prepared from **7a** (0.15 g, 0.27 mmol), according to the general procedure for deprotection (85 mg, 80% yield). HPLC: 0.05% TFA in 0.5:99.5 CH_3CN/H_2O; retention time 5.4-7.8 min; ^{1}H NMR (600 MHz, D_2O): δ 1.60 (1 H, dd, $J_{3ax,3eq}$ 13.2 Hz, $J_{3ax,4}$ 11.4 Hz, H-3ax), 1.89 (3 H, s, NHCOC*H*$_3$), 2.22 (1 H, dd, $J_{3eq,3ax}$ 13.2 Hz, $J_{3eq,4}$ 4.8 Hz, H-3eq), 3.10 (3 H, s, OC*H*$_3$), 3.64 (1 H, d, *J* 8.4 Hz, H-7), 3.68 (1 H, d, *J* 10.8 Hz, H-6), 3.76 (1 H, app.t, *J* 10.2 Hz, H-5), 3.85-3.94 (3 H, m, H-4 and H-a), 4.20 (1 H, d, *J* 8.4 Hz, H-8); ^{13}C NMR (150 MHz, D_2O): δ 22.0 (NHCO*C*H_3), 39.2 (C-3), 40.8 (C-a), 50.8 (O*C*H_3), 51.6 (C-5), 66.4 (C-4), 69.0 (C-6), 70.3 (C-7), 70.9 (C-8), 99.5 (C-2), 172.7 (C-1), 173.0 (*C*O_2H), 174.7 (*C*ONH), 175.1 (NH*C*OCH_3); LRMS (ESI): *m/z* 392.9 [(M-H)$^-$ 100%]; HRMS (ESI): calcd for $C_{14}H_{21}N_2NaO_{11}$ [M-H]$^-$ 393.1150, found 393.1138.

Methyl 5-acetamido-8-(L-alanine)carboxamido-3,5-dideoxy-D-*glycero*-β-D-*galacto*-oct-2-ulopyranosidonic acid (10b).
Prepared from **7b** (0.10 g, 0.18 mmol), according to the general procedure for deprotection (63 mg, 85% yield). HPLC: 0.05% TFA in 0.5:99.5 CH_3CN/H_2O; retention time 8.5-10.5 min; ^{1}H NMR (600 MHz, D_2O): δ 1.28 (3 H, d, *J* 7.2 Hz, H-b), 1.61 (1 H, app.t, $J_{3ax,3eq} \approx J_{3ax,4}$ 13.2 Hz, H-3ax), 1.88 (3 H, s, NHCOC*H*$_3$), 2.22 (1 H, dd, $J_{3eq,3ax}$ 13.2 Hz, $J_{3eq,4}$ 4.8 Hz, H-3eq), 3.10 (3 H, s, OC*H*$_3$), 3.62 (1 H, d, *J* 9.0 Hz, H-7), 3.67 (1 H, d, *J* 10.8 Hz, H-6), 3.76 (1 H, app.t, *J* 10.2 Hz, H-5), 3.88 (1 H, ddd, $J_{4,3ax}$ 13.2 Hz, $J_{4,5}$ 10.8 Hz, $J_{4,3eq}$ 4.8 Hz, H-4), 4.15 (1 H, d, *J* 9.0 Hz, H-8), 4.27 (1 H, q, *J* 7.2 Hz, H-a); ^{13}C NMR (150 MHz, D_2O): δ 16.2 (C-b), 22.0 (NHCO*C*H_3), 39.2 (C-3), 48.4 (C-a), 50.8 (O*C*H_3), 51.5 (C-5), 66.4 (C-4), 69.0 (C-6), 70.3 (C-7), 70.6 (C-8), 99.3 (C-2), 172.3 (C-1), 174.3 (*C*O_2H), 174.7 (*C*ONH), 176.1 (NH*C*OCH_3); LRMS (ESI): *m/z* 407.0 [(M-H)$^-$ 100%]; HRMS (ESI): calcd for $C_{15}H_{23}N_2O_{11}$ [M-H]$^-$ 407.1307, found 407.1296.

Methyl 5-acetamido-3,5-dideoxy-8-(L-leucine)carboxamido-D-*glycero*-β-D-*galacto*-oct-2-ulopyranosidonic acid (10c).
Prepared from **7c** (0.15 g, 0.25 mmol), according to the general procedure for deprotection (97 mg, 88% yield). HPLC: 0.05% TFA in 18:82 CH_3CN/H_2O; retention time 4.9-7.6 min; ^1H NMR (600 MHz, D_2O): δ 0.74-0.79 (2 x 3 H, 2 x d, *J* 6.6 Hz, H-d and H-d'), 1.49-1.62 (4 H, m, H-c, H-b and H-3ax), 1.89 (3 H, s, NHCOC*H*3), 2.22 (1 H, dd, $J_{3eq,3ax}$ 13.2 Hz, $J_{3eq,4}$ 4.8 Hz, H-3eq), 3.10 (3 H, s, OC*H*3), 3.59 (1H, d, *J* 9.0 Hz, H-7), 3.67 (1 H, d, *J* 10.8 Hz, H-6), 3.76 (1 H, app. t, *J* 10.2 Hz, H-5), 3.88 (1 H, ddd, $J_{4,3ax}$ 13.2 Hz, $J_{4,5}$ 10.8 Hz, $J_{4,3eq}$ 4.8 Hz, H-4), 4.15 (1 H, d, *J* 9.0 Hz, H-8), 4.28-4.30 (1 H, m, H-a); ^{13}C NMR (150 MHz, D_2O): δ 20.5 and 22.0 (C-d and C-d'), 22.0 (NHCO*C*H3), 24.4 (C-b), 39.2 (C-3), 50.7 (O*C*H3), 51.2 (C-a), 51.6 (C-5), 66.4 (C-4), 69.2 (C-6), 70.1 (C-7), 70.5 (C-8), 99.5 (C-2), 172.7 (C-1), 174.7 (*C*O2H), 174.8 (*C*ONH), 176.1 (NH*C*OCH3), [C-c was not observed]; LRMS (ESI): *m/z* 449.1 [$(M\text{-}H)^-$ 100%]; HRMS (ESI): calcd for $C_{18}H_{29}N_2O_{11}$ $[M\text{-}H]^-$ 449.1776; Found 449.1759.

Methyl 5-acetamido-3,5-dideoxy-8-(D/L-serine)carboxamido-D-*glycero*-β-D-*galacto*-oct-2-ulopyranosidonic acid (10d-1 and 10d-2).
Prepared from **7d** (0.10 g, 0.17 mmol), according to the general procedure for deprotection (62 mg, 85% yield). The two isomers were separable by HPLC: 0.05% TFA in 0.2:99.8 10:90 CH_3CN/H_2O. The stereochemistry at the serine α-carbon was not assigned. **10d-1**: retention time 7.4-8.26 min; ^1H NMR (300 MHz, D_2O): δ 1.60 (1 H, dd, $J_{3ax,3eq}$ 13.2 Hz, $J_{3ax,4}$ 11.4 Hz, H-3ax), 1.88 (3 H, s, NHCOC*H*3), 2.22 (1 H, dd, $J_{3eq,3ax}$ 13.2 Hz, $J_{3eq,4}$ 4.8 Hz, H-3eq), 3.10 (3 H, s, OC*H*3), 3.63 (1H, d, *J* 8.7 Hz, H-7), 3.65-3.92 (5 H, m, H-4, H-5, H-6 and H-b), 4.23 (1H, d, *J* 8.7 Hz, H-8), 4.43 (1H, t, *J* 4.2 Hz, H-a); ^{13}C NMR (75.5 MHz, D_2O): δ 22.0 (NHCO*C*H3), 39.2 (C-3), 50.8 (O*C*H3), 51.5 (C-5), 54.5 (C-a), 61.0 (C-b), 66.4 (C-4), 68.9 (C-6), 70.2 (C-7), 70.6 (C-8), 99.5 (C-2), 172.6 (C-1), 173.1 (*C*O2H), 174.6 (*C*ONH), 174.7 (NH*C*OCH3); LRMS (ESI): *m/z* 423.0 [$(M\text{-}H)^-$ 100%]; HRMS (ESI): calcd for $C_{15}H_{24}N_2NaO_{12}$ $[M+Na]^+$ 447.1221, found 447.1230. **10d-2**: retention time 8.4-10.1 min; ^1H NMR (300 MHz, D_2O): δ 1.61 (1 H, dd, $J_{3ax,3eq}$ 13.2 Hz, $J_{3ax,4}$ 11.4 Hz, H-3ax), 1.88 (3 H, s, NHCOC*H*3), 2.22 (1 H, dd, $J_{3eq,3ax}$ 13.2 Hz, $J_{3eq,4}$ 4.8 Hz, H-3eq), 3.11 (3 H, s, OC*H*3), 3.63 (1 H, dd, $J_{7,8}$ 9.0 Hz, H-7), 3.66-3.92 (approx. 5H, m, H-4, H-5, H-6 and H-b), 4.21 (1H, d, $J_{8,7}$ 9.0 Hz, H-8), 4.43 (1 H, t, $J_{b,a}$ 4.5 Hz, H-a); ^{13}C NMR (75.5 MHz, D_2O): δ 22.0 (NHCO*C*H3), 39.1 (C-3), 50.8 (O*C*H3), 51.5 (C-5), 54.5 (C-a), 61.0 (C-b), 66.4 (C-4), 69.0 (C-6), 70.2 (C-7), 70.4 (C-8), 99.3 (C-2), 172.2 (C-1), 173.0 (*C*O2H), 174.7 (*C*ONH), 174.7 (NH*C*OCH3); LRMS (ESI): *m/z* 423.0 [$(M\text{-}H)^-$ 100%]; HRMS (ESI): calcd for $C_{15}H_{22}N_2NaO_{12}$ $[M(COOH/COONa)\text{-}H]^-$ 445.1075, found 445.1060.

Methyl 5-acetamido-3,5-dideoxy-8-(L-phenylalanine)carboxamido-D-*glycero*-β-D-*galacto*-oct-2-ulopyranosidonic acid (10e).
Prepared from **7e** (0.15 g, 0.24 mmol), according to the general procedure for deprotection (85 mg, 75% yield). HPLC: 0.05% TFA in 16.5:83.5 CH_3CN/H_2O; retention time 15-19.8 min; ^1H NMR (300 MHz, D_2O): δ 1.63 (1 H, app.t, $J_{3ax,3eq} \approx J_{3ax,4}$ 12.9 Hz, H-3ax), 1.94 (3 H, s, NHCOC*H*3), 2.25 (1 H, dd, $J_{3eq,3ax}$ 13.2 Hz, $J_{3eq,4}$ 4.8 Hz, H-3eq), 2.95- 3.19 (5 H, m, H-b and OC*H*3), 3.53 (1 H, d, *J* 9.0 Hz, H-7), 3.65 (1 H, d, *J* 10.2 Hz, H-6), 3.76 (1 H, app.t, *J* 10.2 Hz, H-5), 3.90 (1 H, ddd, $J_{4,3ax}$ 13.2 Hz, $J_{4,5}$ 10.2 Hz, $J_{4,3eq}$ 4.8 Hz, H-4), 4.15 (1 H, d, *J* 9.0 Hz, H-8), 4.60-4.67 (1 H, m, H-a), 7.17-7.29 (5 H, m, 5 x Ar*H*); ^{13}C NMR (75.5 MHz, D_2O): δ 22.1 (NHCO*C*H3), 36.4 (C-b), 39.2 (C-3), 50.8 (O*C*H3), 51.5 (C-5), 53.7 (C-a), 66.4 (C-4), 69.1 (C-6), 70.2 (C-7), 70.3 (C-8), 99.5 (C-2), 127.1, 128.6 and 129.5 (Ar*C*), 136.4 (*ipso*-Ar*C*), 172.5 (C-1), 174.3 (*C*O2H), 174.5 (*C*ONH), 174.7 (NH*C*OCH3); LRMS (ESI): *m/z* 506.9 [$(M(COOH/COONa)+H)^+$ 100%]; HRMS (ESI): calcd for $C_{21}H_{27}N_2O_{11}$ $[M\text{-}H]^-$ 483.1620, found 483.1603.

Methyl 5-acetamido-3,5-dideoxy-8-(L-tyrosine)carboxamido-D-*glycero*-β-D-*galacto*-oct-2-ulopyranosidonic acid (10f).
Prepared from **7f** (0.10 g, 0.15 mmol), according to the general procedure for deprotection (59 mg, 77% yield). HPLC: 0.05% TFA in 8.5:91.5 20:80 CH_3CN/H_2O; retention time 8.8-18.4 min; ^1H NMR (600 MHz, D_2O): δ 1.58 (1 H, app. t, $J_{3ax,3eq} \approx J_{3ax,4}$ 12.6 Hz, H-3ax), 1.88 (3 H, s, NHCOC*H*3), 2.20 (1 H, dd, $J_{3eq,3ax}$ 13.2 Hz, $J_{3eq,4}$ 4.8 Hz, H-3eq), 2.85 (1 H, dd, *J* 7.8 Hz, *J* 13.8 Hz, H-b), 2.99-3.04 (4 H, m, H-b' and OC*H*3), 3.51 (1 H, d, *J* 9.0 Hz, H-7), 3.60 (1 H, d, *J* 10.2 Hz, H-6), 3.71 (1 H, app.t, *J* 10.2 Hz, H-5), 3.85 (1 H, ddd, $J_{4,3ax}$ 13.2 Hz, $J_{4,5}$ 10.2 Hz, $J_{4,3eq}$ 4.8 Hz, H-4), 4.08 (1 H, d, *J* 9.0 Hz, H-8), 4.49-4.51 (1 H, m, H-a), 6.68 (1 H, d, *J* 8.4 Hz, 2 x Ar*H*), 6.99 (1 H, d, *J* 8.4 Hz, 2 x Ar*H*); ^{13}C NMR (150 MHz, D_2O): δ 22.0 (NHCO*C*H3), 35.6 (C-b), 39.2 (C-3), 50.7 (O*C*H3), 51.5 (C-5), 53.9 (C-a), 66.4 (C-4), 69.1 (C-6), 70.1 (C-7), 70.4 (C-8), 99.5 (C-2), 115.3 (2 x Ar*C*), 128.1 (Ar*C*q), 130.5 (2 x Ar*C*), 154.3 (Ar*C*q), 172.6 (C-1), 174.3 (*C*O2H), 174.5 (*C*ONH), 174.6 (NH*C*OCH3); LRMS (ESI): *m/z* 499.0 [$(M\text{-}H)^-$ 100%]; HRMS (ESI): calcd for $C_{21}H_{27}N_2O_{12}$ $[M\text{-}H]^-$ 499.1569, found 499.1571.

Methyl 5-acetamido-3,5-dideoxy-8-(L-histidine)carboxamido-D-*glycero*-β-D-*galacto*-oct-2-ulopyranosidonic acid (10g).
Prepared from **7g** (0.10 g, 0.16 mmol), according to the general procedure for deprotection (60 mg, 80% yield). HPLC: 0.05% TFA in 0.6:99.4 CH_3CN/H_2O; retention time 4.8-7.7 min; ^1H NMR (600 MHz, D_2O): δ 1.57 (1 H, app.t, $J_{3ax,3eq} \approx J_{3ax,4}$ 12.6 Hz, H-

3ax), 1.88 (3 H, s, NHCOCH_3), 2.20 (1 H, dd, $J_{3eq,3ax}$ 13.2 Hz, $J_{3eq,4}$ 4.8 Hz, H-3eq), 3.06 (3 H, s, OCH_3), 3.16 (1 H, dd, *J* 7.2 Hz, H-b), 3.26 (1 H, dd, *J* 4.8 Hz, *J* 5.4 Hz, H-b'), 3.52 (1 H, d, *J* 9.6 Hz, H-7), 3.63 (1 H, d, *J* 10.8 Hz, H-6), 3.76 (1 H, m, H-5), 3.87 (1 H, ddd, $J_{4,3ax}$ 13.2 Hz, $J_{4,5}$ 10.8 Hz, $J_{4,3eq}$ 4.8 Hz, H-4), 4.10 (1 H, d, *J* 9.0 Hz, H-8), 4.59-4.61 (0.4 H, m, H-a), 7.13 and 8.44 (2 x 1 H, 2 x s, 2 x Ar*H*); ^{13}C NMR (150 MHz, D_2O): δ 22.0 (NHCOCH_3), 26.0 (C-b), 39.2 (C-3), 50.7 (OCH_3), 51.5 (C-5), 51.6 (C-a), 66.3 (C-4), 69.2 (C-6), 70.1 (C-7), 70.3 (C-8), 99.4 (C-2), 117.0, 117.2 (Ar*C*), 128.6 (Ar*C*$_q$), 172.6 (C-1), 173.1 (*C*O_2H), 174.5 (*C*ONH), 174.8 (NH*C*OCH_3); LRMS (ESI): *m/z* 473.0 [(M-H)$^-$ 60%]; HRMS (ESI): calcd for $C_{18}H_{25}N_4O_{11}$ [M-H]$^-$ 473.1525, found 473.1519.

Methyl 5-acetamido-3,5-dideoxy-8-(L-tryptophan)carboxamido-D-*glycero*-β-D-*galacto*-oct-2-ulopyranosidonic acid (10h).

Prepared from **7h** (0.15 g, 0.22 mmol), according to the general procedure for deprotection (86 mg, 75% yield). HPLC: 0.05% TFA in 16.5:83.5 CH_3CN/H_2O; retention time 11.3-18.6 min; ^{1}H NMR (600 MHz, D_2O): δ 1.54 (1 H, app.t, $J_{3ax,3eq} \approx J_{3ax,4}$ 12.6 Hz, H-3ax), 1.85 (3 H, s, NHCOCH_3), 2.18 (1 H, dd, $J_{3eq,3ax}$ 13.2 Hz, $J_{3eq,4}$ 4.8 Hz, H-3eq), 2.97 (3 H, s, OCH_3), 3.16 (1H, dd, *J* 7.2 Hz, H-b), 3.26 (1 H, dd, *J* 4.8 Hz, *J* 5.4 Hz, H-b'), 3.51-3.52 (2 H, m, H-6 and H-7), 3.69 (1 H, app.t, *J* 10.2 Hz, H-5), 3.77-3.81 (1 H, m, H-4), 4.08 (1 H, d, *J* 8.4 Hz, H-8), 4.62 (0.2 H, t, *J* 7.2 Hz, H-a), 7.00 (1 H, app.t, *J* 7.8 Hz, Ar*H*), 7.07-7.11 (2 H, m, Ar*H*), 7.34 (1 H, d, *J* 7.8 Hz, Ar*H*), 7.53 (1 H, d, *J* 7.8 Hz, Ar*H*); ^{13}C NMR (150 MHz, D_2O): δ 22.0 (NHCOCH_3), 26.5 (C-b), 39.2 (C-3), 50.6 (OCH_3), 51.5 (C-5), 53.3 (C-a), 66.4 (C-4), 69.0 (C-6), 70.2 (C-7), 70.8 (C-8), 99.7 (C-2), 108.8 (Ar*C*$_q$), 111.8, 118.3, 119.2, 121.9, and 124.4 (5 x Ar*C*), 126.7, 136.0 (2 x Ar*C*$_q$), 172.9 (*C*O_2H), 174.2 (*C*ONH), 174.9 (NH*C*OCH_3), [C-1 was not observed]; LRMS (ESI): *m/z* 522.0 [(M-H)$^-$ 100%]; HRMS (ESI): calcd for $C_{23}H_{28}N_3O_{11}$ [M-H]$^-$ 522.1729, found 522.1705.

Synthesis of C-7 ether derivatives of Neu5Acβ2Me (3)

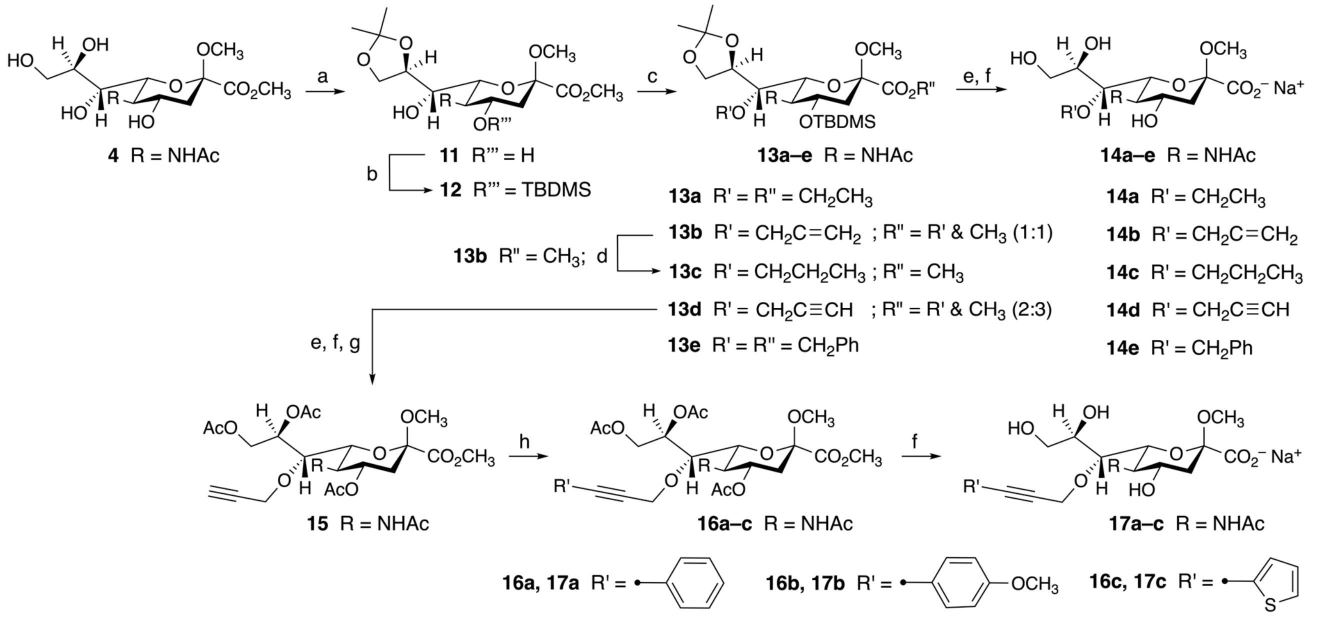

Scheme S2. Synthesis of C-7 *O*-alkylated derivatives of Neu5Acβ2Me. *Reagents and conditions:* (a) 2,2-dimethoxypropane, H$^+$ ion exchange resin, acetone, rt, 16 h (90%); (b) *t*-BuMe$_2$SiCl, pyridine, 0 °C – rt, 16 h (58%); (c) R^{1}X, NaH, TBAI, DMF, 0 °C – rt 16 h (**13a** 56%, **13b** 60%, **13d** 60%, **13e** 36%); (d) H_2, 10% Pd/C, MeOH, rt, 16 h (**13c** 87%); (e) 80% aq. AcOH, 80 °C, 2 h; (f) NaOH, MeOH/H_2O (1:1), pH 13, 0 °C – rt, 16 h; (g) (i) MeOH, H$^+$ ion exchange resin, MW (100 W), 80 ˚C, 15 min; (ii) Ac_2O, pyridine, 16 h (88% over 4 steps from **13d**); (h) R'I, $PdCl_2(PPh_3)_2$, CuI, Et_3N, CH_3CN, rt, 2-6 h (**16a** 60%, **16b** 87%, **16c** 85%). In **16**,**17a–c**, the sphere indicates the point of attachment of R' to the alkyne carbon.

Methyl (methyl 5-acetamido-3,5-dideoxy-8,9-*O*-isopropylidene-D-*glycero*-β-D-*galacto*-2-nonulopyranosid)onate (11).

To a stirring suspension of **4** (3.0 g, 8.90 mmol) in acetone (60 mL) under argon was added 2,2-dimethoxypropane (10.91 mL, 89.0 mmol) and a catalytic amount of Amberlite® IR-120 (H$^+$) resin. The reaction mixture was stirred at room temperature for 16 h. The resin was filtered off and washed with acetone. The combined filtrate was evaporated under reduced pressure to afford a syrup. Column chromatography on silica gel (EtOAc/MeOH, 1:0 10:1) gave **11** [10] (3.0 g, 90%) as a white solid. R_f 0.54 (EtOAc/MeOH 10:1); ^{1}H NMR (300 MHz, $CDCl_3$): δ 1.27 and 1.35 [2 x 3 H, 2 s, C(CH_3)$_2$], 1.74 (1 H, dd, $J_{3eq,3ax}$ 12.9 Hz, $J_{3ax,4}$ 10.8 Hz, H-3ax), 2.05 (3 H, s, NHCOCH_3), 2.43 (1 H, dd, $J_{3eq,3ax}$ 12.9 Hz, $J_{3eq,4}$ 4.8 Hz, H-3eq), 3.23 (3 H, s, OCH_3), 3.45 (1 H,

dd, $J_{7,8}$ 8.4 Hz, $J_{6,7}$ 1.5 Hz, H-7), 3.56 (1 H, dd, $J_{6,5}$ 10.2 Hz, $J_{6,7}$ 1.5 Hz, H-6), 3.78 (3 H, s, CO_2CH_3), 3.84-3.90 (1 H, m, H-5), 3.95-4.14 (3 H, m, H-4, H-9A and H-9B), 4.23-4.29 (1 H, m, H-8), 6.09 (1 H, d, $J_{NH,5}$ 8.1 Hz, N*H*COCH$_3$); LRMS (ESI): *m/z* 377.7 [(M)$^+$ 100%].

Methyl (methyl 5-acetamido-4-*O*-*tert*-butyldimethylsilyl-3,5-dideoxy-8,9-*O*-isopropylidene-D-*glycero*-β-D-*galacto*-2-nonulopyranosid)onate (12).

To a solution of **11** (3.0 g, 8.08 mmol) in pyridine (20 mL) at 0 °C under argon was added *tert*-butyldimethylsilyl chloride (2.19 g, 14.55 mmol). After stirring the solution for 16 h at room temperature, MeOH (3 mL) was added and co-evaporated with toluene (30 mL) to afford a syrup. Column chromatography on silica gel (hexane/EtOAc, 1:1 1:4) gave **12** [11] (2.23 g, 58%) as a white solid. R_f 0.54 (hexane/EtOAc 1:4); ^{1}H NMR (300 MHz, $CDCl_3$): δ 0.07 [6 H, s, Si(C*H*$_3$)$_2$], 0.83 [9 H, s, SiC(C*H*$_3$)$_3$], 1.27 and 1.35 [2 x 3 H, 2 s, C(C*H*$_3$)$_2$], 1.75 (1 H, dd, $J_{3ax,3eq}$ 13.2 Hz, $J_{3ax,4}$ 10.8 Hz, H-3ax), 2.02 (3 H, s, NHCOC*H*$_3$), 2.30 (1 H, dd, $J_{3eq,3ax}$ 13.2 Hz, $J_{3eq,4}$ 5.1 Hz, H-3eq), 3.28 (3 H, s, OC*H*$_3$), 3.44 (1 H, dd, $J_{7,8}$ 8.4 Hz, $J_{6,7}$ 1.5 Hz, H-7), 3.54 (1 H, dd, $J_{6,5}$ 10.5 Hz, $J_{6,7}$ 1.5 Hz, H-6), 3.79 (3 H, s, CO_2CH_3), 3.80-3.90 (1 H, m, H-5), 3.96-4.08 (2 H, m, H-4 and H-9A), 4.12 (1 H, dd, $J_{9B,9A}$ 8.7 Hz, $J_{9B,8}$ 6.3 Hz, H-9B), 4.30 (1 H, m, H-8), 5.22 (1 H, d, $J_{NH,5}$ 8.1 Hz, N*H*COCH$_3$); LRMS (ESI): *m/z* 489.8 [(M-H)$^-$ 100%].

Ethyl (methyl 5-acetamido-4-*O*-*tert*-butyldimethylsilyl-3,5-dideoxy-7-*O*-ethyl-8,9-*O*-isopropylidene-D-*glycero*-β-D-*galacto*-2-nonulopyranosid)onate (13a).

To a stirring solution of **12** (0.2 g, 0.4 mmol), ethyl iodide (0.06 mL, 0.8 mmol) and TBAI (0.03 g, 0.08 mmol) in dry DMF (5 mL) at 0 °C under argon, was added sodium hydride (0.03 g, 0.8 mmol). The mixture was stirred at rt, and the progress of reaction was monitored by TLC (hexane/EtOAc, 1:1). After 16 h, the reaction mixture was cooled to 0 °C and a drop of glacial acetic acid was added followed by addition of EtOAc (25 mL). The solution was washed with satd $NaHCO_3$, satd NaCl, dried (Na_2SO_4) and concentrated under reduced pressure to afford a syrup. The crude product was purified by flash chromatography (hexane/EtOAc, 1:0 1:1) to afford **13a** [12, 13] (120 mg, 56%) as a white solid. R_f 0.56 (hexane/EtOAc 1:1); ^{1}H NMR (300 MHz, $CDCl_3$): δ 0.02 and 0.03 [2 x 3 H, 2 s, 2 x Si(C*H*$_3$)$_2$], 0.83 [9 H, s, SiC(C*H*$_3$)$_3$], 1.12 and 1.28 (2 x 3 H, t, *J* 7.2 Hz, H-b and H-b'), 1.29 and 1.38 [2 x 3 H, 2 s, 2 x C(C*H*$_3$)$_2$], 1.70 (1 H, dd, $J_{3ax,3eq}$ 12.9 Hz, $J_{3ax,4}$ 10.8 Hz, H-3ax), 1.94 (3 H, s, NHCOC*H*$_3$), 2.24 (1 H, dd, $J_{3eq,3ax}$ 12.9 Hz, $J_{3eq,4}$ 5.1 Hz, H-3eq), 3.19 (3 H, s, OC*H*$_3$), 3.61-3.90 (5 H, m), 3.98-4.13 (3 H, m), 4.18-4.25 (3 H, m), 5.16 (0.8 H, d, $J_{NH,5}$ 7.8 Hz, N*H*COCH$_3$); ^{13}C NMR (75.5 MHz, $CDCl_3$): δ -4.3 and -4.7 [Si(*C*H$_3$)$_2$], 14.1 and 15.8 (C-b and C-b'), 17.8 [Si*C*(CH$_3$)$_3$], 23.7 (NHCO*C*H$_3$), 25.3 and 26.6 [C(*C*H$_3$)$_2$], 25.6 [SiC(*C*H$_3$)$_3$], 40.9 (C-3), 50.7 (O*C*H$_3$), 53.4 (C-5), 61.7 (C-a), 66.3 (C-9), 68.1 (C-4*), 68.9 (C-6*), 71.6 (C-7*), 98.9 (C-2), 108.0 [*C*(CH$_3$)$_2$], 167.8 and 169.5 (C-1 and NH*C*OCH$_3$), [C-a' and C-8 were not observed].

Methyl/Prop-2-enyl [methyl 5-acetamido-4-*O*-*tert*-butyldimethylsilyl-3,5-dideoxy-8,9-*O*-isopropylidene-7-*O*-(prop-2-enyl)-D-*glycero*-β-D-*galacto*-2-nonulopyranosid]onate (13b).

Compound **13b** [14] was prepared by alkylation of **12** (0.1 g, 0.2 mmol) with allyl bromide (0.03 mL, 0.4 mmol) in a similar manner to that described for the synthesis of **13a** from **12**. The crude reaction product was purified by flash chromatography (hexane/EtOAc, 1:0 1:1) to give the 7-*O*-allylated product **13b** as a mixture (~ 1:1) of the methyl and allyl esters (0.065 g, 60%) as a white solid. An inseparable impurity of allyl alcohol was also present in the isolated product. R_f 0.46 (hexane/EtOAc 1:1); **13b** methyl ester: ^{1}H NMR (300 MHz, $CDCl_3$): δ 0.02, 0.3 [2 x 3 H, 2 x s, Si(C*H*$_3$)$_2$], 0.83 [9 H, s, SiC(C*H*$_3$)$_3$], 1.29 and 1.38 [2 x 3 H, 2 x s, 2 x C(C*H*$_3$)$_2$], 1.69 (1 H, dd, $J_{3ax,3eq}$ 13.2 Hz, $J_{3ax,4}$ 11.4 Hz, H-3ax), 1.94 (3 H, s, NHCOC*H*$_3$), 2.25 (1 H, dd, $J_{3eq,3ax}$ 13.2 Hz, $J_{3eq,4}$ 5.1 Hz, H-3eq), 3.19 (3H, s, OC*H*$_3$), 3.70 (1 H, m, H-7), 3.75 (4 H, m, H-6 and CO_2CH_3), 3.91-4.26 (7 H, m, H-4, H-5, H-8, H-9A, H-9B and H-a), 5.14 (2 H, d, *J* 9.3 Hz, H-c), 5.89-6.02 (1 H, m, H-b); ^{13}C NMR (75.5 MHz, $CDCl_3$): δ -4.8 and -4.3 [2 x Si(*C*H$_3$)$_2$], 17.8 [Si*C*(CH$_3$)$_3$], 23.8 (NHCO*C*H$_3$), 25.3 and 26.6 [2 x C(*C*H$_3$)$_2$], 25.6 [SiC(*C*H$_3$)$_3$], 41.0 (C-3), 50.9 (O*C*H$_3$), 52.5 (CO$_2$*C*H$_3$), 53.6 (C-5), 66.3 (C-9), 67.7 (C-4*), 71.4 (C-6*), 74.1 (C-a), 76.3 (C-7*), 76.8 (C-8*), 99.0 (C-2), 108.1 [*C*(CH$_3$)$_2$], 116.9 (C-c), 135.3 (C-b), 168.4 and 169.5 (C-1 and N*H*COCH$_3$); LRMS (ESI): *m/z* 554.2 ([M+Na]$^+$ 100%).

Methyl (methyl 5-acetamido-4-*O*-*tert*-butyldimethylsilyl-3,5-dideoxy-8,9-*O*-isopropylidene-7-*O*-propyl-D-*glycero*-β-D-*galacto*-2-nonulopyranosid)onate (13c).

A mixture of **13b** methyl ester (0.15 g, 0.28 mmol) and 10% Pd/C (15 mg, 10% wt. of **13b**) in MeOH (5 mL) was stirred under an H_2 atmosphere for 16 h. The reaction mixture was filtered through celite, the filtrate was concentrated under reduced pressure and the residue purified by flash chromatography (hexane/EtOAc, 1:0 1:1) to afford **13c** [12, 13] (125 g, 87%) as a white solid. R_f 0.68 (hexane/EtOAc 1:1); ^{1}H NMR (300 MHz, $CDCl_3$): δ 0.02, 0.03 [2 x 3 H, 2 x s, Si(C*H*$_3$)$_2$], 0.83 [9 H, s, SiC(C*H*$_3$)$_3$], 0.89 (3 H, t, *J* 7.5 Hz, H-c), 1.29 and 1.38 [2 x 3 H, 2 x s, 2 x C(C*H*$_3$)$_2$], 1.58-1.75 (3 H, m, H-3ax and H-b), 1.95 (3 H, s, NHCOC*H*$_3$), 2.25 (1 H, dd, $J_{3eq,3ax}$ 12.3 Hz, $J_{3eq,4}$ 4.8 Hz, H-3eq), 3.20 (3H, s, OC*H*$_3$), 3.52-3.64 (2 H, m, H-a), 3.76 (3 H, s, CO_2CH_3), 3.70-

4.23 (7 H, m, H-4, H-5, H-6, H-7, H-8, H-9A and H-9B), 5.10 (1 H, d, *J* 7.8 Hz, N*H*COCH$_3$); ^{13}C NMR (75.5 MHz, CDCl$_3$): δ -4.8 and -4.3 [2 x Si(*C*H$_3$)$_2$], 10.5 (C-c), 17.8 [Si*C*(CH$_3$)$_3$], 23.6 (C-b), 23.7 (NHCOC*H*$_3$), 25.3 and 26.6 [2 x C(*C*H$_3$)$_2$], 25.7 [SiC(*C*H$_3$)$_3$], 41.1 (C-3), 50.9 (O*C*H$_3$), 52.6 (CO$_2$CH$_3$), 53.3 (C-5), 66.5 (C-9), 68.4 (C-4*), 71.6 (C-6*), 75.0 (C-a), 76.4 (C-7*), 77.1 (C-8*), 99.0 (C-2), 108.1 [*C*(CH$_3$)$_2$], 168.5, 169.5 (C-1 and N*H*COCH$_3$).

Methyl/Prop-2-ynyl [methyl 5-acetamido-4-*O*-*tert*-butyldimethylsilyl-3,5-dideoxy-8,9-*O*-isopropylidene-7-*O*-(prop-2-ynyl)-D-*glycero*-β-D-*galacto*-2-nonulopyranosid]onate (**13d**).

Compound **13d** was prepared by alkylation of **12** (1.0 g, 2.04 mmol) with propargyl bromide (2.77 mL, 14.3 mmol) in a similar manner to that described for the synthesis of **13a** from **12**. The crude reaction product was purified by flash chromatography (hexane/EtOAc, 1:0 1:1) to afford the 7-*O*-propargylated product **13d** as a mixture (~ 3:2) of the methyl and propargyl esters (0.65 g, 60%) as a white solid; R_f 0.59 (hexane/EtOAc, 1:1); **13d** methyl/propargyl ester: ^{1}H NMR (300 MHz, CDCl$_3$): δ 0.00 and 0.01 [2 x 3 H, 2 x s, Si(C*H*$_3$)$_2$], 0.81 [9 H, s, SiC(C*H*$_3$)$_3$], 1.27 and 1.36 [2 x 3 H, 2 s, 2 x C(C*H*$_3$)$_2$], 1.57-1.66 (1 H, m, H-3ax), 1.94 (3 H, s, NHCOC*H*$_3$), 2.22-2.31 (1 H, m, H-3eq), 2.45-2.47 (1 H, m, H-c), 3.16 and 3.19† (3 H, s, OC*H*$_3$), 3.41-3.52 (1 H, m, H-5), 3.73 (3 H, s, CO$_2$C*H*$_3$), 3.73-3.97 (1 H, m, H-7), 4.08-4.23 (4 H, m, H-6, H-8, H-9A and H-9B), 4.32-4.54 (3 H, m, H-a and H-4), 5.63 (1 H, m, N*H*COCH$_3$); ^{13}C NMR (75.5 MHz, CDCl$_3$): δ -4.8 and -4.4 [2 x Si(*C*H$_3$)$_2$], 17.8 [Si*C*(CH$_3$)$_3$], 23.8 (NHCO*C*H$_3$), 25.0, 25.0† and 26.5, 26.5† [2 x C(*C*H$_3$)$_2$], 25.6, 25.6† [SiC(*C*H$_3$)$_3$], [29.6 (not assigned)], 41.1, 41.3† (C-3), 50.8, 50.9† (O*C*H$_3$), 52.5 (CO$_2$*C*H$_3$), [52.7 (not assigned)], 54.6, 54.7† (C-5), 59.6, 59.7† (C-a), 65.8, 65.8† (C-9), 66.2, 66.4† (C-4), 70.5, 70.6† (C-6*), 74.5 (C-b), 75.3, 75.5† (C-7*), 75.5† (C-b), 77.0, 77.1† (C-8*), 99.0, 99.1† (C-2), 107.9 and 108.0† [*C*(CH$_3$)$_2$], 167.0, 168.3†(C-1), 169.9, 169.9† (NH*C*OCH$_3$), [C-c was not assigned] [†Assignment belongs to trans-esterified product].

Benzyl (methyl 5-acetamido-7-*O*-benzyl-4-*O*-*tert*-butyldimethylsilyl-3,5-dideoxy-8,9-*O*-isopropylidene-D-*glycero*-β-D-*galacto*-non-2-ulopyranosid)onate (13e).

Compound **13e** was prepared by alkylation of **12** (0.2 g, 0.40 mmol) with benzyl bromide (0.95 mL, 0.8 mmol) in a similar manner to that described for the synthesis of **13a** from **12**. The crude reaction product was purified by flash chromatography (hexane/EtOAc, 1:0 1:1) to afford the desired compound **13e** (85 mg, 36%) as a white solid. R_f 0.84 (hexane/EtOAc 1:1); ^{1}H NMR (300 MHz, CDCl$_3$): δ -0.06 and -0.01 [2 x 3 H, 2 x s, Si(C*H*$_3$)$_2$], 0.81 [9 H, s, SiC(C*H*$_3$)$_3$], 1.31 and 1.43 [2 x 3 H, 2 x s, 2 x C(C*H*$_3$)$_2$], 1.59 (1 H, dd, $J_{3ax,3eq}$ 12.9 Hz, $J_{3ax,4}$ 10.8 Hz, H-3ax), 1.67 (3 H, s, NHCOC*H*$_3$), 2.24 (1 H, dd, $J_{3eq,3ax}$ 12.9 Hz, $J_{3eq,4}$ 7.8 Hz, H-3eq), 3.10 (3 H, s, OC*H*$_3$), 3.18 (1 H, m, H-5), 3.83-4.17 (5 H, m, H-6, H-7, H-8, H-9A and H-9B), 4.35-4.39 (2 H, m, H-4 and N*H*COCH$_3$), 4.74 (1 H, d, $J_{a,b}$ 12.0 Hz, H-a), 4.84 (1 H, d, $J_{b,a}$ 12.0 Hz, H-b), 5.16 (1 H, d, $J_{a',b'}$ 12.3 Hz, H-a'), 5.22 (1 H, d, $J_{b',a'}$ 12.3 Hz, H-b'), 7.28-7.45 (10 H, m, 10 x Ar*H*); ^{13}C NMR (75.5 MHz, D$_2$O): δ -4.3 and -4.9 [2 x Si(*C*H$_3$)$_2$], 17.8 [Si*C*(CH$_3$)$_3$], 23.8 (NHCO*C*H$_3$), 25.2 and 25.6 [2 x C(*C*H$_3$)$_2$], 26.6 [SiC(*C*H$_3$)$_3$], 41.1 (C-3), 50.8 (O*C*H$_3$), 55.0 (C-5), 65.8 (C-9), 65.8 (C-4*), 67.1 (C-6*), 70.5 (C-7*), 73.8, 74.3 (C-a, C-a'), 78.2 (C-8*), 98.9 (C-2), 107.8 [*C*(CH$_3$)$_2$], 127.8, 128.2, 128.4, 128.4, 128.5, 128.6 and 129.0 (10 x Ar*C*), 135.3 and 138.9 (*ipso*-Ar), 167.6, 169.7 (C-1, NH*C*OCH$_3$).

General procedure for global deprotection of compounds 13a-e.

A solution of protected 7-*O*-ether in 80% acetic acid was heated at 80 °C. After 2 h the reaction mixture was concentrated under vacuum and co-evaporated with toluene (3 x 10 mL) to remove traces of acid. The crude mixture was dissolved in MeOH:H$_2$O (1:1) and the temperature adjusted to 0 °C, followed by addition of aq NaOH (2.0 M) to pH 13. The reaction mixture was stirred at rt and monitored by TLC (Hexane/EtOAc, 1:5). After 16 h, the reaction mixture was acidified to pH 8 with Amberlite® IR-120 (H^+) resin, the resin was filtered-off and the filtrate evaporated under reduced pressure to afford a yellow syrup. The crude product was purified by column chromatography (EtOAc/MeOH/H$_2$O, 7:2:1) to give a white solid, which was further purified on RP-HPLC and then lyophilized. Where TFA was used in the eluent during RP-HPLC purification, isolated sodium salts were converted to the free acid form.

Methyl 5-acetamido-3,5-dideoxy-7-*O*-ethyl-D-*glycero*-β-D-*galacto*-non-2-ulopyranosidonic acid (14a).

Prepared from **13a** (0.12 g, 0.20 mmol), according to the general procedure for global deprotection (61 mg, 85% yield). HPLC: 0.05% TFA in 0.3:99.7 CH$_3$CN/H$_2$O; retention time 11.0-16.5 min; ^{1}H NMR (300 MHz, D$_2$O): δ 1.06 (3 H, t, *J* 6.9 Hz, H-b), 1.64 (1 H, dd, $J_{3ax,3eq}$ 12.9 Hz, $J_{3ax,4}$ 10.8 Hz, H-3ax), 1.91 (3 H, s, NHCOC*H*$_3$), 2.19 (1 H, dd, $J_{3eq,3ax}$ 12.9 Hz, $J_{3eq,4}$ 9.3 Hz, H-3eq), 3.13 (3 H, s, OC*H*$_3$), 3.40-3.65 (4 H, m, H-9A, H-9B and H-a), 3.70-3.84 (5 H, m, H-4, H-5, H-6, H-7 and H-8); ^{13}C NMR (75.5 MHz, D$_2$O): δ 14.4 (C-b), 22.1 (NHCO*C*H$_3$), 39.0 (C-3), 50.8 (O*C*H$_3$), 51.5 (C-5), 62.5 (C-a), 67.1 (C-9), 69.2 (C-4*), 69.9 (C-7*), 70.4 (C-6*), 76.3 (C-8*), 99.2 (C-2), 172.1, 174.2 (C-1, NH*C*OCH$_3$); LRMS (ESI): *m/z* 349.9 ($[M-H]^-$ 100%); HRMS (ESI): calcd for $C_{14}H_{24}NO_9$ $[M-H]^-$ 350.1457, found 350.1453.

Methyl 5-acetamido-3,5-dideoxy-7-*O*-(prop-2-enyl)-D-*glycero*-β-D-*galacto*-non-2-ulopyranosidonic acid (14b).
Prepared from **13b** (0.06 g, 0.11 mmol), according to the general procedure for global deprotection (38 mg, 95% yield). HPLC: 0.05% TFA in 1.7:98.3 CH_3CN/H_2O; retention time 13.5-16.0 min; ^{1}H NMR (300 MHz, D_2O): δ 1.56-1.66 (1 H, m, H-3ax), 1.93 (3 H, s, NHCOC*H*$_3$), 2.20 (1 H, dd, $J_{3eq,3ax}$ 12.6 Hz, $J_{3eq,4}$ 4.2 Hz, H-3eq), 3.12 (3 H, s, OC*H*$_3$), 3.47 (1 H, d, $J_{7,8}$ 8.7 Hz, H-7), 3.56 (1 H, dd, $J_{9A,9B}$ 12.0 Hz, $J_{9A,8}$ 5.1 Hz, H-9A), 3.75 (1 H, dd, $J_{9B,9A}$ 12.0 Hz, $J_{9B,8}$ 3.3 Hz, H-9B), 3.78-3.84 (4 H, m, H-4, H-5, H-6 and H-8), 3.90-4.50 (2 H, m, H-a), 5.13-5.32 (2 H, m, H-c), 5.79-5.92 (1 H, m, H-b); ^{13}C NMR (75.5 MHz, D_2O): δ 22.1 (NHCO*C*H$_3$), 39.1 (C-3), 50.8 (O*C*H$_3$), 51.6 (C-5), 62.5 (C-9), 67.3 (C-4*), 69.9 (C-7*), 70.4 (C-6*), 74.3 (C-a), 76.3 (C-8*), 99.4 (C-2), 119.2 (C-c), 133.2 (C-b), 174.2 (NH*C*OCH$_3$), [C-1 was not observed]; LRMS (ESI): *m/z* 361.9 [(M-H)$^-$ 100%]; HRMS (ESI): calcd for $C_{15}H_{24}NO_9$ [M-H]$^-$ 362.1457, found 362.1455.

Methyl 5-acetamido-3,5-dideoxy-7-*O*-propyl-D-*glycero*-β-D-*galacto*-non-2-ulopyranosidonic acid, sodium salt (14c).
Prepared from **13c** (0.12 g, 0.24 mmol), according to the general procedure for global deprotection. The crude mixture was first purified on a Grace Pure^{TM} SPE C18-Aq (5000 mg/20mL) column and finally on a Sephadex^{TM} LH-20 column to give **14c** (80 mg, 90%) as a white solid. ^{1}H NMR (300 MHz, D_2O): δ 0.93 (3 H, t, $J_{b,c}$ 7.5 Hz, H-c), 1.55-1.74 (3 H, m, H-b and H-3ax), 2.06 (3 H, s, NHCOC*H*$_3$), 2.35 (1 H, dd, $J_{3eq,3ax}$ 13.2 Hz, $J_{3eq,4}$ 4.5 Hz, H-3eq), 3.23 (3 H, s, OC*H*$_3$), 3.51-3.61 (3 H, m, H-7 and H-a), 3.69 (1 H, dd, $J_{9A,9B}$ 12.0 Hz, $J_{9A,8}$ 5.4 Hz, H-9A), 3.85-3.98 (5 H, m, H-4, H-5, H-6, H-8 and H-9B); ^{13}C NMR (75.5 MHz, D_2O): δ 9.8 (C-c), 22.2 (NHCO*C*H$_3$), 22.6 (C-b), 39.4 (C-3), 50.7 (O*C*H$_3$), 51.7 (C-5), 62.6 (C-9), 67.6 (C-4), 70.0 (C-7*), 70.3 (C-6*), 75.2 (C-a), 76.3 (C-8*), 174.2 (NH*C*OCH$_3$), [C-1 and C-2 were not observed]; LRMS (ESI): *m/z* 363.9 [(M-Na)$^-$ 100%]; HRMS (ESI): calcd for $C_{15}H_{26}NO_9$ [M-Na]$^-$ 364.1613, found 364.1612.

Methyl 5-acetamido-3,5-dideoxy-7-*O*-(prop-2-ynyl)-D-*glycero*-β-D-*galacto*-non-2-ulopyranosidonic acid, sodium salt (14d).
Prepared from **13d** (0.14 g, 0.25 mmol), according to the general procedure for global deprotection (85 mg, 89% yield). HPLC: 0.1:99.9 CH_3CN/H_2O; retention time 5.9-6.5 min; ^{1}H NMR (300 MHz, D_2O): δ 1.67 (1 H, dd, $J_{3ax,3eq}$ 12.9 Hz, $J_{3ax,4}$ 11.1 Hz, H-3ax), 2.07 (3 H, s, NHCOC*H*$_3$), 2.32 (1 H, dd, $J_{3eq,3ax}$ 12.9 Hz, $J_{3eq,4}$ 4.5 Hz, H-3eq), 2.95 (0.2 H, app.t, *J* 2.4 Hz, H-c), 3.20 (3 H, s, OC*H*$_3$), 3.67 (1 H, dd, $J_{7,8}$ 8.1 Hz, $J_{7,6}$ 0.6 Hz, H-7), 3.72 (1 H, dd, $J_{9A,9B}$ 12.0 Hz, $J_{9A,8}$ 5.7 Hz, H-9A), 3.82-3.96 (4 H, m, H-5, H-6, H-8 and H-9B), 3.97-4.02 (1 H, m, H-4), 4.34 (2 H, s, H-a); ^{13}C NMR (75.5 MHz, D_2O): δ 22.2 (NHCO*C*H$_3$), 39.6 (C-3), 50.5 (O*C*H$_3$), 51.8 (C-5), 59.8 (C-a), 62.6 (C-9), 67.5 (C-4), 70.1 (C-6, C-8), 76.6 (C-7), 78.3 (C-b), 100.5 (C-2), 174.2 (C-1), 175.0 (NH*C*OCH$_3$), [C-c was not observed]; LRMS (ESI): *m/z* 360.1 [(M-Na)$^-$ 100%].

Methyl 5-acetamido-7-*O*-benzyl-3,5-dideoxy-D-*glycero*-β-D-*galacto*-non-2-ulopyranosidonic acid (14e).
Prepared from **13e** (0.08 g, 0.13 mmol), according to the general procedure for global deprotection (45 mg, 85% yield). HPLC: 0.05% TFA in 10:90 CH_3CN/H_2O; retention time 14.0-16.5 min; ^{1}H NMR (300 MHz, D_2O): δ 1.56 (1 H, app.t, $J_{3eq,3ax} \approx J_{3ax,4}$ 12.9 Hz, H-3ax), 1.97 (3 H, s, NHCOC*H*$_3$), 2.20 (1 H, dd, $J_{3eq,3ax}$ 12.9 Hz, $J_{3eq,4}$ 4.5 Hz, H-3eq), 3.11 (3 H, s, OC*H*$_3$), 3.64-3.70 (2 H, m, H-5 and H-7), 3.79-3.96 (5 H, m, H-4, H-6, H-8, H-9A and H-9B), 4.49 (2 H, app. AB q, *J* 9.6 Hz, H-a), 7.29-7.40 (5 H, m, Ar*H*); ^{13}C NMR (75.5 MHz, D_2O): δ 22.2 (NHCO*C*H$_3$), 39.2 (C-3), 50.7 (O*C*H$_3$), 51.6 (C-5), 62.6 (C-9), 67.4 (C-4), 70.0 (C-7*), 70.9 (C-6*), 74.9 (C-8*), 76.1 (C-a), 128.6, 128.7, 129.1 (Ar*C*), 136.5 (*ipso*-Ar), 174.3 (NH*C*OCH$_3$), [C-1 and C-2 were not prominent]; LRMS (ESI): *m/z* 411.9 [(M-H)$^-$ 100%]; HRMS (ESI): calcd for $C_{19}H_{26}NO_9$ [M-H]$^-$ 412.1613, found 412.1602.

Methyl [methyl 5-acetamido-4,8,9-tri-*O*-acetyl-3,5-dideoxy-7-*O*-(prop-2-ynyl)-D-*glycero*-β-D-*galacto*-non-2-ulopyranosid]onate (**15**).
A solution of **13d** (1.12 g, 2.02 mmol) in 80% acetic acid was heated at 80 °C for 2 h. The reaction mixture was concentrated under vacuum and co-evaporated with toluene (3 x 10 mL) to remove traces of acid. The crude mixture was dissolved in MeOH:H_2O (1:1) and the temperature was adjusted to 0 °C, followed by addition of aq NaOH (2.0 M) to make pH 13. The reaction mixture was stirred at rt and reaction progress was monitored by TLC (hexane/EtOAc, 1:5). After 16 h, the reaction mixture was acidified to pH 3 with Amberlite® IR-120 (H^+) resin. The resin was filtered-off and the filtrate was evaporated under reduced pressure to afford a yellow syrup which crystallized on drying under high vacuum. The crude product was transferred to a Teflon septum sealed 30 mL pressure tube containing anhydrous MeOH (20 mL) and dry Amberlite® IR-120 (H^+) resin (0.5 g). The mixture was then microwave irradiated (max. power 100 W) for 15 min at 80 °C. After completion of the holding time, the reaction mixture was cooled, filtered and concentrated under reduced pressure to give a yellow residue. The residue was dissolved in pyridine (30 mL) and acetic anhydride (15 mL), and stirred under argon for 16 h at room temperature. The reaction mixture was concentrated to dryness, and the residue was dissolved in EtOAc (50 mL), washed with dil HCl (1 M, 20 mL), water (20 mL), dried (Na_2SO_4), and concentrated. The residue was purified by column chromatography on silica to give **15** (0.8 g,

88% over 4 steps) as a yellow solid. R_f 0.5 (EtOAc); ^{1}H NMR (300 MHz, CDCl$_3$): δ 1.85 (1 H, dd, $J_{3eq,3ax}$ 12.9 Hz, $J_{3ax,4}$ 11.7 Hz, H-3ax), 1.94, 2.00, 2.01, 2.07 (4 x 3 H, 4 x s, 3 x OCOC*H*$_3$ and NHCOC*H*$_3$), 2.39 (1 H, dd, $J_{3eq,3ax}$ 12.9 Hz, $J_{3eq,4}$ 5.1 Hz, H-3eq), 2.43 (1H, t, *J* 2.4 Hz, H-c), 3.23 (1 H, m, OC*H*$_3$), 3.72-3.78 (4 H, m, H-6 and CO$_2$C*H*$_3$), 3.87-3.89 (1 H, m, H-7), 4.15-4.27 (2 H, m, H-5 and H-9A), 4.30 and 4.48 (2 H, dd, *J* 2.4 Hz, J_{gem} 15.6 Hz, H-a), 4.90 (1 H, dd, $J_{9A,9B}$ 12.6 Hz, $J_{9A,8}$ 2.4 Hz, H-9B), 5.16-5.35 (3 H, m, H-8, H-4 and N*H*COCH$_3$); ^{13}C NMR (75.5 MHz, CDCl$_3$): δ 20.8, 20.9 and 21.1 (3 x OCO*C*H$_3$), 23.3 (NHCO*C*H$_3$), 36.8 (C-3), 49.6 (C-5), 51.2 (O*C*H$_3$), 52.6 (CO$_2$*C*H$_3$), 66.0 (C-a), 62.9 (C-9), 69.2 (C-4), 72.5 (C-6), 73.7 (C-8), 75.2 (C-b*), 75.7 (C-7), 79.3 (C-c*), 99.6 (C-2), 167.4 (C-1), 170.3, 170.5, 170.6 and 171.0 (3 x O*C*OCH$_3$, NH*C*OCH$_3$); LRMS (ESI): *m/z* 524.0 [(M+Na)$^+$ 100%]; HRMS (ESI): calcd for $C_{22}H_{31}NNaO_{12}$ [M+Na]$^+$ 524.1738, found 524.1742.

Methyl [methyl 5-acetamido-4,8,9-tri-*O*-acetyl-3,5-dideoxy-7-*O*-(3-phenyl-prop-2-ynyl)-D-*glycero*-β-D-*galacto*-2-nonulopyranosid]onate (**16a**).

To a mixture of **15** (0.1 g, 0.19 mmol), PdCl$_2$(PPh$_3$)$_2$ (2 mg, 0.004 mmol), and CuI (1.5 mg, 0.008 mmol) in CH$_3$CN (5 mL) under argon, were added Et$_3$N (84 µL, 0.59 mmol) and iodobenzene (26 µL, 0.23 mmol). The reaction mixture was stirred at rt and the progress of the reaction was monitored by TLC (EtOAc). After 6 h, the reaction mixture was concentrated under reduced pressure to give a crude residue which was dissolved in a small volume of CH$_2$Cl$_2$, adsorbed onto silica, and purified using a Reveleris® flash chromatography system [40 g column; flow rate 30 mL/min; eluent: 1:1 hexane/EtOAc to 100% EtOAc] to afford **16a** as a white solid (70 mg, 60%). R_f 0.67 (EtOAc); ^{1}H NMR (300 MHz, CDCl$_3$): δ 1.79 (3 H, s, NHCOC*H*$_3$), 1.85 (1 H, m, H-3ax), 2.00, 2.01, 2.03 (3 x 3 H, 3 x s, 3 x OCOC*H*$_3$), 2.39 (1 H, dd, $J_{3eq,3ax}$ 12.9 Hz, $J_{3eq,4}$ 5.1 Hz, H-3eq), 3.24 (3 H, s, OC*H*$_3$), 3.77 (3 H, s, CO$_2$C*H*$_3$), 3.81 (1 H, dd, $J_{6,5}$ 10.5 Hz, $J_{6,7}$ 1.8 Hz, H-6), 4.03-4.05 (1 H, m, H-7), 4.23 (1 H, app.q, *J* 10.5 Hz, H-5), 4.33 (1 H, dd, $J_{9A,9B}$ 12.6Hz, $J_{9A,8}$ 7.8 Hz, H-9A), 4.53 and 4.72 (2 H, dd, J_{gem} 15.9 Hz, H-a), 4.90 (1 H, dd, $J_{9B,9A}$ 12.6Hz, $J_{9B,8}$ 2.4 Hz, H-9B), 5.18-5.35 (3 H, m, H-8, H-4 and N*H*COCH$_3$), 7.26-7.30 (3 H, m, Ar*H*), 7.40-7.43 (2 H, m, Ar*H*); ^{13}C NMR (75.5 MHz, CDCl$_3$): δ 20.8, 20.9 and 21.1 (3 x OCO*C*H$_3$), 23.2 (NHCO*C*H$_3$), 36.9 (C-3), 49.8 (C-5), 51.2 (O*C*H$_3$), 52.6 (CO$_2$*C*H$_3$), 60.7 (C-a), 63.0 (C-9), 69.2 (C-4), 72.6 (C-6), 73.9 (C-8), 75.2 (C-7), 84.7 (C-b*), 87.2 (C-c*), 98.6 (C-2), 122.4 (*ipso*-Ar), 128.3, 128.5, and 131.7 (5 x Ar*C*), 167.4, 170.2, 170.6, 170.6 and 171.0 (C-1, 3 x O*C*OCH$_3$, NH*C*OCH$_3$); LRMS (ESI): *m/z* 600.0 [(M+Na)$^+$ 100%]; HRMS (ESI): calcd for $C_{28}H_{35}NNaO_{12}$ [M+Na]$^+$ 600.2051, found 600.2065.

Methyl {methyl 5-acetamido-4,8,9-tri-*O*-acetyl-3,5-dideoxy-7-*O*-[3-(4-methoxyphenyl)-prop-2-ynyl]-D-*glycero*-β-D-*galacto*-2-nonulopyranosid}onate (**16b**).

Compound **16b** was prepared by coupling **15** (0.1 g, 0.19 mmol) with 4-iodoanisole (0.05 g, 0.23 mmol) over 2 h, in a similar manner to that described for the synthesis of **16a** from **15**. The crude reaction product was purified using a Reveleris® flash chromatography system [40 g column; flow rate 30 mL/min; eluent: 1:1 hexane/EtOAc to 100% EtOAc] to afford **16b** as a white solid (105 mg, 87%). R_f 0.75 (EtOAc); ^{1}H NMR (300 MHz, CDCl$_3$): δ 1.78 (3 H, s, NHCOC*H*$_3$), 1.85 (1 H, m, H-3ax), 2.00, 2.01, 2.03 (3 x 3 H, 3 x s, 3 x OCOC*H*$_3$),2.39 (1 H, dd, $J_{3eq,3ax}$ 12.9 Hz, $J_{3eq,4}$ 4.8 Hz, H-3eq), 3.24 and 3.76 (2 x 3 H, 2 x s, 2 x OC*H*$_3$), 3.78 (3 H, s, CO$_2$C*H*$_3$), 3.82 (1 H, dd, $J_{6,5}$ 10.5 Hz, $J_{6,7}$ 1.8 Hz, H-6), 4.03-4.06 (1 H, m, H-7), 4.21 (1 H, app.q, *J* 10.5 Hz, H-5), 4.32 (1 H, dd, $J_{9A,9B}$ 12.6Hz, $J_{9A,8}$ 8.1 Hz, H-9A), 4.51 and 4.68 (2 H, dd, J_{gem} 15.9 Hz, H-a), 4.93 (1H, dd, $J_{9B,9A}$ 12.6Hz, $J_{9B,8}$ 2.4 Hz, H-9B), 5.18-5.35 (3 H, m, H-8, H-4 and N*H*COCH$_3$), 6.80 (2 H, d, *J* 9.0 Hz, Ar*H*), 7.35 (2 H, d, *J* 9.0 Hz, Ar*H*); ^{13}C NMR (75.5 MHz, CDCl$_3$): δ 20.8, 20.9 and 21.1 (3 x OCO*C*H$_3$), 23.2 (NHCO*C*H$_3$), 36.9 (C-3), 49.8 (C-5), 51.1 and 52.6 (O*C*H$_3$), 55.2 (CO$_2$CH$_3$), 60.7 (C-a), 63.0 (C-9), 69.2 (C-4), 72.5 (C-6), 73.9 (C-8), 75.0 (C-7), 83.2 (C-b*), 87.1 (C-c*), 98.6 (C-2), 113.9 (2 x Ar*C*), 114.5 (Ar*C*$_q$), 133.2 (2 x Ar*C*), 159.7 (Ar*C*$_q$), 167.4 (C-1), 170.2, 170.6, 170.6 and 171.0 (3 x O*C*OCH$_3$, NH*C*OCH$_3$); LRMS (ESI): *m/z* 630.2 [(M+Na)$^+$ 100%]; HRMS (ESI): calcd for $C_{29}H_{37}NNaO_{13}$ [M+Na]$^+$ 630.2157, found 630.2166.

Methyl {methyl 5-acetamido-4,8,9-tri-*O*-acetyl-3,5-dideoxy-7-*O*-[3-(2-thiofuranyl)-prop-2-ynyl]-D-*glycero*-β-D-*galacto*-2-nonulopyranosid}onate (**16c**).

Compound **16c** was prepared by coupling **15** (0.1 g, 0.19 mmol) with 2-iodothiophene (26 µL, 0.23 mmol) over 2 h, in a similar manner to that described for the synthesis of **16a** from **15**. The crude reaction product was purified using a Reveleris® flash chromatography system [40 g column; flow rate 30 mL/min; eluent: 1:1 hexane/EtOAc to 100% EtOAc] to afford **16c** as a white solid (99 mg, 85%). R_f 0.62 (EtOAc); ^{1}H NMR (300 MHz, CDCl$_3$): δ 1.83 (3 H, s, NHCOC*H*$_3$), 1.85 (1 H, m, H-3ax), 2.00, 2.01, 2.05 (3 x 3 H, 3 x s, 3 x OCOC*H*$_3$), 2.40 (1 H, dd, $J_{3eq,3ax}$ 12.9 Hz, $J_{3eq,4}$ 5.1 Hz, H-3eq), 3.24 (3 H, s, OC*H*$_3$), 3.77 (3 H, s, CO$_2$C*H*$_3$), 3.81 (1 H, dd, $J_{6,5}$ 10.5 Hz, $J_{6,7}$ 1.8 Hz, H-6), 3.98-4.00 (1 H, m, H-7), 4.23 (1 H, app.q, *J* 10.5 Hz, H-5), 4.30 (1 H, dd, $J_{9A,9B}$ 12.6Hz, $J_{9A,8}$ 7.8 Hz, H-9A), 4.53 and 4.73 (2 H, dd, J_{gem} 16.2 Hz, H-a), 4.93 (1 H, dd, $J_{9B,9A}$ 12.6Hz, $J_{9B,8}$ 2.4 Hz, H-9B), 5.18-5.32 (2 H, m, H-8 and H-4), 5.34 (1 H, d, $J_{NH,5}$ 10.5 Hz, N*H*COCH$_3$), 6.94 (1 H, dd, *J* 3.9 Hz, *J* 5.1 Hz, Ar*H*), 7.20-7.23 (2 H, m, Ar*H*); ^{13}C NMR (75.5 MHz, CDCl$_3$): δ 20.8, 20.9 and 21.1 (3 x OCO*C*H$_3$), 23.3 (NHCO*C*H$_3$), 36.9 (C-3), 49.9 (C-5), 51.2 (O*C*H$_3$), 52.6 (CO$_2$*C*H$_3$), 60.7 (C-a), 62.9 (C-9), 69.2 (C-4), 72.6 (C-6), 73.8 (C-8), 75.4 (C-7), 80.4 (C-b*), 88.8 (C-c*), 98.6 (C-

2), 122.3 (ArC_q), 127.0, 127.5 and 132.6 (3 x Ar*C*), 167.4, 170.2, 170.5, 170.6 and 171.0 (3 x O*C*OCH_3, NH*C*OCH_3, C-1); LRMS (ESI): *m/z* 606.1 [$(M+Na)^+$ 100%]; HRMS (ESI): calcd for $C_{26}H_{33}NNaO_{12}S$ $[M+Na]^+$ 606.1615, found 606.1610.

Methyl 5-acetamido-3,5-dideoxy-7-*O*-(3-phenyl-prop-2-ynyl)-D-*glycero*-β-D-*galacto*-2-nonulopyranosidonic acid, sodium salt (**17a**).

Prepared from **16a** (0.07 g, 0.12 mmol), according to the general procedure for de-esterification (52 mg, 90% yield). HPLC: 0.5:99.5 CH_3CN/H_2O; retention time 8.0-13.5 min; ^1H NMR (300 MHz, D_2O): δ 1.65-1.74 (1 H, m, H-3ax), 2.02 (3 H, s, NHCOCH_3), 2.34 (1 H, dd, $J_{3eq,3ax}$ 12.9 Hz, $J_{3eq,4}$ 4.5 Hz, H-3eq), 3.24 (3 H, s, OCH_3), 3.74-3.80 (2 H, m, H-7 and H-9A), 3.86-4.06 (5 H, m, H-4, H-5, H-6, H-8 and H-9B), 4.57 (2 H, d, *J* 2.4 Hz, H-a), 7.40-7.48 (3 H, m, Ar*H*), 7.54-7.59 (2H, m, Ar*H*); ^{13}C NMR (75.5 MHz, D_2O): δ 22.2 (NHCO*C*H_3), 39.6 (C-3), 50.5 (O*C*H_3), 51.9 (C-5), 60.3 (C-a), 62.8 (C-9), 67.7 (C-4), 70.1 (C-6*), 70.2 (C-8*), 76.1 (C-7), 84.0 (C-b*), 87.2 (C-c*), 100.5 (C-2), 121.6 (*ipso*-Ar*C*), 128.6, 129.1 and 131.7 (5 x Ar*C*), 174.2, 175.0 (C-1, NH*C*OCH_3); LRMS (ESI): *m/z* 435.9 [$(M-Na)^-$ 100%].

Methyl 5-acetamido-3,5-dideoxy-7-*O*-[3-(4-methoxyphenyl)-prop-2-ynyl]-D-*glycero*-β-D-*galacto*-2-nonulopyranosidonic acid, sodium salt (**17b**).

Prepared from 1**6b** (0.1 g, 0.17 mmol), according to the general procedure for de-esterification (77 mg, 95% yield). HPLC: 10:90 CH_3CN/H_2O; retention time 10.5-14.5 min; ^1H NMR (300 MHz, D_2O): δ 1.65-1.73 (1 H, m, H-3ax), 2.02 (3 H, s, NHCOCH_3), 2.34 (1 H, dd, $J_{3eq,\,3ax}$ 13.5 Hz, $J_{3eq,\,4}$ 4.2 Hz, H-3eq), 3.22 (3 H, s, OCH_3), 3.72-3.79 (2 H, m, H-7 and H-9A), 3.85 (3 H, s, OCH_3), 3.90-4.70 (5 H, m, H-4, H-5, H-6, H-8 and H-9B), 4.55 (2 H, d, *J* 2.4 Hz, H-a), 7.00 (2 H, d, *J* 8.7 Hz, Ar*H*), 7.52 (2 H, d, *J* 8.7 Hz, Ar*H*); ^{13}C NMR (75.5 MHz, D_2O): δ 22.2 (NHCO*C*H_3), 39.6 (C-3), 50.5 (O*C*H_3), 51.9 (C-5), 55.3 (O*C*H_3), 60.4 (C-a), 62.9 (C-9), 67.7 (C-4*), 70.2 (C-6*), 70.2 (C-8*), 76.0 (C-7), 82.8 (C-b*), 87.1 (C-c*), 100.5 (C-2), 114.0 (ArC_q), 114.2, 133.4 (4 x Ar*C*), 159.4 (ArC_q), 174.2, 175.0 (C-1, NH*C*OCH_3); LRMS (ESI): *m/z* 465.9 [$(M-Na)^-$ 100%].

Methyl 5-acetamido-3,5-dideoxy-7-*O*-[3-(2-thiofuranyl)-prop-2-ynyl]-D-*glycero*-β-D-*galacto*-2-nonulopyranosidonic acid, sodium salt (**17c**).

Prepared from **16c** (0.1 g, 0.18 mmol), according to the general procedure for de-esterification (70 mg, 92% yield). HPLC: 3.3:96.7 CH_3CN/H_2O; retention time 7.5-12.5 min; ^1H NMR (300 MHz, D_2O): δ 1.65-1.73 (1 H, m, H-3ax), 2.04 (3 H, s, NHCOCH_3), 2.34 (1 H, dd, $J_{3eq,3ax}$ 13.2 Hz, $J_{3eq,4}$ 4.2 Hz, H-3eq), 3.21 (3 H, s, OCH_3), 3.72-3.78 (2 H, m, H-7 and H-9A), 3.89-4.05 (5 H, m, H-4, H-5, H-6, H-8 and H-9B), 4.59 (2 H, d, *J* 1.8 Hz, H-a), 7.09 (1 H, app.q, *J* 3.9 Hz, H-b'), 7.39 (1 H, dd, *J* 3.9 Hz, *J* 1.2 Hz, H-a'), 7.50 (1 H, dd, *J* 5.1 Hz, *J* 1.2 Hz, H-c'); ^{13}C NMR (75.5 MHz, D_2O): δ 22.2 (NHCO*C*H_3), 39.6 (C-3), 50.5 (O*C*H_3), 51.9 (C-5), 60.3 (C-a), 62.8 (C-9), 67.7 (C-4*), 70.1 (C-6*), 70.2 (C-8*), 76.1 (C-7), 80.5 (C-b*), 87.8 (C-c*), 100.5 (C-2), 121.2 (*ipso*-Ar), 127.3, 128.6, 133.4 (Ar*C*), 174.2, 175.0 (C-1, NH*C*OCH_3); LRMS (ESI): *m/z* 441.8 [$(M-Na)^-$ 100%].

Synthesis of C-5 amide derivatives of Neuβ2Me (3)

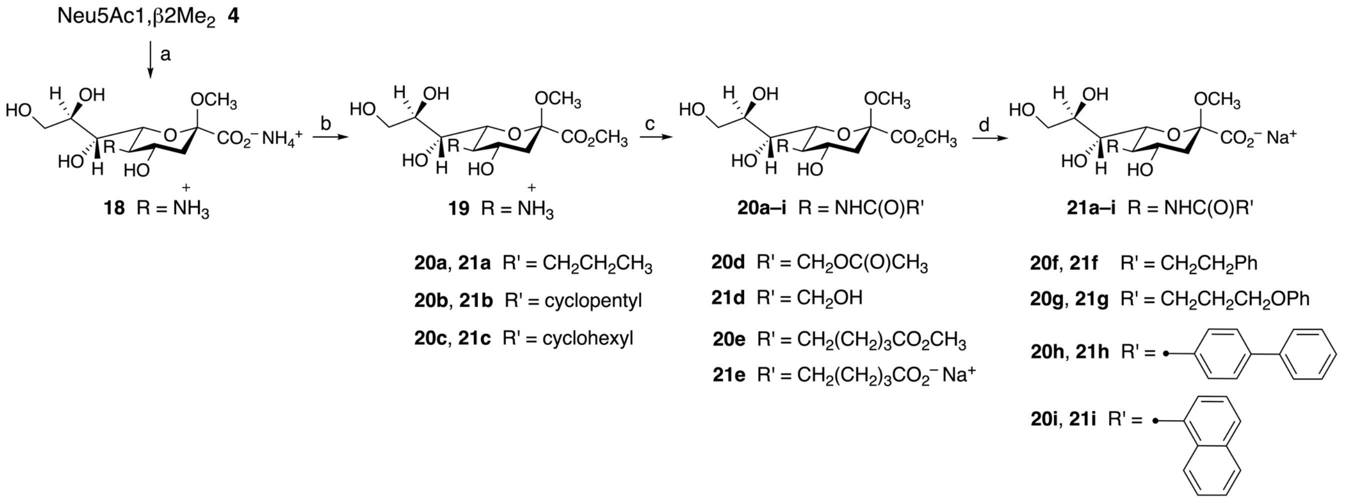

Scheme S3. Synthesis of C-5 amide derivatives of Neuβ2Me. *Reagents and conditions:* a) NaOH (2.0 M), MW (100W), 120 °C, 15 min (80%); b) $SOCl_2$, MeOH, 0 °C – rt, 16 h (92%); c) R'COCl, Et_3N, 1,4-dioxane/water (5:1), 0 °C – 40 °C, 2 h (**20a** 87%, **20b** 90%, **20c** 91%, **20d** 91%, **20e** 78%, **20f** 80%, **20g** 82%, **20h** 80%, **20i** 88%); d) NaOH, MeOH/H_2O (1:1), pH 13, 0 °C – rt, 16 h. In **20**,**21h,i**, the sphere indicates the point of attachment of R' to the amide carbon.

Methyl 5-amino-3,5-dideoxy-D-*glycero*-β-D-*galacto*-non-2-ulopyranosidonic acid, ammonium salt (18).

In a Teflon septum sealed 30 mL pressure tube, a mixture of **4** (1.0 g, 2.97 mmol) and 2.0 M NaOH (15 mL) was microwave irradiated (max. power 100 W) for 15 min at 120 °C. After completion of the holding time, the reaction mixture was cooled and concentrated under reduced pressure to give a yellow residue. The residue was taken up in water (~1 mL) and added to a column of Dowex® 50Wx8 (H^+) resin (20.0 g). The column was first eluted with water (200 mL) and then with 1.5 M ammonium hydroxide (100 mL). Fractions containing **18** [15] were combined, concentrated under reduced pressure and lyophilized to afford a pale-yellow fluffy solid (0.45 g, 80% yield). R_f 0.30 (*i*PrOH/H_2O/AcOH 15:4:0.5); ^{1}H NMR (300 MHz, D_2O): δ 1.58 (1 H, dd, $J_{3ax,3eq}$ 12.9 Hz, $J_{3ax,4}$ 11.4 Hz, H-3_{ax}), 2.26 (1 H, dd, $J_{3eq,3ax}$ 12.9 Hz, $J_{3eq,4}$ 5.1 Hz, H-3_{eq}), 2.79 (1 H, app.t, $J_{5,4}$ ≈ $J_{5,6}$ ≈ $J_{5,NH}$ 9.9Hz, H-5), 3.20 (3 H, s, OCH_3), 3.68 (1 H, dd, $J_{6,5}$ 9.9Hz, $J_{6,7}$ 1.5Hz, H-6), 3.61-3.95 (5 H, m, H-4, H-7, H-8, H-9A, H-9B); ^{13}C NMR (75.5 MHz, D_2O): δ 40.2 (C-3), 50.4 (OCH_3), 52.9 (C-5), 63.8 (C-9), 68.7 (C-4*), 68.9 (C-7*), 70.2 (C-8*), 72.4 (C-6), 100.7 (C-2), 175.9 (C-1); LRMS (ESI): *m/z* 280.0 [$(M-NH_4)^-$, 100%].

Methyl (methyl 5-amino-3,5-dideoxy-D-*glycero*-β-D-*galacto*-non-2-ulopyranosid)onate (19).

To a stirring solution of **18** (1.9 g, 6.76 mmol) in anhydrous MeOH (100 mL) at 0 °C under argon, $SOCl_2$ (0.73 mL, 10.14 mmol) was added drop wise. The reaction mixture was initially stirred at 0 °C and then warmed to rt. The progress of reaction was monitored by TLC (*i*PrOH/H_2O/AcOH 15:4:0.5). After 16 h, the reaction mixture was concentrated under reduced pressure to give a brown syrup, which was purified by flash chromatography (*i*PrOH/H_2O/AcOH 15:4:0.5) to furnish **19** (1.84 g, 92%) as a yellow solid. R_f 0.71 (*i*PrOH/H_2O/AcOH 15:4:0.5); ^{1}H NMR (300 MHz, CD_3OD): δ 1.65 (1 H, dd, $J_{3ax,3eq}$ 12.9 Hz, $J_{3ax,4}$ 11.1 Hz, H-3_{ax}), 2.34 (1 H, dd, $J_{3eq,3ax}$ 12.9 Hz, $J_{3eq,4}$ 4.8 Hz, H-3_{eq}), 3.16-3.23 (4 H, m, OCH_3 and H-5), 3.68-3.84 (7 H, m, H-6, H-8, H-9A, H-9B and CO_2CH_3), 4.01-4.13 (2 H, m, H-4 and H-7); ^{13}C NMR (75.5 MHz, CD_3OD): δ 41.4 (C-3), 49.9 (OCH_3), 52.1 (CO_2CH_3), 53.7 (C-5), 64.8 (C-9), 66.1 (C-4), 69.5 (C-6*), 70.6 (C-7), 71.6 (C-8*), 100.4 (C-2), 170.7 (C-1); LRMS (ESI): *m/z* 296.0 [$(M+H)^+$ 100%]; HRMS (ESI): calcd for $C_{11}H_{22}NO_8$ $[M+H]^+$ 296.1340, found 296.1335.

Methyl (methyl 5-butanamido-3,5-dideoxy-D-*glycero*-β-D-*galacto*-non-2-ulopyranosid)onate (20a).

A solution of compound **19** (0.1 g, 0.33 mmol) in water (1 mL) was diluted with dioxane (5 mL) followed by the addition of Et_3N (0.5 mL). The reaction mixture was then cooled to 0 °C and butyryl chloride (0.14 mL, 1.35 mmol) in dioxane (0.5 mL) was added drop wise to the solution of **19** with stirring. The reaction temperature was first raised to rt and then to 40 °C, at which temperature reaction was continued for 2 h. After completion of the reaction, the mixture was cooled and concentrated under reduced pressure to give a yellow residue. The residue was purified by flash chromatography (EtOAc EtOAc/MeOH 5:1) to furnish **20a** (104 mg, 87%) as a yellow solid. R_f 0.45 (EtOAc/MeOH 5:1); ^{1}H NMR (300 MHz, CD_3OD): δ 0.95 (3 H, t, *J* 7.5 Hz, H-c), 1.59-1.69 (3 H, m, H-b and H-3ax), 2.22 (2 H, t, *J* 7.5 Hz, H-a), 2.34 (1 H, dd, $J_{3eq,3ax}$ 12.9 Hz, $J_{3eq,4}$ 4.8 Hz, H-3_{eq}), 3.26 (3 H, s, OCH_3), 3.49 (1 H, d, *J* 9.0 Hz, H-7), 3.61 (1 H, dd, $J_{9A,9B}$ 12.3 Hz, $J_{9A,8}$ 6.3 Hz, H-9A), 3.78-3.83 (7 H, m, H-5, H-6, H-8, H-9B and CO_2CH_3), 3.95-4.01 (1 H, m, H-4); ^{13}C NMR (75.5 MHz, CD_3OD): δ 14.1 (C-c), 20.3 (C-b), 39.1 (C-3), 41.7 (C-a), 51.7 (OCH_3), 53.2 (CO_2CH_3), 53.6 (C-5), 65.3 (C-9), 67.6 (C-4), 70.1 (C-7), 71.4 (C-6*), 72.4 (C-8*), 100.4 (C-2), 171.0 (C-1), 177.7 (*C*ONH); LRMS: *m/z* 388.1 [$(M+Na)^+$ 100%]; HRMS (ESI): calcd for $C_{15}H_{27}NNaO_{12}$ $[M+Na]^+$ 388.1578, found 388.1576.

Methyl (methyl 5-cyclopentylcarboxamido-3,5-dideoxy-D-*glycero*-β-D-*galacto*-non-2-ulopyranosid)onate (20b).

Compound **20b** was prepared by coupling **19** (0.1 g, 0.33 mmol) with cyclopentanecarbonyl chloride (0.16 mL, 1.35 mmol) in a similar manner to that described for the synthesis of **20a** from **19**. The crude reaction product was purified by flash chromatography (hexane/EtOAc, 1:1 EtOAc) to give **20b** (120 mg, 90%) as a white solid. R_f 0.51 (EtOAc/MeOH 5:1); ^{1}H NMR (300 MHz, CD_3OD): δ 1.58-1.86 (11 H, m, cyclopentyl-CH_2 and H-3_{ax}), 2.33 (1 H, dd, $J_{3eq,3ax}$ 12.9 Hz, $J_{3eq,4}$ 5.1 Hz, H-3_{eq}), 2.63-2.71 (1 H, m, H-a), 3.26 (3 H, s, OCH_3), 3.47 (1 H, d, *J* 9.3 Hz, H-7), 3.61 (1 H, dd, $J_{9A,9B}$ 10.8 Hz, $J_{9A,8}$ 4.8 Hz, H-9A), 3.78-3.83 (7 H, m, H-5, H-6, H-8, H-9B and CO_2CH_3), 3.97-4.05 (1 H, m, H-4); ^{13}C NMR (75.5 MHz, CD_3OD): δ 27.0 (C-c), 27.0 (C-c'), 31.2 (C-b), 31.8 (C-b'), 41.8 (C-3), 46.6 (C-a), 51.6 (OCH_3), 53.1 (CO_2CH_3), 53.7 (C-5), 65.4 (C-9), 67.6 (C-4), 70.2 (C-7), 71.3 (C-6*), 72.4 (C-8*), 100.4 (C-2), 170.8 (C-1), 181.0 (*C*ONH); LRMS (ESI): *m/z* 414.2 [$(M+Na)^+$ 100%]; HRMS (ESI): calcd for $C_{17}H_{29}NNaO_9$ $[M+Na]^+$ 414.1735, found 414.1740.

Methyl (methyl 5-cyclohexylcarboxamido-3,5-dideoxy-D-*glycero*-β-D-*galacto*-non-2-ulopyranosid)onate (20c).

Compound **20c** was prepared by coupling **19** (0.1 g, 0.33 mmol) with cyclohexanecarbonyl chloride (0.18 mL, 1.35 mmol) in a similar manner to that described for the synthesis of **20a** from **19**. The crude reaction product was purified by flash chromatography (hexane/EtOAc, 1:1 EtOAc) to give **20c** (125 mg, 91%) as a white solid. R_f 0.51 (EtOAc/MeOH 5:1); ^{1}H NMR (300 MHz, CD_3OD): δ 1.20-1.51 (6 H, m, H-b, H-b' and H-d), 1.63 (1 H, dd, $J_{3ax,3eq}$ 12.9 Hz, $J_{3ax,4}$ 11.1 Hz, H-3_{ax}), 1.76-1.84 (4 H, m, H-c and H-c'), 2.18-2.27 (1 H, m, H-a), 2.33 (1 H, dd, $J_{3eq,3ax}$ 12.9 Hz, $J_{3eq,4}$ 4.8 Hz, H-3_{eq}), 3.26 (3 H, s, OCH_3), 3.44 (1 H,

d, *J* 10.2 Hz, H-7), 3.61 (1 H, dd, $J_{9A,9B}$ 11.4 Hz, $J_{9A,8}$ 5.7 Hz, H-9A), 3.78-3.84 (7 H, m, H-5, H-6, H-8, H-9B and CO_2CH_3), 3.96-4.05 (1 H, m, H-4); ^{13}C NMR (75.5 MHz, CD_3OD): δ 26.6, 26.8, 26.9, 30.4, 31.0 (cyclohexyl-CH_2), 41.8 (C-3), 46.6 (C-a), 51.6 (OCH_3), 53.1 (CO_2CH_3), 53.5 (C-5), 65.4 (C-9), 67.5 (C-4), 70.2 (C-7), 71.3 (C-6*), 72.4 (C-8*), 100.4 (C-2), 170.7 (C-1), 180.9 (*C*ONH); LRMS (ESI): *m/z* 428.2 [$(M+Na)^+$ 100%]; HRMS (ESI): calcd for $C_{18}H_{31}NNaO_9$ $[M+Na]^+$ 428.1891, found 428.1893.

Methyl (methyl 5-acetoxyacetamido-3,5-dideoxy-D-*glycero*-β-D-*galacto*-non-2-ulopyranosid)onate (20d).
Compound **20d** was prepared by coupling **19** (0.1 g, 0.33 mmol) with acetyloxyacetyl chloride (0.14 mL, 1.35 mmol) in a similar manner to that described for the synthesis of **20a** from **19**. The crude reaction product was purified by flash chromatography (hexane/EtOAc, 1:1 EtOAc) to give **20d** (122 mg, 91%) as a white solid. An inseparable impurity of acetoxyglycolic acid was present in the isolated product. R_f 0.51 (EtOAc/MeOH 5:1);^{1}H NMR (300 MHz, CD_3OD): δ 1.64 (1 H, dd, $J_{3ax,3eq}$ 12.9 Hz, $J_{3ax,4}$ 11.1 Hz, H-3_{ax}), 2.13 (3 H, s, $OCOCH_3$), 2.34 (1 H, dd, $J_{3eq,3ax}$ 12.9 Hz, $J_{3eq,4}$ 5.1 Hz, H-3_{eq}), 3.26 (3 H, s, OCH_3), 3.51 (1 H, d, *J* 8.7 Hz, H-7), 3.64 (1 H, dd, $J_{9A,9B}$ 12.3 Hz, $J_{9A,8}$ 6.3 Hz, H-9A), 3.77-3.84 (5 H, m, H-8, H-9B and CO_2CH_3), 3.90-3.94 (2 H, m, H-5 and H-6), 4.03-4.12 (1 H, m, H-4), 4.58 (2 H, s, $COCH_2$); ^{13}C NMR (75.5 MHz, CD_3OD): δ 20.6 ($OCOCH_3$), 41.4 (C-3), 51.7 (OCH_3), 53.4 (CO_2CH_3 and C-5), 63.6 ($COCH_2$), 65.2 (C-9), 67.3 (C-4), 69.9 (C-7), 71.6 (C-8), 71.9 (C-6), 100.5 (C-2), 171.4, 172.1, 172.4 (C-1, $OCOCH_3$, NH*C*O), ; LRMS (ESI): *m/z* 418.1 [$(M+Na)^+$ 100%].

Methyl [methyl 3,5-dideoxy-5-(5-methoxycarbonyl-pentanamido)-D-*glycero*-β-D-*galacto*-non-2-ulopyranosid]onate (20e).
Compound **20e** was prepared by coupling **19** (0.1 g, 0.33 mmol) with methyl adipoyl chloride (0.23 mL, 1.35 mmol) in a similar manner to that described for the synthesis of **20a** from **19**. The crude reaction product was purified by flash chromatography (hexane/EtOAc, 1:1 EtOAc) to give **20e** (115 mg, 78%) as a white solid. R_f 0.42 (EtOAc/MeOH 5:1); ^{1}H NMR (300 MHz, CD_3OD): δ 1.59-1.66 (5 H, m, H-3ax, H-b and H-c), 2.24-2.36 (5H, m, H-3_{eq}, H-a and H-d), 3.26 (3 H, s, OCH_3), 3.48 (1 H, d, *J* 9.0 Hz, H-7), 3.60-3.66 (4 H, m, CO_2CH_3 and H-9A), 3.78-3.83 (7 H, m, H-5, H-6, H-8, H-9B and CO_2CH_3), 3.95-4.00 (1 H, m, H-4); ^{13}C NMR (75.5 MHz, CD_3OD): δ 25.5 (C-b), 26.3 (C-c), 34.4 (C-a), 36.7 (C-d), 41.7 (C-3), 51.6 (OCH_3), 52.1 (C-5), 53.1 and 53.6 (2 x CO_2CH_3), 65.3 (C-9), 67.6 (C-4), 70.2 (C-7), 71.4 (C-6*), 72.3 (C-8*), 100.4 (C-2), 170.8 (C-1), 175.8 (*C*O_2CH_3), 177.3 (*C*ONH); LRMS (ESI): *m/z* 460.2 [$(M+Na)^+$ 100%]; HRMS (ESI): calcd for $C_{18}H_{31}NNaO_{11}$ $[M+Na]^+$ 460.1789, found 460.1789.

Methyl [methyl 3,5-dideoxy-5-(3-phenyl-propanamido)-D-*glycero*-β-D-*galacto*-non-2-ulopyranosid]onate (20f).
Compound **20f** was prepared by coupling **19** (0.1 g, 0.33 mmol) with hydrocinnamoyl chloride (0.20 mL, 1.35 mmol) in a similar manner to that described for the synthesis of **20a** from **19**. The crude reaction product was purified by flash chromatography (hexane/EtOAc, 1:1 EtOAc) to give **20f** (118 mg, 80%) as a white solid. R_f 0.69 (EtOAc/MeOH 5:1); ^{1}H NMR (300 MHz, CD_3OD): δ 1.62 (1 H, dd, $J_{3ax,3eq}$ 12.9 Hz, $J_{3ax,4}$ 11.1 Hz, H-3_{ax}), 2.33 (1 H, dd, $J_{3eq,3ax}$ 12.9 Hz, $J_{3eq,4}$ 4.8 Hz, H-3_{eq}), 2.54 and 2.92 (2 x 2 H, 2 x app.t, *J* 8.7 Hz, H-a and H-b), 3.25-3.38 (4 H, m, OCH_3 and H-7), 3.58 (1 H, dd, $J_{9A,9B}$ 12.0 Hz, $J_{9A,8}$ 6.0 Hz, H-9A), 3.78-3.87 (7 H, m, H-5, H-6, H-8, H-9B and CO_2CH_3), 3.93-4.02 (1 H, m, H-4), 7.15-7.28 (5 H, m, Ar-*H*); ^{13}C NMR (75.5 MHz, CD_3OD): δ 32.6 (C-a), 38.9 (C-b), 41.4 (C-3), 52.0 (OCH_3), 53.5 (CO_2CH_3), 54.0 (C-5), 65.3 (C-9), 67.6 (C-4), 69.9 (C-7), 71.3 (C-6*), 72.1 (C-8*), 100.5 (C-2), 127.4, 129.5, and 129.7 (5 x Ar*C*) and 142.1 (ArC_q), 171.6 (C-1), 177.4 (*C*ONH); LRMS (ESI): *m/z* 450.2 [$(M+Na)^+$ 100%]; HRMS (ESI): calcd for $C_{20}H_{29}NNaO_9$ $[M+Na]^+$ 450.1735, found 450.1734.

Methyl [methyl 3,5-dideoxy-5-(4-phenoxy-butanamido)-D-*glycero*-β-D-*galacto*-non-2-ulopyranosid]onate (20g).
Compound **20g** was prepared by coupling **19** (0.1 g, 0.33 mmol) with 4-(phenoxy)butyryl chloride (0.23 mL, 1.35 mmol) in a similar manner to that described for the synthesis of **20a** from **19**. The crude reaction product was purified by flash chromatography (hexane/EtOAc, 1:1 EtOAc) to give **20g** (130 mg, 82%) as a white solid. R_f 0.66 (EtOAc/MeOH 5:1); ^{1}H NMR (300 MHz, CD_3OD): δ 1.63 (1 H, dd, $J_{3ax,3eq}$ 13.2 Hz, $J_{3ax,4}$ 11.4 Hz, H-3_{ax}), 2.03-2.13 (2 H, m, H-b), 2.33 (1 H, dd, $J_{3eq,3ax}$ 13.2 Hz, $J_{3eq,4}$ 4.8 Hz, H-3_{eq}), 2.45 (2 H, app.t, *J* 7.8 Hz, H-c), 3.26 (3 H, s, OCH_3), 3.47-3.63 (2 H, m, H-7 and H-9A), 3.77-3.86 (7 H, m, H-5, H-6, H-8, H-9B and CO_2CH_3), 3.98-4.02 (3 H, m, H-4 and H-a), 6.88-6.91 (3 H, m, Ar-*H*), 7.21-7.26 (2 H, m, Ar-*H*); ^{13}C NMR (75.5 MHz, CD_3OD): δ 26.5 (C-b), 33.7 (C-c), 41.4 (C-3), 52.0 (OCH_3), 53.5 (CO_2CH_3), 54.1 (C-5), 65.1 (C-9), 67.7 (C-4), 68.4 (C-a), 69.9 (C-7), 71.4 (C-6*), 72.1 (C-8*), 100.6 (C-2), 115.8, 122.2, 130.8 (5 x Ar*C*), 160.0 (*ipso*-Ar*C*), 171.7 (C-1), 177.6 (*C*ONH); LRMS (ESI): *m/z* 480.2 [$(M+Na)^+$ 100%]; HRMS (ESI): calcd for $C_{21}H_{31}NNaO_{10}$ $[M+Na]^+$ 480.1840, found 480.1839.

Methyl [methyl 3,5-dideoxy-5-(4-phenyl-benzamido)-D-*glycero*-β-D-*galacto*-non-2-ulopyranosid]onate (20h).
Compound **20h** was prepared by coupling **19** (0.1 g, 0.33 mmol) with biphenyl-4-carbonyl chloride (0.29 g, 1.35 mmol) in a similar manner to that described for the synthesis of **20a** from **19**. The crude reaction product was purified by flash

chromatography (hexane/EtOAc, 1:1 EtOAc) to give **20h** (130 mg, 80%) as a white solid. R_f 0.63 (EtOAc/MeOH 5:1); ^{1}H NMR (300 MHz, CD_3OD): δ 1.63 (1 H, dd, $J_{3ax,3eq}$ 12.9 Hz, $J_{3ax,4}$ 11.1 Hz, H-3_{ax}), 2.40 (1 H, dd, $J_{3eq,3ax}$ 12.9 Hz, $J_{3eq,4}$ 4.8 Hz, H-3_{eq}), 3.26 (3 H, s, OCH_3), 3.57-3.67 (2 H, m, H-7 and H-9A), 3.78-3.67 (5 H, m, H-8, H-9B and CO_2CH_3), 4.01 (1 H, d, *J* 10.2 Hz, H-6), 4.12 (1 H, app.t, *J* 10.2 Hz, H-5), 4.18-4.27 (1 H, m, H-4), 7.34-7.47 (3 H, m, Ar-*H*), 7.64-7.72 (4 H, m, Ar-*H*), 7.96 (2 H, d, *J* 8.7 Hz, Ar-*H*); ^{13}C NMR (75.5 MHz, CD_3OD): δ 41.5 (C-3), 52.1 (OCH_3), 54.2 (CO_2CH_3 and C-5), 65.0 (C-9), 67.6 (C-4), 70.0 (C-7), 71.4 (C-6*), 72.1 (C-8*), 100.7 (C-2), 128.2, 129.3, 129.4, 130.3 (9 x Ar*C*), 133.7, 141.0 and 145.9 (3 x ArC_q), 171.8 (C-1), 171.9 (*C*ONH); LRMS (ESI): *m/z* 498.2 [$(M+Na)^+$ 100%]; HRMS (ESI): calcd for $C_{24}H_{29}NNaO_9$ $[M+Na]^+$ 498.1735, found 498.1730.

Methyl [methyl 3,5-dideoxy-5-(1-naphthylcarboxamido)-D-*glycero*-β-D-*galacto*-non-2-ulopyranosid]onate (20i).
Compound **20i** was prepared by coupling **19** (0.1 g, 0.33 mmol) with 1-naphthoyl chloride (0.20 mL, 1.35 mmol) in a similar manner to that described for the synthesis of **20a** from **19**. The crude reaction product was purified by flash chromatography (hexane/EtOAc, 1:1 EtOAc) to give **20i** (133 mg, 88%) as a white solid. R_f 0.73 (EtOAc/MeOH 5:1); ^{1}H NMR (300 MHz, CD_3OD): δ 1.74 (1H, dd, $J_{3ax,3eq}$ 12.9 Hz, $J_{3ax,4}$ 10.8 Hz, H-3_{ax}), 2.41 (1 H, dd, $J_{3eq,3ax}$ 12.9 Hz, $J_{3eq,4}$ 4.5 Hz, H-3_{eq}), 3.26 (3 H, s, OCH_3), 3.70-3.90 (7 H, m, H-7, H-8, H-9A, H-9B and CO_2CH_3), 3.98 (1 H, d, *J* 10.2 Hz, H-6), 4.10-4.25 (2 H, m, H-4 and H-5), 7.47-7.57 (3 H, m, Ar-*H*), 7.70 (1 H, d, *J* 6.9 Hz, Ar-*H*), 7.88-7.98 (2 H, m, Ar-*H*), 8.31 (1 H, d, *J* 7.8 Hz, Ar-*H*); ^{13}C NMR (75.5 MHz, CD_3OD): δ 42.0 (C-3), 51.6 (OCH_3), 53.2 (CO_2CH_3), 54.2 (C-5), 65.3 (C-9), 67.7 (C-4), 70.3 (C-7), 71.5 (C-6*), 72.3 (C-8*), 100.5 (C-2), 125.8, 126.5, 126.6, 127.4, 128.0, 129.3 (6 x Ar*C*), 131.4 (ArC_q), 131.5 (Ar*C*), 135.1. 135.5 (2 x ArC_q), 171.0 (C-1), 173.9 (*C*ONH); LRMS (ESI): *m/z* 472.2 [$(M+Na)^+$ 100%]; HRMS (ESI): calcd for $C_{22}H_{27}NNaO_9$ $[M+Na]^+$ 472.1578, found 472.1576.

General procedure for base catalyzed de-esterification of compounds 20a-i.
The esterified C-5 amide derivative was taken up in 0.1 M NaOH/MeOH (1:1) at 0 °C. The reaction mixture was stirred at rt and monitored by TLC (EtOAc/MeOH, 5:1). After 16 h, the reaction mixture was acidified to pH 8 with Amberlite® IR-120 (H^+), the resin was filtered-off and the filtrate evaporated under reduced pressure to afford a yellow syrup. The crude product was purified over a Sephadex™ LH-20 column ($MeOH/H_2O$, 1:10 1:5) followed by lyophilization to afford an amorphous solid.

Methyl 5-butanamido-3,5-dideoxy-D-*glycero*-β-D-*galacto*-non-2-ulopyranosidonic acid, sodium salt (21a).
Prepared from **20a** (0.07 g, 0.19 mmol), according to the general procedure for de-esterification (56 mg, 84% yield). ^{1}H NMR (300 MHz, D_2O): δ 0.93 (3 H, t, *J* 7.5 Hz, H-c), 1.57-1.70 (3 H, m, H-b and H-3ax), 2.28 (2 H, t, *J* 7.5 Hz, H-a), 2.35 (1 H, dd, $J_{3eq,3ax}$ 13.2 Hz, $J_{3eq,4}$ 4.8 Hz, H-3_{eq}), 3.26 (3 H, s, OCH_3), 3.54 (1 H, d, *J* 9.3 Hz, H-7), 3.66 (1 H, dd, $J_{9A,9B}$ 11.7 Hz, $J_{9A,8}$ 5.4 Hz, H-9A), 3.79-3.94 (4 H, m, H-5, H-6, H-8 and H-9B), 3.97-4.06 (1 H, m, H-4); ^{13}C NMR (75.5 MHz, D_2O): δ 12.8 (C-c), 19.0 (C-b), 37.9 (C-a), 39.8 (C-3), 50.4 (OCH_3), 51.8 (C-5), 63.5 (C-9), 66.9 (C-4), 68.4 (C-7), 69.9 (C-6*), 70.2 (C-8*), 175.0 (C-1), 177.8 (*C*ONH), [C-2 was not observed]; LRMS (ESI): *m/z* 396.0 [$(M+Na)^+$ 100%].

Methyl 5-cyclopentylcarboxamido-3,5-dideoxy-D-*glycero*-β-D-*galacto*-non-2-ulopyranosidonic acid, sodium salt (21b).
Prepared from **20b** (0.10 g, 0.26 mmol), according to the general procedure for de-esterification (82 mg, 85% yield). ^{1}H NMR (300 MHz, D_2O): δ 1.50-1.61 (6 H, m, cyclopentyl-CH_2), 1.66 (1 H, dd, $J_{3ax,3eq}$ 13.2 Hz, $J_{3ax,4}$ 11.4 Hz, H-3_{ax}), 1.76-1.86 (2 H, m, cyclopentyl-CH_2), 2.28 (1 H, dd, $J_{3eq,3ax}$ 13.2 Hz, $J_{3eq,4}$ 4.8 Hz, H-3_{eq}), 2.62-2.67 (1 H, m, H-a), 3.26 (3 H, s, OCH_3), 3.47 (1 H, d, *J* 9.6 Hz, H-7), 3.61 (1 H, dd, $J_{9A,9B}$ 12.0 Hz, $J_{9A,8}$ 6.0 Hz, H-9A), 3.78-3.83 (4 H, m, H-5, H-6, H-8 and H-9B), 3.93-3.99 (1 H, m, H-4); ^{13}C NMR (75.5 MHz, D_2O): δ 25.5 (C-c), 25.6 (C-c'), 29.7 (C-b), 30.5 (C-b'), 39.5 (C-3), 45.3 (C-a), 50.7 (OCH_3), 51.6 (C-5), 63.4 (C-9), 66.4 (C-4), 68.2 (C-7), 69.8 (C-6*), 70.5 (C-8*), 172.7 (C-1), 181.0 (*C*ONH), [C-2 was not observed]; LRMS (ESI): *m/z* 400.1 [$(M+H)^+$ 100%].

Methyl 5-cyclohexylcarboxamido-3,5-dideoxy-D-*glycero*-β-D-*galacto*-non-2-ulopyranosidonic acid, sodium salt (21c).
Prepared from **20c** (0.10 g, 0.25 mmol), according to the general procedure for de-esterification (85 mg, 85% yield). ^{1}H NMR (300 MHz, D_2O): δ 1.17-1.45 (5 H, m, cyclohexyl-CH_2), 1.65-1.84 (6H, m, H-3_{ax} and cyclohexyl-CH_2), 2.24-2.33 (1H, m, H-a), 2.33 (1 H, dd, $J_{3ax,3eq}$ 13.2 Hz, $J_{3eq,4}$ 5.1 Hz, H-3_{eq}), 3.27 (3 H, s, OCH_3), 3.53 (1 H, d, *J* 9.6 Hz, H-7), 3.66 (1 H, dd, $J_{9A,9B}$ 12.0 Hz, $J_{9A,8}$ 5.7 Hz, H-9A), 3.82-3.94 (4 H, m, H-5, H-6, H-8 and H-9B), 4.02-4.10 (1 H, m, H-4); ^{13}C NMR (75.5 MHz, D_2O): δ 25.0, 25.2 (C-c, C-c', C-d), 28.8 (C-b), 29.5 (C-b'), 39.4 (C-3), 45.2 (C-a), 50.8 (OCH_3), 51.4 (C-5), 63.3 (C-9), 66.2 (C-4), 68.1 (C-7), 69.7 (C-6*), 70.6 (C-8*), 99.4 (C-2), 172.4 (C-1), 181.0 (*C*ONH); LRMS (ESI): *m/z* 414.1 [$(M+H)^+$ 100%].

Methyl 3,5-dideoxy-5-(2-hydroxyacetamido)-D-*glycero*-β-D-*galacto*-non-2-ulopyranosidonic acid (21d).
Prepared from **20d** (0.10 g, 0.26 mmol), according to the general procedure for de-esterification (79 mg, 90% yield). Characterized as the free acid. ^{1}H NMR (300 MHz, D_2O): δ 1.68 (1 H, dd, $J_{3ax,3eq}$ 13.2, $J_{3ax,4}$ 11.4 Hz, H-3_{ax}), 2.36 (1 H, dd,

$J_{3eq,3ax}$ 13.2, $J_{3eq,4}$ 5.1 Hz, H-3$_{eq}$), 3.22 (3 H, s, OC*H*$_3$), 3.54 (1 H, app.d, $J_{7,8}$ 9.3 Hz, H-7), 3.68 (1 H, dd, $J_{9A,9B}$ 11.7, $J_{9A,8}$ 5.7 Hz, H-9A), 3.84-4.14 (5 H, m, H-4, H-5, H-6, H-8, H-9B), 4.17 (2 H, s, COC*H*$_2$OH); ^{13}C NMR (75.5 MHz, D_2O): δ 39.3 (C-3), 50.7 (O*C*H$_3$), 51.4 (C-5), 60.9 (C-9), 63.3 (*C*H$_2$OH), 66.3 (C-4), 67.9 (C-7), 69.9 (C-8), 70.2 (C-6), 99.6 (C-2), 172.9 (NH*C*O), 175.6 (C-1); LRMS (ESI): *m/z* 338.0 [(M-Na)$^-$ 100%]; HRMS (ESI): calcd for $C_{12}H_{20}NO_{10}$ [M-Na]$^-$ 338.1093, found 338.1091.

Methyl 5-(5-carboxy-pentamido)-3,5-dideoxy-D-*glycero*-β-D-*galacto*-non-2-ulopyranosidonic acid, sodium salt (21e). Prepared from **20e** (0.10 g, 0.23 mmol), according to the general procedure for de-esterification (85 mg, 90% yield). ^{1}H NMR (300 MHz, D_2O): δ 1.61- 1.69 (4 H, m, H-b and H-c), 1.74 (1 H, dd, $J_{3ax,3eq}$ 13.2 Hz, $J_{3ax,4}$ 11.4 Hz, H-3$_{ax}$), 2.32-2.45 (5 H, m, H-3$_{eq}$, H-a and H-d), 3.27 (3 H, s, OC*H*$_3$), 3.56 (1 H, d, *J* 9.9 Hz, H-7), 3.66 (1 H, dd, $J_{9A,9B}$ 12.3 Hz, $J_{9A,8}$ 6.0 Hz, H-9A), 3.82-3.90 (3 H, m, H-6, H-8 and H-9B), 3.93 (1 H, app.t, *J* 10.5 Hz, H-5), 4.00-4.09 (1 H, m, H-4); ^{13}C NMR (75.5 MHz, D_2O): δ 23.7 (C-b), 24.7 (C-c), 33.3 (C-a), 35.6 (C-d), 39.4 (C-3), 50.7 (O*C*H$_3$), 51.6 (C-5), 63.3 (C-9), 66.4 (C-4), 68.1 (C-7), 69.8 (C-6), 70.4 (C-8), 177.3 (C-1, C-e, *C*O$_2$H), 178.5 (*C*ONH), [C-2 was not observed]; LRMS (ESI): *m/z* 432.1 [(M(CO_2H/CO_2Na)+H)$^+$ 100%].

Methyl 3,5-dideoxy-5-(3-phenyl-propanamido)-D-*glycero*-β-D-*galacto*-non-2-ulopyranosidonic acid, sodium salt (21f). Prepared from **20f** (0.08 g, 0.19 mmol), according to the general procedure for de-esterification (68 mg, 88% yield). ^{1}H NMR (300 MHz, D_2O): δ 1.66 (1 H, dd, $J_{3ax,3eq}$ 13.2 Hz, $J_{3ax,4}$ 11.1 Hz, H-3$_{ax}$), 2.33 (1 H, dd, $J_{3eq,3ax}$ 13.2 Hz, $J_{3eq,4}$ 4.8 Hz, H-3$_{eq}$), 2.66 and 2.97 (2 x 2 H, 2 x app.t, *J* 7.2 Hz, H-a and H-b), 3.14 (1 H, d, *J* 9.3 Hz, H-7), 3.21 (3 H, s, OC*H*$_3$), 3.51-3.88 (5 H, m, H-5, H-6, H-8, H-9A and H-9B), 3.91-4.00 (1 H, m, H-4), 7.28-7.41 (5 H, m, Ar-*H*); ^{13}C NMR (75.5 MHz, D_2O): δ 30.9 (C-a), 37.2 (C-b), 39.5 (C-3), 50.6 (O*C*H$_3$), 51.7 (C-5), 63.8 (C-9), 66.7 (C-4), 68.4 (C-7), 70.0 (C-6*), 70.0 (C-8*), 126.5, 128.3, 128.7 (Ar*C*), 140.6 (*ipso*-Ar*C*), 173.8 (C-1), 176.3 (*C*ONH), [C-2 was not observed]; LRMS (ESI): *m/z* 436.1 [(M+H)$^+$ 100%].

Methyl 3,5-dideoxy-5-(4-phenoxy-butanamido)-D-*glycero*-β-D-*galacto*-non-2-ulopyranosidonic acid, sodium salt (21g). Prepared from **20g** (0.11 g, 0.24 mmol), according to the general procedure for de-esterification (90 mg, 90% yield). ^{1}H NMR (300 MHz, D_2O): δ 1.75 (1 H, dd, $J_{3ax,3eq}$ 13.2 Hz, $J_{3ax,4}$ 11.1 Hz, H-3$_{ax}$), 2.05-2.14 (2 H, m, H-b), 2.37 (1 H, dd, $J_{3eq,3ax}$ 13.2 Hz, $J_{3eq,4}$ 4.8 Hz, H-3$_{eq}$), 2.51 (2 H, app.t, *J* 7.2 Hz, H-c), 3.26 (3 H, s, OC*H*$_3$), 3.50-3.58 (2 H, m, H-7 and H-9A), 3.76-3.88 (3 H, m, H-6, H-8 and H-9B), 3.94 (1 H, app.t, *J* 9.9 Hz, H-5), 3.98-4.04 (1 H, m, H-4), 4.06-4.16 (2 H, m, H-a), 7.03-7.09 (3 H, m, Ar-*H*), 7.36-7.42 (3 H, m, Ar-*H*); ^{13}C NMR (75.5 MHz, D_2O): δ 24.7 (C-b), 32.2 (C-c), 39.4 (C-3), 52.7 (O*C*H$_3$), 51.6 (C-5), 63.4 (C-9), 66.4 (C-4), 67.1 (C-a), 68.2 (C-7), 69.8 (C-6*), 70.4 (C-8*), 114.9, 121.4, and 129.8 (Ar*C*), 157.9 (*ipso*-Ar*C*), 172.4 (C-1), 176.6 (*C*ONH), [C-2 was not observed]; LRMS (ESI): *m/z* 466.1 [(M+H)$^+$ 100%].

Methyl 3,5-dideoxy-5-(4-phenyl-benzamido)-D-*glycero*-β-D-*galacto*-non-2-ulopyranosidonic acid, sodium salt (21h). Prepared from **20h** (0.12 g, 0.25 mmol), according to the general procedure for de-esterification (99 mg, 90% yield). ^{1}H NMR (300 MHz, CD_3OD): δ 1.61-1.76 (1 H, br.s, H-3$_{ax}$), 2.37-2.54 (1 H, br.s, H-3$_{eq}$), 3.26† (3 H, s, OC*H*$_3$), 3.55-4.25† (7 H, m, H-4, H-5, H-6, H-7, H-8, H-9A and H-9B), 7.34-7.47 (3 H, m, Ar-*H*), 7.63-7.70 (4 H, m, Ar-*H*), 8.00 (2 H, d, *J* 8.1 Hz, Ar-*H*); ^{13}C NMR (75.5 MHz, CD_3OD): δ 42.1 (C-3), 51.4 (O*C*H$_3$), 54.4 (C-5), 65.1 (C-9), 68.4 (C-4*), 70.2 (C-7*), 71.8 (C-6*), 72.1 (C-8*), 127.9, 128.1, 129.1, 129.4, and 130.0 (Ar*C*), 134.2, 141.2 and 145.6 (Ar*C*$_q$), 171.0 (*C*ONH), [C-1 and C-2 were not observed]; LRMS (ESI): *m/z* 484.1 [(M+H)$^+$ 100%].

Methyl 3,5-dideoxy-5-(1-naphthylcarboxamido)-D-*glycero*-β-D-*galacto*-non-2-ulopyranosidonic acid (21i). Prepared from **20i** (145 mg, 0.23 mmol), according to the general procedure for de-esterification (105 mg, 85% yield). HPLC: 3:97 CH_3CN/H_2O; retention time 8.5-12.0 min; ^{1}H NMR (300 MHz, D_2O; predominantly acid, contains trace triethylammonium salt): δ 1.27 [1.5 H equiv., t, *J* 7.2 Hz, N(CH$_2$C*H*$_3$)$_3$], 1.78 (1 H, dd, $J_{3ax,3eq}$ 13.2 Hz, $J_{3ax,4}$ 11.4 Hz, H-3$_{ax}$), 2.44 (1 H, dd, $J_{3eq,3ax}$ 13.2 Hz, $J_{3eq,4}$ 4.8 Hz, H-3$_{eq}$), 3.18 [1 H equiv., q, *J* 7.2 Hz, N(C*H*$_2$CH$_3$)$_3$], 3.23 (3 H, s, OC*H*$_3$), 3.74 (1 H, dd, $J_{9A,9B}$ 12.0 Hz, $J_{9A,8}$ 5.7 Hz, H-9A), 3.82 (1 H, dd, $J_{7,8}$ 9.3 Hz, $J_{7,6}$ 0.9 Hz, H-7), 3.89-4.00 (3 H, m, H-6, H-8 and H-9B), 4.11-4.19 (1 H, m, H-4), 4.28 (1 H, app.t, *J* 10.2 Hz, H-5), 7.57-7.73 (4 H, m, Ar-*H*), 8.01-8.19 (3 H, m, Ar-*H*); ^{13}C NMR (75.5 MHz, D_2O): δ 8.1 [N(*C*H$_3$CH$_2$)$_3$], 40.2 (C-3), 46.6 [N(CH$_3$*C*H$_2$)$_3$], 50.4 (O*C*H$_3$), 52.5 (C-5), 63.5 (C-9), 67.0 (C-4), 68.7 (C-7), 70.1 (C-6*), 70.2 (C-8*), 100.5 (C-2), 124.5, 125.1, 125.3, 126.7, 127.4, and 128.4 (6 x Ar*C*), 129.2 (Ar*C*$_q$), 130.8 (Ar*C*), 133.1 and 133.2 (2 x Ar*C*$_q$) (10 x Ar*C*), 173.1 (C-1), 175.3 (*C*ONH); LRMS (ESI): *m/z* 434.0 [(M-Na)$^-$ 100%].

Synthesis of C-4 amide derivatives of Neu5Acβ2Me

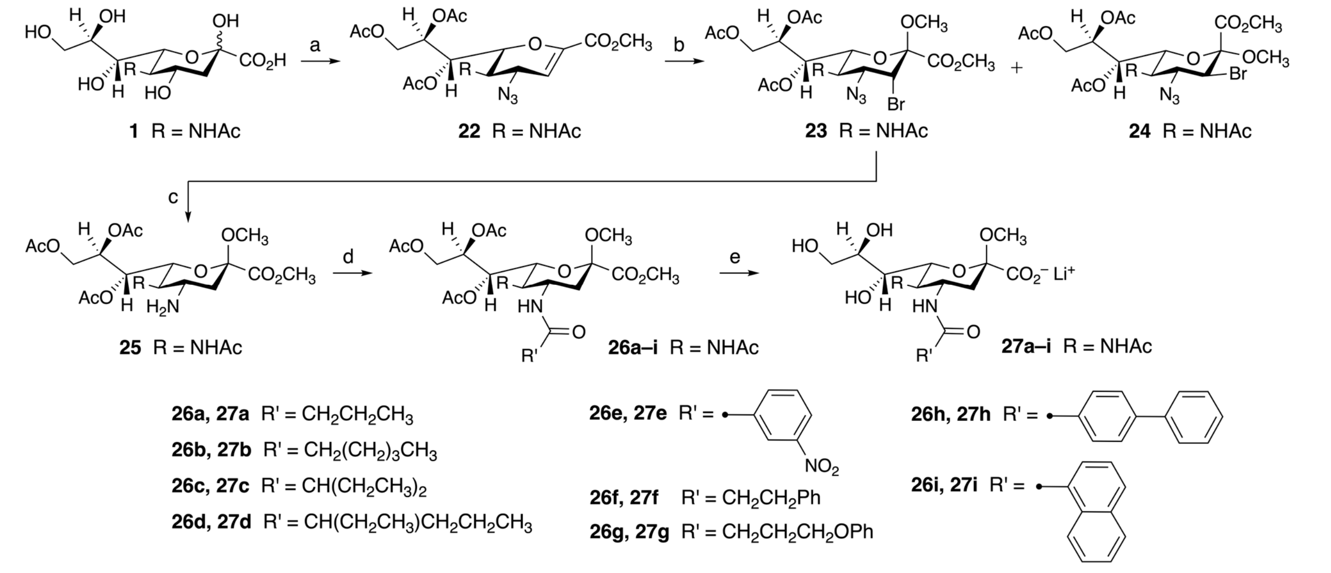

Scheme S4. Synthesis of C-4 amido derivatives of 4-deoxy-Neu5Acβ2Me. *Reagents and conditions:* (a) over 4 steps according to the reported procedures [2]: (i) MeOH, H^+ ion exchange resin, rt, 16 h; (ii) Ac_2O, pyridine, rt, 16 h; (iii) TMSOTf, EtOAc, 50 °C, 2 h; (iv) $TMSN_3$, *t*-BuOH, reflux, 6 h; (b) according to the reported procedure [3]: NBS, MeOH, 0 – 5 °C, 72 h (**23** + **24**, 87%); (c) according to the reported procedure [3]: Bu_3SnH, AIBN, dioxane, 65 °C, 16 h (77%); (d) R'COCl , Et_3N, DCM, 0 °C – rt, 2 h (**26a** 55%, **26b** 67%, **26c** 60%, **26d** 65%, **26e** 65%, **26f** 65%, **26g** 65%, **26h** 60%, **26i** 65%); (e) LiOH, MeOH/H_2O (1:1), pH 13, 0 °C – rt, 16 h. In **26**,**27e,h,i**, the sphere indicates the point of attachment of R' to the amide carbon.

Methyl (methyl 5-acetamido-7,8,9-tri-*O*-acetyl-4-amino-3,4,5-trideoxy-D-*glycero*-β-D-*galacto*-non-2-ulopyranosid)onate (25).

According to the method reported by Ciccotosto ***et al*** [3], methyl (methyl 5-acetamido-7,8,9-tri-*O*-acetyl-4-azido-3-bromo-3,4,5-trideoxy-β-D-*erythro*-L-*manno*-non-2-ulopyranosid)onate (**23** [3]) (3.1 g, 5.46 mmol) was dissolved in anhydrous 1,4-dioxane (60 ml) under argon and tributyltin hydride (5.88 mL, 21.86 mmol) and azobisisobutyronitrile (89 mg, 0.54 mmol) were added successively. The reaction was heated to 65 °C and stirred for 16 h before being cooled to rt. The solvent was removed under vacuum and the residue diluted with CH_3CN (25 ml) and washed with hexane (3 x 20 mL), concentrated under reduced pressure. The crude product was purified by flash chromatography (EtOAc/MeOH/H_2O 7:2:1) to afford **25** as a white solid (1.94 g, 77%). R_f 0.35 (EtOAc/MeOH/H_2O 7:2:1); ^{1}H NMR (300 MHz, $CDCl_3$): δ 1.68 (1 H, t, $J_{3ax,4} \approx J_{3ax,3eq}$ 13.2 Hz, H-3ax), 1.81 (3 H, s, NHCOC*H*3), 1.90, 1.94, 2.00 (3 x 3 H, 3 x s, 3 x OCOC*H*3), 2.25 (1 H, dd, $J_{3eq,3ax}$ 13.2 Hz, $J_{3eq,4}$ 4.5, H-3eq), 3.08 (3 H, s, OC*H*3), 3.17-3.26 (1 H, m, H-4), 3.66 (1 H, dd, *J* 10.5, H-5), 3.69 (3 H, s, CO_2CH_3), 3.92 (1 H, dd, $J_{6,5}$ 10.5 Hz, $J_{6,7}$ 1.8 Hz, H-6), 4.05 (1 H, dd, $J_{9A,9B}$ 12.6 Hz, $J_{9A,8}$ 6.3 Hz, H-9A), 4.51 (1 H, dd, $J_{9B,9A}$ 12.6 Hz, $J_{9B,8}$ 2.7, H-9B), 5.14-5.19 (1 H, m, H-8), 5.26 (1 H, dd, $J_{7,8}$ 5.4 Hz, $J_{7,6}$ 1.8 Hz, H-6); LRMS (ESI): *m/z* 485.3 [$(M+Na)^+$ 100%].

Methyl [methyl 5-acetamido-7,8,9-tri-*O*-acetyl-4-butanamido-3,4,5-trideoxy-D-*glycero*-β-D-*galacto*-non-2-ulopyranosid]onate (26a).

To a solution of **25** (0.2 g, 0.43 mmol) in anhyd CH_2Cl_2 (10 mL) at rt under argon, was added Et_3N (0.5 mL). The reaction mixture was then cooled to 0 °C and butyryl chloride (0.07 mL, 0.65 mmol) in CH_2Cl_2 (0.5 mL) was added drop wise. The reaction temperature was raised to rt and the progress of reaction was monitored by TLC (hexane/EtOAc, 1:5). After 2 h, the reaction mixture was concentrated under reduced pressure to give a yellow syrup, which was purified by flash chromatography (hexane/EtOAc, 1:1 1:5) to furnish **26a** (120 mg, 55%) as a yellow solid. R_f 0.20 (hexane/EtOAc 1:5); ^{1}H NMR (300 MHz, $CDCl_3$): δ 0.87 (3 H, app.t, *J* 7.5 Hz, H-c), 1.50-1.57 (2 H, m, H-b), 1.63 (1 H, t, $J_{3ax,4} \approx J_{3ax,3eq}$ 12.3 Hz, H-3ax), 1.87 (3 H, s, NHCOC*H*3), 2.01, 2.05, 2.10 (3 x 3 H, 3 x s, 3 x OCOC*H*3), 2.40 (1 H, dd, $J_{3eq,3ax}$ 12.9 Hz, $J_{3eq,4}$ 3.6 Hz, H-3eq), 3.24 (3 H, s, OC*H*3), 3.78 (3 H, s, CO_2CH_3), 3.85-3.96 (2 H, m, H-5 and H-6), 4.10 (1 H, dd, $J_{9A,9B}$ 12.3 Hz, $J_{9A,8}$ 7.5 Hz, H-9A), 4.32-4.43 (1 H, m, H-4), 4.76 (1 H, dd, $J_{9B,9A}$ 12.3 Hz, $J_{9B,8}$ 2.4 Hz, H-9B), 5.21-5.24 (1 H, m, H-8), 5.41 (1 H, d, *J* 3.6 Hz, H-7), 5.65 (1 H, d, *J* 8.4 Hz, N*H*CO), 5.70 (1 H, d, *J* 9.0 Hz, N*H*COCH3); ^{13}C NMR (75.5 MHz, $CDCl_3$): δ 13.6 (C-c), 19.0 (C-a), 20.7, 20.7, 20.9 (3 x OCOCH3), 22.9 (NHCOCH3), 38.0 (C-b), 38.4 (C-3), 46.9 (C-4), 49.1 (C-5), 51.1 (OCH3), 52.5 (CO_2CH_3), 62.4 (C-9), 68.7 (C-

7), 71.7 (C-8), 71.9 (C-6), 98.4 (C-2), 167.8 (C-1), 169.9, 170.6, 171.6, 173.6 (NHCO, 3 x COCH3, NHCOCH3); LRMS (ESI): *m/z* 555.1 [(M+Na)$^+$ 100%]; HRMS (ESI): calcd for $C_{23}H_{37}N_2O_{12}$ [M+H]$^+$ 533.2341, found 533.2363.

Methyl [methyl 5-acetamido-7,8,9-tri-*O*-acetyl-3,4,5-trideoxy-4-hexanamido-D-*glycero*-β-D-*galacto*-non-2-ulopyranosid]onate (26b).

Compound **26b** was prepared by coupling **25** (0.12 g, 0.26 mmol) with hexanoyl chloride (0.04 mL, 0.3 mmol) in a similar manner to that described for the synthesis of **26a** from **25**. The crude reaction product was purified by flash chromatography (hexane/EtOAc, 1:1 1:5) to give the desired compound **26b** (97 mg, 67%) as a white solid. R_f 0.27 (hexane/EtOAc 1:5); ^{1}H NMR (300 MHz, $CDCl_3$): δ 0.84 (3 H, app.t, *J* 6.9 Hz, H-e), 1.24-1.27 (4 H, m, 2 x C*H*$_2$), 1.49-1.57 (2 H, m, C*H*$_2$) 1.63 (1H, t, $J_{3ax,4} \approx J_{3ax,3eq}$ 12.9 Hz, H-3ax), 1.87 (3 H, s, NHCOC*H*$_3$), 2.00, 2.05, 2.09 (3 x 3 H, 3 x s, 3 x OCOC*H*$_3$), 1.90-2.30 [(obscured) 2 H, m, C*H*$_2$], 2.41 (1 H, dd, $J_{3eq,3ax}$ 12.9 Hz, $J_{3eq,4}$ 3.6 Hz, H-3eq), 3.23 (3 H, s, OC*H*$_3$), 3.77 (3 H, s, CO_2CH_3), 3.86-3.95 (2 H, m, H-5 and H-6), 4.10 (1 H, dd, $J_{9A,9B}$ 12.3 Hz, $J_{9A,8}$ 7.5 Hz, H-9A), 4.19-4.28 (1 H, m, H-4), 4.76 (1 H, dd, $J_{9A,9B}$ 12.6 Hz, $J_{9A,8}$ 2.4 Hz, H-9B), 5.19-5.23 (1 H, m, H-8), 5.41 (1 H, d, *J* 3.0 Hz, H-7), 5.75 (1 H, d, *J* 8.4 Hz, N*H*CO), 5.84 (1 H, d, *J* 8.1 Hz, N*H*COCH$_3$); ^{13}C NMR (75.5 MHz, $CDCl_3$): δ 13.9 (C-e), 20.8, 21.0, 21.1 (3 x OCO*C*H$_3$), 22.4 (*C*H$_2$), 23.0 (NHCO*C*H$_3$), 25.4 (*C*H$_2$), 31.4 (*C*H$_2$), 36.7 (*C*H$_2$), 38.1 (C-3), 47.1 (C-4), 49.2 (C-5), 51.2 (O*C*H$_3$), 52.8 (CO_2CH_3), 62.4 (C-9), 68.7 (C-7), 71.7 (C-8), 71.8 (C-6), 98.4 (C-2), 167.8 (C-1), 170.0, 170.7, 171.8, 174.0 (NH*C*O, 3 x *C*OCH$_3$, NH*C*OCH$_3$); LRMS (ESI): *m/z* 583.2 [(M+Na)$^+$ 100%]; HRMS (ESI): calcd for $C_{25}H_{41}N_2O_{12}$ [M+H]$^+$ 561.2654, found 561.2665.

Methyl [methyl 5-acetamido-7,8,9-tri-*O*-acetyl-3,4,5-trideoxy-4-(2-ethyl-butanamido)-D-*glycero*-β-D-*galacto*-non-2-ulopyranosid]onate (26c).

Compound **26c** was prepared by coupling **25** (0.2 g, 0.43 mmol) with 2-ethylbutyryl chloride (0.08 mL, 0.64 mmol) in a similar manner to that described for the synthesis of **26a** from **25**. The crude reaction product was purified by flash chromatography (hexane/EtOAc, 1:1 1:5) to give the desired compound **26c** (145 mg, 60%) as a white solid. R_f 0.53 (hexane/EtOAc 1:5); ^{1}H NMR (300 MHz, $CDCl_3$): δ 0.76-0.82 (6 H, m, H-c and H-c'), 1.34-1.52 (4 H, m, H-b and H-b'), 1.60 [1 H, app.t, H-3ax], 1.71-1.79 (1 H, m, H-a), 1.85 (3 H, s, NHCOC*H*$_3$), 2.01, 2.05, 2.10 (3 x 3 H, 3 x s, 3 x OCOC*H*$_3$), 2.42 (1 H, dd, $J_{3eq,3ax}$ 12.9 Hz, $J_{3eq,4}$ 4.2 Hz, H-3eq), 3.24 (3 H, s, OC*H*$_3$), 3.78 (3 H, s, CO_2CH_3), 3.90-3.95 (2 H, m, H-5 and H-6), 4.11 (1 H, dd, $J_{9A,9B}$ 12.3 Hz, $J_{9A,8}$ 7.5 Hz, H-9A), 4.32-4.48 (1 H, m, H-4), 4.78 (1 H, dd, $J_{9B,9A}$ 12.3 Hz, $J_{9B,8}$ 2.4 Hz, H-9B), 5.19-5.27 (1 H, m, H-8), 5.42 (1 H, d, *J* 3.3 Hz, H-7), 5.65 (1 H, d, *J* 8.4 Hz, N*H*CO), 5.75 (1 H, d, *J* 8.1 Hz, N*H*COCH$_3$); ^{13}C NMR (75.5 MHz, $CDCl_3$): δ 11.8 and 11.9 (C-c and C-c'), 20.6, 20.7, 21.0 (3 x OCO*C*H$_3$), 23.0 (NHCO*C*H$_3$), 25.5 and 25.6 (C-b and C-b'), 38.4 (C-3), 46.9 (C-4), 48.4 (C-5), 51.1 (O*C*H$_3$), 51.2 (C-a), 52.8 (CO_2CH_3), 62.4 (C-9), 68.7 (C-7), 71.7 (C-8), 72.1 (C-6), 98.4 (C-2), 162.3 (C-1), 167.7, 169.9, 170.6, 170.8, 171.7 and 176.1 (NH*C*O, 3 x O*C*OCH$_3$, NH*C*OCH$_3$); LRMS (ESI): *m/z* 583.2 [(M+Na)$^+$ 100%]; HRMS (ESI): calcd for $C_{25}H_{41}N_2O_{12}$ [M+H]$^+$ 561.2654, found 561.2666.

Methyl [methyl 5-acetamido-7,8,9-tri-*O*-acetyl-3,4,5-trideoxy-4-(2-ethyl-hexanamido)-D-*glycero*-β-D-*galacto*-non-2-ulopyranosid]onate (26d).

Compound **26d** was prepared by coupling **25** (0.2 g, 0.43 mmol) with 2-ethylhexanoyl chloride (0.11 mL, 0.64 mmol) in a similar manner to that described for the synthesis of **26a** from **25**. The crude reaction product was purified by flash chromatography (hexane/EtOAc, 1:1 1:5) to give the desired compound **26d** (165 mg, 65%) as a white solid. R_f 0.53 and 0.58 (hexane/EtOAc 1:5); ^{1}H NMR (300 MHz, $CDCl_3$): δ 0.75-0.85 [6 H, m, H-e (Hex) and H-g (Et)], 1.08-1.52 (8 H, m, H-b, H-c, H-d and H-f), 1.70-1.90 (1 H, m, H-a), 1.85, 1.86† (3 H, s, NHCOC*H*$_3$), 2.00, 2.05, 2.10 (3 x 3 H, 3 x s, 3 x OCOC*H*$_3$), 2.39, 2.43† (1 H, dd, $J_{3eq,3ax}$ 12.9 Hz, $J_{3eq,4}$ 3.6 Hz, H-3eq), 3.24 (3 H, s, OC*H*$_3$), 3.78 (3 H, s, CO_2CH_3), 3.85-3.96 (2 H, m, H-5 and H-6), 4.10 (1 H, dd, $J_{9A,8}$ 7.2 Hz, $J_{9A,9B}$ 12.3 Hz, H-9A), 4.32-4.45 (1 H, m, H-4), 4.75 (1 H, d, *J* 12.3 Hz, H-9B), 5.20-5.23 (1 H, m, H-8), 5.40 (1 H, d, *J* 2.4 Hz, H-7), 5.59-5.62 (2 H, m, N*H*CO and N*H*COCH$_3$); ^{13}C NMR (75.5 MHz, $CDCl_3$): δ 11.8, 12.0† (C-e*), 13.8 (C-g*), 20.6, 20.7 and 20.9 (3 x OCO*C*H$_3$), 22.5, 22.6† (C-f*), 23.0 (NHCO*C*H$_3$), 25.7, 25.9† (C-d), 29.4, 29.6† (C-c*), 32.2 (C-b*), 38.3 (C-3), 46.9 (C-a), 48.4, 48.5† (C-4), 49.5, 49.6† (C-5), 51.1 (O*C*H$_3$), 52.5 (CO_2CH_3), 62.4 (C-9), 68.7 (C-7), 71.7, 71.8† (C-8), 72.1, 72.2† (C-6), 98.4 (C-2), 167.8 (C-1), 169.9, 170.6, 170.8, 171.6, 171.7 and 176.2 (NH*C*O, 3 x *C*OCH$_3$, NH*C*OCH$_3$), [†Assignment belongs to corresponding diastereomer]; LRMS (ESI): *m/z* 611.2 [(M+Na)$^+$ 100%]; HRMS (ESI): calcd for $C_{27}H_{45}N_2O_{12}$ [M+H]$^+$ 589.2967, found 589.2961.

Methyl [methyl 5-acetamido-7,8,9-tri-*O*-acetyl-3,4,5-trideoxy-4-(3-nitrobenzamido)-D-*glycero*-β-D-*galacto*-non-2-ulopyranosid]onate (26e).

Compound **26e** was prepared by coupling **25** (0.2 g, 0.43mmol) with 3-nitrobenzoyl chloride (0.12 g, 0.64 mmol) in a similar manner to that described for the synthesis of **26a** from **25**. The crude reaction product was purified by flash chromatography

(hexane/EtOAc, 1:1 1:5) to give the desired compound **26e** (172 mg, 65%) as a white solid. R_f 0.5 (hexane/EtOAc 1:5); ^{1}H NMR (300 MHz, $CDCl_3$): δ 1.75 (1 H, t, $J_{3ax,4} \approx J_{3ax,3eq}$ 12.6 Hz, H-3ax), 1.87 (3 H, s, NHCOC*H*$_3$), 2.02, 2.07, 2.11 (3 x 3 H, 3 x s, 3 x OCOC*H*$_3$), 2.62 (1 H, dd, $J_{3eq,3ax}$ 13.2 Hz, $J_{3eq,4}$ 4.5 Hz, H-3eq), 3.27 (3 H, s, OC*H*$_3$), 3.78 (3 H, s, CO_2C*H*$_3$), 4.00-4.06 (3 H, m, H-5 and H-6), 4.14 (1 H, dd, $J_{9A,9B}$ 12.3 Hz, $J_{9A,8}$ 7.5 Hz, H-9A), 4.45-4.58 (1 H, m, H-4), 4.79 (1 H, dd, $J_{9A,9B}$ 12.3 Hz, $J_{9A,8}$ 2.4, H-9B), 5.23-5.27 (1 H, m, H-8), 5.50 (1 H, d, *J* 2.7 Hz, H-7), 5.86 (1 H, d, *J* 8.4 Hz, N*H*COCH$_3$), 6.94 (1 H, d, *J* 7.8 Hz, N*H*CO), 7.59 (1 H, t, *J* 7.8, Ar*H*), 8.00 (1 H, d, *J* 7.8 Hz, Ar*H*), 8.30 (1 H, dd, *J* 1.2 Hz, *J* 7.8 Hz, Ar*H*), 8.58 (1 H, s, Ar*H*); ^{13}C NMR (75.5 MHz, $CDCl_3$): δ 20.6, 20.7, 20.9 (3 x OCO*C*H$_3$), 22.9 (NHCO*C*H$_3$), 37.7 (C-3), 48.6 (C-4), 48.9 (C-5), 51.1 (O*C*H$_3$), 52.6 (CO_2*C*H$_3$), 62.4 (C-9), 68.7 (C-7), 71.5 (C-8), 72.1 (C-6), 98.4 (C-2), 122.0, 126.0, 129.7, 133.1 (4 x Ar*C*), 135.2, 147.9 (2 x Ar*C*$_q$), 165.2 (C-1), 167.6, 169.9, 170.2, 170.7, 170.8, 172.4 (NH*C*O, 3 x O*C*OCH$_3$, NH*C*OCH$_3$); LRMS (ESI): *m/z* 634.1 [(M+Na)$^+$ 100%]; HRMS (ESI): calcd for $C_{26}H_{34}N_3O_{14}Na$ [M+H]$^+$ 612.2035, found 612.2028.

Methyl [methyl 5-acetamido-7,8,9-tri-*O*-acetyl-3,4,5-trideoxy-4-(3-phenyl-propanamido)-D-*glycero*-β-D-*galacto*-non-2-ulopyranosid]onate (26f).

Compound **26f** was prepared by coupling **25** (0.2 g, 0.43 mmol) with hydrocinnamoyl chloride (0.09 mL, 0.64 mmol) in a similar manner to that described for the synthesis of **26a** from **25**. The crude reaction product was purified by flash chromatography (hexane/EtOAc, 1:1 1:5) to give the desired compound **26f** (160 mg, 65%) as a white solid. R_f 0.44 (hexane/EtOAc 1:5); ^{1}H NMR (300 MHz, $CDCl_3$): δ 1.52 (1 H, t, $J_{3ax,4} \approx J_{3ax,3eq}$ 12.6 Hz, H-3ax), 1.80 (3 H, s, NHCOC*H*$_3$), 2.00, 2.04 and 2.09 (3 x 3 H, 3 x s, 3 x OCOC*H*$_3$), 2.30-2.40 (3 H, m, H-b and H-3eq), 2.83-2.88 (2 H, m, H-a), 3.23 (3 H, s, OC*H*$_3$), 3.77 (3 H, s, CO_2C*H*$_3$), 3.79-3.89 (2 H, m, H-5 and H-6), 4.10 (1 H, dd, $J_{9A,9B}$ 12.6 Hz, $J_{9A,8}$ 7.4 Hz, H-9A), 4.23-4.38 (1 H, m, H-4), 4.75 (1 H, dd, $J_{9B,9A}$ 12.6 Hz, $J_{9B,8}$ 2.4 Hz, H-9B), 5.19-5.23 (1 H, m, H-8), 5.38-5.40 (1 H, m, H-7), 5.60 (2 H, m, N*H*COCH$_3$ and N*H*CO), 7.12-7.15 (3 H, m, Ar*H*), 7.18-7.26 (2 H, m, Ar*H*); ^{13}C NMR (75.5 MHz, $CDCl_3$): δ 20.7, 20.9 (3 x OCO*C*H$_3$), 22.9 (NHCO*C*H$_3$), 31.5 (C-a), 37.9 (C-b), 38.1 (C-3), 47.1 (C-4), 49.1 (C-5), 51.0 (O*C*H$_3$), 52.5 (CO_2*C*H$_3$), 62.4 (C-9), 68.7 (C-7), 71.6 (C-8), 71.9 (C-6), 98.4 (C-2), 126.2, 128.2, 128.4 (5 x Ar*C*), 140.5 (*ipso*-Ar*C*), 167.7 (C-1), 169.8, 170.6, 171.5, 172.5 (NH*C*O, 3 x O*C*OCH$_3$, NH*C*OCH$_3$); LRMS (ESI): *m/z* 617.2 [(M+Na)$^+$ 100%]; HRMS (ESI): calcd for $C_{28}H_{39}N_2O_{12}$ [M+H]$^+$ 595.2497, found 595.2489.

Methyl [methyl 5-acetamido-7,8,9-tri-*O*-acetyl-3,4,5-trideoxy-4-(4-phenoxy-butanamido)-D-*glycero*-β-D-*galacto*-non-2-ulopyranosid]onate (26g).

Compound **26g** was prepared by coupling **25** (0.2 g, 0.43 mmol) with 4-(phenoxy)butyryl chloride (0.11 mL, 0.64 mmol) in a similar manner to that described for the synthesis of **26a** from **25**. The crude reaction product was purified by flash chromatography (hexane/EtOAc, 1:1 1:5) to give the desired compound **26g** (175 mg, 65%) as a white solid. R_f 0.38 (hexane/EtOAc 1:5); ^{1}H NMR (300 MHz, $CDCl_3$): δ 1.58 (1 H, t, $J_{3ax,4} \approx J_{3ax,3eq}$ 12.9 Hz, H-3ax), 1.75 (2H, m, H-b), 1.85 (3 H, s, NHCOC*H*$_3$), 2.00, 2.05, 2.10 (3 x 3 H, 3 x s, 3 x OCOC*H*$_3$), 2.29-2.40 (3 H, m, H-a and H-3eq), 3.23 (3 H, s, OC*H*$_3$), 3.77 (3 H, s, CO_2C*H*$_3$), 3.87-3.95 (4 H, m, H-5, H-6 and H-c), 4.10 (1 H, dd, $J_{9A,9B}$ 12.3 Hz, $J_{9A,8}$ 7.5 Hz, H-9A), 4.28-4.42 (1 H, m, H-4), 4.75 (1 H, dd, $J_{9A,9B}$ 12.3 Hz, $J_{9A,8}$ 2.4 Hz, H-9B), 5.19-5.24 (1 H, m, H-8), 5.40 (1 H, d, *J* 2.7 Hz, H-7), 5.74 (1 H, d, *J* 7.8 Hz, N*H*COCH$_3$), 5.88 (1 H, d, *J* 8.7 Hz, N*H*CO), 6.75-6.93 (3 H, m, Ar*H*), 7.21-7.26 (2 H, m, Ar*H*); ^{13}C NMR (75.5 MHz, $CDCl_3$): δ 20.7, 20.9, 20.9 (3 x OCO*C*H$_3$), 22.9 (NHCO*C*H$_3$), 25.1 (C-b), 32.8 (C-a), 37.9 (C-3), 47.0 (C-4), 49.1 (C-5), 51.1 (O*C*H$_3$), 52.6 (CO_2*C*H$_3$), 62.4 (C-9), 66.5 (C-c), 68.7 (C-7), 71.7 (C-8), 72.0 (C-6), 98.4 (C-2), 114.3, 120.7, 129.4 (Ar*C*), 158.6 (*ipso*-Ar*C*), 167.7 (C-1), 169.9, 170.7, 171.7, 173.1 (NH*C*O, 3 x O*C*OCH$_3$, NH*C*OCH$_3$); LRMS (ESI): *m/z* 647.2 [(M+Na)$^+$ 100%]; HRMS (ESI): calcd for $C_{29}H_{40}N_2O_{13}Na$ [M+Na+H]$^+$ 647.2422, found 647.2416.

Methyl [methyl 5-acetamido-7,8,9-tri-*O*-acetyl-3,4,5-trideoxy-4-(4-phenyl-benzamido)-D-*glycero*-β-D-*galacto*-non-2-ulopyranosid]onate (26h).

Compound **26h** was prepared by coupling **25** (0.2 g, 0.43 mmol) with biphenyl-4-carbonyl chloride (0.14 mL, 0.64 mmol) in a similar manner to that described for the synthesis of **26a** from **25**. The crude reaction product was purified by flash chromatography (hexane/EtOAc, 1:1 1:5) to give the desired compound **26h** (160 mg, 60%) as a white solid. R_f 0.20 (hexane/EtOAc 1:1); ^{1}H NMR (300 MHz, $CDCl_3$): δ [H-3ax obscured], 1.84 (3 H, s, NHCOC*H*$_3$), 2.02, 2.07, 2.13 (3 x 3 H, 3 x s, 3 x OCOC*H*$_3$), 2.62 (1 H, dd, $J_{3eq,3ax}$ 12.9 Hz, $J_{3eq,4}$ 4.5 Hz, H-3eq), 3.28 (3 H, s, OC*H*$_3$), 3.79 (3 H, s, CO_2C*H*$_3$), 4.04-4.18 (3 H, m, H-5, H-6 and H-9A), 4.51-4.65 (1 H, m, H-4), 4.80 (1 H, dd, $J_{9A,9B}$ 12.3 Hz, $J_{9A,8}$ 2.4 Hz, H-9B), 5.24-5.29 (1 H, m, H-8), 5.50 (1 H, d, *J* 3.6 Hz, H-7), 6.03 (1 H, d, *J* 8.1 Hz, N*H*COCH$_3$), 6.63 (1 H, d, *J* 8.1 Hz, N*H*CO) 7.35-7.45 (3 H, m, Ar*H*), 7.54-7.61 (4 H, m, Ar*H*), 7.76 (2 H, d, *J* 3.6 Hz, Ar*H*); ^{13}C NMR (75.5 MHz, CD_3OD): δ 19.2, 19.3, 19.5 (3 x OCO*C*H$_3$), 21.2 (NHCO*C*H$_3$), 37.1 (C-3), 48.4 (C-4), 48.9 (C-5), 50.4 (O*C*H$_3$), 51.7 (CO_2*C*H$_3$), 62.0 (C-9), 68.5 (C-7), 71.0 (C-8), 71.9 (C-6), 98.6 (C-2), 126.6, 126.6, 127.5, 127.6, 128.7 (Ar*C*), 132.8, 139.8, 144.3 (3 x Ar*C*$_q$), 168.0 (C-1), 168.5, 170.1, 170.5, 171.0, 172.5 (NH*C*O, 3 x

O*C*OCH$_3$, NH*C*OCH$_3$); LRMS (ESI): *m/z* 665.2 [(M+Na)$^+$ 100%]; HRMS (ESI): calcd for $C_{32}H_{38}N_2O_{12}Na$ [M+Na+H]$^+$ 665.2316, found 665.2290.

Methyl [methyl 5-acetamido-7,8,9-tri-*O*-acetyl-3,4,5-trideoxy-4-(1-naphthylcarboxamido)-D-*glycero*-β-D-*galacto*-non-2-ulopyranosid]onate (26i).

Compound **26i** was prepared by coupling **25** (0.12 g, 0.26 mmol) with 1-naphthoyl chloride (0.06 mL, 0.39 mmol) in a similar manner to that described for the synthesis of **26a** from **25**. The crude reaction product was purified by flash chromatography (hexane/EtOAc, 1:1 1:5) to give the desired compound **26i** (100 mg, 65%) as a white solid. R_f 0.79 (EtOAc); ^{1}H NMR (300 MHz, CDCl$_3$): δ 1.82 (1 H, t, $J_{3ax,4}$ ≈ $J_{3ax,3eq}$ 12.6 Hz, H-3ax), 1.89 (3 H, s, NHCOC*H*$_3$), 2.07, 2.12, 2.15 (3 x 3 H, 3 x s, 3 x OCOC*H*$_3$), 2.60 (1 H, dd, $J_{3eq,3ax}$ 12.9 Hz, $J_{3eq,4}$ 4.2 Hz, H-3eq), 3.35 (3 H, s, OC*H*$_3$), 3.84 (3 H, s, CO$_2$C*H*$_3$), 4.10-4.21 (3 H, m, H-5, H-6 and H-9A), 4.80-4.85 (2 H, m, H-4 and H-9B), 5.32-5.34 (1 H, m, H-8), 5.52 (1 H, d, *J* 4.2 Hz, H-7), 5.87 (1 H, d, *J* 8.7 Hz, N*H*COCH$_3$), 6.16 (1 H, d, *J* 9.0 Hz, N*H*CO) 7.40-7.58 (4 H, m, Ar*H*), 7.85-7.93 (2 H, m, Ar*H*), 8.26 (1 H, d, *J* 8.7 Hz, Ar*H*); ^{13}C NMR (75.5 MHz, CDCl$_3$): δ 20.8, 21.0, 21.1 (3 x OCO*C*H$_3$), 23.0 (NHCO*C*H$_3$), 38.3 (C-3), 47.3 (C-4), 49.3 (C-5), 51.1 (O*C*H$_3$), 52.8 (CO$_2$*C*H$_3$), 62.4 (C-9), 68.5 (C-7), 71.6 (C-8), 71.9 (C-6), 98.5 (C-2), 124.7, 125.0, 125.1, 126.5, 127.2, 128.4 (6 x Ar*C*), 129.9 (Ar*C*$_q$), 131.1 (Ar*C*), 133.1, 133.6 (2 x Ar*C*$_q$), 167.8 (C-1), 169.9, 170.6, 170.7, 171.8 (NH*C*O, 3 x O*C*OCH$_3$, NH*C*OCH$_3$); LRMS (ESI): *m/z* 639.2 [(M+Na)$^+$ 100%]; HRMS (ESI): calcd for $C_{30}H_{37}N_2O_{12}$ [M+H]$^+$ 617.2341, found 617.2344.

General procedure for base catalyzed de-acetylation and de-esterification of compounds 26a-i.

To a solution of protected C-4 amide (**26a-i**) in MeOH:H$_2$O (1:1) at 0 °C, was added aq LiOH (2.0 M) to pH 13. The reaction mixture was stirred at rt and monitored by TLC (hexane/EtOAc 1:5). After 16 h, the reaction mixture was acidified to pH 8 with Amberlite® IR-120 (H$^+$) resin, the resin was filtered-off and the filtrate evaporated under reduced pressure to afford yellow syrup. The crude product was purified by column chromatography (EtOAc/MeOH/H$_2$O, 7:2:1) to give a solid residue, which was further purified by RP-HPLC and then lyophilized.

Methyl 5-acetamido-4-butanamido-3,4,5-trideoxy-D-*glycero*-β-D-*galacto*-non-2-ulopyranosidonic acid, lithium salt (27a).

Prepared from **26a** (0.12 g, 0.23 mmol), according to the general procedure for de-esterification (83 mg, 90% yield). HPLC: 3:97 CH$_3$CN/H$_2$O; retention time 6.5-7.5 min; ^{1}H NMR (300 MHz, D$_2$O): δ 0.87 (3 H, app.t, *J* 7.5 Hz, H-c), 1.57 (2 H, q, *J* 7.5 Hz, H-b), 1.69 (1 H, t, $J_{3ax,3eq}$ 13.5 Hz, $J_{3ax,4}$ 12.3 Hz, H-3ax), 1.98 (3 H, s, NHCOC*H*$_3$), 2.15-2.22 (3 H, m, H-3eq and H-a), 3.24 (3 H, s, OC*H*$_3$), 3.55 (1 H, d, *J* 9.9 Hz, H-7), 3.66 (1 H, dd, $J_{9A,9B}$ 12.0 Hz, $J_{9A,8}$ 6.0 Hz, H-9A), 3.83-4.00 (4 H, m, H-5, H-6, H-8 and H-9B), 4.28-4.37 (1 H, m, H-4); ^{13}C NMR (75.5 MHz, D$_2$O): δ 12.6 (C-c), 19.0 (C-b), 22.0 (NHCO*C*H$_3$), 37.5 (C-a), 37.8 (C-3), 46.4 (C-4), 49.5 (C-5), 50.4 (O*C*H$_3$), 63.4 (C-9), 68.4 (C-7), 69.9 (C-8*), 70.3 (C-6*), 99.6 (C-2), 174.2, 175.3, 176.8 (C-1, NH*C*O, NH*C*OCH$_3$); LRMS (ESI): *m/z* 395.1 [(M-3H)$^-$ 100%].

Methyl 5-acetamido-3,4,5-trideoxy-4-hexanamido-D-*glycero*-β-D-*galacto*-non-2-ulopyranosidonic acid, lithium salt (27b).

Prepared from **26b** (0.09 g, 0.16 mmol), according to the general procedure for de-esterification (60 mg, 89% yield). HPLC: 0.4:99.6 CH$_3$CN/H$_2$O; retention time 13.5-14.5 min; ^{1}H NMR (300 MHz, D$_2$O): δ 0.87 (3 H, app.t, *J* 7.2 Hz, H-e), 1.17-1.37 (4 H, m, 2 x C*H*$_2$), 1.49-1.57 (2 H, p, *J* 7.2 Hz, C*H*$_2$), 1.69 (1 H, t, $J_{3ax,3eq}$ 13.2 Hz, $J_{3ax,4}$ 12.6 Hz, H-3ax), 1.99 (3 H, s, NHCOC*H*$_3$), 2.16-2.22 (3 H, m, H-3eq and C*H*$_2$), 3.24 (3 H, s, OC*H*$_3$), 3.54 (1 H, d, *J* 9.9 Hz, H-7), 3.66 (1 H, dd, $J_{9A,9B}$ 12.3 Hz, $J_{9A,8}$ 6.0 Hz, H-9A), 3.83-3.99 (4 H, m, H-5, H-6, H-8 and H-9B), 4.27-4.36 (1 H, m, H-4); ^{13}C NMR (75.5 MHz, D$_2$O): δ 13.1 (C-e), 21.6 (*C*H$_2$), 22.0 (NHCO*C*H$_3$), 25.0 (*C*H$_2$), 30.2 (*C*H$_2$), 35.9 (*C*H$_2$), 37.5 (C-3), 46.4 (C-4), 49.6 (C-5), 50.4 (O*C*H$_3$), 63.4 (C-9), 68.4 (C-7), 69.9 (C-8*), 70.3 (C-6*), 99.6 (C-2), 174.1, 175.3, 177.0 (C-1, NH*C*O, NH*C*OCH$_3$); LRMS (ESI): *m/z* 419.3 [(M-Li)$^-$ 100%].

Methyl 5-acetamido-3,4,5-trideoxy-4-(2-ethyl-butanamido)-D-*glycero*-β-D-*galacto*-non-2-ulopyranosidonic acid, lithium salt (27c).

Prepared from **26c** (0.14 g, 0.24 mmol), according to the general procedure for de-esterification (92 mg, 92% yield). HPLC: 0.1:99.9 CH$_3$CN/H$_2$O; retention time 14.0-15.5 min; ^{1}H NMR (300 MHz, D$_2$O): δ 0.80 and 0.82 (2 x 3 H, 2 x t, *J* 7.5 Hz, H-c and H-c'), 1.41-1.51 (4 H, m, H-b and H-b'), 1.71 (1 H, t, $J_{3ax,3eq}$ 13.2 Hz, $J_{3ax,4}$ 12.3 Hz, H-3ax), 1.98 (3 H, s, NHCOC*H*$_3$), 2.00-2.06 (1H, m, H-a), 2.20 (1 H, dd, $J_{3eq,3ax}$ 13.2 Hz, $J_{3eq,4}$ 4.8, H-3eq), 3.24 (3 H, s, OC*H*$_3$), 3.54 (1 H, dd, $J_{7,8}$ 9.3 Hz, $J_{7,6}$ 0.6 Hz, H-7), 3.65 (1 H, dd, $J_{9A,9B}$ 12.3 Hz, $J_{9A,8}$ 6.0 Hz, H-9A), 3.83-3.95 (3 H, m, H-6, H-8 and H-9B), 3.97 (1 H, app.q, *J* 10.5 Hz, H-5), 4.32-4.41 (1 H, m, H-4); ^{13}C NMR (75.5 MHz, D$_2$O): δ 11.2 (C-c and C-c'), 22.1 (NHCO*C*H$_3$), 25.2 and 25.4 (C-b and C-b'), 37.8 (C-3), 46.2 (C-4), 49.1 (C-5), 50.5 (O*C*H$_3$), 50.8 (C-a), 63.5 (C-9), 68.4 (C-7), 69.9 (C-8*), 70.4 (C-6*), 99.6 (C-2), 174.1, 175.2 and 179.3 (C-1, NH*C*O, NH*C*OCH$_3$); LRMS (ESI): *m/z* 419.4 [(M-Li)$^-$ 100%].

Methyl 5-acetamido-3,4,5-trideoxy-4-(2-ethyl-hexanamido)-D-*glycero*-β-D-*galacto*-non-2-ulopyranosidonic acid, lithium salt (27d).

Prepared from **26d** (0.16 g, 0.29 mmol), according to the general procedure for de-esterification (115 mg, 90% yield). HPLC: 5.5:94.5 CH_3CN/H_2O; retention time 15.0-16.7 min; ^1H NMR (300 MHz, D_2O): δ 0.77-0.88 [(6 H, m, H-e (Hex) and H-g (Et)], 1.10-1.32 (4 H, m, H-d and H-f), 1.41-1.51 (4 H, m, H-b and H-c), 1.70, 1.70† (1 H, t, $J_{3ax,4}$ ≈ $J_{3ax,3eq}$ 12.3 Hz, H-3ax), 1.98, 2.00† (3 H, s, NHCOC*H*3), 2.08-2.15 (1 H, m, H-a), 2.16, 2.21† [1 H, dd, $J_{3ax,4}$ 3.9 Hz ($J_{3eq,4}$, not assigned) H-3eq], 3.24 (3 H, s, OC*H*3), 3.53 (1 H, d, *J* 9.6 Hz, H-7), 3.65 (1 H, dd, $J_{9A,9B}$ 12.3 Hz, $J_{9A,8}$ 6.0 Hz, H-9A), 3.83-4.02 (4 H, m, H-5, H-6, H-8 and H-9B), 4.31-4.41 (1 H, m, H-4); ^{13}C NMR (75.5 MHz, D_2O): δ 11.1 [C-e* (Hex)], 13.1 (C-g*, (Et)], 21.8, 21.8, 22.1 (C-b*), 22.2 (NHCO*C*H3), 25.5, 25.7† (C-d*), 28.9, 29.0† (C-c*), 31.6, 31.7† (C-f*), 37.9 (C-3), 46.1 (C-4), 48.9 (C-a*), 49.1 (C-5*), 50.4 (O*C*H3), 63.4 (C-9), 68.4 (C-7), 69.8 (C-8*), 70.4, 70.5† (C-6*), 99.6 (C-2), 174.0, 174.1†, 175.2, 179.4, 179.4† (C-1, NH*C*O, NH*C*OCH3), [†Assignment belongs to corresponding diastereomer]; LRMS (ESI): *m/z* 447.1 [(M-Li)⁻ 100%].

Methyl 5-acetamido-3,4,5-trideoxy-4-(3-nitro-benzamido)-D-*glycero*-β-D-*galacto*-non-2-ulopyranosidonic acid, lithium salt (27e).

Prepared from **26e** (0.17 g, 0.28 mmol), according to the general procedure for de-esterification (120 mg, 92% yield). HPLC: 0.4:99.6 CH_3CN/H_2O; retention time 13.8-15.0 min; ^1H NMR (300 MHz, D_2O): δ 1.88 (1 H, t, $J_{3ax,4}$ ≈ $J_{3ax,3eq}$ 13.2 Hz, H-3ax), 1.92 (3 H, s, NHCOC*H*3), 2.33 (1 H, dd, $J_{3eq,3ax}$ 13.2 Hz, $J_{3eq,4}$ 4.8 Hz, H-3eq), 3.29 (3 H, s, OC*H*3), 3.63 (1 H, dd, $J_{7,8}$ 9.3 Hz, $J_{7,6}$ 1.2 Hz, H-7), 3.69 (1 H, dd, $J_{9A,9B}$ 12.0 Hz, $J_{9A,8}$ 6.0 Hz, H-9A), 3.86-3.97 (2 H, m, H-8 and H-9B), 4.03 (1 H, dd, $J_{6,5}$ 10.5 Hz, $J_{6,7}$ 1.2 Hz, H-6), 4.15 (1 H, app.t, *J* 10.5 Hz, H-5), 4.55-4.64 (1 H, m, H-4), 7.74 (1 H, t, *J* 8.1, Ar*H*), 8.04-8.08 (1 H, m, Ar*H*), 8.41-8.44 (1 H, m, Ar*H*), 8.51-8.53 (1 H, m, Ar*H*); ^{13}C NMR (75.5 MHz, D_2O): δ 21.8 (NHCO*C*H3), 37.1 (C-3), 47.8 (C-4), 49.8 (C-5), 50.5 (O*C*H3), 63.5 (C-9), 68.4 (C-7), 69.9 (C-8), 70.2 (C-6), 99.6 (C-2), 122.2, 126.5, 130.2, and 133.3 (4 x Ar*C*), 135.1 (ArC_q), 147.8 (ArC_q), 168.5, 174.3 and 175.3 (C-1, NH*C*O, NH*C*OCH3); LRMS (ESI): *m/z* 470.0 [(M-Li)⁻ 100%].

Methyl 5-acetamido-3,4,5-trideoxy-4-(3-phenyl-propanamido)-D-*glycero*-β-D-*galacto*-non-2-ulopyranosidonic acid, lithium salt (27f).

Prepared from **26f** (0.16 g, 0.27 mmol), according to the general procedure for de-esterification (117 mg, 95% yield). HPLC: 2.5:97.5 CH_3CN/H_2O; retention time 12.0-13.5 min; ^1H NMR (300 MHz, D_2O): δ 1.47 (1 H, t, $J_{3ax,3eq}$ 13.5 Hz, $J_{3ax,4}$ 12.6 Hz, H-3ax), 1.89 (3 H, s, NHCOC*H*3), 1.98 (1 H, dd, $J_{3eq,3ax}$ 13.5 Hz, $J_{3eq,4}$ 4.8 Hz, H-3eq), 2.44-2.59 (2 H, m, C*H*2), 2.82-2.97 (2 H, m, C*H*2), 3.22 (3 H, s, OC*H*3), 3.51 (1 H, d, *J* 9.6 Hz, H-7), 3.64 (1 H, dd, $J_{9A,9B}$ 12.3 Hz, $J_{9A,8}$ 6.0 Hz, H-9A), 3.82-3.91 (4 H, m, H-5, H-6, H-8 and H-9B), 4.13-4.27 (1 H, m, H-4), 7.23-7.30 (3 H, m, Ar*H*), 7.33-7.39 (2 H, m, Ar*H*); ^{13}C NMR (75.5 MHz, D_2O): δ 21.9 (NHCO*C*H3), 31.2 (C-a), 37.3 (C-3, C-b), 46.5 (C-4), 49.3 (C-5), 50.4 (O*C*H3), 63.4 (C-9), 68.4 (C-7), 69.8 (C-8*), 70.2 (C-6*), 99.5 (C-2), 126.5, 128.3, and 128.7 (5 x Ar*C*), 140.3 (*ipso*-Ar*C*), 174.2, 175.2, 175.4 (C-1, NH*C*O, NH*C*OCH3); LRMS (ESI): *m/z* 458.1 [(M-2H)⁻ 100%].

Methyl 5-acetamido-3,4,5-trideoxy-4-(4-phenoxy-butanamido)-D-*glycero*-β-D-*galacto*-non-2-ulopyranosidonic acid, lithium salt (27g).

Prepared from **26g** (0.17 g, 0.27 mmol), according to the general procedure for de-esterification (112 mg, 85% yield). HPLC: 4:96 CH_3CN/H_2O; retention time 13.5-14.5 min; ^1H NMR (300 MHz, D_2O): δ 1.59 (1 H, t, $J_{3ax,4}$ ≈ $J_{3ax,3eq}$ 12.9 Hz, H-3ax), 1.90 (3 H, s, NHCOC*H*3), 1.99-2.06 (1 H, m, H-b), 2.12 (1 H, dd, $J_{3eq,3ax}$ 13.5 Hz, $J_{3eq,4}$ 4.8 Hz, H-3eq), 2.39 (2 H, t, *J* 7.5 Hz, H-a), 3.24 (3 H, s, OC*H*3), 3.54 (1 H, d, *J* 9.6 Hz, H-7), 3.65 (1 H, dd, $J_{9A,9B}$ 12.3 Hz, $J_{9A,8}$ 6.0, H-9A), 3.83-3.97 (4 H, m, H-5, H-6, H-8 and H-9B), 4.05 (2 H, d, *J* 6.3 Hz, H-c) 4.26-4.37 (1 H, m, H-4), 7.00-7.08 (3 H, m, Ar*H*), 7.36-7.41 (2 H, m, Ar*H*); ^{13}C NMR (75.5 MHz, D_2O): δ 21.8 (NHCO*C*H3), 24.8 (C-b), 32.4 (C-a), 37.3 (C-3), 46.6 (C-4), 49.5 (C-5), 50.4 (O*C*H3), 63.4 (C-9), 67.1 (C-c), 68.4 (C-7), 69.8 (C-8*), 70.2 (C-6*), 99.6 (C-2), 114.8, 121.4, and 129.8 (5 x Ar*C*), 157.9 (*ipso*-Ar*C*), 174.2, 175.2, 175.7 (C-1, NH*C*O, NH*C*OCH3); LRMS (ESI): *m/z* 483.4 [(M-Li)⁻ 100%].

Methyl 5-acetamido-3,4,5-trideoxy-4-(4-phenyl-benzamido)-D-*glycero*-β-D-*galacto*-non-2-ulopyranosidonic acid, lithium salt (27h).

Prepared from **26h** (0.16 g, 0.25 mmol), according to the general procedure for de-esterification (110 mg, 88% yield). HPLC: 10:90 CH_3CN/H_2O; retention time 16.5-15.0 min; ^1H NMR (300 MHz, D_2O): δ 1.80 (1 H, t, $J_{3ax,4}$ ≈ $J_{3ax,3eq}$ 12.9 Hz, H-3ax), 1.87 (3 H, s, NHCOC*H*3), 2.28 (1 H, dd, $J_{3eq,3ax}$ 13.2 Hz, $J_{3eq,4}$ 4.5 Hz, H-3eq), 3.30 (3 H, s, OC*H*3), 3.62 (1 H, d, *J* 10.2 Hz, H-7), 3.65 (1 H, dd, $J_{9A,9B}$ 11.7 Hz, $J_{9A,8}$ 5.7 Hz, H-9A), 3.86-3.97 (2 H, m, H-8 and H-9B), 4.00 (1 H, dd, $J_{6,5}$ 10.2 Hz, $J_{6,7}$ 0.9 Hz, H-6), 4.13 (1 H, app.t, *J* 10.5 Hz, H-5), 4.50-4.59 (1 H, m, H-4), 7.33-7.42 (3 H, m, Ar*H*), 7.46-7.51 (4 H, m, Ar*H*), 7.62 (2 H, m, Ar*H*); ^{13}C NMR (75.5 MHz, D_2O): δ 21.8 (NHCO*C*H3), 37.2 (C-3), 47.6 (C-4), 49.6 (C-5), 50.5 (O*C*H3), 63.5 (C-9), 68.4 (C-7), 69.9 (C-8),

70.3 (C-6), 126.9, 127.5, 128.2, and 129.0 (9 x Ar*C*), 131.9, 139.1, and 143.9 (3 x ArC_q), 170.0, 174.2 and 175.1 (NH*C*OCH_3, NH*C*O, C-1), [C-2 was not observed]; LRMS (ESI): *m/z* 501.6 [$(M-Li)^-$ 100%].

Methyl 5-acetamido-3,4,5-trideoxy-4-(1-naphthylcarboxamido)-D-*glycero*-β-D-*galacto*-non-2-ulopyranosidonic acid, lithium salt (27i).

Prepared from **26i** (0.10 g, 0.16 mmol), according to the general procedure for de-esterification (70 mg, 90% yield). HPLC: 5:95 CH_3CN/H_2O; retention time 13.0-14.7 min; ^1H NMR (300 MHz, D_2O): δ 1.76 (1 H, t, $J_{3ax,4} \approx J_{3ax,3eq}$ 12.9 Hz, H-3ax), 1.98 (3 H, s, NHCOC*H*3), 2.33 (1 H, dd, $J_{3eq,3ax}$ 13.2 Hz, $J_{3eq,4}$ 4.5 Hz, H-3eq), 3.22 (3 H, s, OC*H*3), 3.60 (1 H, d, *J* 9.6 Hz, H-7), 3.62 (1 H, dd, $J_{9A,9B}$ 11.7 Hz, $J_{9A,8}$ 6.0 Hz, H-9A), 3.77-3.89 (2 H, m, H-8 and H-9B), 3.95 (1 H, dd, *J* 10.5 Hz, H-6), 4.04 (1 H, app.t, *J* 10.5 Hz, H-5), 4.56-4.65 (1 H, m, H-4), 7.46-7.58 (4 H, m, Ar*H*), 7.91-8.00 (3 H, m, Ar*H*); ^{13}C NMR (75.5 MHz, D_2O): δ 22.1 (NHCO*C*H3), 37.4 (C-3), 47.2 (C-4), 49.8 (C-5), 50.5 (O*C*H3), 63.5 (C-9), 68.4 (C-7), 69.9 (C-8), 70.4 (C-6), 99.7 (C-2), 124.2, 125.1, 125.2, 126.7, 127.3, 128.4 (6 x Ar*C*), 129.1 (ArC_q), 130.8 (Ar*C*), 132.9, 133.2 (2 x ArC_q), 172.2, 174.2, 175.3 (C-1, NH*C*O, NH*C*OCH_3); LRMS (ESI): *m/z* 475.1 [$(M-Li)^-$ 100%].

References:

1. Chopra, P. **2013**. The Development of Carbohydrate-based Chemical Probes of CMP-*N*-Acetylneuraminate Synthetase. Doctoral dissertation, Griffith Univeristy, Queensland, Australia. Available online: http://hdl.handle.net/10072/366420 (accessed on 14 July 2025).
2. Chandler, M.; Bamford, M. J.; Conroy, R.; Lamont, B.; Patel, B.; Patel, V. K.; Steeples, I. P.; Storer, R.; Weir, N. G.; Wright, M.; et al. Synthesis of the potent influenza neuraminidase inhibitor 4-guanidino-Neu5Ac2en. X-Ray molecular structure of 5-acetamido-4-amino-2,6-anhydro-3,4,5-trideoxy-D-*erythro*-L-*gluco*-nononic acid. *J. Chem. Soc., Perkin Trans. 1* **1995**, 1173-1180. https://doi.org/10.1039/P19950001173
3. Ciccotosto, S.; von Itzstein, M. Synthesis of methyl 5-acetamido-3,4,5-trideoxy-4-guanidinyl-D-*glycero*-D-*galacto*-2-nonulopyranosidonic acid (4-deoxy-4-guanidino-Neu5Acα2Me). *Tetrahedron Lett.* **1995**, *36*, 5405-5408. https://doi.org/10.1016/0040-4039(95)00991-K
4. Kuhn, R.; Lutz, P.; MacDonald, D. L. Syntheses anomerer sialinsaure-methylketoside. *Chem. Ber.* **1966**, *99*, 611-617. https://doi.org/10.1002/cber.19660990235
5. Chopra, P.; Thomson, R. J.; Grice, I. D.; von Itzstein, M. Rapid and clean microwave-assisted synthesis of *N*-acetylneuraminic acid methyl ester and its β-methyl glycoside. *Tetrahedron Lett.* **2012**, *53*, 6254-6256. https://doi.org/10.1016/j.tetlet.2012.09.017
6. Schmid, W.; Christian, R.; Zbiral, E. Synthesis of both epimeric 2-deoxy-*N*-acetylneuraminic acids and their behaviour towards CMP-sialate synthetase - A comparison with 2-β-methylketoside of *N*-acetylneuraminic acid. *Tetrahedron Lett.* **1988**, *29*, 3643-3646. https://doi.org/10.1016/S0040-4039(00)82143-7
7. Byramova, N. E.; Tuzikov, A. B.; Bovin, N. V. A simple procedure for the synthesis of the methyl and benzyl glycosides of Neu5Ac and 4-*epi*-Neu5Ac. Conversion of the benzyl and methyl glycosides of Neu5Ac into *N*-trifluoroacetylneuraminic acid benzyl glycosides. *Carbohydr. Res.* **1992**, *237*, 161-175. https://doi.org/10.1016/s0008-6215(92)84240-s
8. Czarniecki, M. F.; Thornton, E. R. Carbon-13 nuclear magnetic resonance spin-lattice relaxation in the *N*-acylneuraminic acids. Probes for internal dynamics and conformational analysis. *J. Am. Chem. Soc.* **1977**, *99*, 8273. https://doi.org/10.1021/ja00467a025
9. Kiefel, M. J.; Chopra, P.; Madge, P. D.; Szyczew, A.; Thomson, R. J.; Grice, I. D.; von Itzstein, M. Synthesis of C-9 oxidised *N*-acetylneuraminic acid derivatives as biological probes. *Tetrahedron Lett.* **2011**, *52*, 98-100. https://doi.org/10.1016/j.tetlet.2010.10.156
10. Brandstetter, H. H.; Zbiral, E. Strukturelle Abwandlungen an *N*-Acetylneuraminsäure, 2 [Transformations with *N*-acetylneuraminic acid. 2]. *Liebigs Ann. Chem.* **1983**, 2055-65. https://doi.org/10.1002/jlac.198319831202
11. Zbiral, E.; Phadtare, S.; Schmid, W. Strukturelle Abwandlungen an *N*-Acetylneuraminsäure, 6. Synthesen von 7-Oxo- und 8-Oxo-*N*-acetylneuraminsäure-Derivaten. *Liebigs Ann. Chem.* **1987**, *1987*, 39-43. https://doi.org/10.1002/jlac.198719870107
12. Honda, T.; Masuda, T.; Yoshida, S.; Arai, M.; Kaneko, S.; Yamashita, M. Synthesis and anti-Influenza virus activity of 7-*O*-alkylated derivatives related to zanamivir. *Bioorg. Med. Chem. Lett.* **2002**, *12*, 1925-1928. https://doi.org/10.1016/S0960-894X(02)00329-3
13. Masuda, T.; Yoshida, S.; Arai, M.; Kaneko, S.; Yamashita, M.; Honda, T. Synthesis and anti-influenza evaluation of polyvalent sialidase inhibitors bearing 4-guanidino-Neu5Ac2en derivatives. *Chem. Pharm. Bull.* **2003**, *51*, 1386-1398. https://doi.org/10.1248/cpb.51.1386
14. Masuda, T.; Shibuya, S.; Arai, M.; Yoshida, S.; Tomozawa, T.; Ohno, A.; Yamashita, M.; Honda, T. Synthesis and anti-influenza evaluation of orally active bicyclic ether derivatives related to zanamivir. *Bioorg. Med. Chem. Lett.* **2003**, *13*, 669-673. https://doi.org/10.1016/S0960-894X(02)01039-9
15. Chopra, P.; Madge, P. D.; Thomson, R. J.; Grice, I. D.; von Itzstein, M. Microwave-assisted synthesis of *N*-glycolylneuraminic acid derivatives. *Tetrahedron Lett.* **2013**, *54*, 5558-5561. https://doi.org/10.1016/j.tetlet.2013.07.107
