# Supplementary material for: Exploring the Scope of Functionalized N-Acylneuraminic Acid β-Methyl Glycosides as Inhibitors of Neisseria meningitidis CMP-Sialic Acid Synthetase
Source: Molecules. 2025 Nov 7;30(22):4329. doi: 10.3390/molecules30224329 (PMC12654856; doi:10.3390/molecules30224329)

Supporting Information – 2: NMR spectra

Article

Exploring the Scope of Functionalized *N*-Acylneuraminic Acid β-Methyl Glycosides as Inhibitors of *Neisseria meningitidis* CMP-Sialic Acid Synthetase

Pradeep Chopra [1], Jana Führing [2], Preston Ng [1], Thomas Haselhorst [1], Jeffrey C. Dyason [1], Faith J. Rose [1], Robin J. Thomson [1], Rita Gerardy-Schahn [2], I. Darren Grice [1], Michael P. Jennings [1*], Anja K. Münster-Kühnel [2*] and Mark von Itzstein [1*]

[1] Institute for Biomedicine and Glycomics, Griffith University, Gold Coast, Queensland 4222, Australia; pchopra@uga.edu (P.C.); t.haselhorst@griffith.edu.au (T.H.); r.thomson@griffith.edu.au (R.J.T.); d.grice@griffith.edu.au (I.D.G.)

[2] Institut für Klinische Biochemie, Medizinische Hochschule, Carl-Neuberg-Straße 1, 30625 Hannover, Germany; fuehring.jana@mh-hannover.de (J.F.); gerardy-schahn.rita@mh-hannover.de (R.G.-S.)

* Correspondence: m.jennings@griffith.edu.au (M.P.J.); muenster.anja@mh-hannover.de (A.K.M.-K.); m.vonitzstein@griffith.edu.au (M.v.I.)

NMR spectra (^{1}H and ^{13}C) for synthesized compounds

Compounds **4** and **3** S3

Series I – C-9 carboxamide derivatives of Neu5Acβ2Me S5

Series II – C-7 ether derivatives of Neu5Acβ2Me S31

Series III– C-5 amide derivatives of Neu5acylβ2Me S49

Series IV – C-4 amide derivatives of Neu5Acβ2Me S69

^{1}H NMR (CD_3OD): Methyl (methyl 5-acetamido-3,5-dideoxy-D-*glycero*-β-D-*galacto*-non-2-ulopyranosid)onate (**4**)

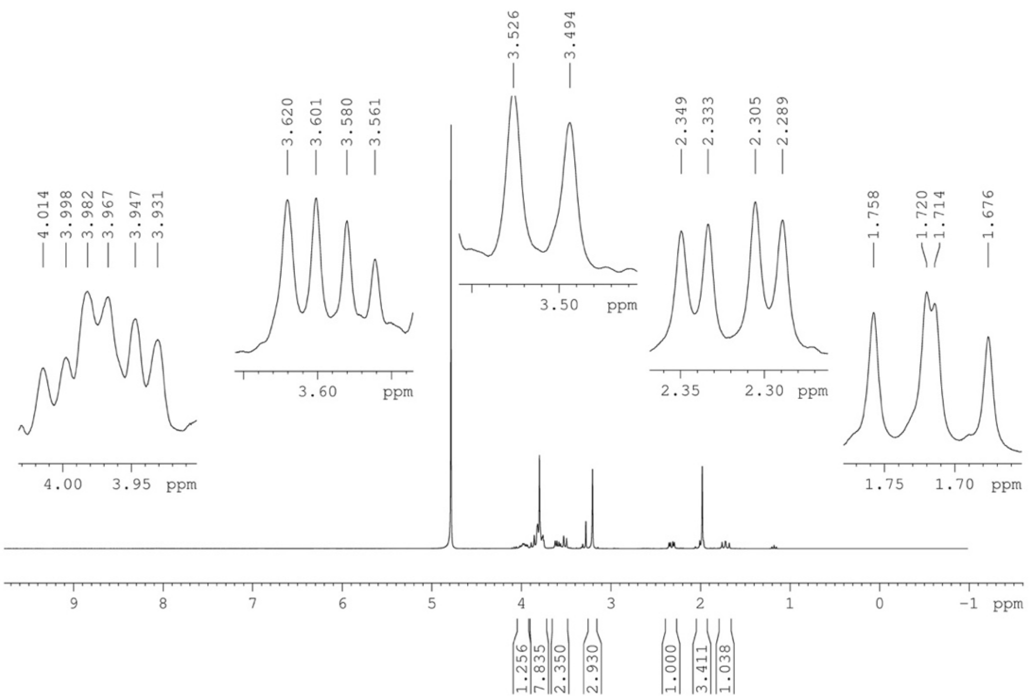

^{13}C NMR (CD_3OD): Methyl (methyl 5-acetamido-3,5-dideoxy-D-*glycero*-β-D-*galacto*-non-2-ulopyranosid)onate (**4**)

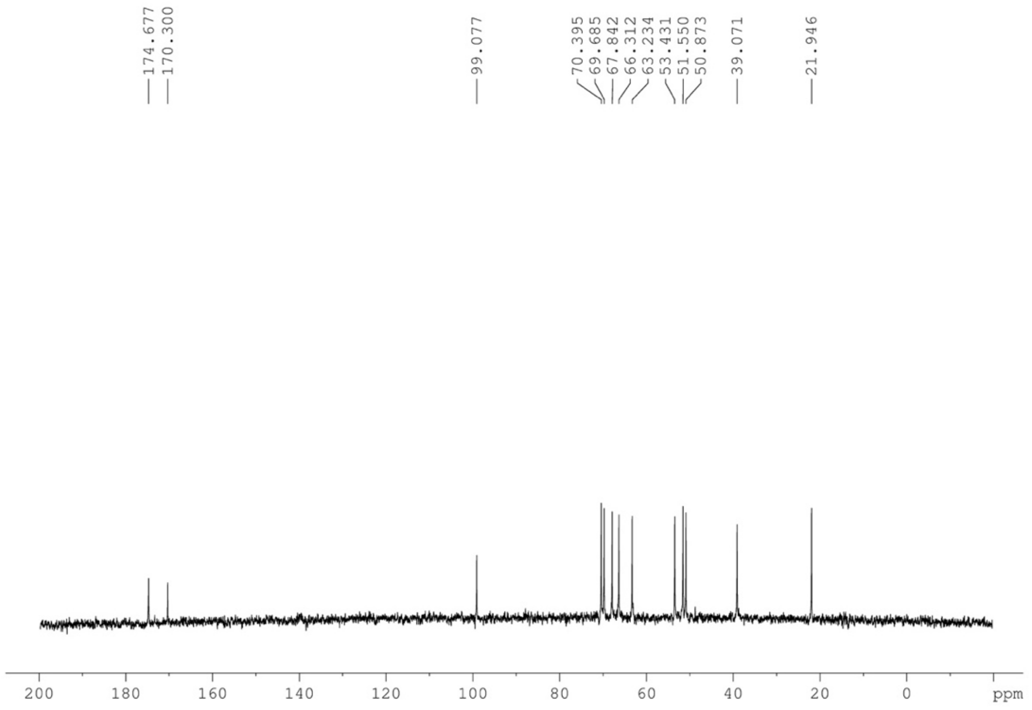

^{1}H NMR (D_2O): Methyl 5-acetamido-3,5-dideoxy-D-*glycero*-β-D-*galacto*-non-2-ulopyranosidonic acid (**3**)

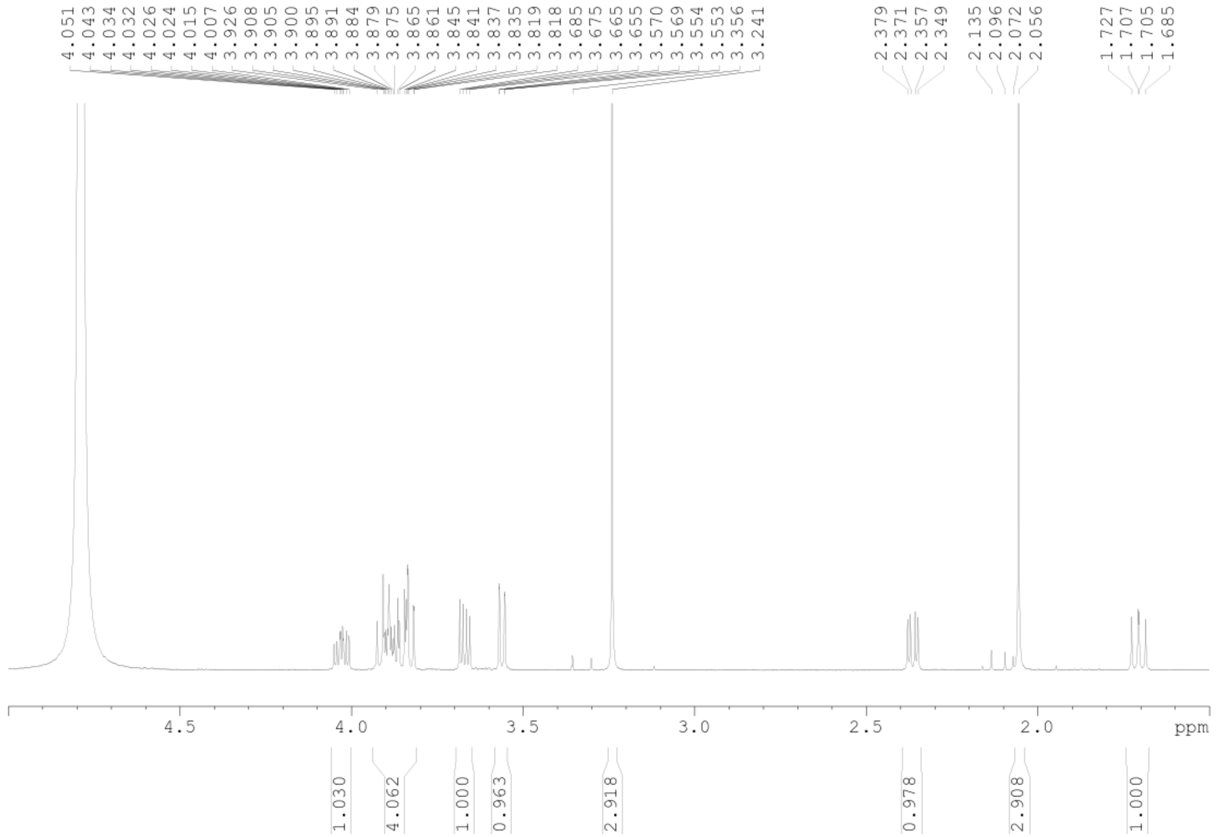

^{13}C NMR (D_2O): Methyl 5-acetamido-3,5-dideoxy-D-*glycero*-β-D-*galacto*-non-2-ulopyranosidonic acid (**3**)

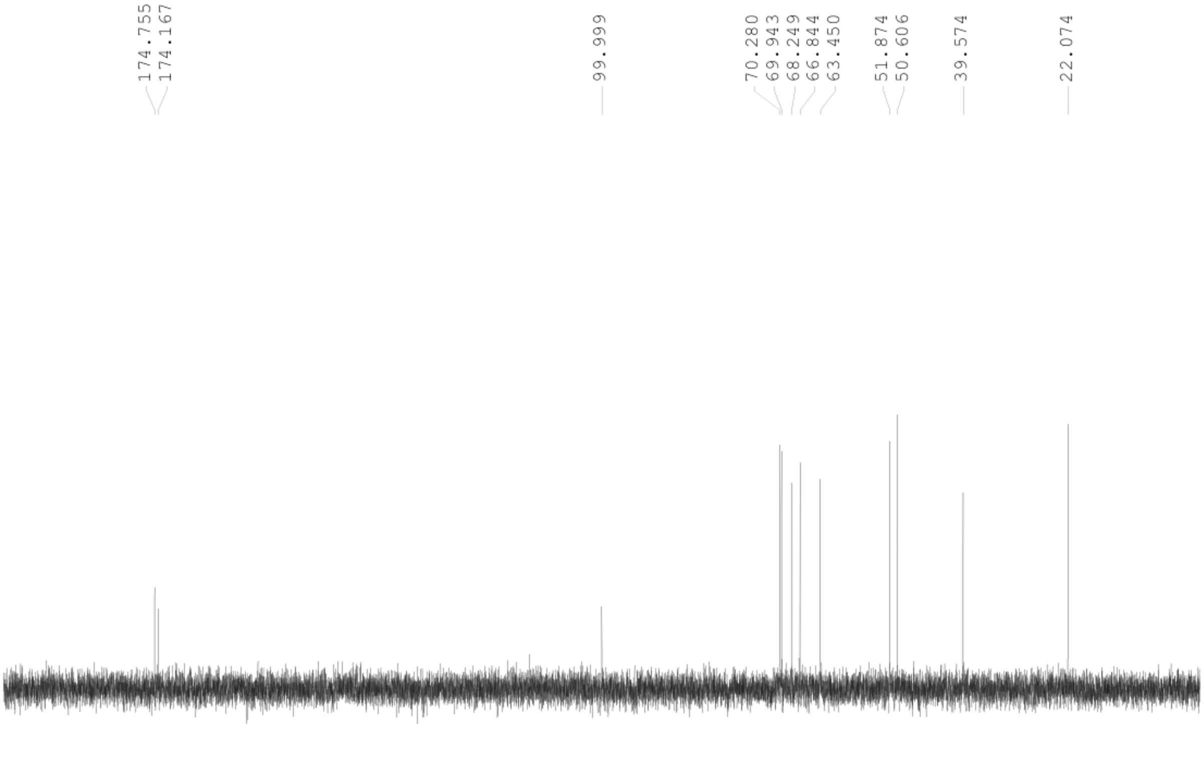

^{1}H NMR (CD_3OD): Methyl (methyl 5-acetamido-4,7,8-tri-*O*-acetyl-8-carboxy-3,5-dideoxy-D-*glycero*-β-D-*galacto*-oct-2-ulopyranosid)onate (**5**)

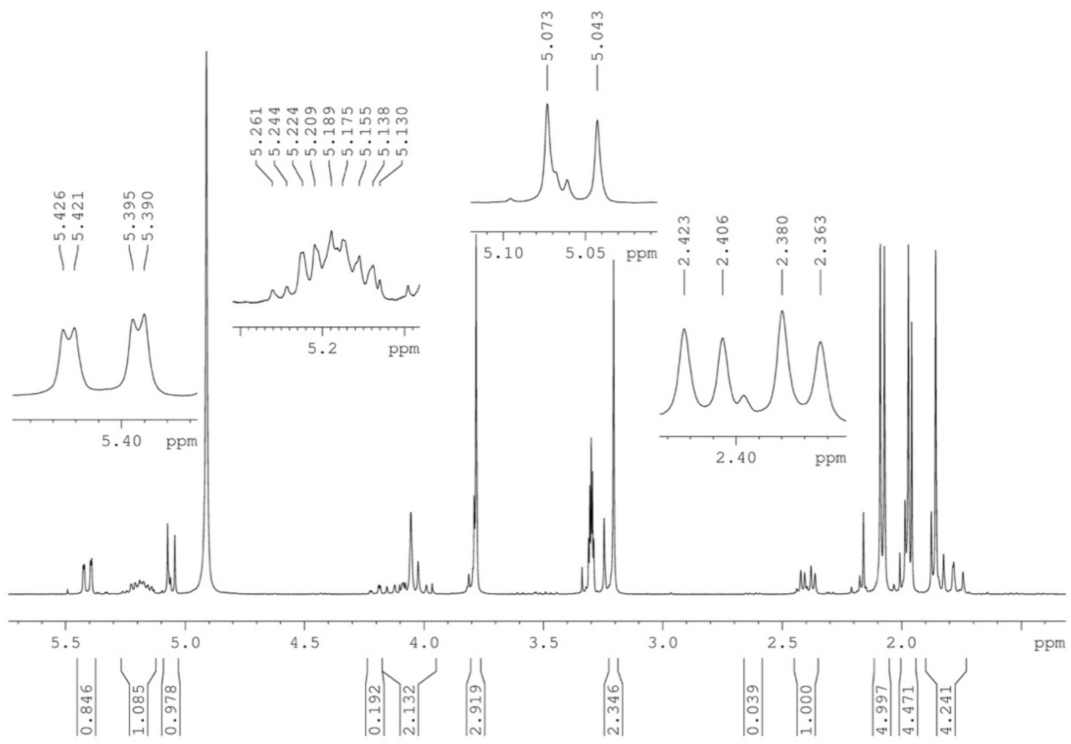

^{13}C NMR (CD_3OD): Methyl (methyl 5-acetamido-4,7,8-tri-*O*-acetyl-8-carboxy-3,5-dideoxy-D-*glycero*-β-D-*galacto*-oct-2-ulopyranosid)onate (**5**)

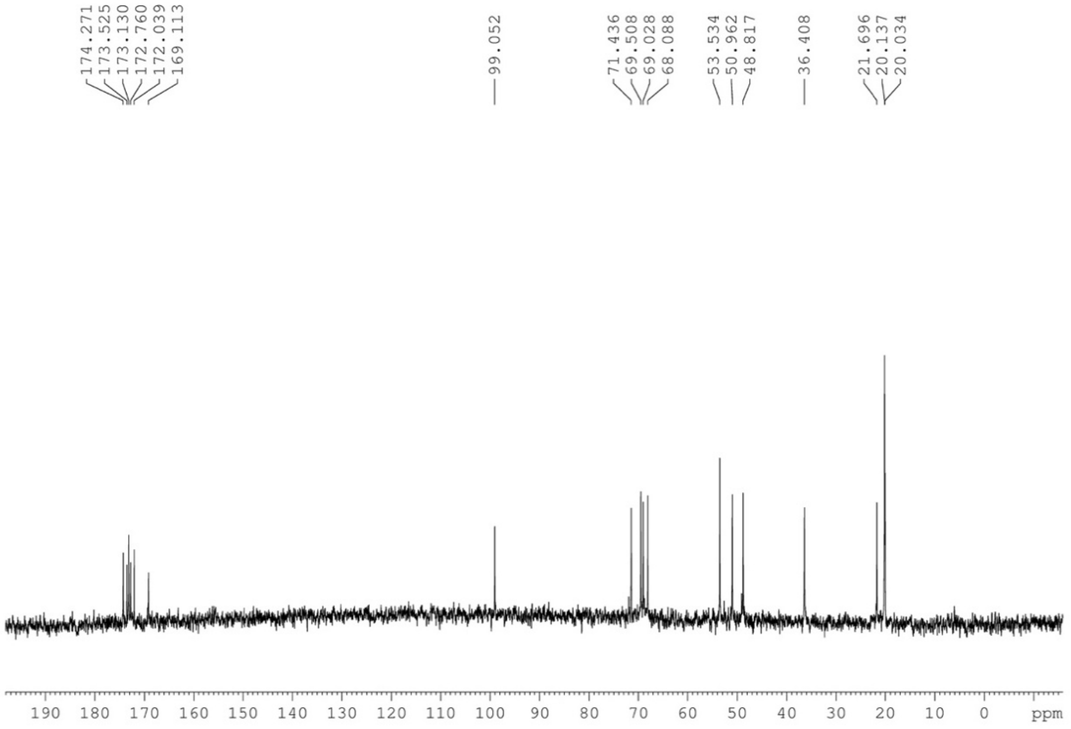

^{1}H NMR ($CDCl_3$): Methyl [methyl 5-acetamido-4,7,8-tri-*O*-acetyl-8-(*N*-butyl)carboxamido-3,5-dideoxy-D-*glycero*-β-D-*galacto*-oct-2-ulopyranosid]onate (**6a**)

[Note: the spectra of **6a** provided may contain a small amount of free butylamine]

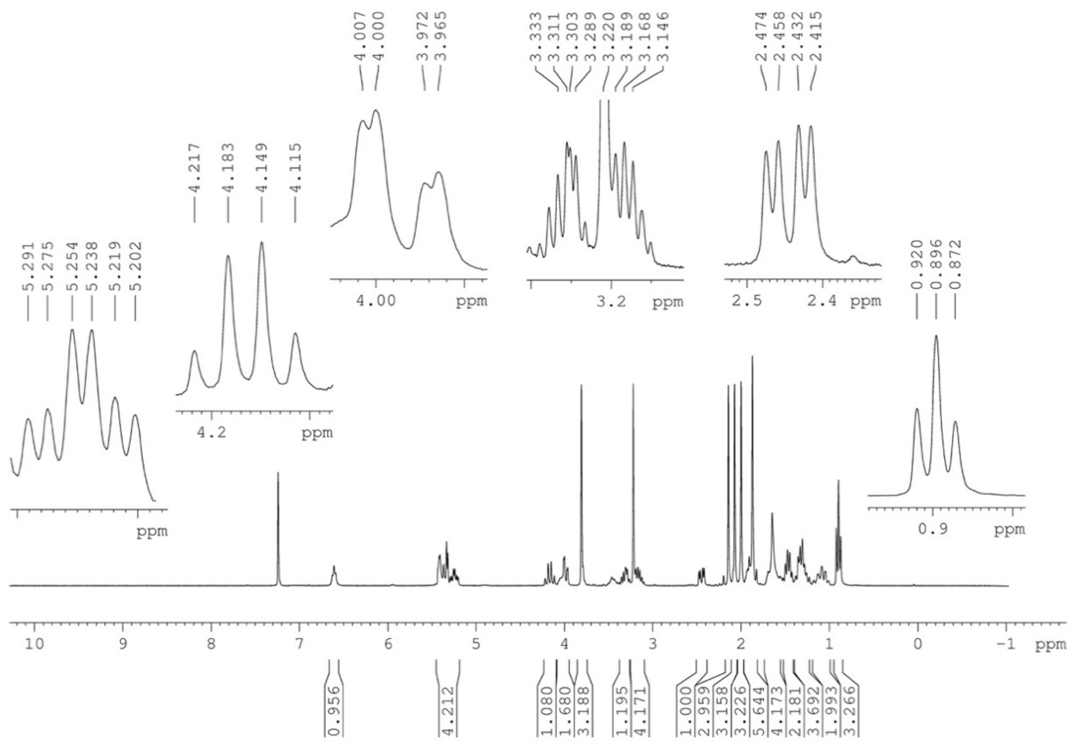

^{13}C NMR ($CDCl_3$): Methyl [methyl 5-acetamido-4,7,8-tri-*O*-acetyl-8-(*N*-butyl)carboxamido-3,5-dideoxy-D-*glycero*-β-D-*galacto*-oct-2-ulopyranosid]onate (**6a**)

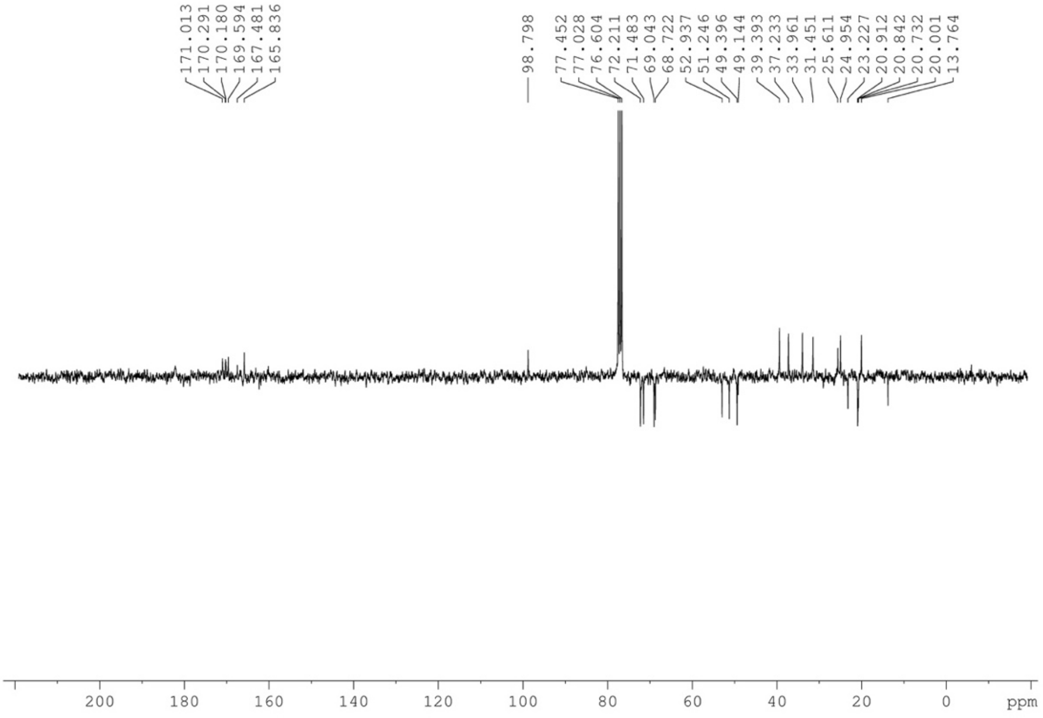

^{1}H NMR ($CDCl_3$): Methyl [methyl 5-acetamido-4,7,8-tri-*O*-acetyl-8-(*N*-cyclohexyl)carboxamido-3,5-dideoxy-D-*glycero*-β-D-*galacto*-oct-2-ulopyranosid]onate (**6b**)

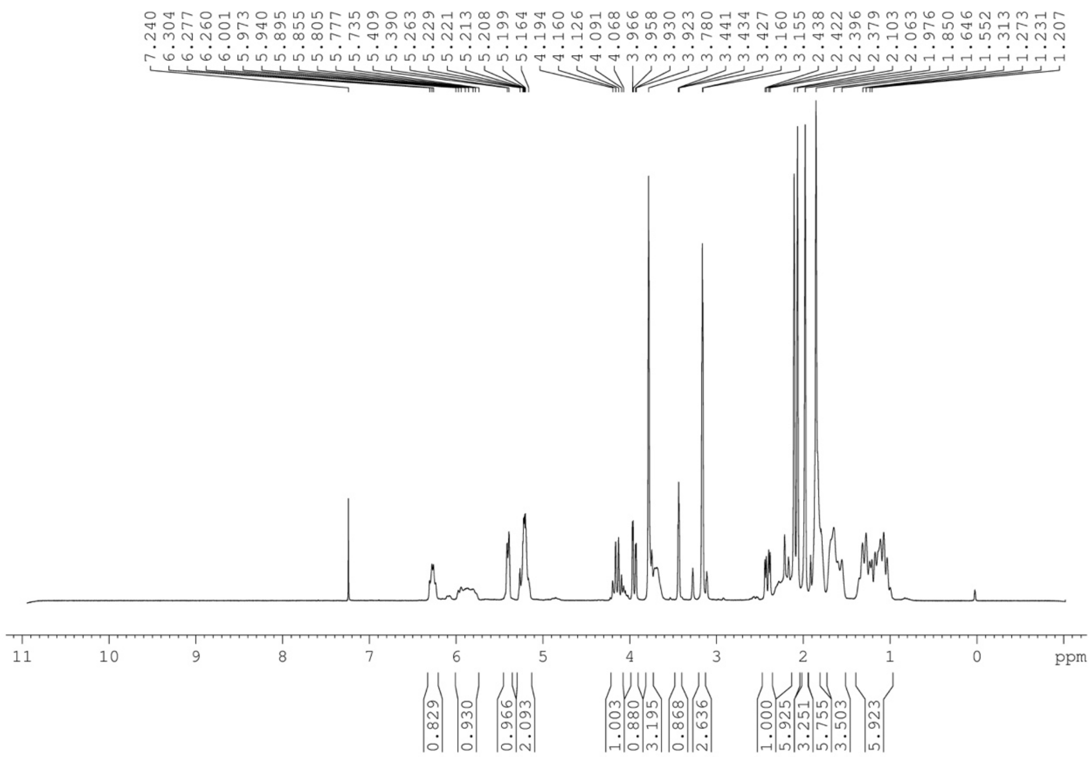

^{13}C NMR ($CDCl_3$): Methyl [methyl 5-acetamido-4,7,8-tri-*O*-acetyl-8-(*N*-cyclohexyl)carboxamido-3,5-dideoxy-D-*glycero*-β-D-*galacto*-oct-2-ulopyranosid]onate (**6b**)

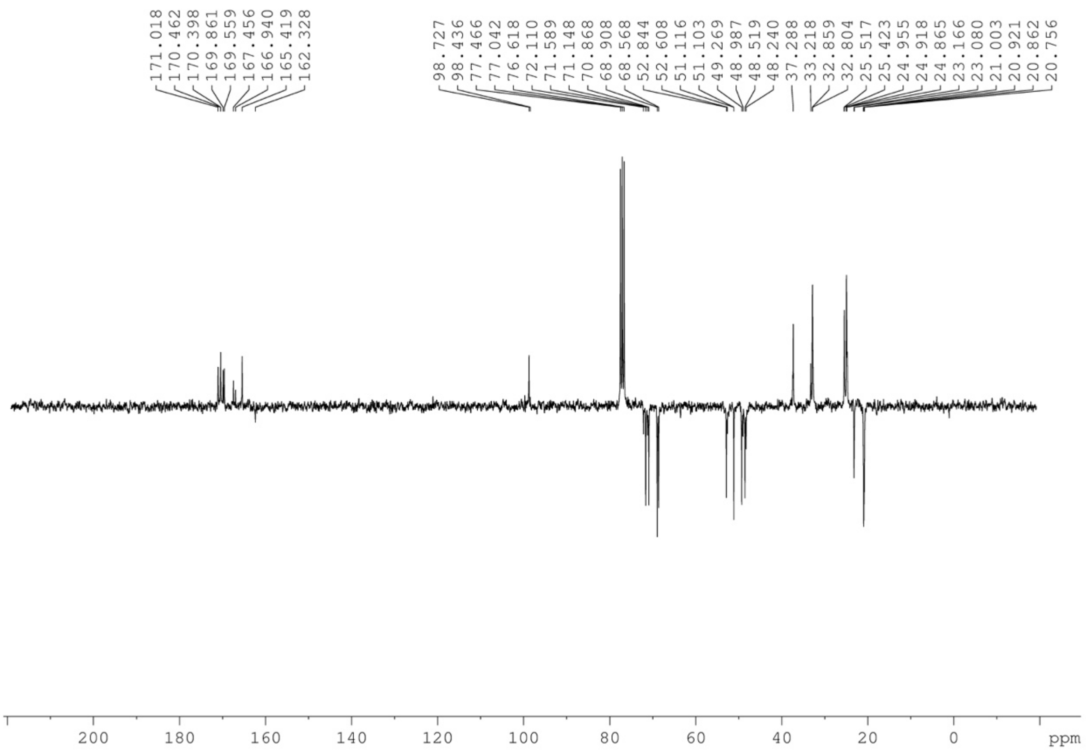

^{1}H NMR ($CDCl_3$): Methyl [methyl 5-acetamido-4,7,8-tri-*O*-acetyl-8-(*N*-benzyl)carboxamido-3,5-dideoxy-D-*glycero*-β-D-*galacto*-oct-2-ulopyranosid]onate (**6c**)

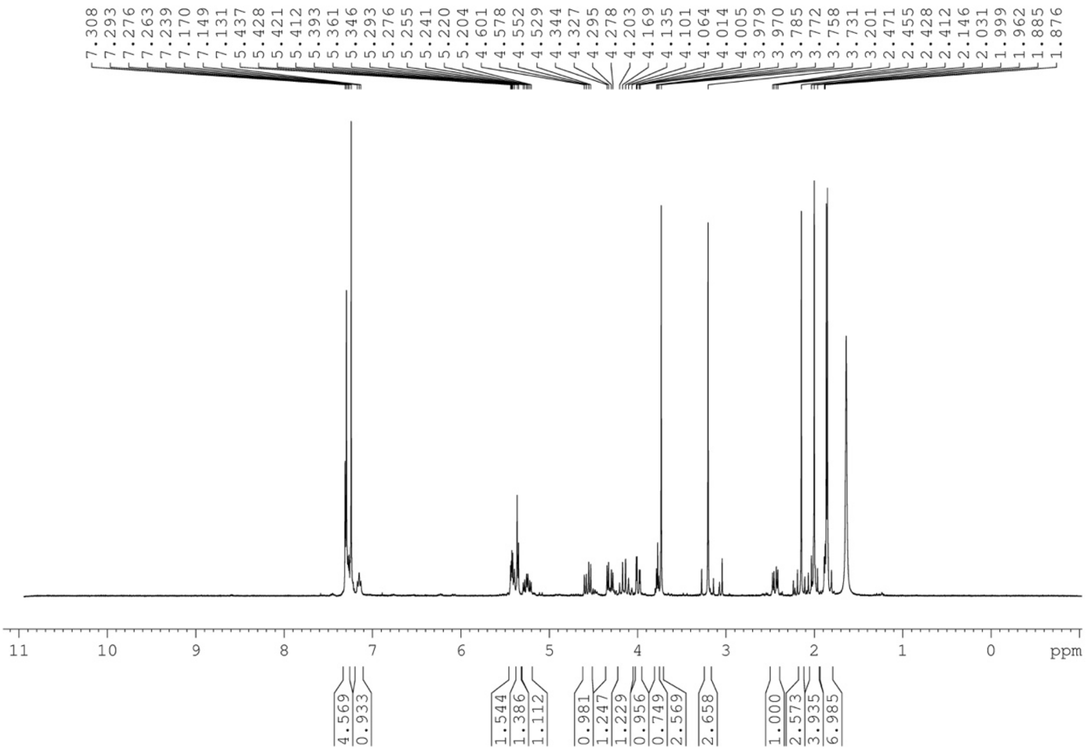

^{13}C NMR ($CDCl_3$): Methyl [methyl 5-acetamido-4,7,8-tri-*O*-acetyl-8-(*N*-benzyl)carboxamido-3,5-dideoxy-D-*glycero*-β-D-*galacto*-oct-2-ulopyranosid]onate (**6c**)

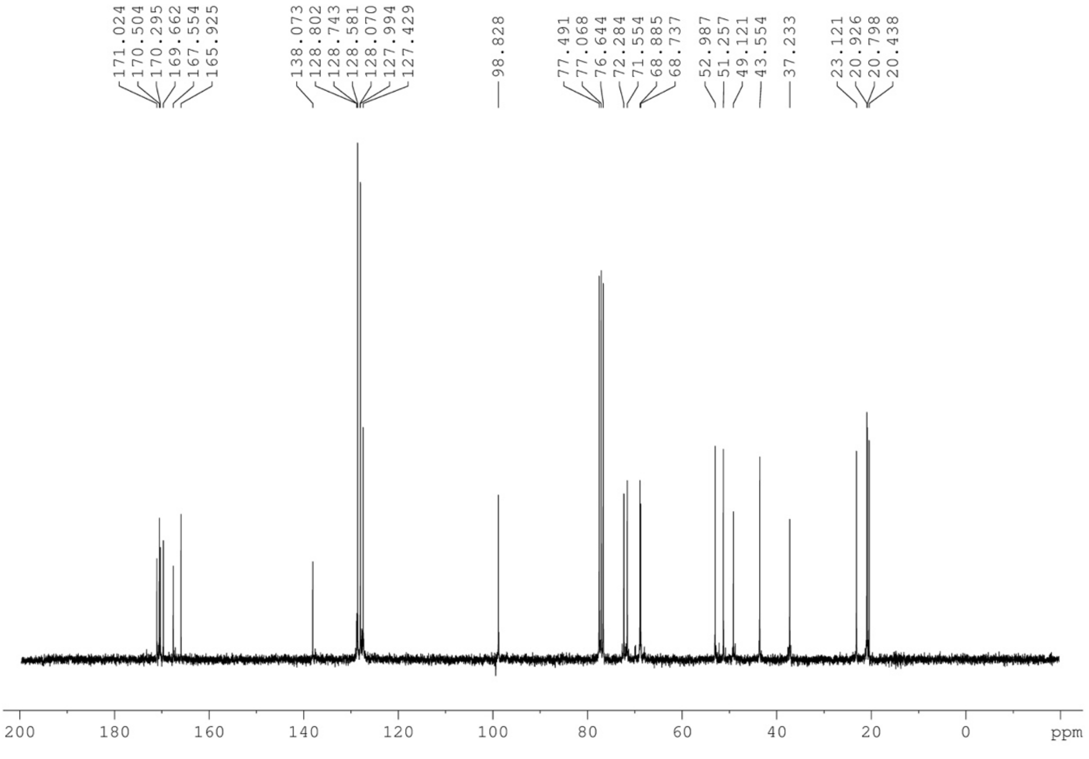

^{1}H NMR ($CDCl_3$): Methyl {methyl 5-acetamido-4,7,8-tri-*O*-acetyl-3,5-dideoxy-8-[*N*-(3-pyridyl-methyl)]carboxamido-D-*glycero*-β-D-*galacto*-oct-2-ulopyranosid}onate (**6d**)

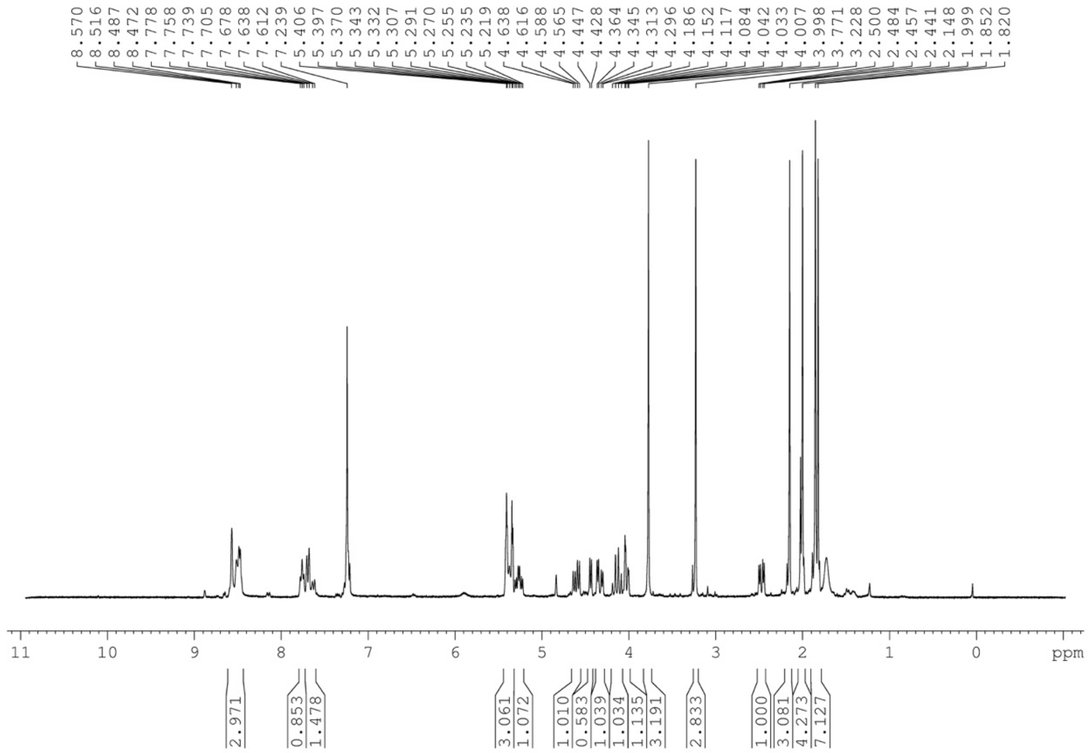

^{13}C NMR ($CDCl_3$): Methyl {methyl 5-acetamido-4,7,8-tri-*O*-acetyl-3,5-dideoxy-8-[*N*-(3-pyridyl-methyl)]carboxamido-D-*glycero*-β-D-*galacto*-oct-2-ulopyranosid}onate (**6d**)

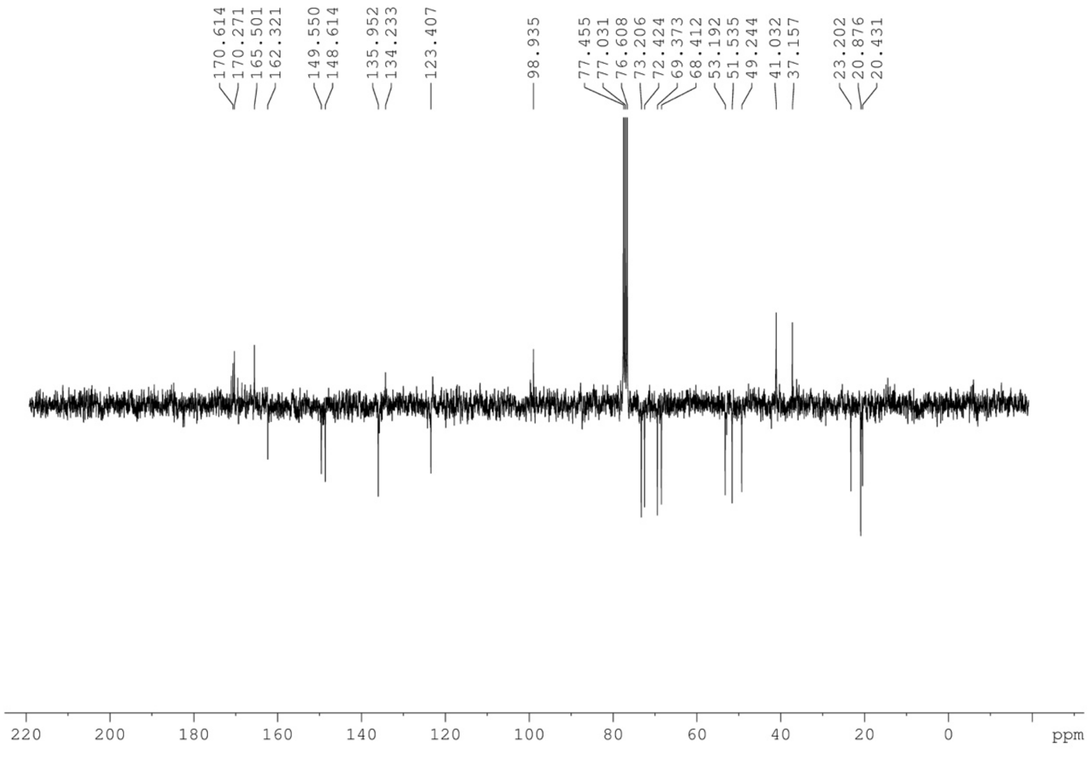

^{1}H NMR ($CDCl_3$): Methyl [methyl 5-acetamido-4,7,8-tri-*O*-acetyl-3,5-dideoxy-8-(glycine methyl ester)carboxamido-D-*glycero*-β-D-*galacto*-oct-2-ulopyranosid]onate (**7a**)

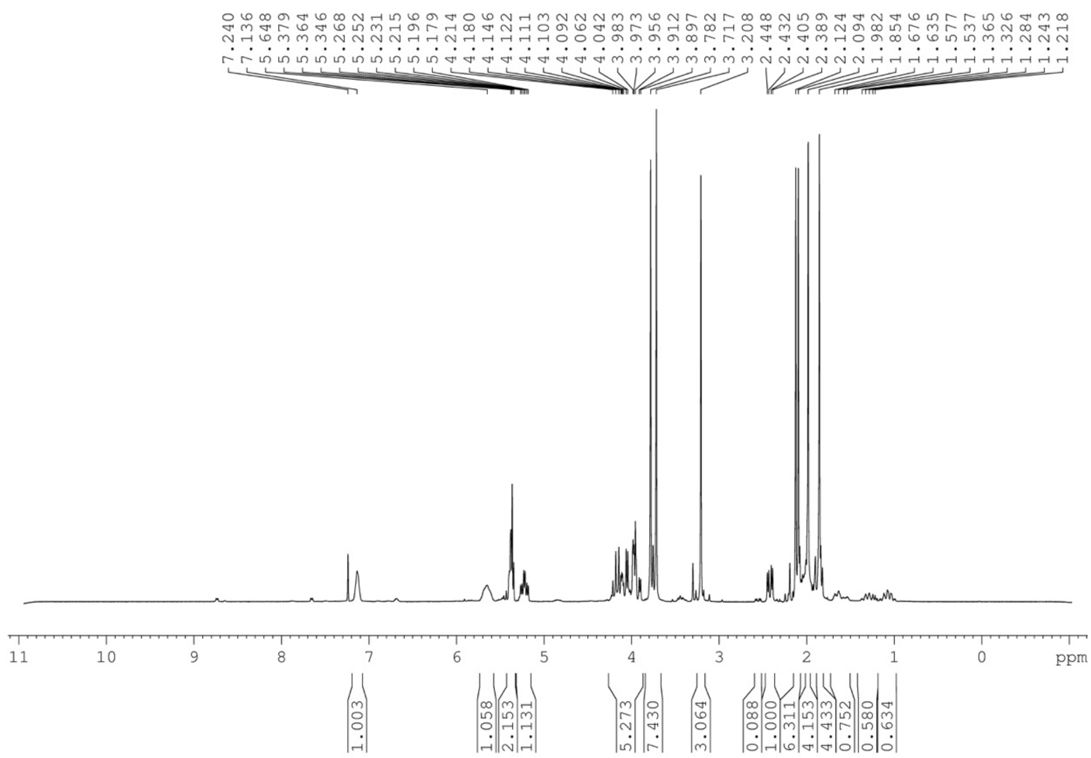

^{13}C NMR ($CDCl_3$): Methyl [methyl 5-acetamido-4,7,8-tri-*O*-acetyl-3,5-dideoxy-8-(glycine methyl ester)carboxamido-D-*glycero*-β-D-*galacto*-oct-2-ulopyranosid]onate (**7a**)

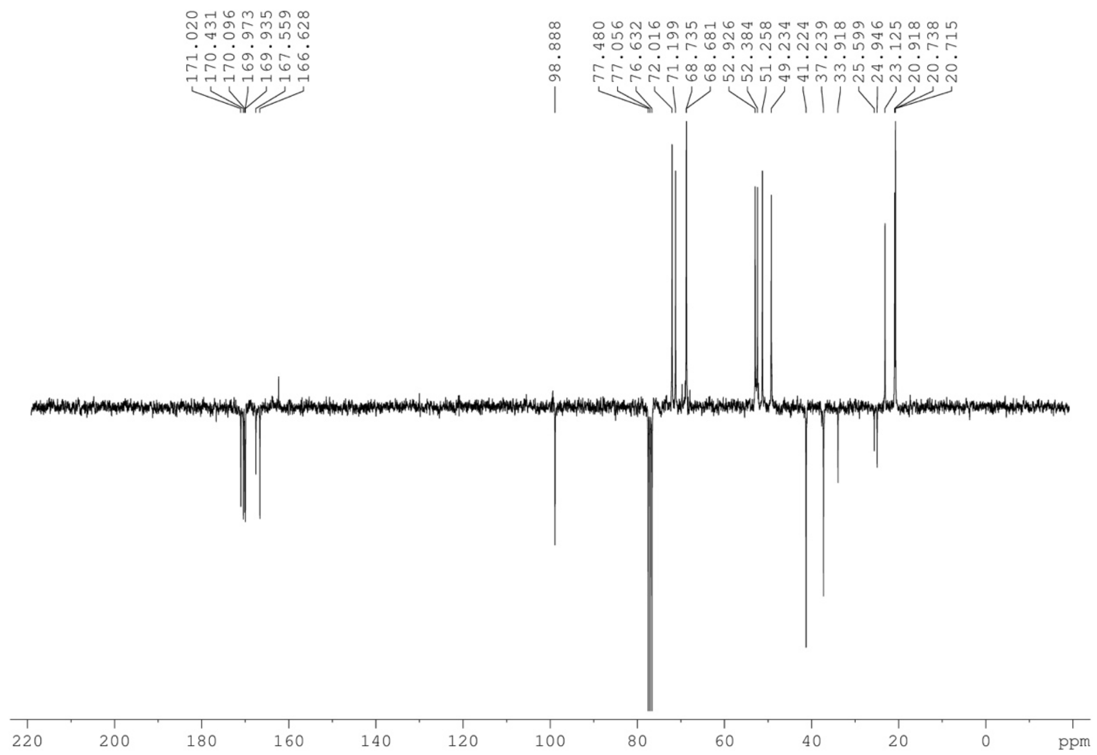

^{1}H NMR ($CDCl_3$): Methyl [methyl 5-acetamido-4,7,8-tri-*O*-acetyl-8-(L-alanine methyl ester)carboxamido-3,5-dideoxy-D-*glycero*-β-D-*galacto*-oct-2-ulopyranosid]onate (**7b**)

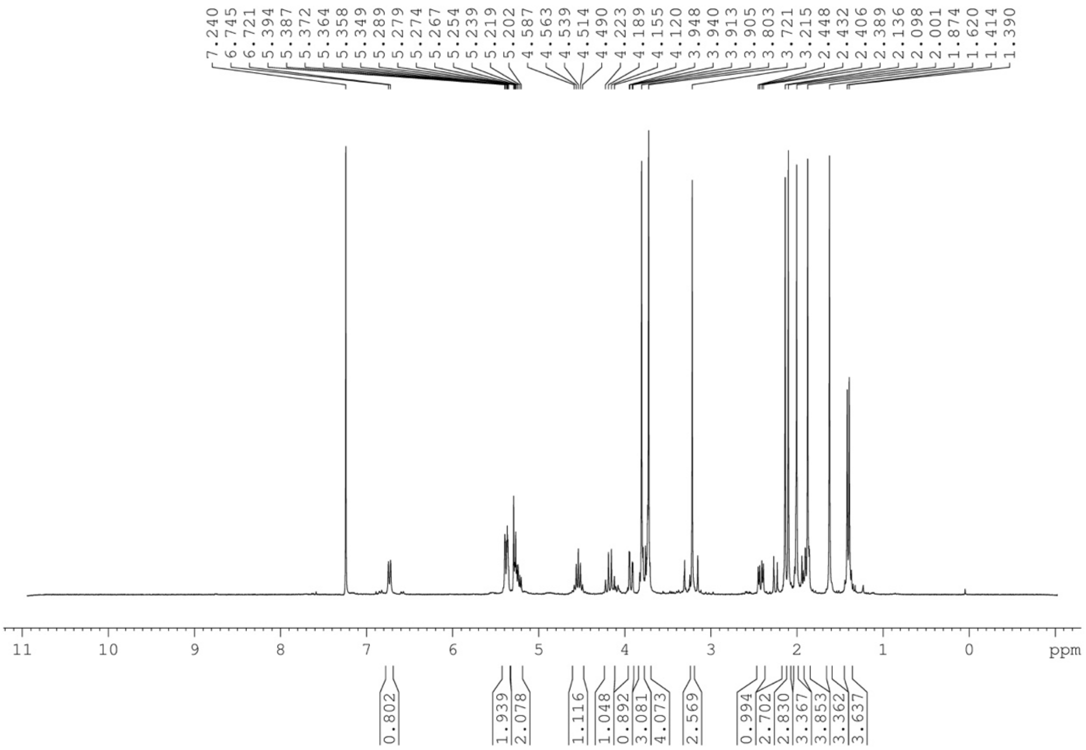

^{13}C NMR ($CDCl_3$): Methyl [methyl 5-acetamido-4,7,8-tri-*O*-acetyl-8-(L-alanine methyl ester)carboxamido-3,5-dideoxy-D-*glycero*-β-D-*galacto*-oct-2-ulopyranosid]onate (**7b**)

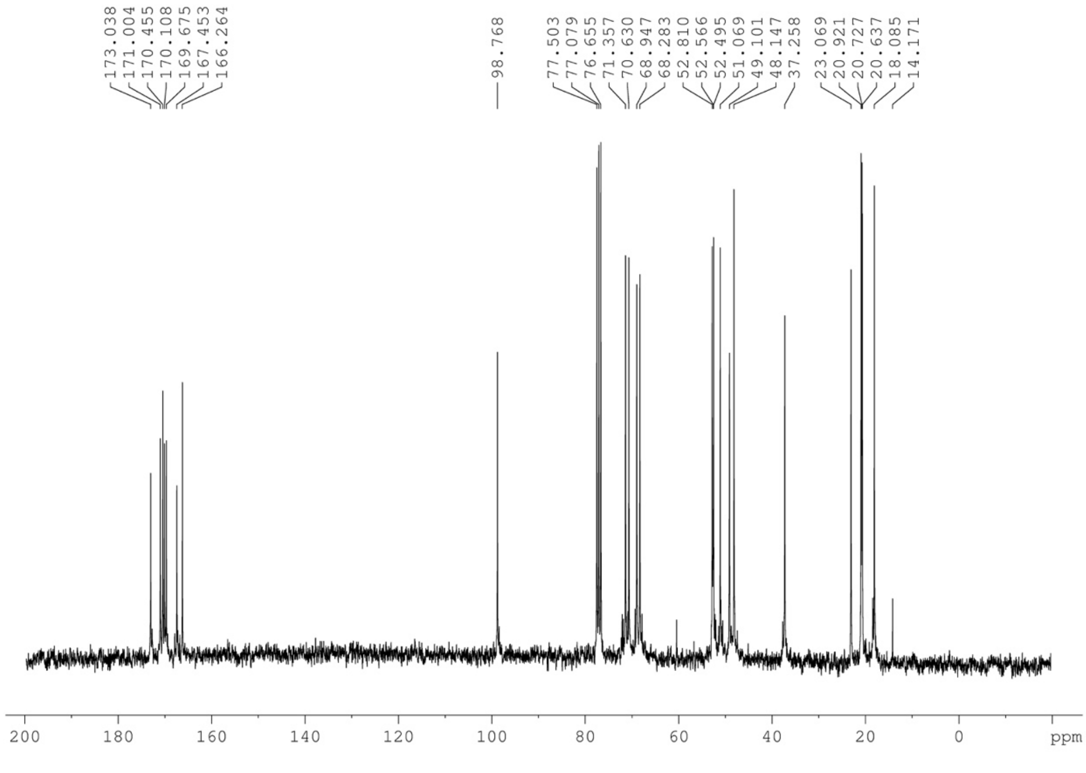

^{1}H NMR ($CDCl_3$): Methyl [methyl 5-acetamido-4,7,8-tri-*O*-acetyl-3,5-dideoxy-8-(L-leucine methyl ester)carboxamido-D-*glycero*-β-D-*galacto*-oct-2-ulopyranosid]onate (**7c**)

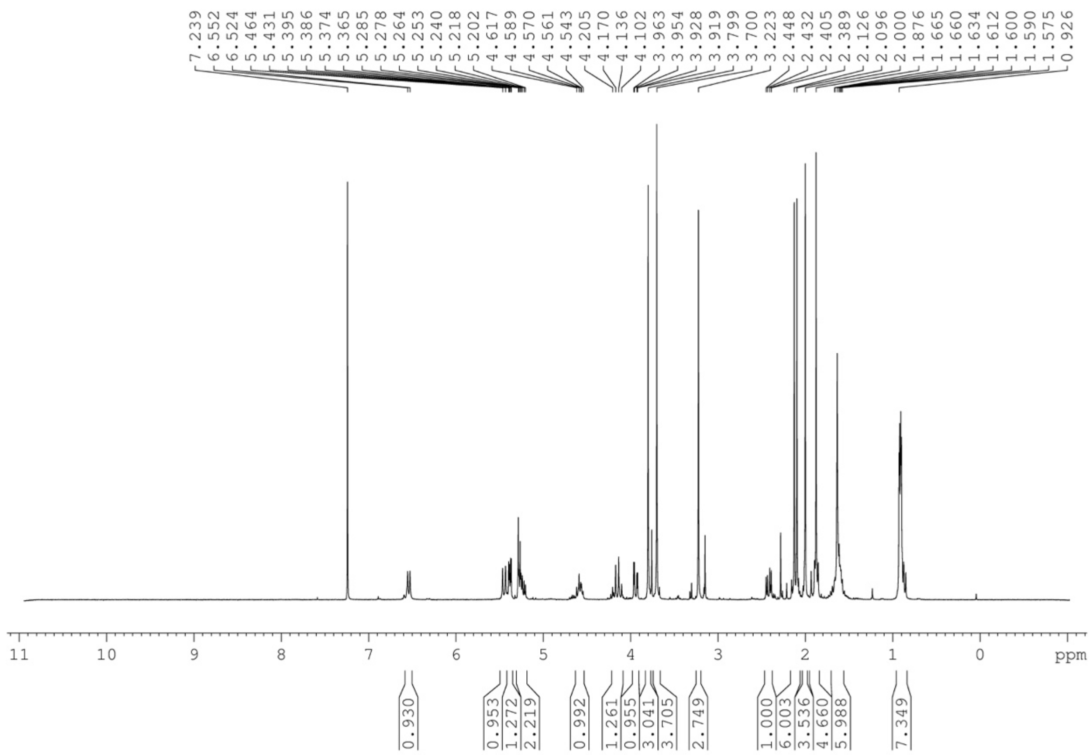

^{13}C NMR ($CDCl_3$): Methyl [methyl 5-acetamido-4,7,8-tri-*O*-acetyl-3,5-dideoxy-8-(L-leucine methyl ester)carboxamido-D-*glycero*-β-D-*galacto*-oct-2-ulopyranosid]onate (**7c**)

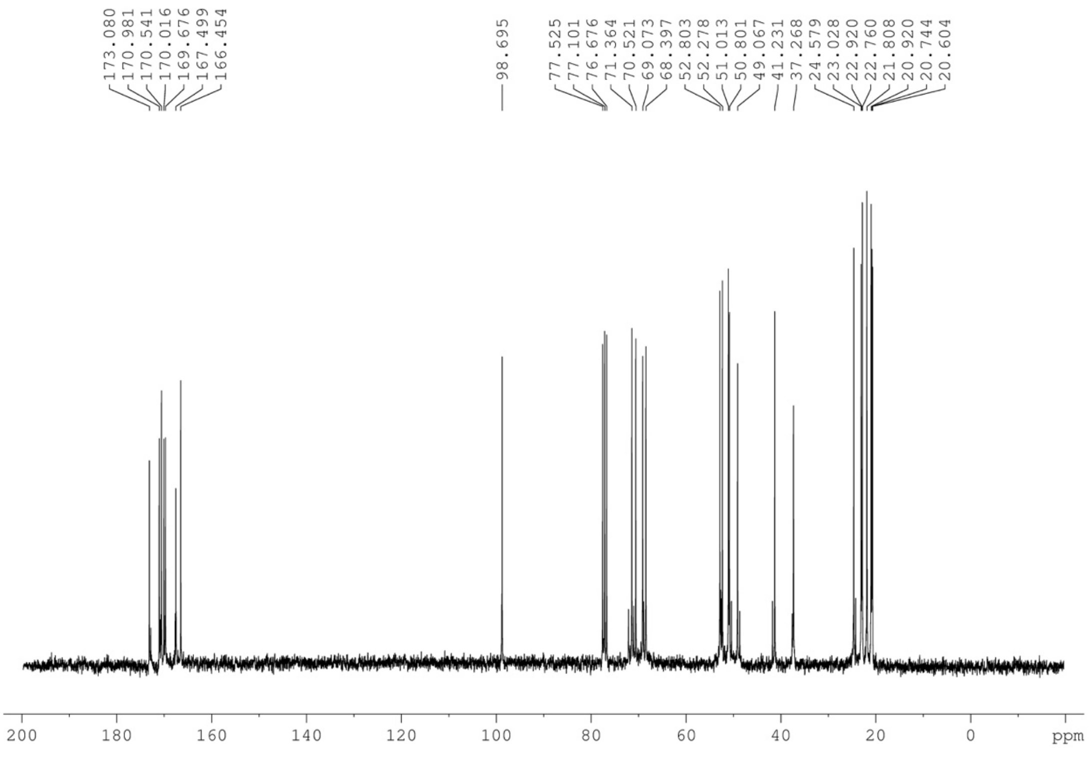

^{1}H NMR ($CDCl_3$): Methyl [methyl 5-acetamido-4,7,8-tri-*O*-acetyl-3,5,-dideoxy-8-(D/L-serine methyl ester)carboxamido-D-*glycero*-β-D-*galacto*-oct-2-ulopyranosid]onate (**7d**)

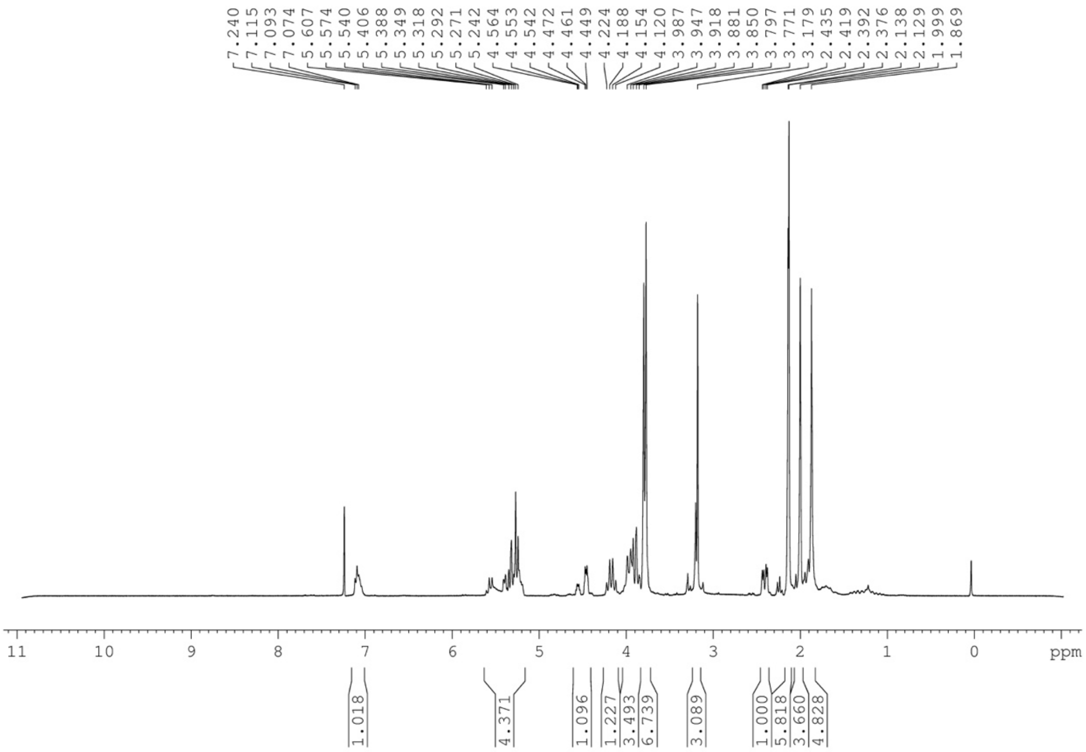

^{13}C NMR ($CDCl_3$): Methyl [methyl 5-acetamido-4,7,8-tri-*O*-acetyl-3,5,-dideoxy-8-(D/L-serine methyl ester)carboxamido-D-*glycero*-β-D-*galacto*-oct-2-ulopyranosid]onate (**7d**)

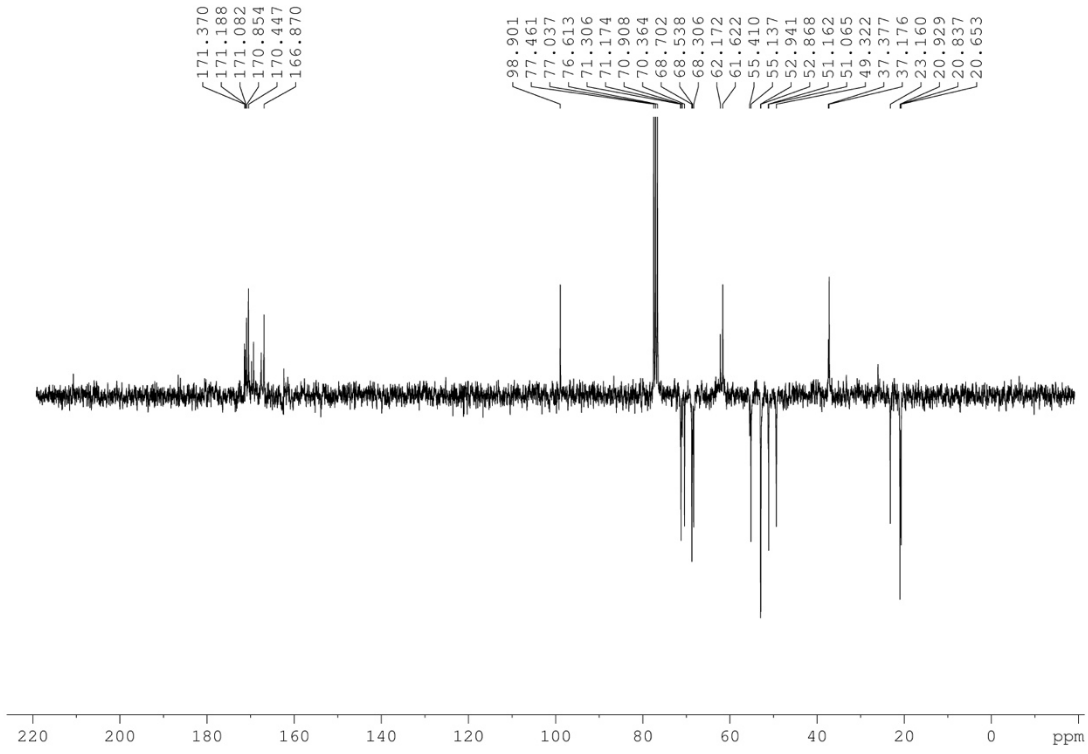

^{1}H NMR ($CDCl_3$): Methyl [methyl 5-acetamido-4,7,8-tri-*O*-acetyl-3,5-dideoxy-8-(L-phenylalanine methyl ester)carboxamido-D-*glycero*-β-D-*galacto*-oct-2-ulopyranosid]onate (**7e**)

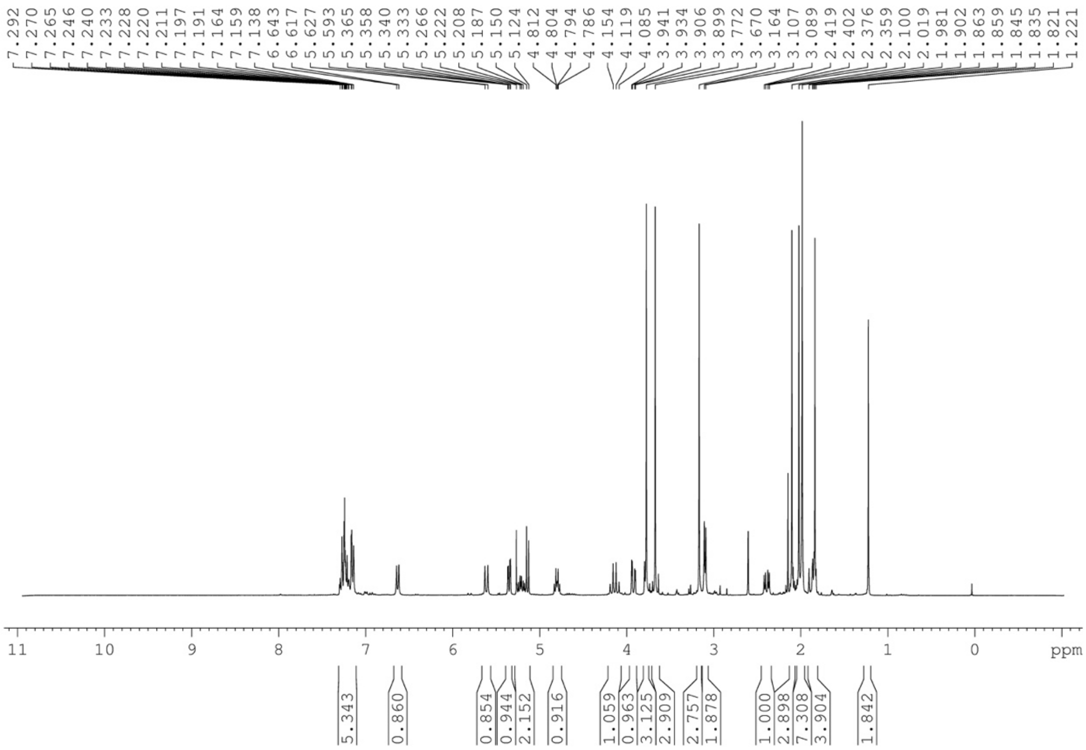

^{13}C NMR ($CDCl_3$): Methyl [methyl 5-acetamido-4,7,8-tri-*O*-acetyl-3,5-dideoxy-8-(L-phenylalanine methyl ester)carboxamido-D-*glycero*-β-D-*galacto*-oct-2-ulopyranosid]onate (**7e**)

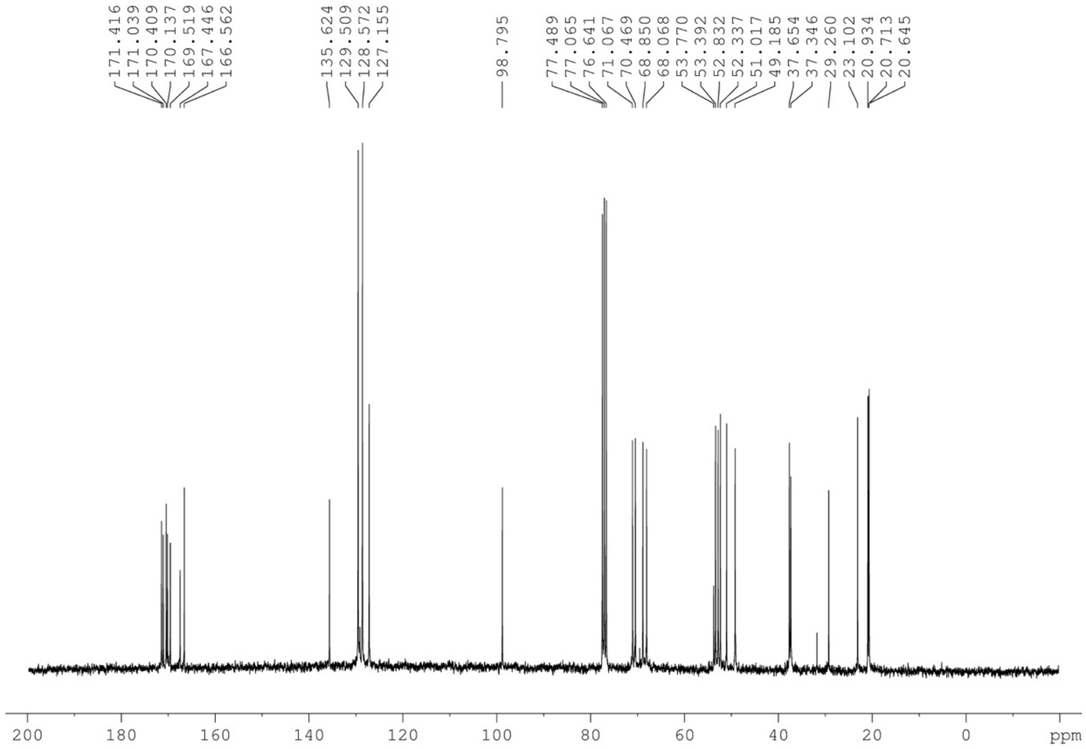

^{1}H NMR (CD_3OD): Methyl [methyl 5-acetamido-4,7,8-tri-*O*-acetyl-3,5-dideoxy-8-(L-tyrosine methyl ester)carboxamido-D-*glycero*-β-D-*galacto*-oct-2-ulopyranosid]onate (**7f**)

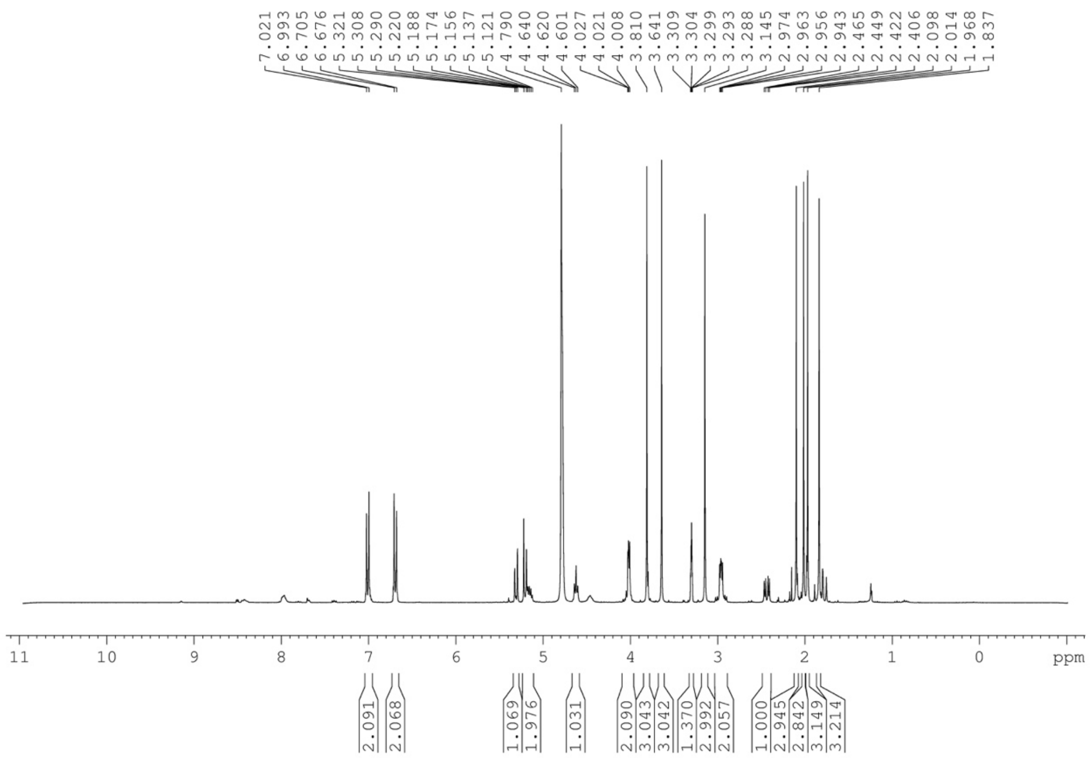

^{13}C NMR (CD_3OD): Methyl [methyl 5-acetamido-4,7,8-tri-*O*-acetyl-3,5-dideoxy-8-(L-tyrosine methyl ester)carboxamido-D-*glycero*-β-D-*galacto*-oct-2-ulopyranosid]onate (**7f**)

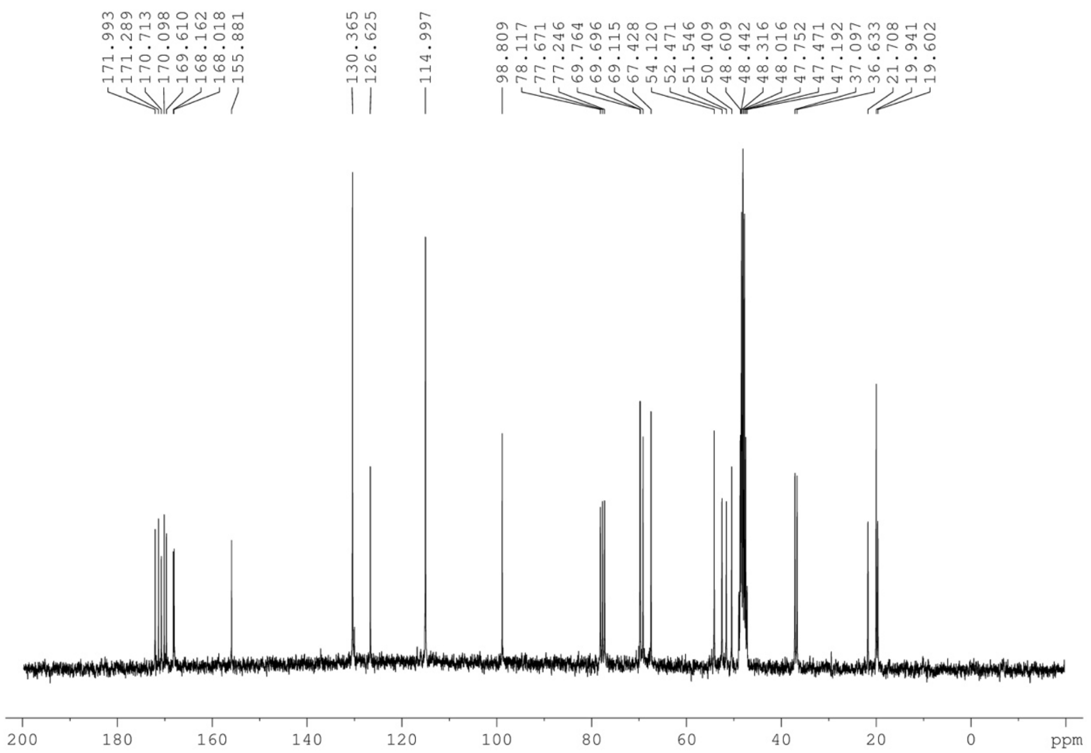

^{1}H NMR ($CDCl_3$): Methyl [methyl 5-acetamido-4,7,8-tri-*O*-acetyl-3,5-dideoxy-8-(L-histidine methyl ester)carboxamido-D-*glycero*-β-D-*galacto*-oct-2-ulopyranosid]onate (**7g**)

[Note: a small amount of unreacted histidine methyl ester co-eluted with the product]

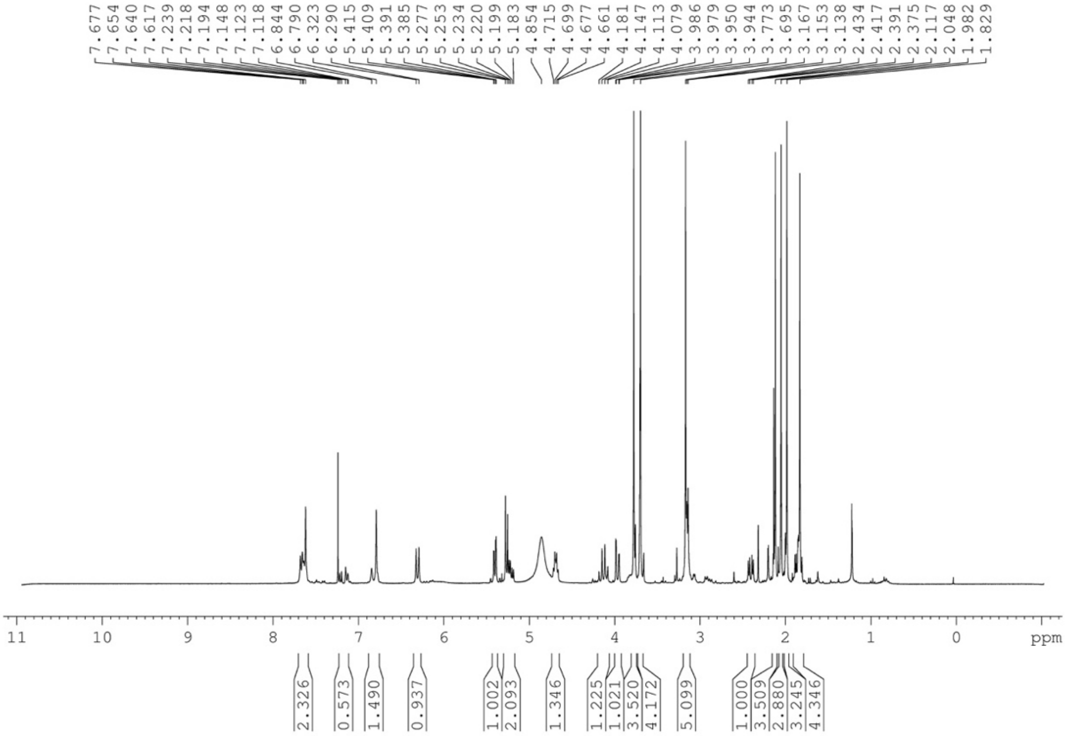

^{13}C NMR ($CDCl_3$): Methyl [methyl 5-acetamido-4,7,8-tri-*O*-acetyl-3,5-dideoxy-8-(L-histidine methyl ester)carboxamido-D-*glycero*-β-D-*galacto*-oct-2-ulopyranosid]onate (**7g**)

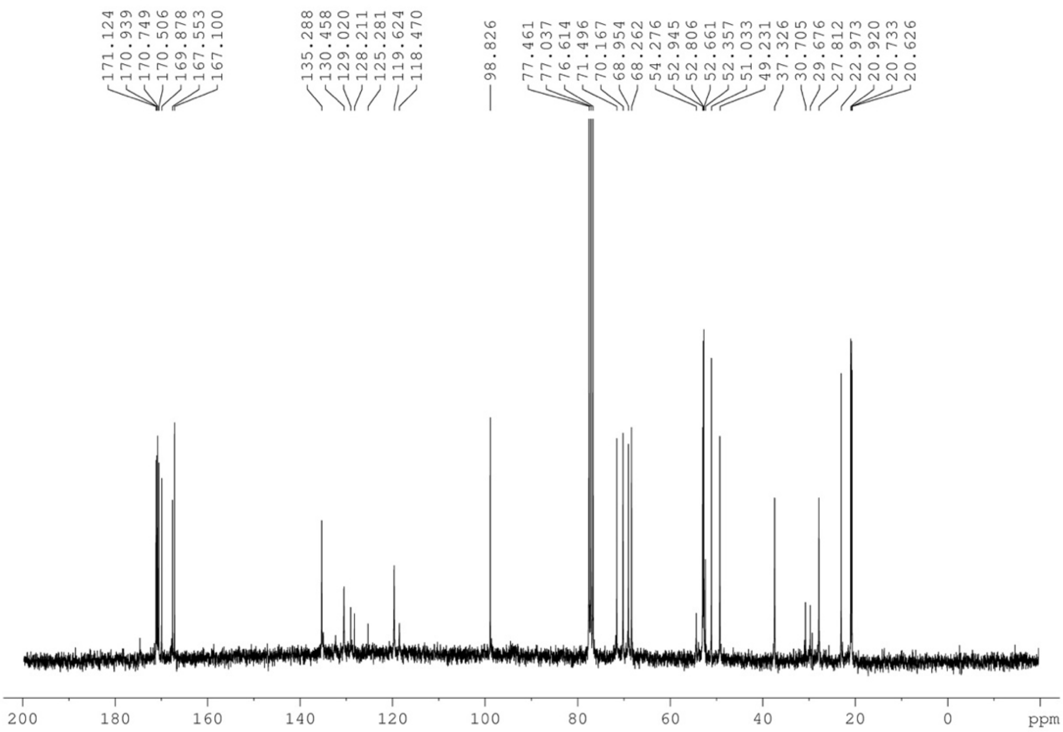

^{1}H NMR ($CDCl_3$): Methyl [methyl 5-acetamido-4,7,8-tri-*O*-acetyl-3,5-dideoxy-8-(L-tryptophan methyl ester)carboxamido-D-*glycero*-β-D-*galacto*-oct-2-ulopyranosid]onate (**7h**)

[Note: some unreacted tryptophan methyl ester is present in this product.]

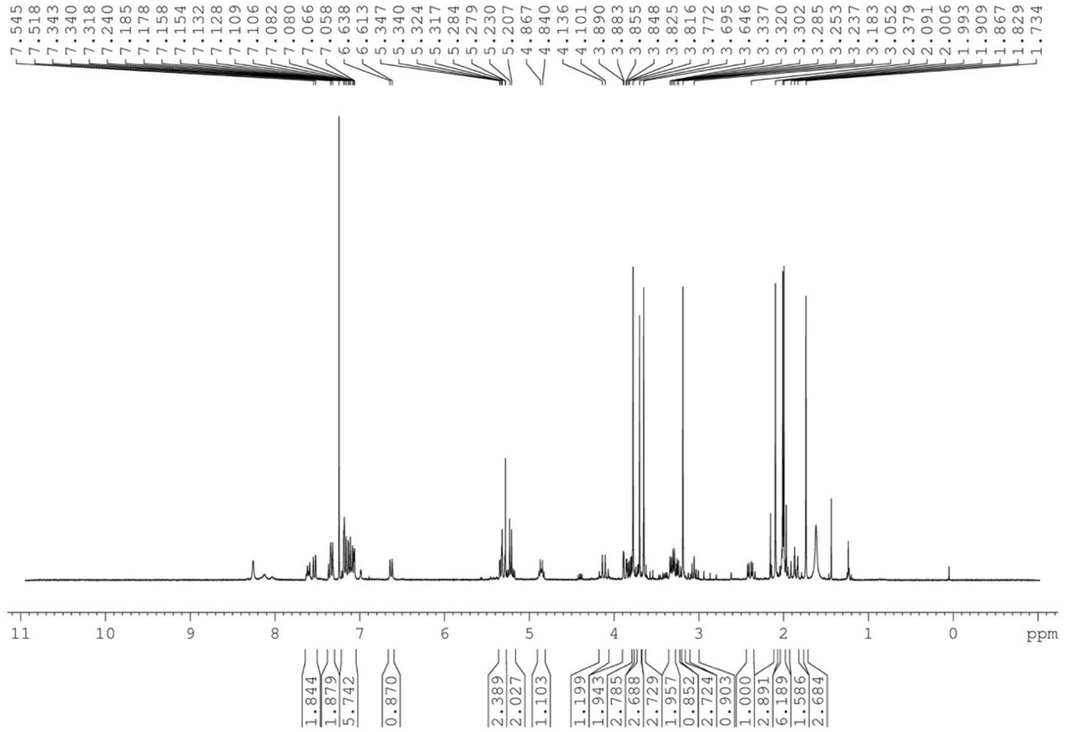

^{13}C NMR ($CDCl_3$): Methyl [methyl 5-acetamido-4,7,8-tri-*O*-acetyl-3,5-dideoxy-8-(L-tryptophan methyl ester)carboxamido-D-*glycero*-β-D-*galacto*-oct-2-ulopyranosid]onate (**7h**)

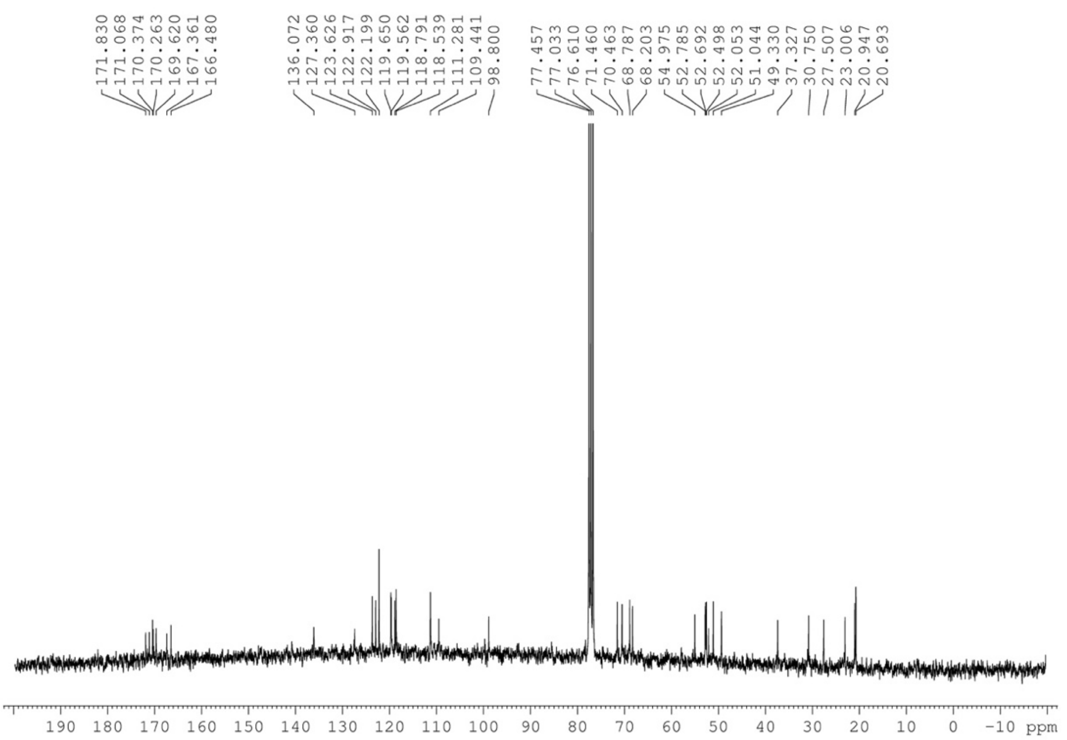

^{1}H NMR (D_2O): Methyl 5-acetamido-8-carboxy-3,5-dideoxy-D-*glycero*-β-D-*galacto*-oct-2-ulopyranosidonic acid (**8**)

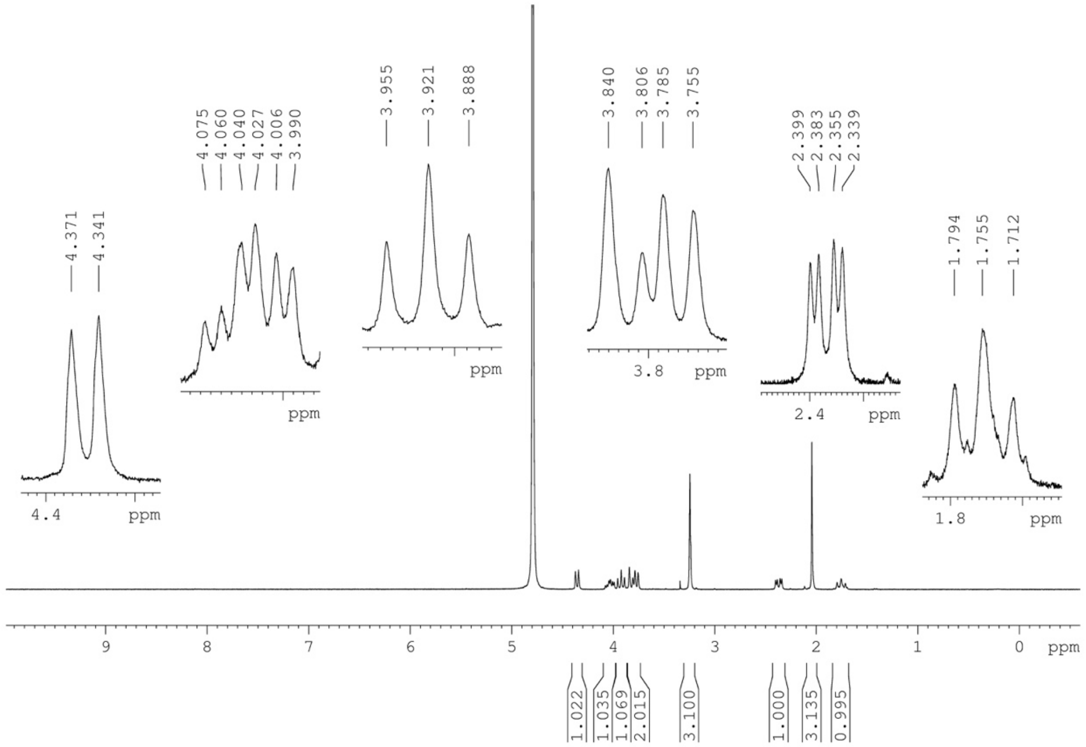

^{13}C NMR (D_2O): Methyl 5-acetamido-8-carboxy-3,5-dideoxy-D-*glycero*-β-D-*galacto*-oct-2-ulopyranosidonic acid (**8**)

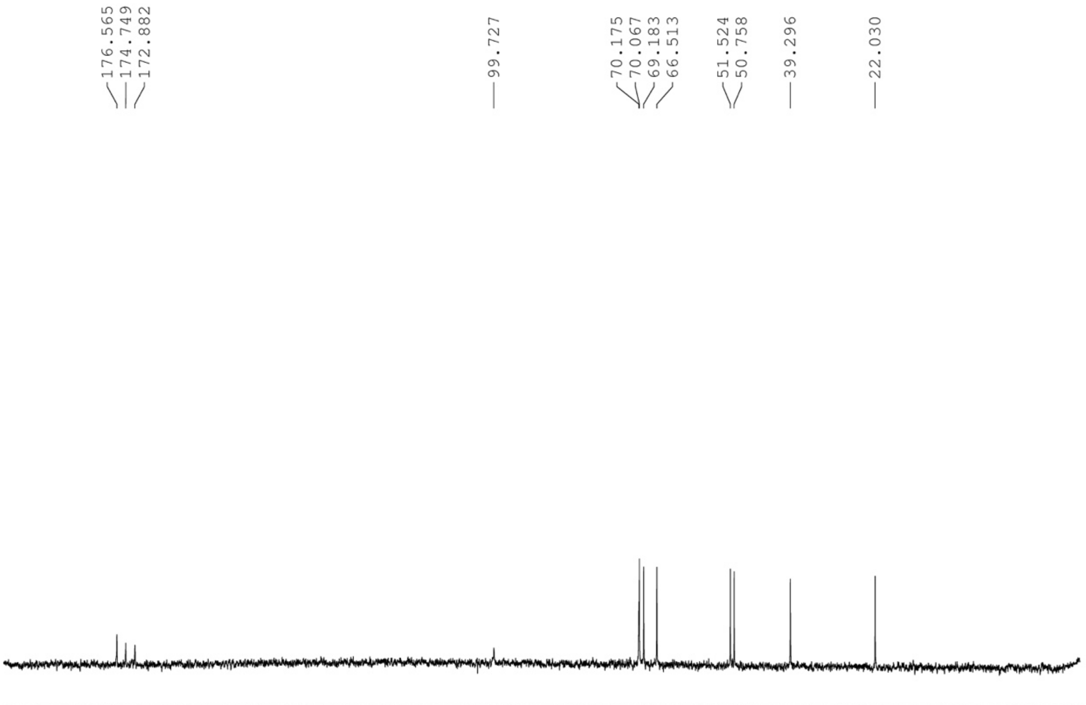

^{1}H NMR (D_2O): Methyl 5-acetamido-8-(*N*-butyl)carboxamido-3,5-dideoxy-D-*glycero*-β-D-*galacto*-oct-2-ulopyranosidonic acid (**9a**)

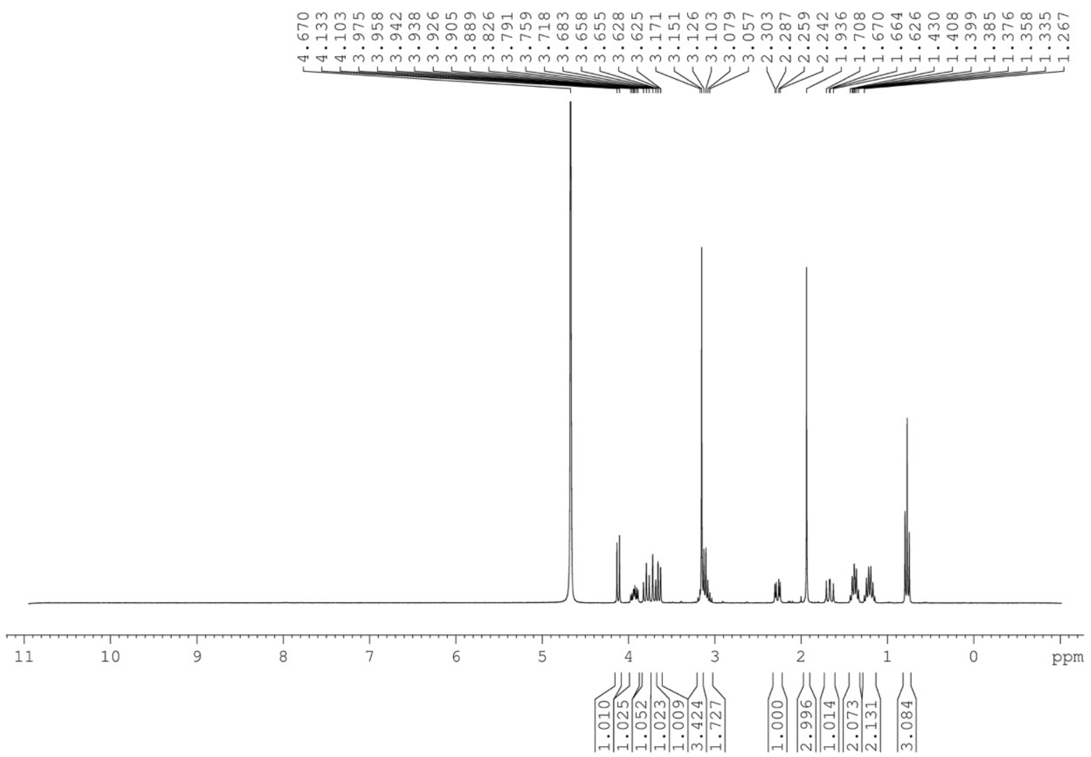

^{13}C NMR (D_2O): Methyl 5-acetamido-8-(*N*-butyl)carboxamido-3,5-dideoxy-D-*glycero*-β-D-*galacto*-oct-2-ulopyranosidonic acid (**9a**)

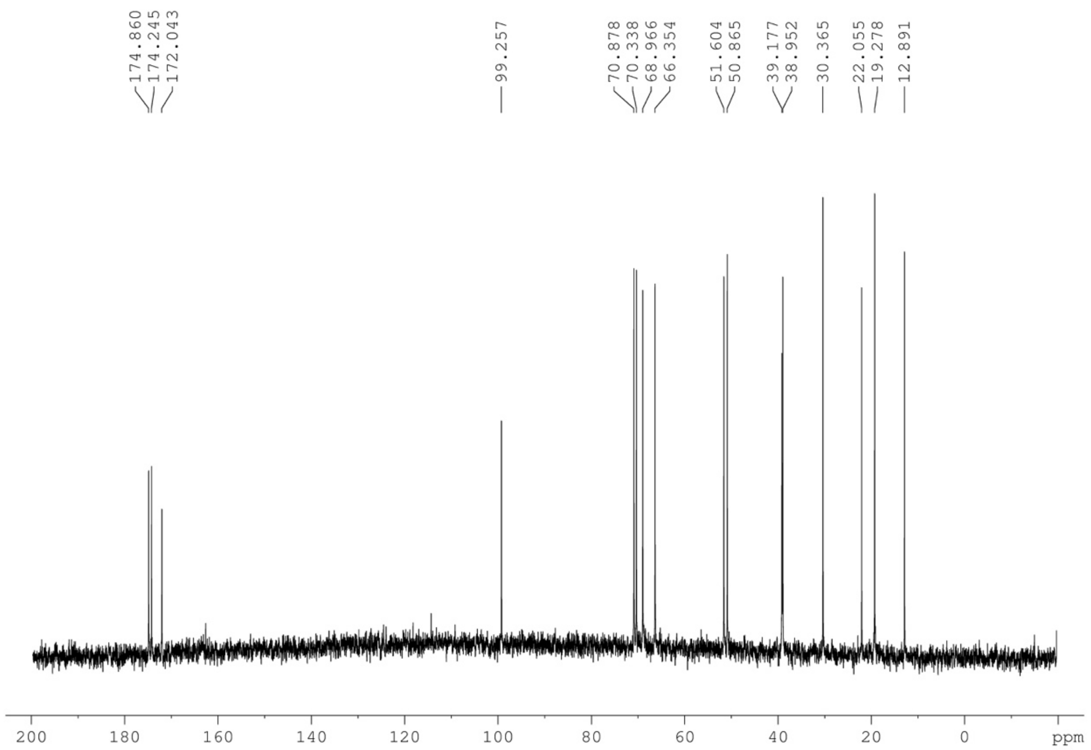

^{1}H NMR (D_2O): Methyl 5-acetamido-8-(*N*-cyclohexyl)carboxamido-3,5-dideoxy-D-*glycero*-β-D-*galacto*-oct-2-ulopyranosidonic acid (**9b**)

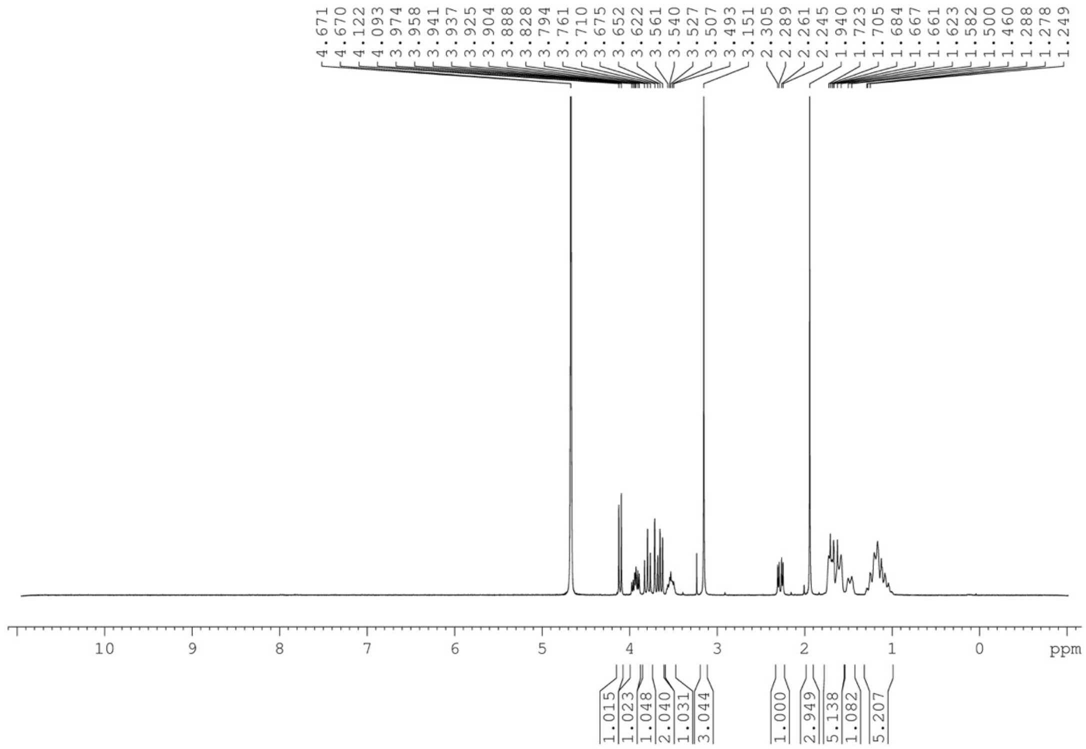

^{13}C NMR (D_2O): Methyl 5-acetamido-8-(*N*-cyclohexyl)carboxamido-3,5-dideoxy-D-*glycero*-β-D-*galacto*-oct-2-ulopyranosidonic acid (**9b**)

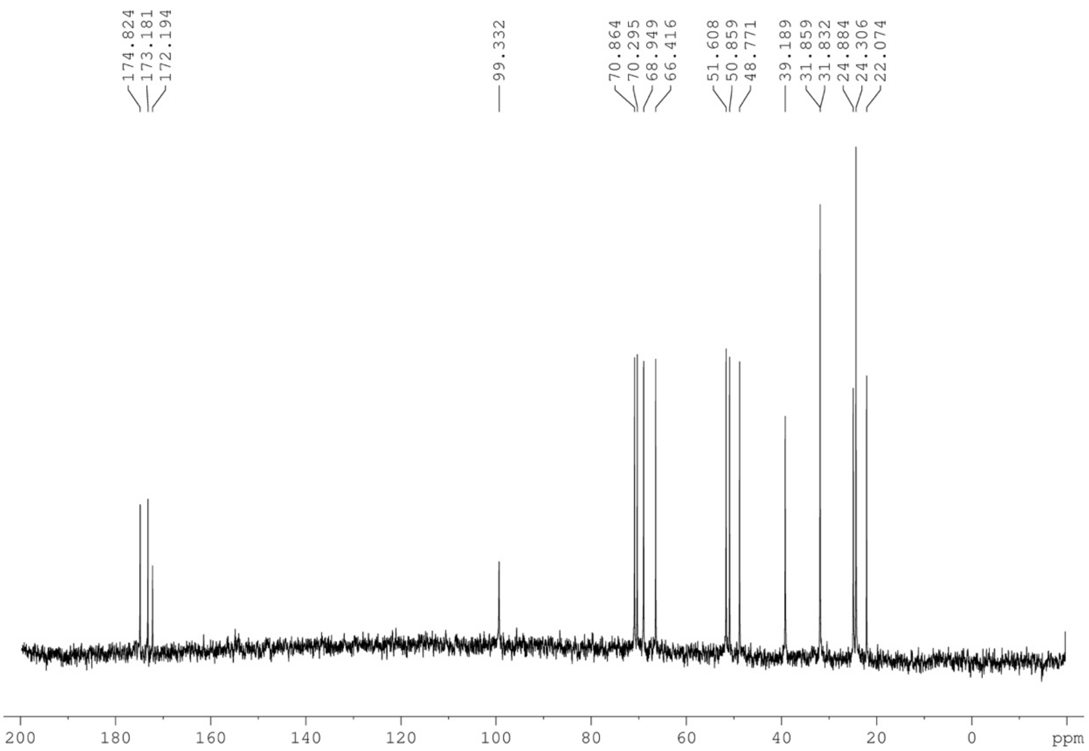

^{1}H NMR (CD_3OD): Methyl 5-acetamido-8-(*N*-benzyl)carboxamido-3,5-dideoxy-D-*glycero*-β-D-*galacto*-oct-2-ulopyranosidonic acid (**9c**)

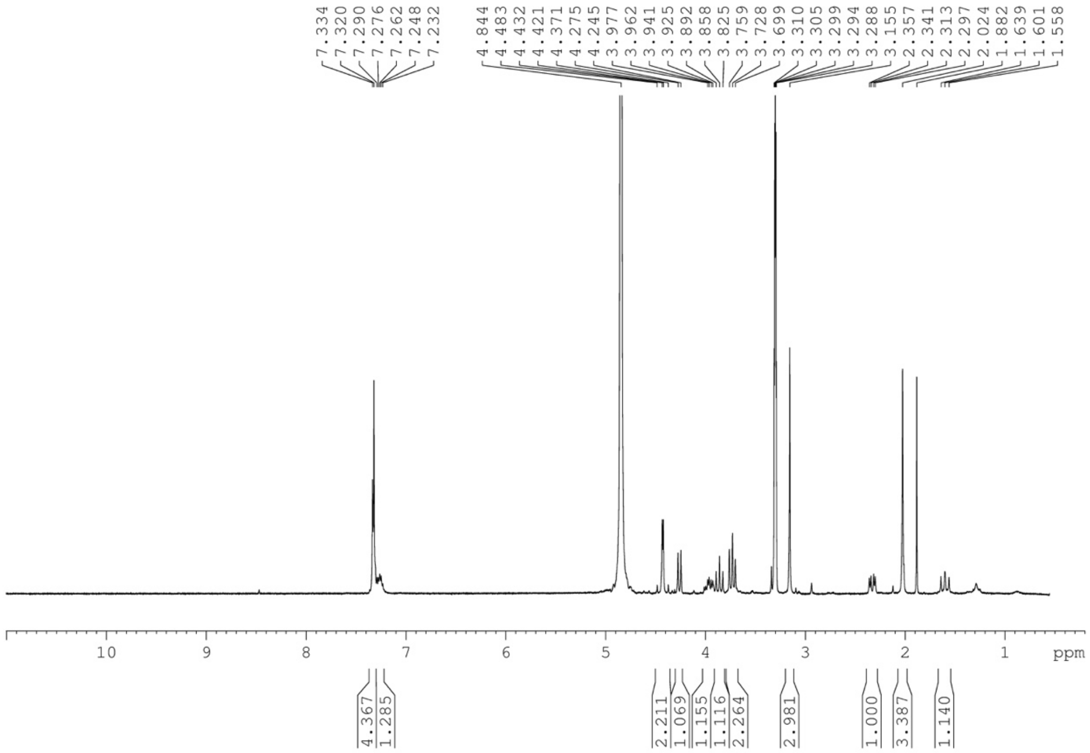

^{13}C NMR (D_2O): Methyl 5-acetamido-8-(*N*-benzyl)carboxamido-3,5-dideoxy-D-*glycero*-β-D-*galacto*-oct-2-ulopyranosidonic acid (**9c**)

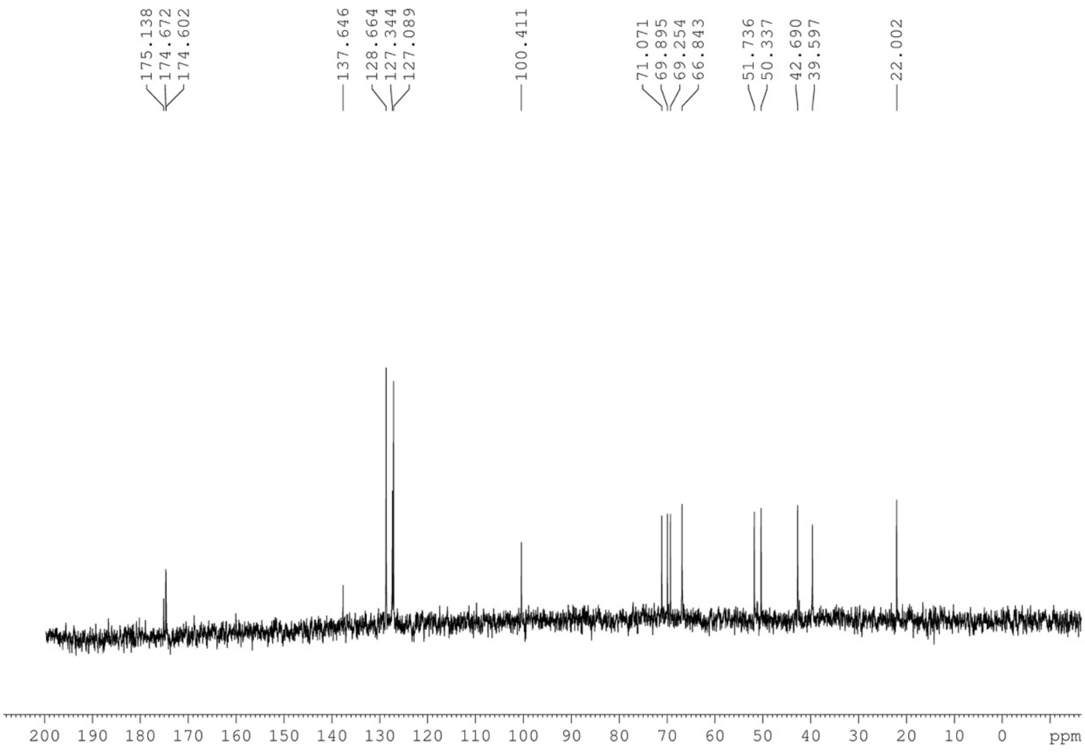

^{1}H NMR (D_2O): Methyl 5-acetamido-3,5-dideoxy-8-[*N*-(3-pyridyl-methyl)]carboxamido-D-*glycero*-β-D-*galacto*-oct-2-ulopyranosidonic acid (**9d**)

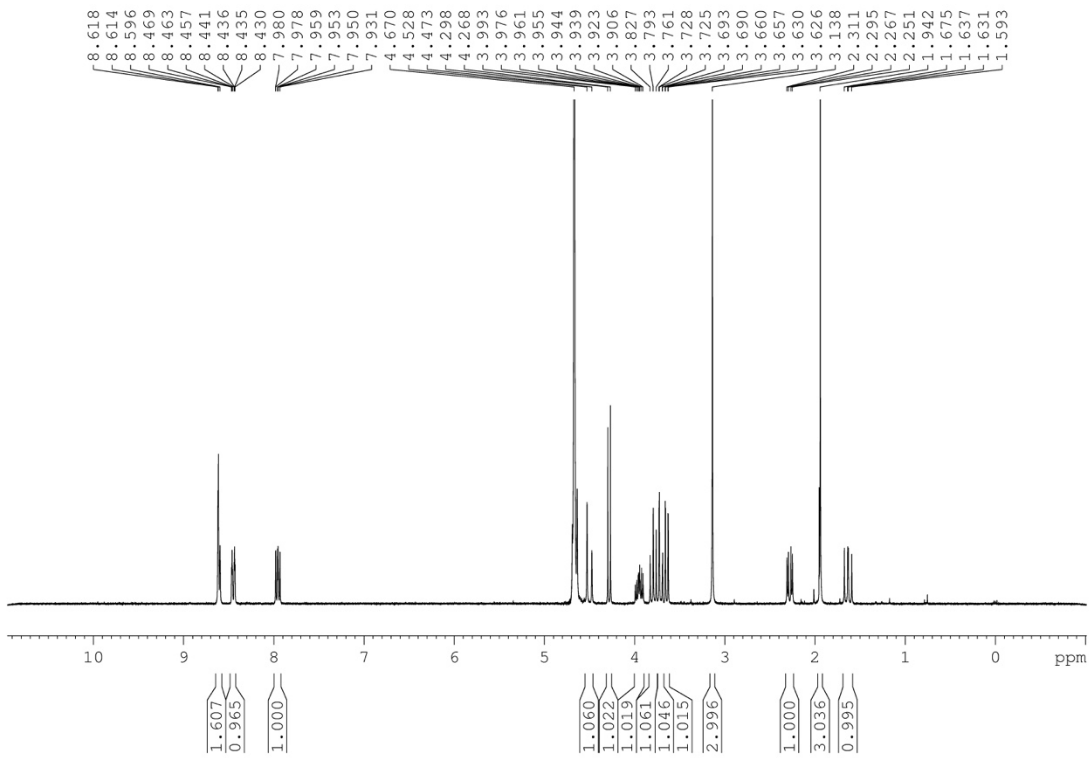

^{13}C NMR (D_2O): Methyl 5-acetamido-3,5-dideoxy-8-[*N*-(3-pyridyl-methyl)]carboxamido-D-*glycero*-β-D-*galacto*-oct-2-ulopyranosidonic acid (**9d**)

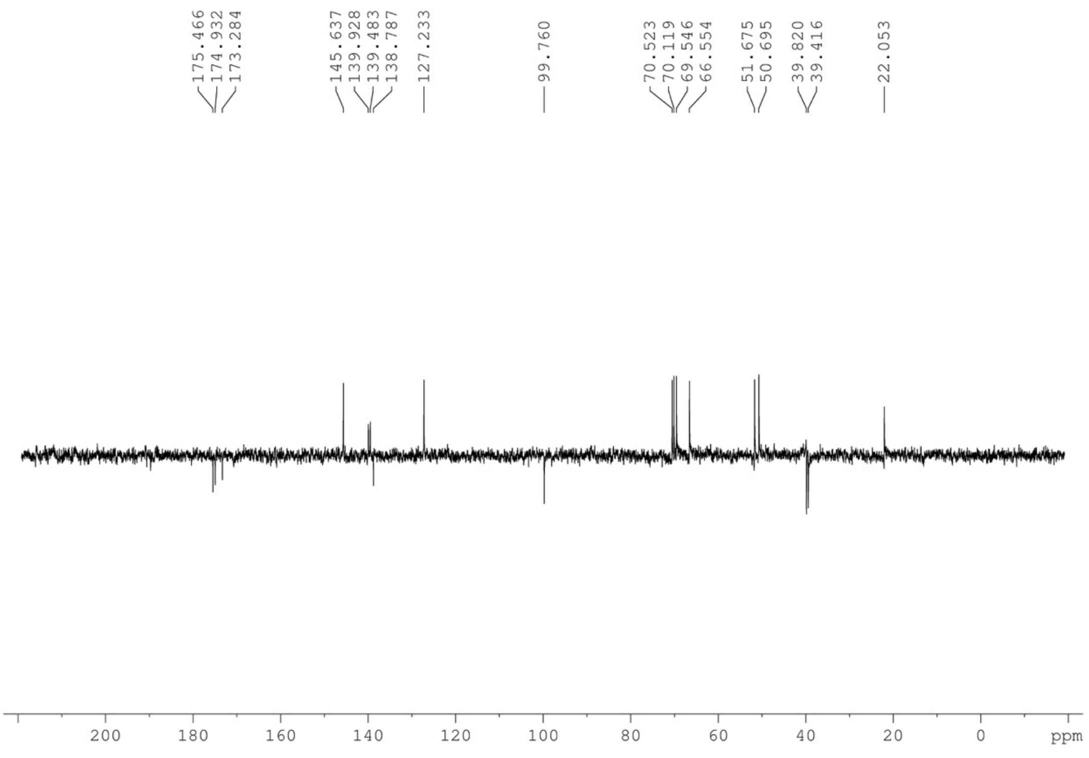

^{1}H NMR (D_2O): Methyl 5-acetamido-3,5-dideoxy-8-(glycine)carboxamido-D-*glycero*-β-D-*galacto*-oct-2-ulopyranosidonic acid (**10a**)

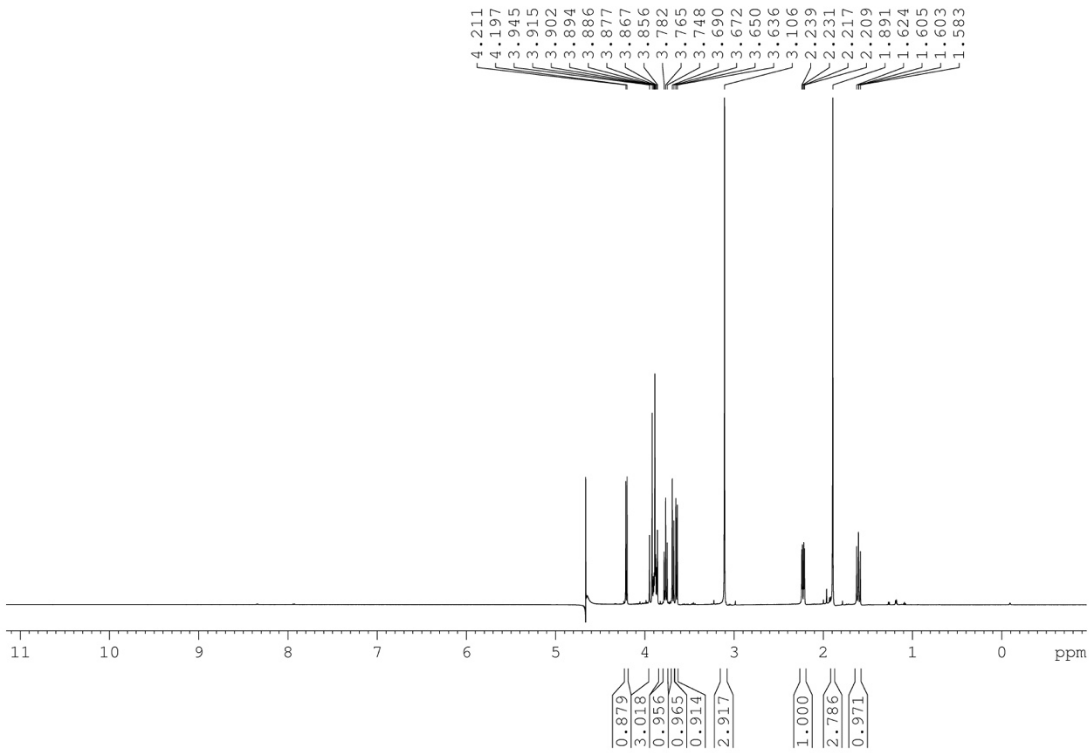

^{13}C NMR (D_2O): Methyl 5-acetamido-3,5-dideoxy-8-(glycine)carboxamido-D-*glycero*-β-D-*galacto*-oct-2-ulopyranosidonic acid (**10a**)

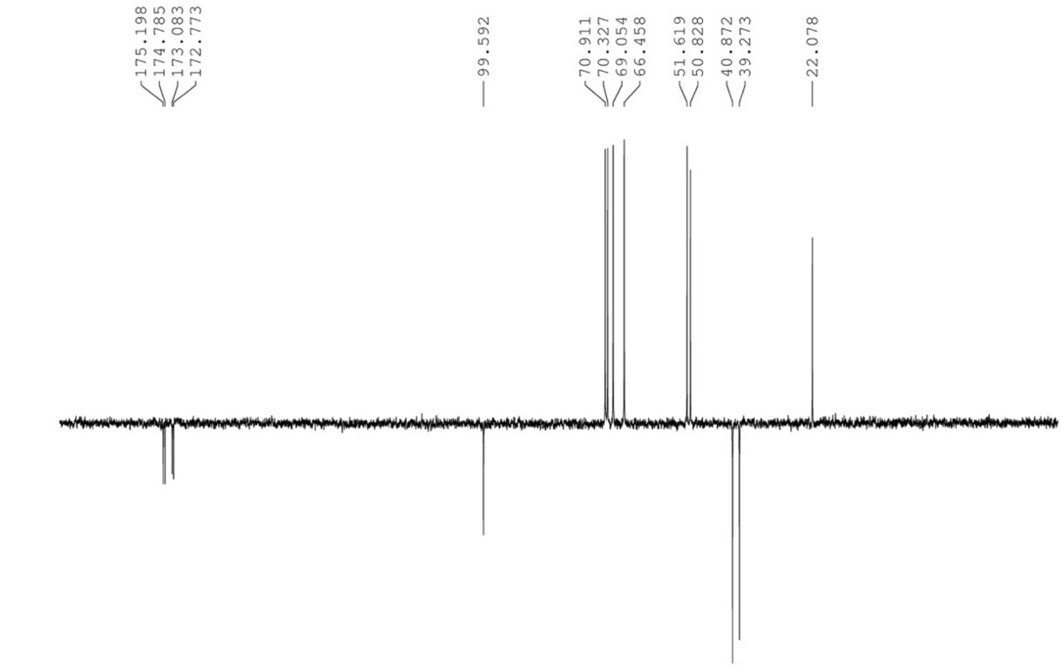

^{1}H NMR (D_2O): Methyl 5-acetamido-8-(L-alanine)carboxamido-3,5-dideoxy-D-*glycero*-β-D-*galacto*-oct-2-ulopyranosidonic acid (**10b**)

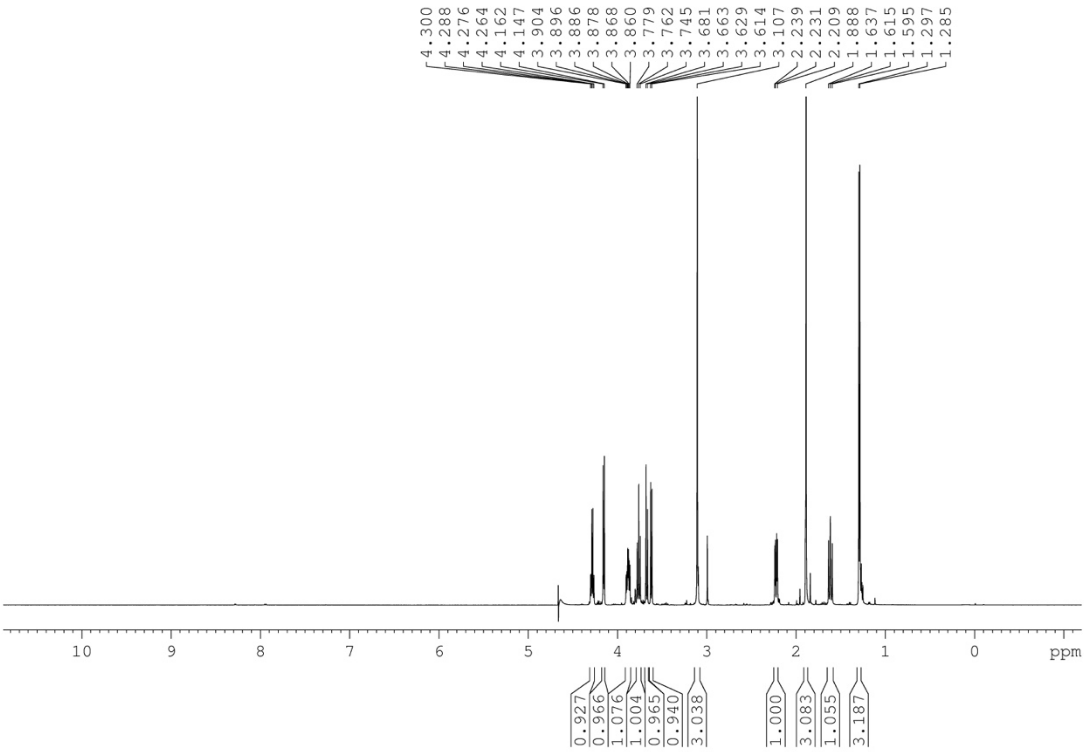

^{13}C NMR (D_2O): Methyl 5-acetamido-8-(L-alanine)carboxamido-3,5-dideoxy-D-*glycero*-β-D-*galacto*-oct-2-ulopyranosidonic acid (**10b**)

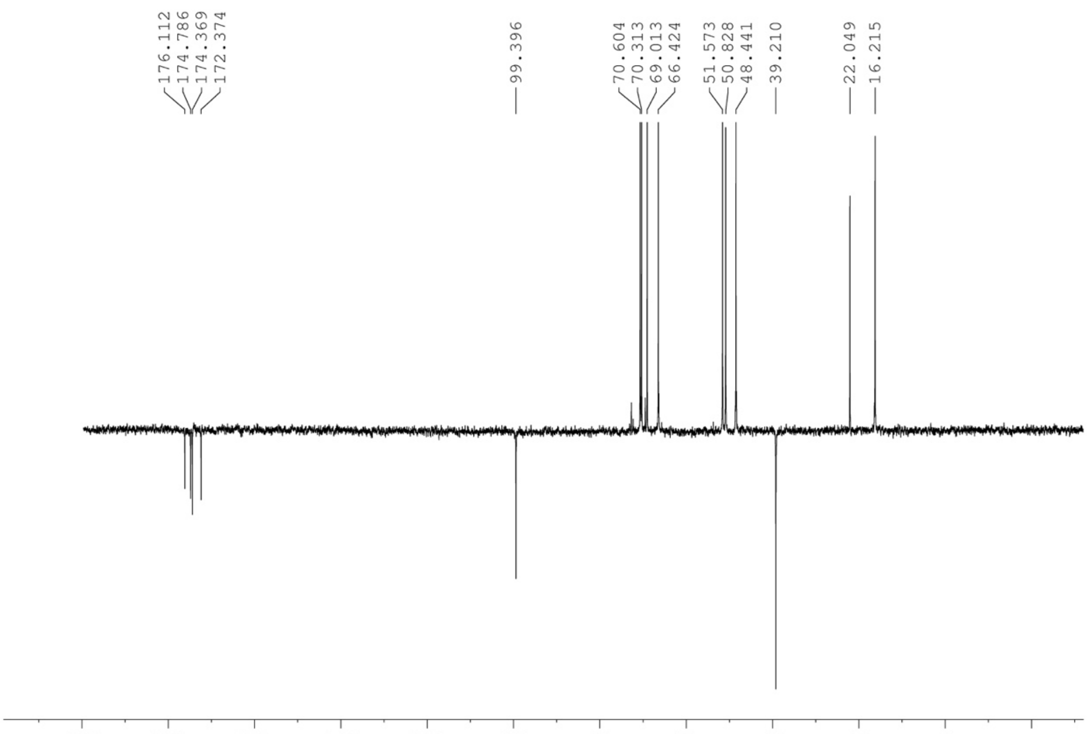

^{1}H NMR (D_2O): Methyl 5-acetamido-3,5-dideoxy-8-(L-leucine)carboxamido-D-*glycero*-β-D-*galacto*-oct-2-ulopyranosidonic acid (**10c**)

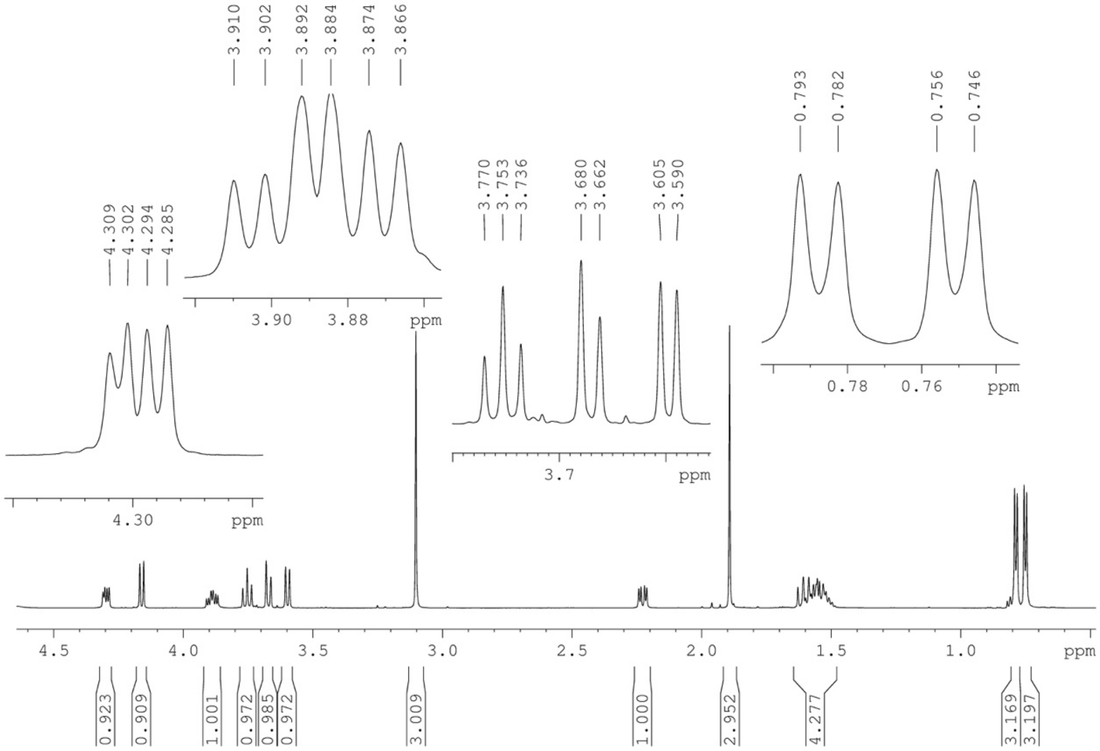

^{13}C NMR (D_2O): Methyl 5-acetamido-3,5-dideoxy-8-(L-leucine)carboxamido-D-*glycero*-β-D-*galacto*-oct-2-ulopyranosidonic acid (**10c**)

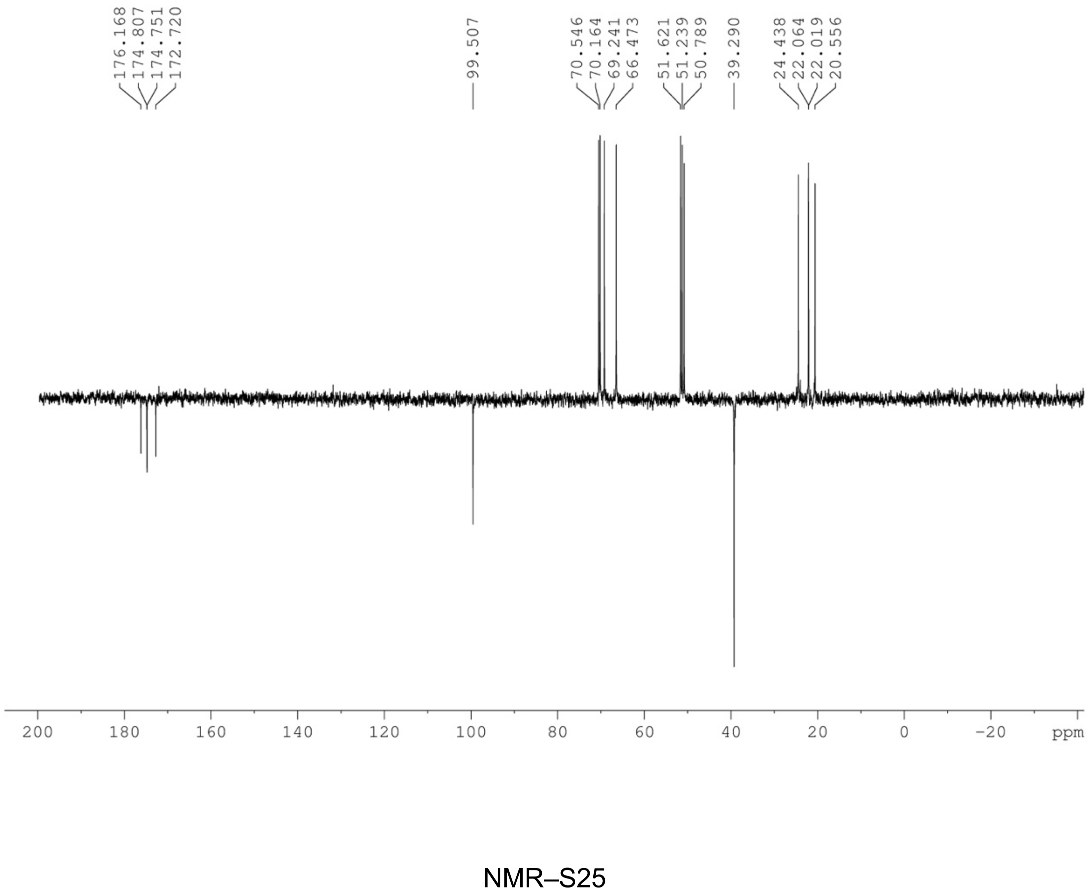

^{1}H NMR (D_2O): Methyl 5-acetamido-3,5-dideoxy-8-(D/L-serine)carboxamido-D-*glycero*-β-D-*galacto*-oct-2-ulopyranosidonic acid (**10d-1**)

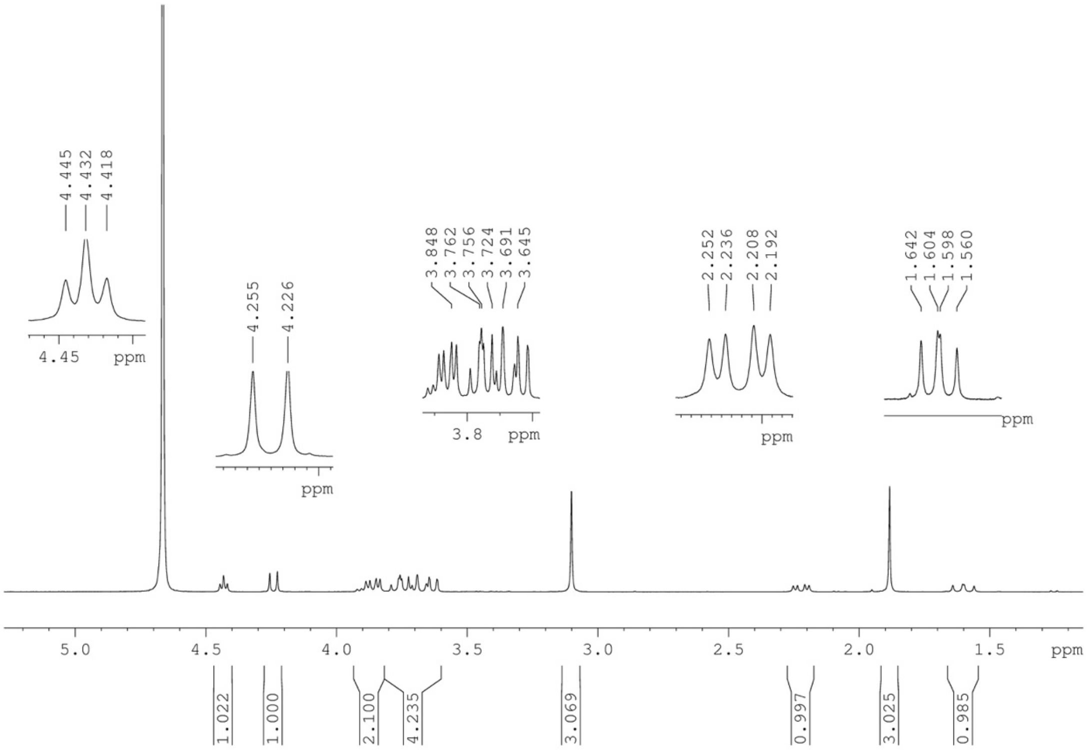

^{13}C NMR (D_2O): Methyl 5-acetamido-3,5-dideoxy-8-(D/L-serine)carboxamido-D-*glycero*-β-D-*galacto*-oct-2-ulopyranosidonic acid (**10d-1**)

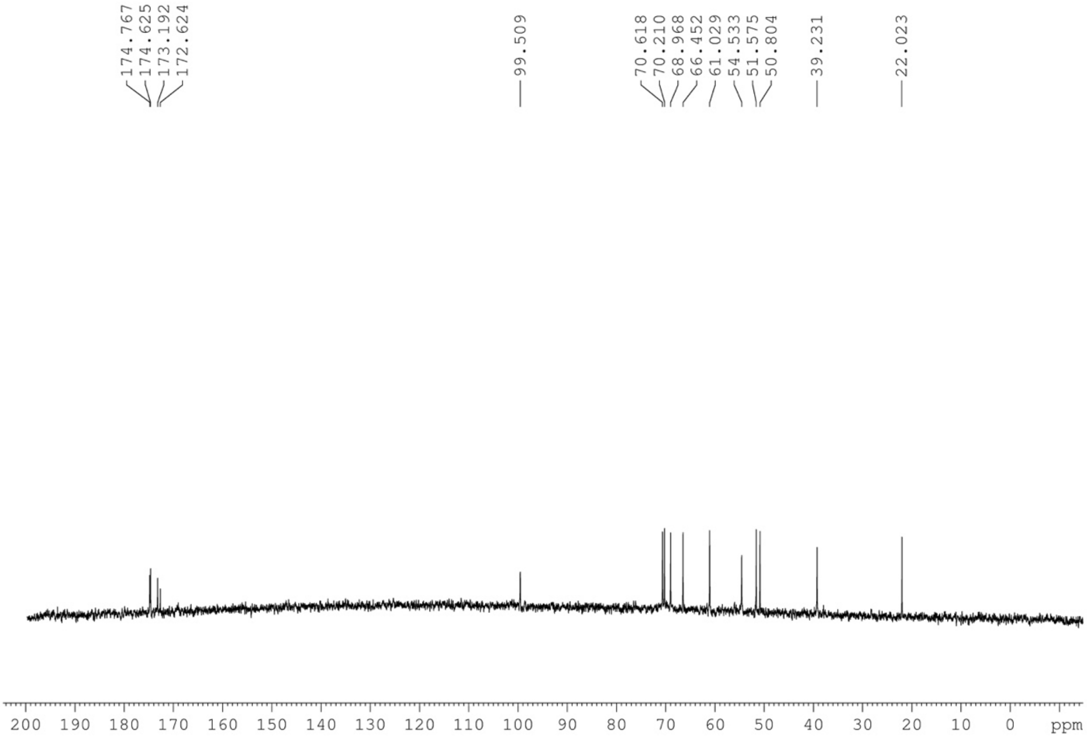

^{1}H NMR (D_2O): Methyl 5-acetamido-3,5-dideoxy-8-(D/L-serine)carboxamido-D-*glycero*-β-D-*galacto*-oct-2-ulopyranosidonic acid (**10d-2**)

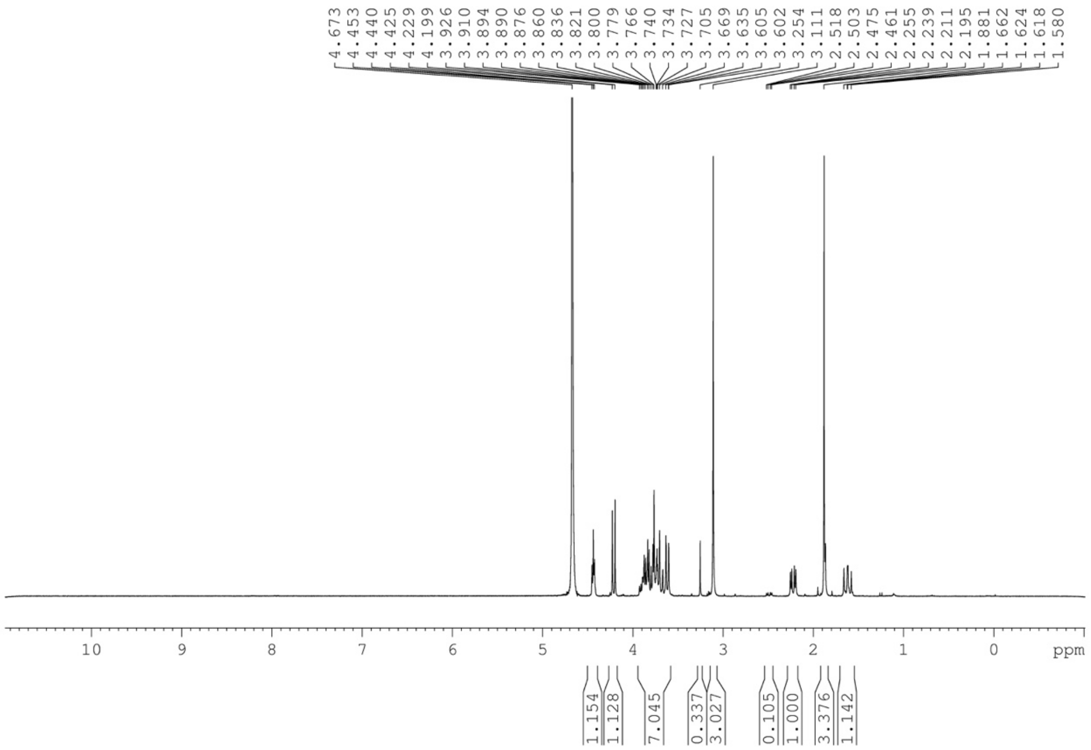

^{13}C NMR (D_2O): Methyl 5-acetamido-3,5-dideoxy-8-(D/L-serine)carboxamido-D-*glycero*-β-D-*galacto*-oct-2-ulopyranosidonic acid (**10d-2**)

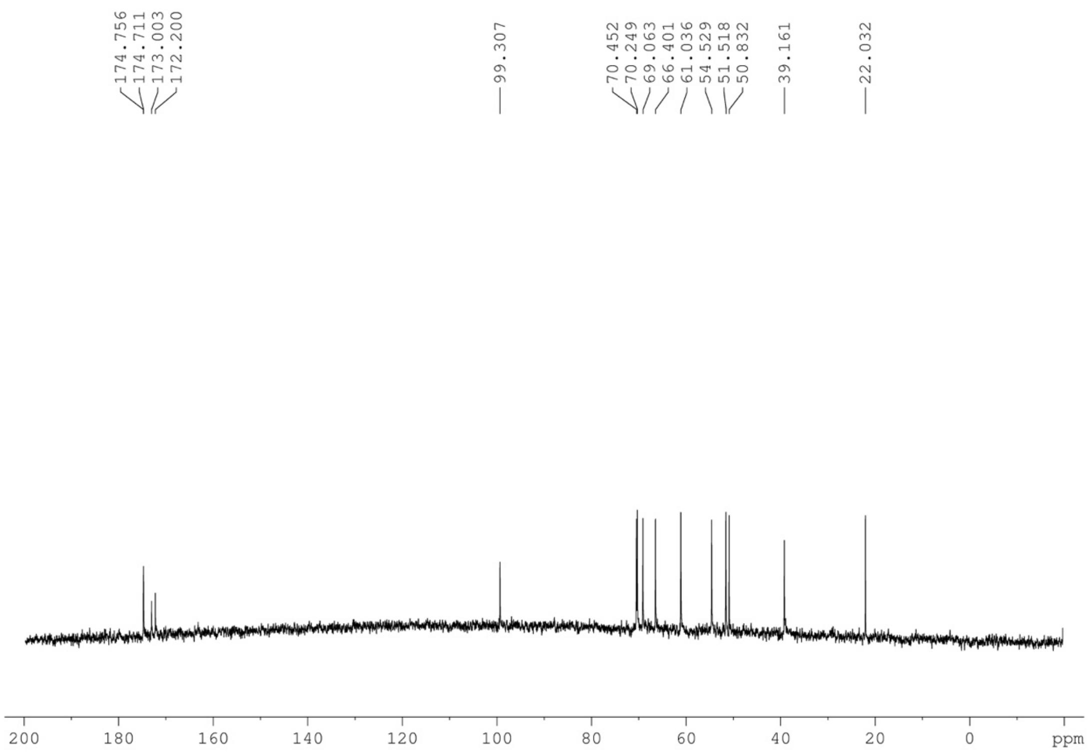

^{1}H NMR (D_2O): Methyl 5-acetamido-3,5-dideoxy-8-(L-phenylalanine)carboxamido-D-*glycero*-β-D-*galacto*-oct-2-ulopyranosidonic acid (**10e**)

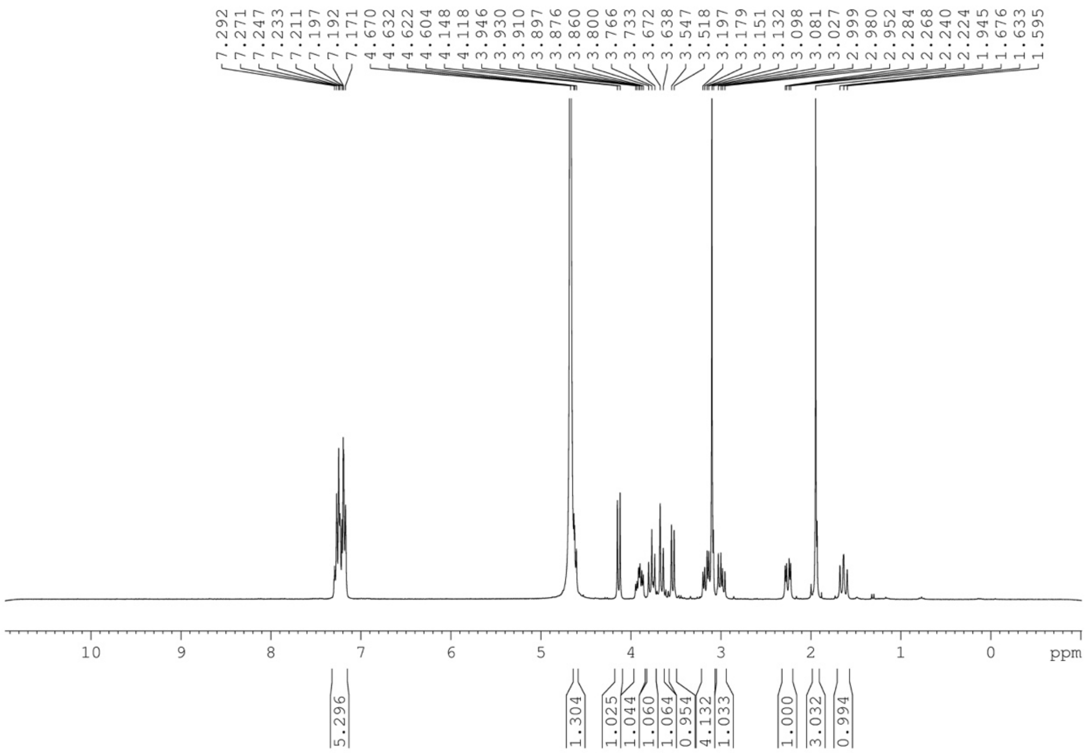

^{13}C NMR (D_2O): Methyl 5-acetamido-3,5-dideoxy-8-(L-phenylalanine)carboxamido-D-*glycero*-β-D-*galacto*-oct-2-ulopyranosidonic acid (**10e**)

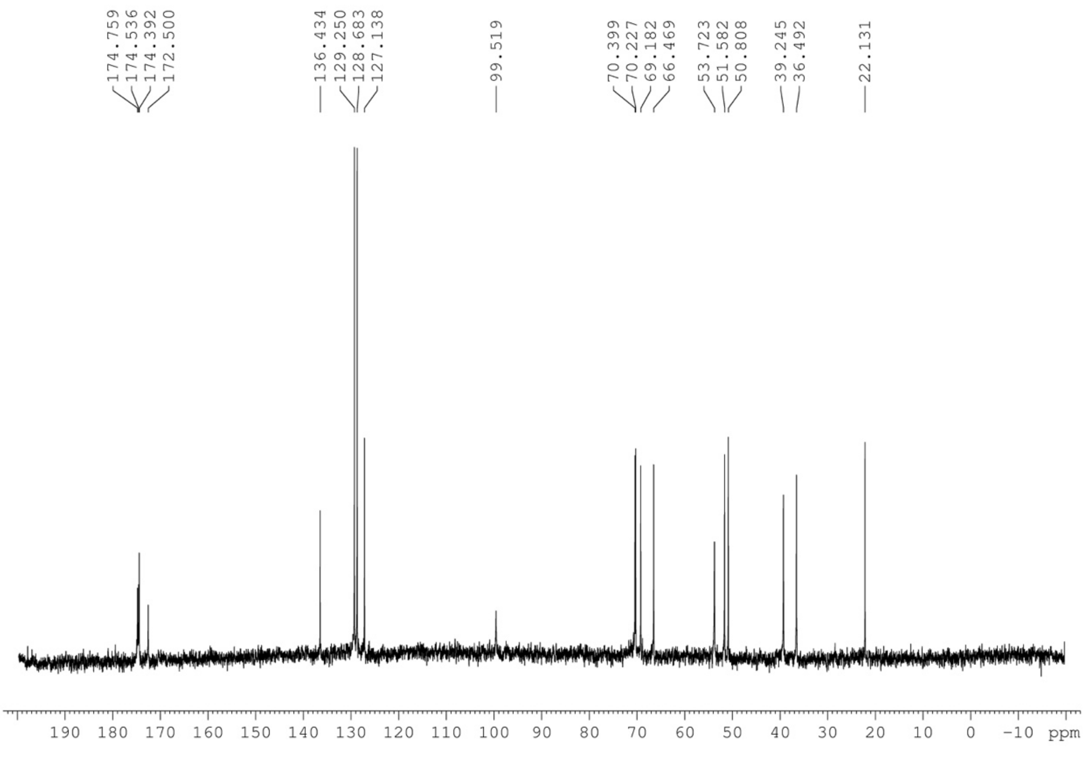

^{1}H NMR (D_2O): Methyl 5-acetamido-3,5-dideoxy-8-(L-tyrosine)carboxamido-D-*glycero*-β-D-*galacto*-oct-2-ulopyranosidonic acid (**10f**)

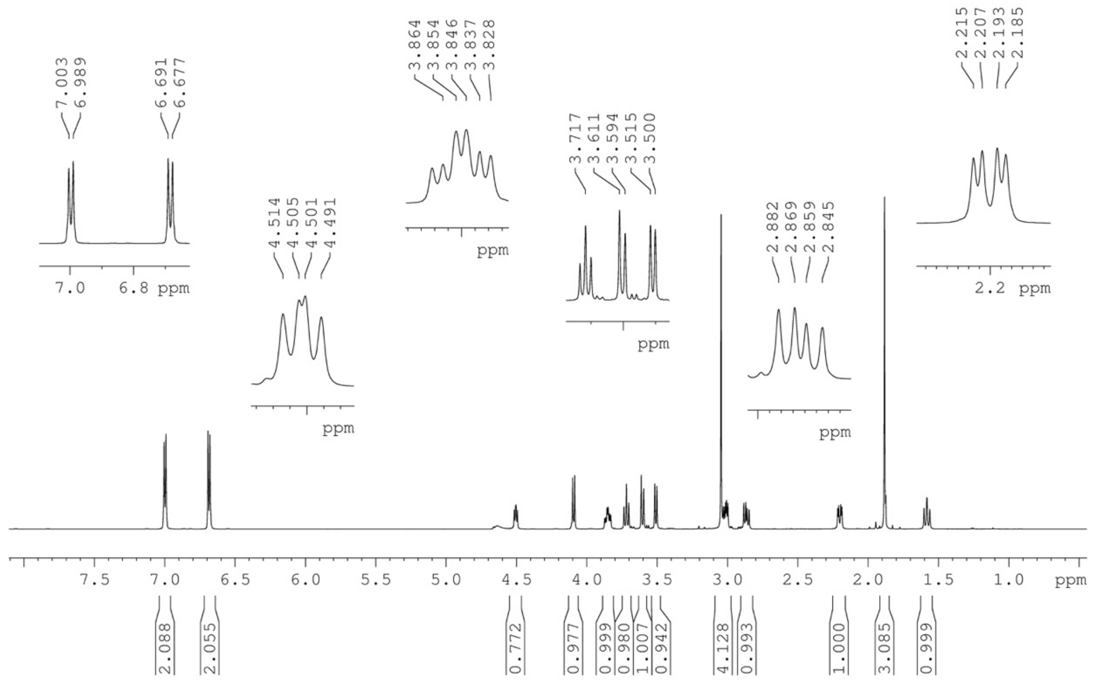

^{13}C NMR (D_2O): Methyl 5-acetamido-3,5-dideoxy-8-(L-tyrosine)carboxamido-D-*glycero*-β-D-*galacto*-oct-2-ulopyranosidonic acid (**10f**)

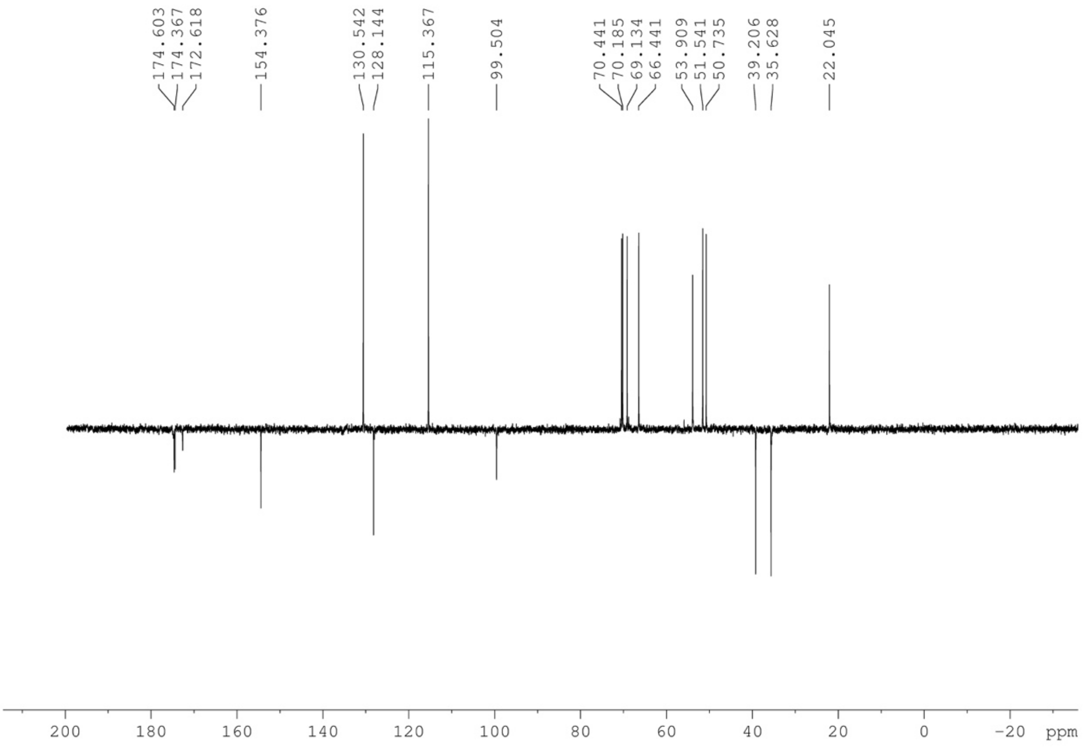

^{1}H NMR (D_2O): Methyl 5-acetamido-3,5-dideoxy-8-(L-histidine)carboxamido-D-*glycero*-β-D-*galacto*-oct-2-ulopyranosidonic acid (**10g**)

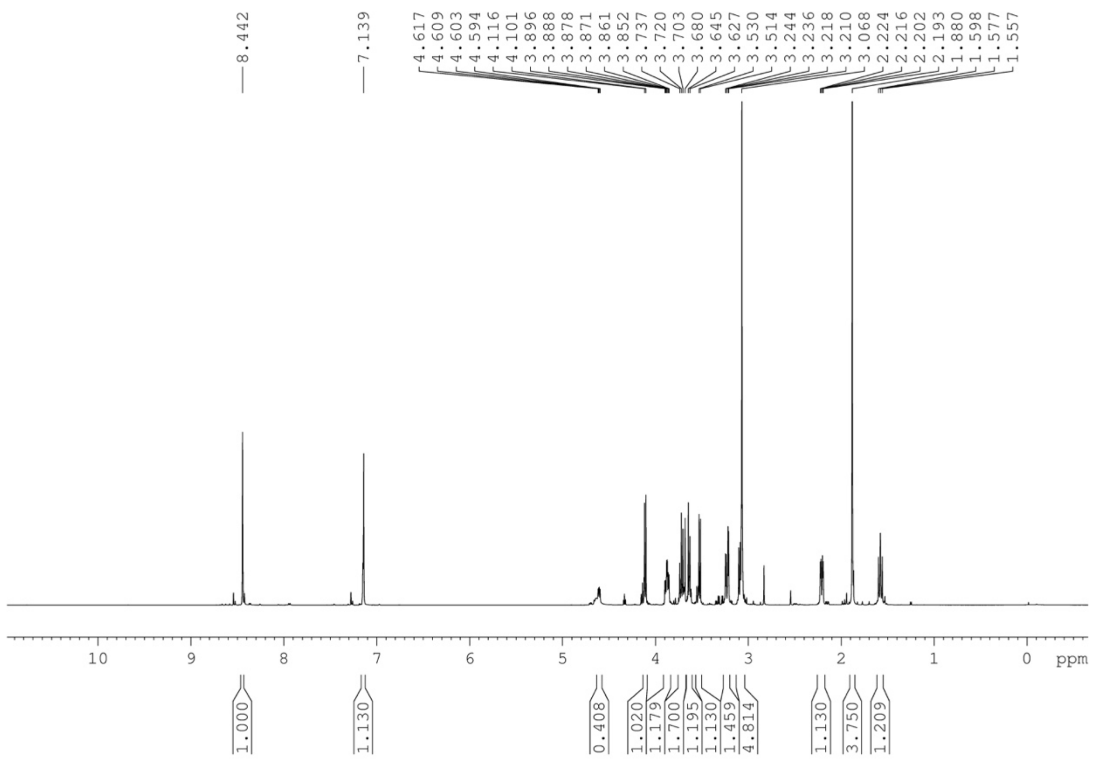

^{13}C NMR (D_2O): Methyl 5-acetamido-3,5-dideoxy-8-(L-histidine)carboxamido-D-*glycero*-β-D-*galacto*-oct-2-ulopyranosidonic acid (**10g**)

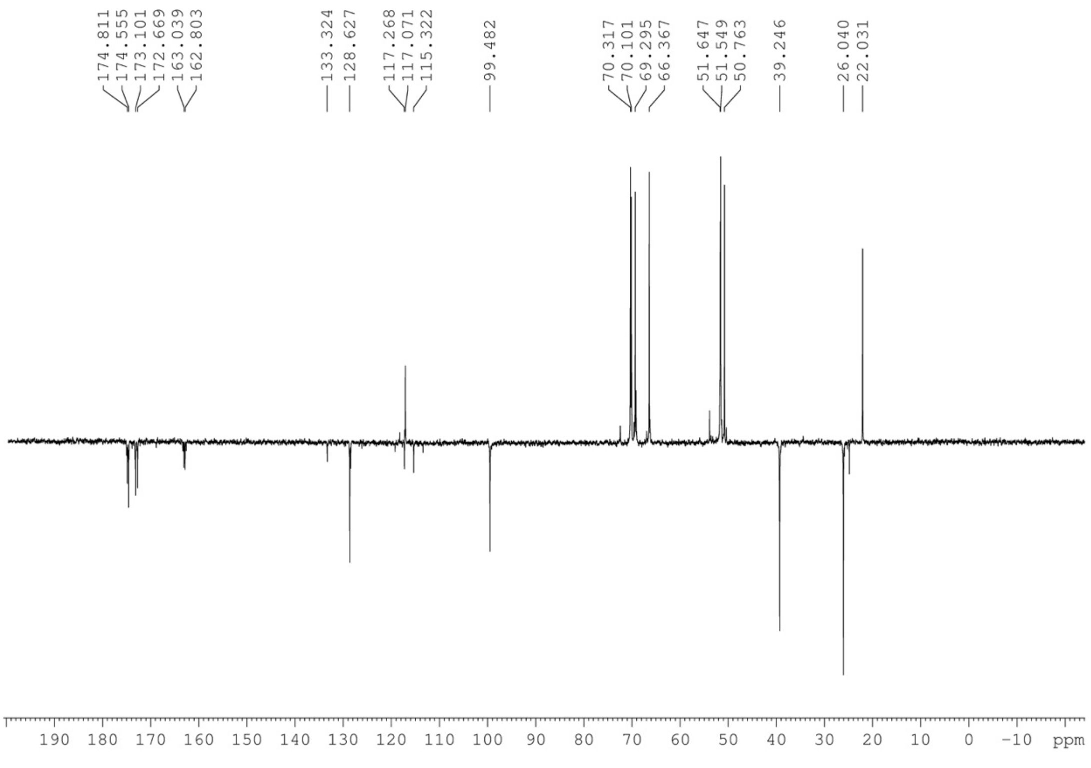

^{1}H NMR (D_2O): Methyl 5-acetamido-3,5-dideoxy-8-(L-tryptophan)carboxamido-D-*glycero*-β-D-*galacto*-oct-2-ulopyranosidonic acid (**10h**)

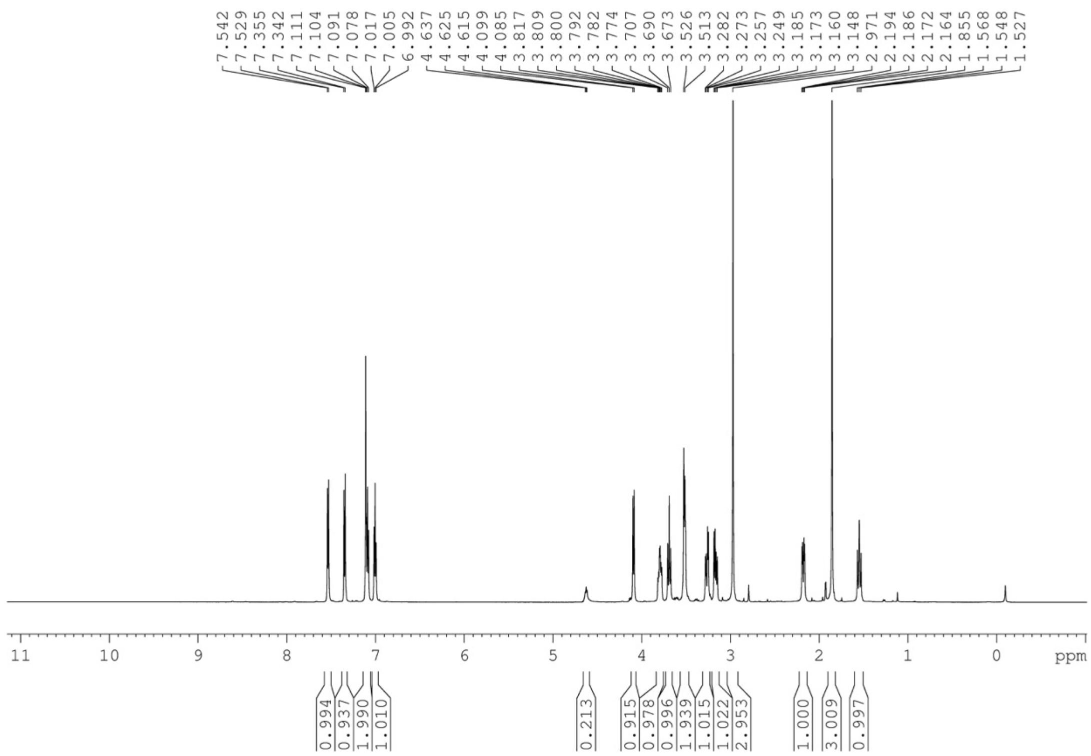

^{13}C NMR (D_2O): Methyl 5-acetamido-3,5-dideoxy-8-(L-tryptophan)carboxamido-D-*glycero*-β-D-*galacto*-oct-2-ulopyranosidonic acid (**10h**)

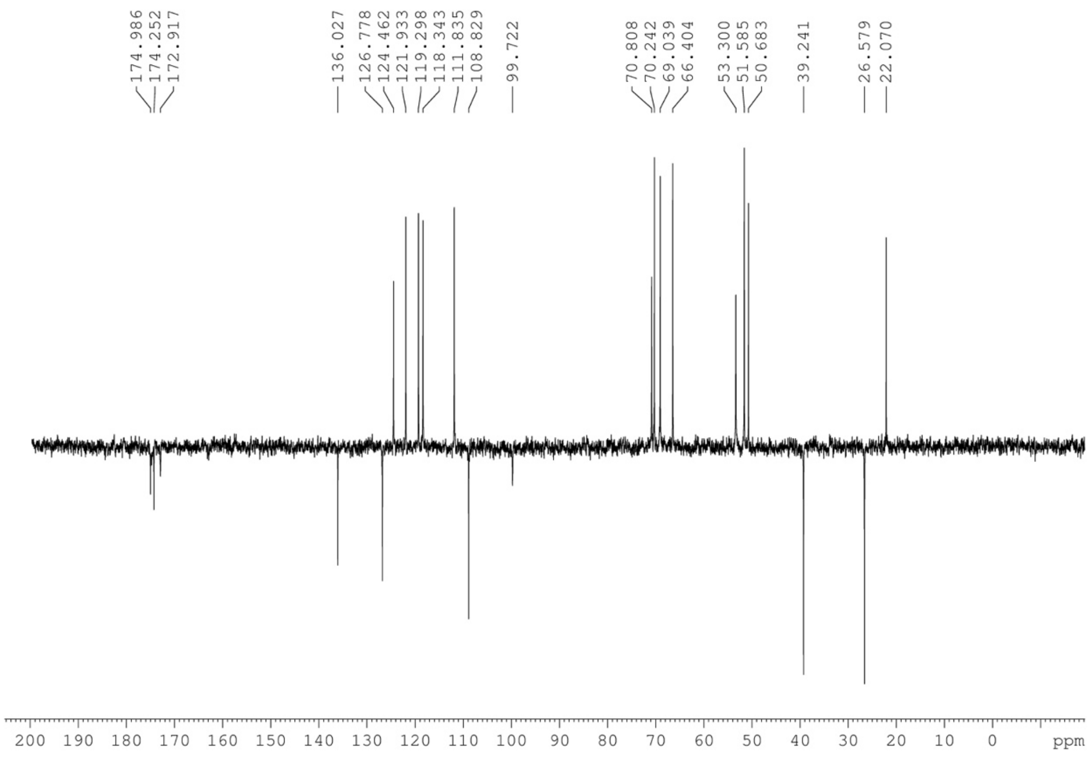

^{1}H NMR ($CDCl_3$): Methyl (methyl 5-acetamido-3,5-dideoxy-8,9-*O*-isopropylidene-D-*glycero*-β-D-*galacto*-2-nonulopyranosid)onate (**11**)

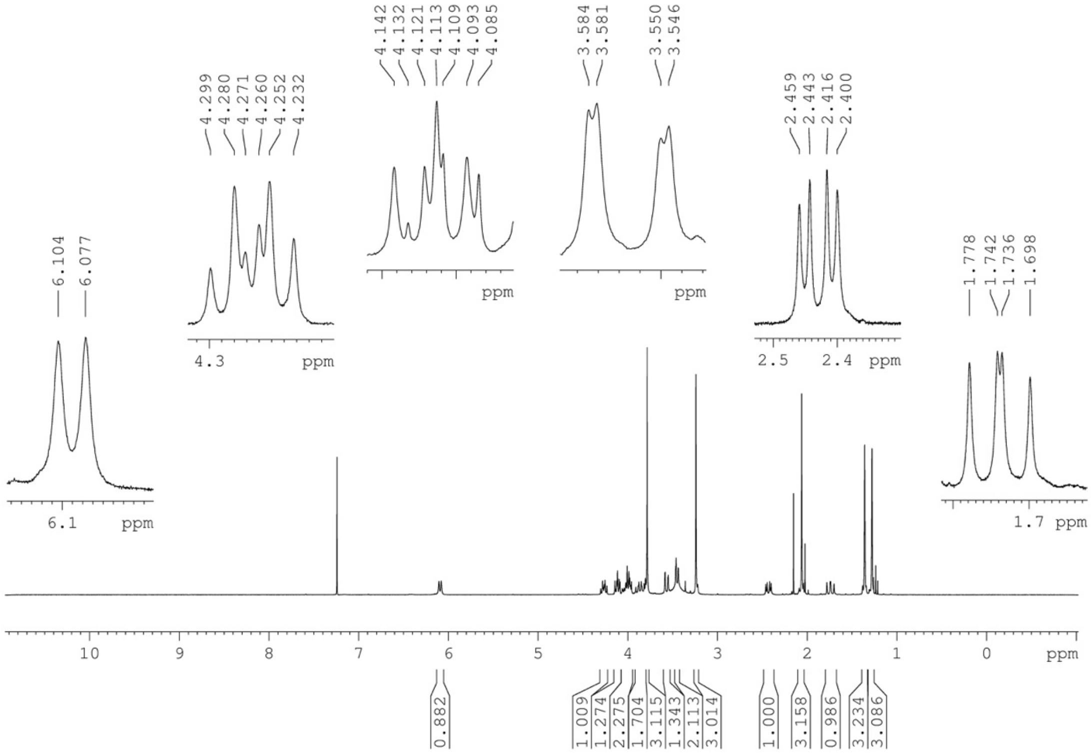

^{1}H NMR ($CDCl_3$): Methyl (methyl 5-acetamido-4-*O*-*tert*-butyldimethylsilyl-3,5-dideoxy-8,9-*O*-isopropylidene-D-*glycero*-β-D-*galacto*-2-nonulopyranosid)onate (**12**)

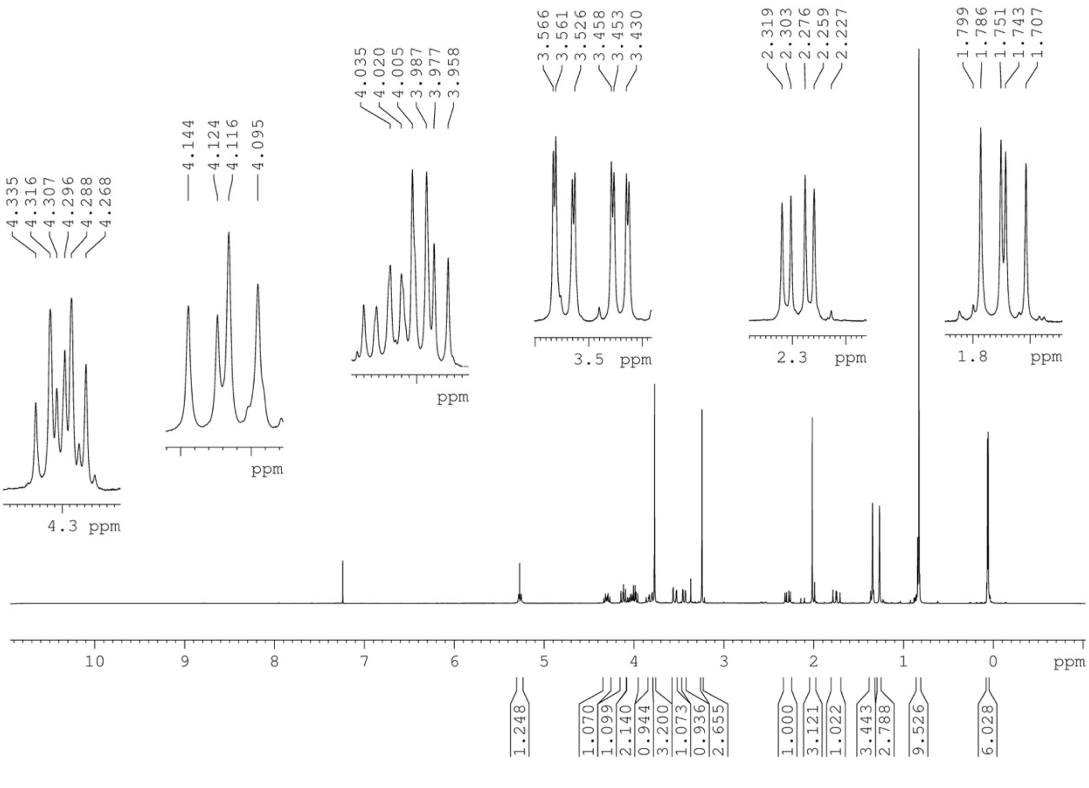

^{1}H NMR ($CDCl_3$): Ethyl (methyl 5-acetamido-4-*O*-*tert*-butyldimethylsilyl-3,5-dideoxy-7-*O*-ethyl-8,9-*O*-isopropylidene-D-*glycero*-β-D-*galacto*-2-nonulopyranosid)onate (**13a**)

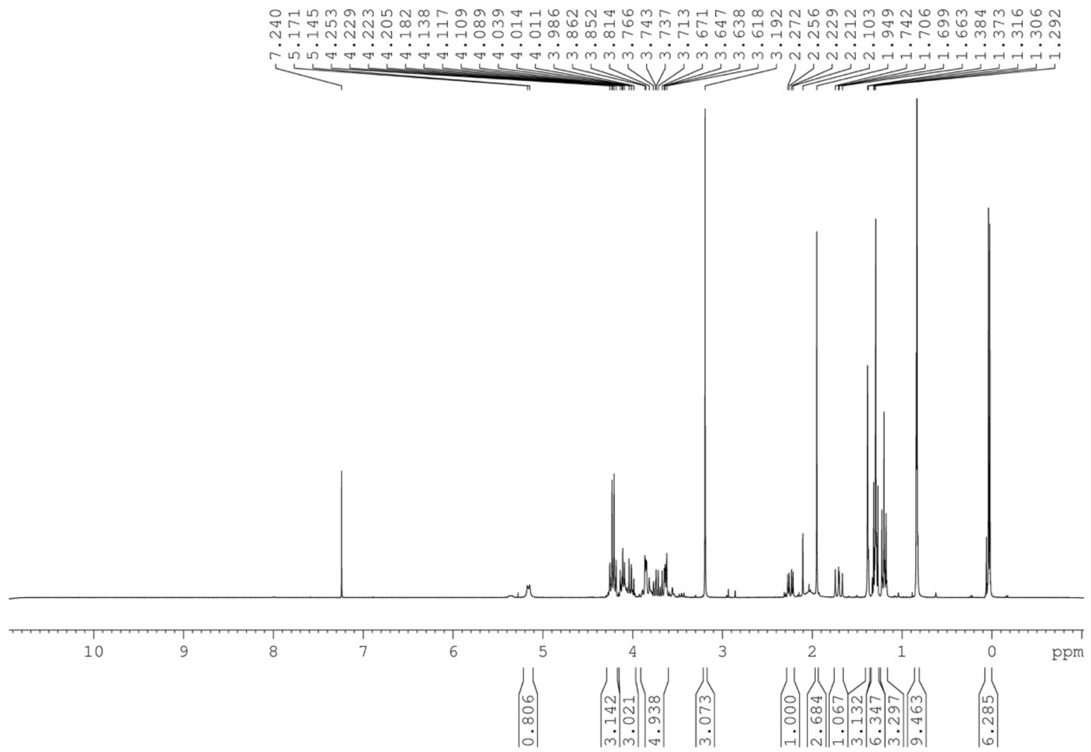

^{13}C NMR ($CDCl_3$): Ethyl (methyl 5-acetamido-4-*O*-*tert*-butyldimethylsilyl-3,5-dideoxy-7-*O*-ethyl-8,9-*O*-isopropylidene-D-*glycero*-β-D-*galacto*-2-nonulopyranosid)onate (**13a**)

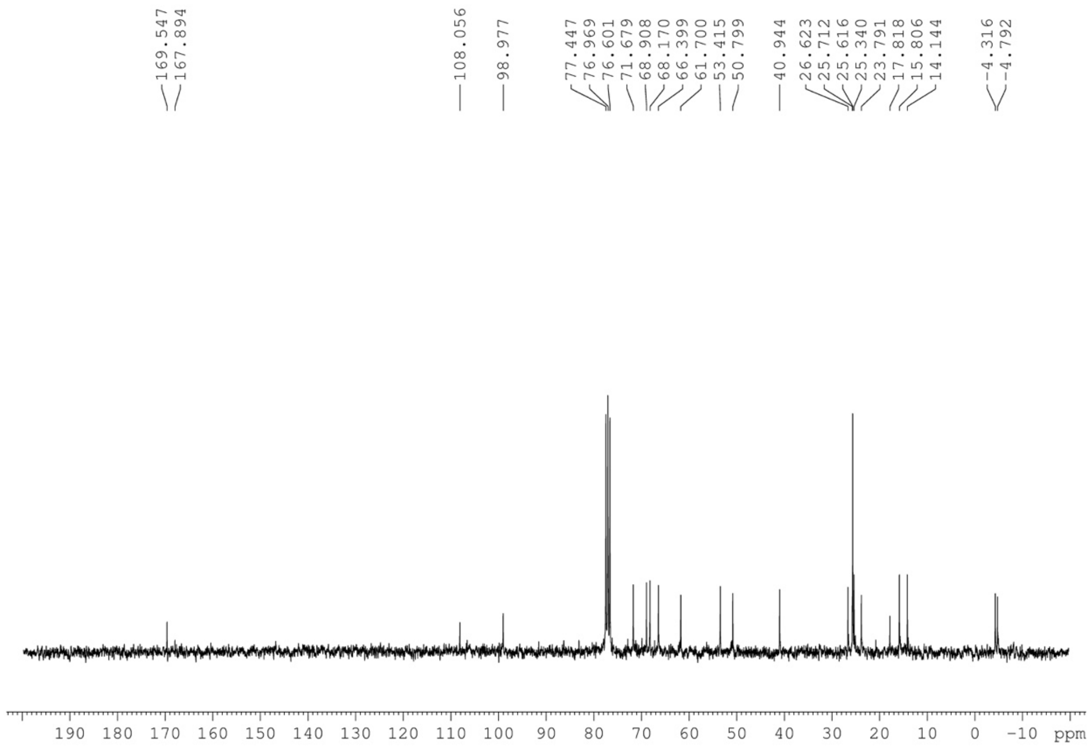

^{1}H NMR (CDCl$_3$): Methyl/Prop-2-enyl [methyl 5-acetamido-4-*O*-*tert*-butyldimethylsilyl-3,5-dideoxy-8,9-*O*-isopropylidene-7-*O*-(prop-2-enyl) -D-*glycero*-β-D-*galacto*-2-nonulopyranosid]onate (**13b**)

[Note: an inseparable impurity of allyl alcohol was present in the isolated product.]

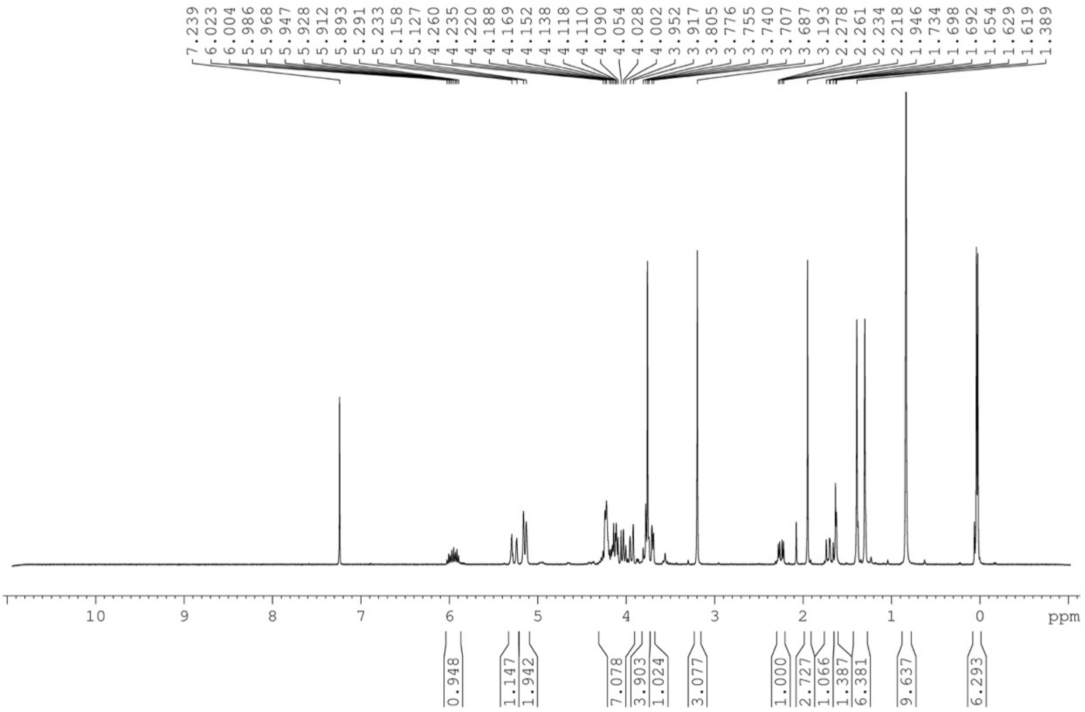

^{13}C NMR (CDCl$_3$): Methyl/Prop-2-enyl [methyl 5-acetamido-4-*O*-*tert*-butyldimethylsilyl-3,5-dideoxy-8,9-*O*-isopropylidene-7-*O*-(prop-2-enyl)-D-*glycero*-β-D-*galacto*-2-nonulopyranosid]onate (**13b**)

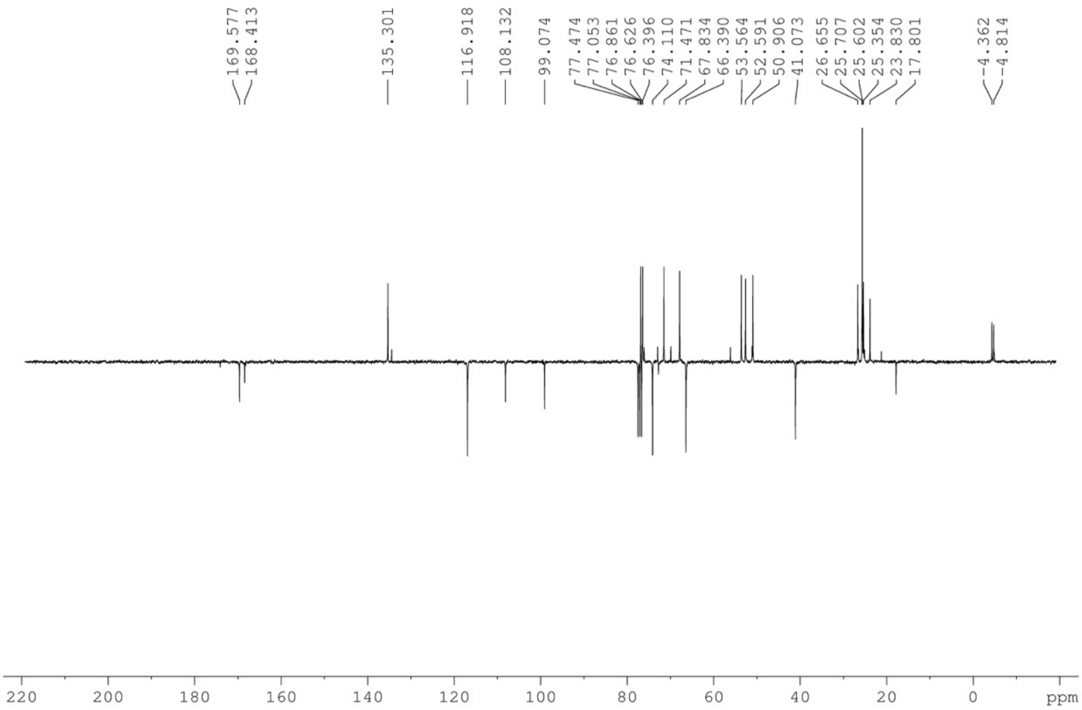

^{1}H NMR ($CDCl_3$): Methyl (methyl 5-acetamido-4-*O*-*tert*-butyldimethylsilyl-3,5-dideoxy-8,9-*O*-isopropylidene-7-*O*-propyl-D-*glycero*-β-D-*galacto*-2-nonulopyranosid)onate (**13c**)

[Note: an impurity was present in this product; resonances at δ 1.65 and 3.60 ppm]

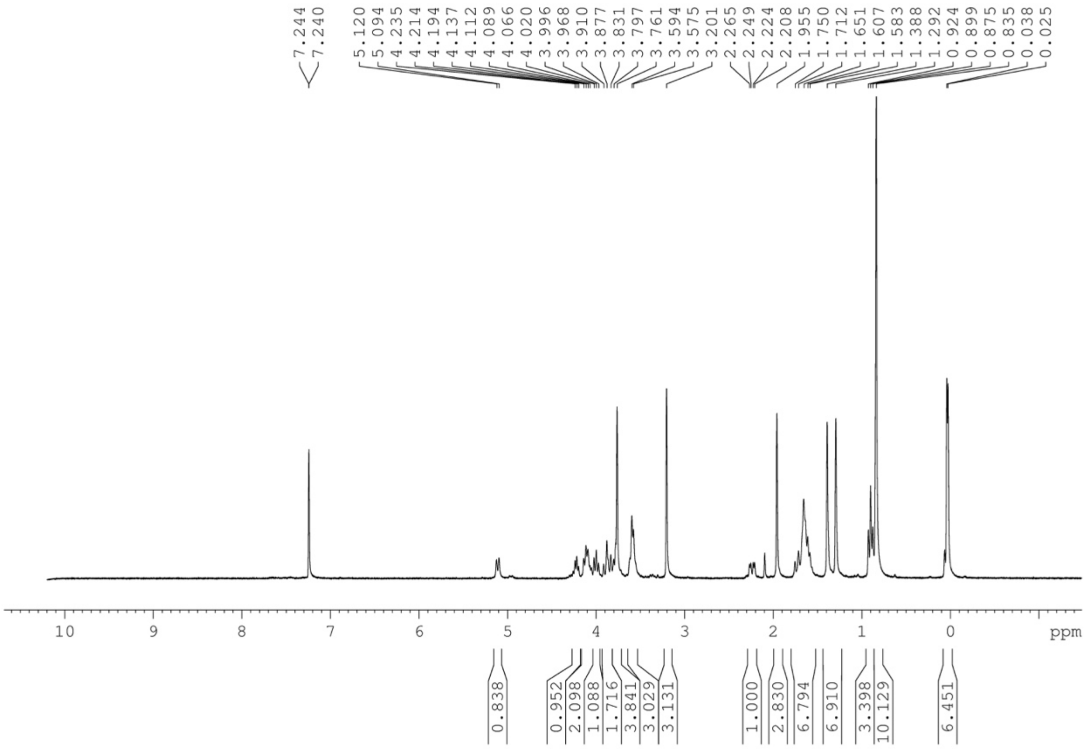

^{13}C NMR ($CDCl_3$): Methyl (methyl 5-acetamido-4-*O*-*tert*-butyldimethylsilyl-3,5-dideoxy-8,9-*O*-isopropylidene-7-*O*-propyl-D-*glycero*-β-D-*galacto*-2-nonulopyranosid)onate (**13c**)

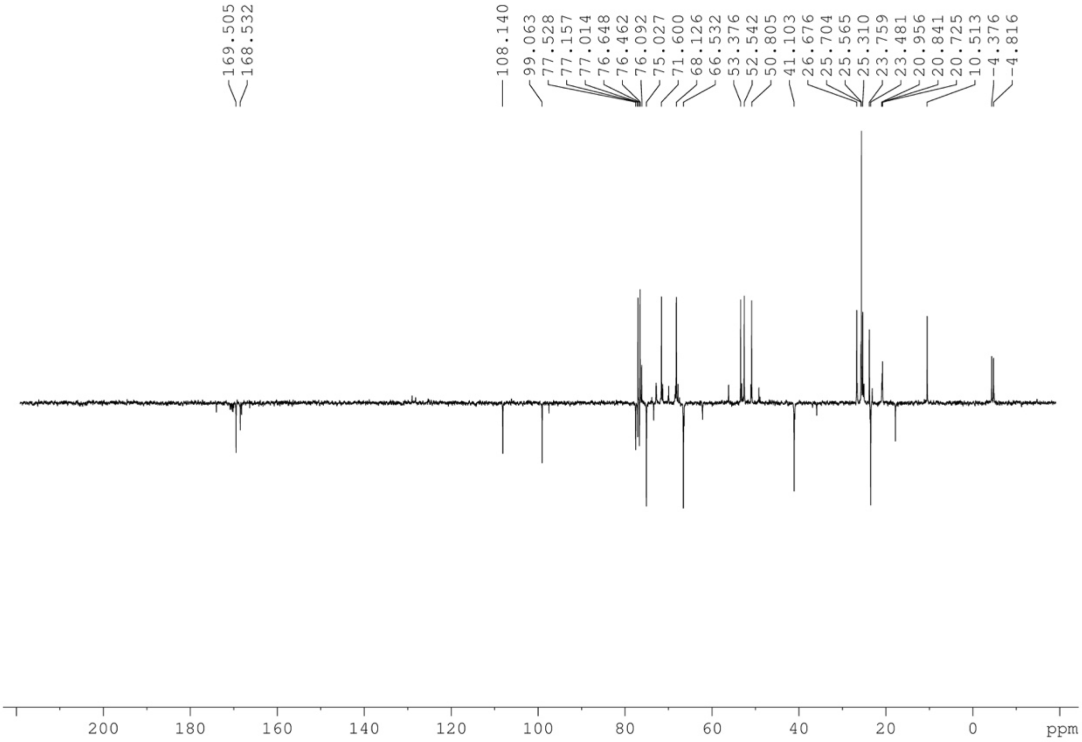

^{1}H NMR ($CDCl_3$): Methyl/Prop-2-ynyl [methyl 5-acetamido-4-*O*-*tert*-butyldimethylsilyl-3,5-dideoxy-8,9-*O*-isopropylidene-7-*O*-(prop-2-ynyl)-D-*glycero*-β-D-*galacto*-2-nonulopyranosid]onate (**13d**)

[Note: mixed ester sample.]

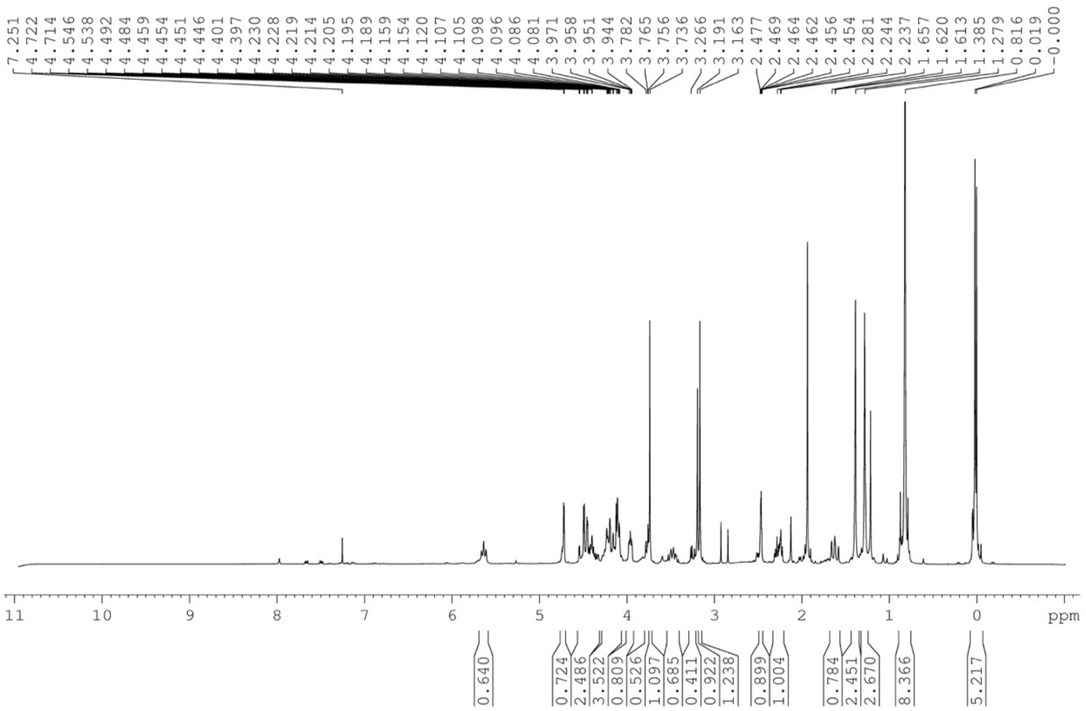

^{13}C NMR ($CDCl_3$): Methyl/Prop-2-ynyl [methyl 5-acetamido-4-*O*-*tert*-butyldimethylsilyl-3,5-dideoxy-8,9-*O*-isopropylidene-7-*O*-(prop-2-ynyl)-D-*glycero*-β-D-*galacto*-2-nonulopyranosid]onate (**13d**)

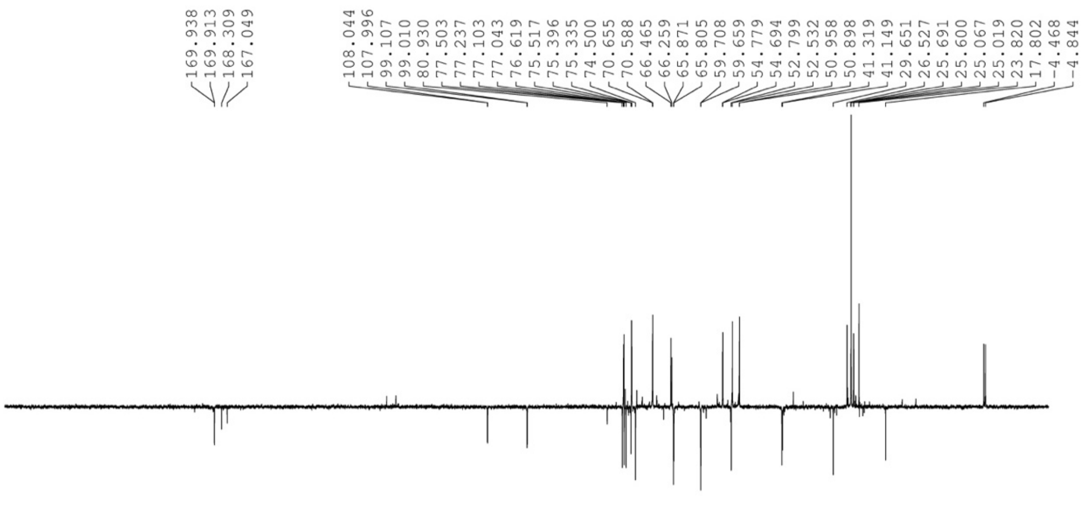

^{1}H NMR ($CDCl_3$): Benzyl (methyl 5-acetamido-7-*O*-benzyl-4-*O*-*tert*-butyldimethylsilyl-3,5-dideoxy-8,9-*O*-isopropylidene-D-*glycero*-β-D-*galacto*-non-2-ulopyranosid)onate (**13e**)

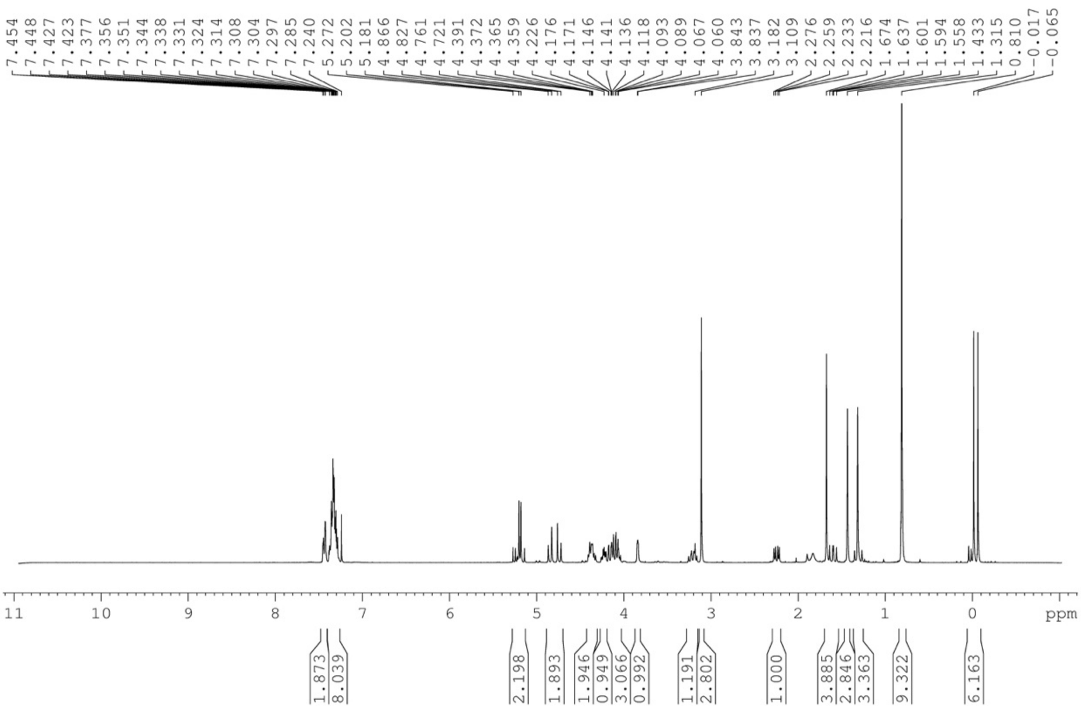

^{13}C NMR ($CDCl_3$): Benzyl (methyl 5-acetamido-7-*O*-benzyl-4-*O*-*tert*-butyldimethylsilyl-3,5-dideoxy-8,9-*O*-isopropylidene-D-*glycero*-β-D-*galacto*-non-2-ulopyranosid)onate (**13e**)

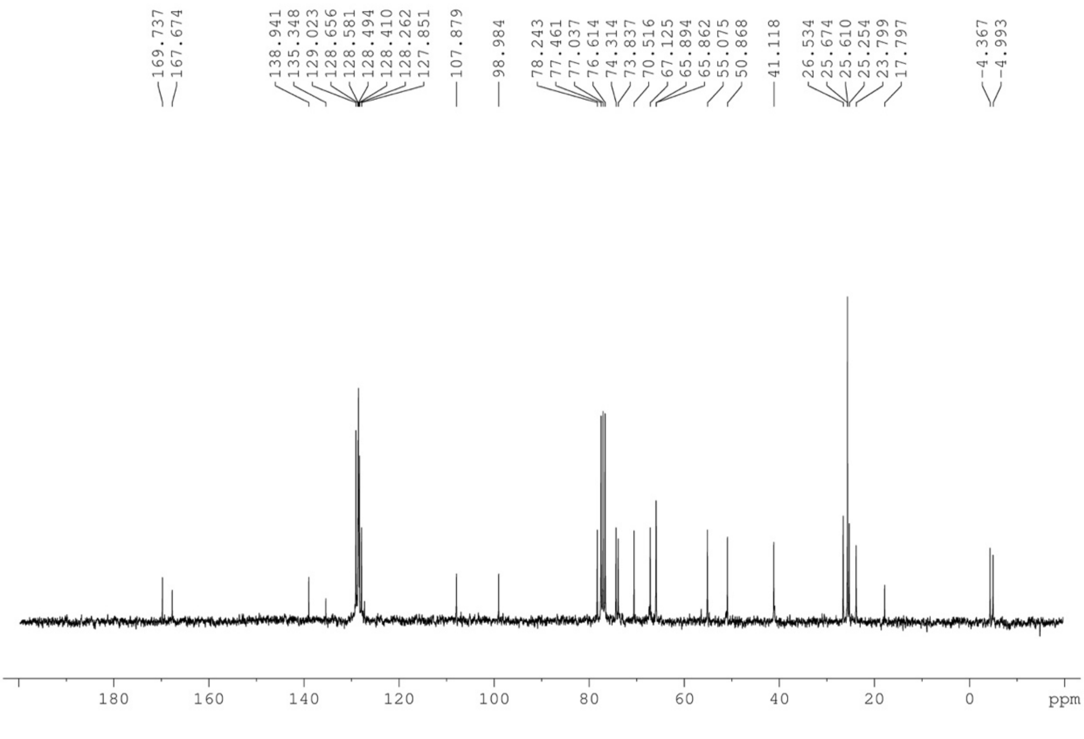

^{1}H NMR (D_2O): Methyl 5-acetamido-3,5-dideoxy-7-*O*-ethyl-D-*glycero*-β-D-*galacto*-non-2-ulopyranosidonic acid (**14a**)

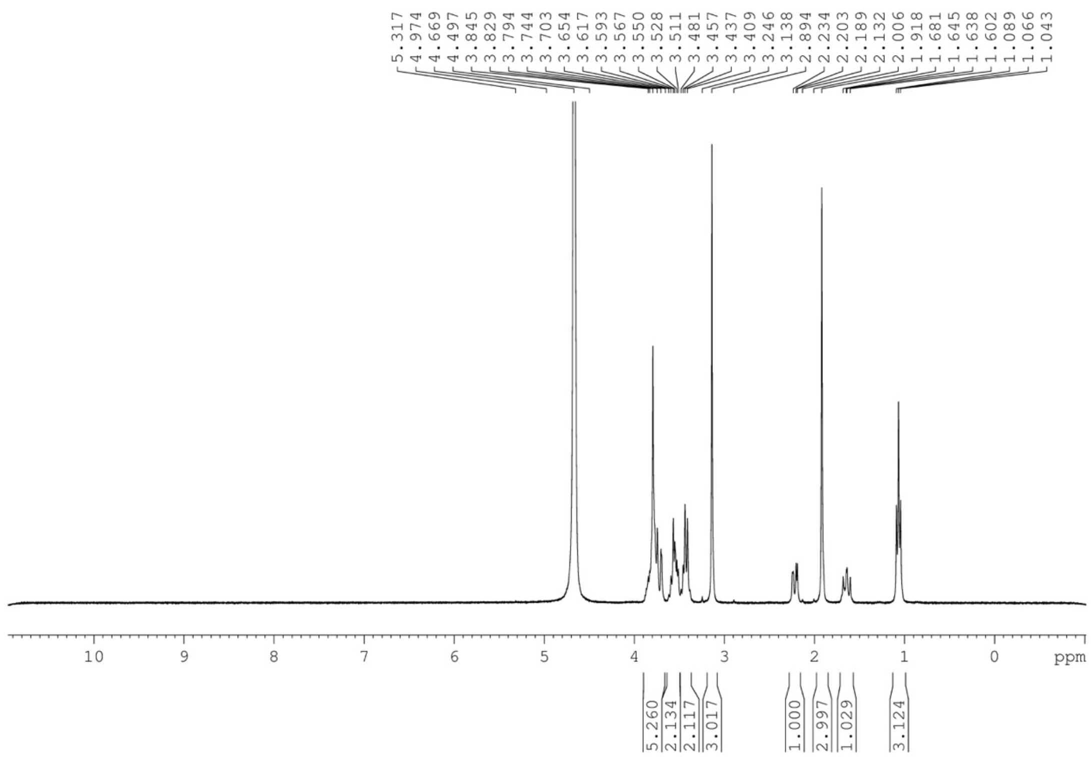

^{13}C NMR (D_2O): Methyl 5-acetamido-3,5-dideoxy-7-*O*-ethyl-D-*glycero*-β-D-*galacto*-non-2-ulopyranosidonic acid (**14a**)

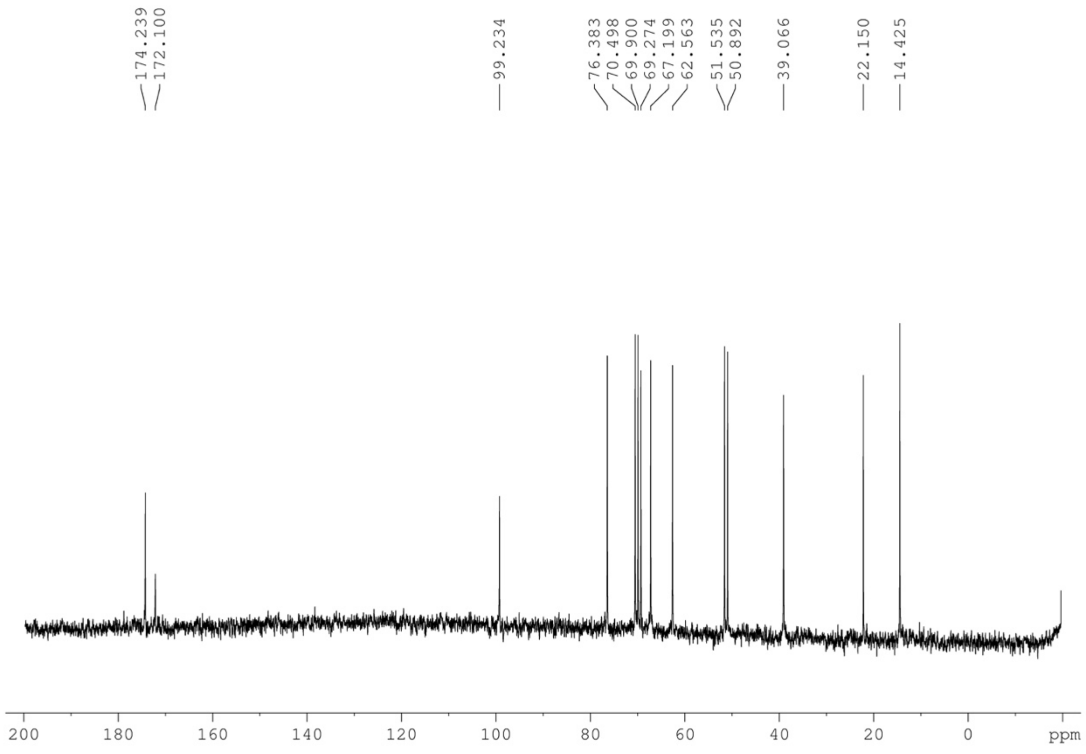

^{1}H NMR (D_2O): Methyl 5-acetamido-3,5-dideoxy-7-*O*-(prop-2-enyl)-D-*glycero*-β-D-*galacto*-non-2-ulopyranosidonic acid (**14b**)

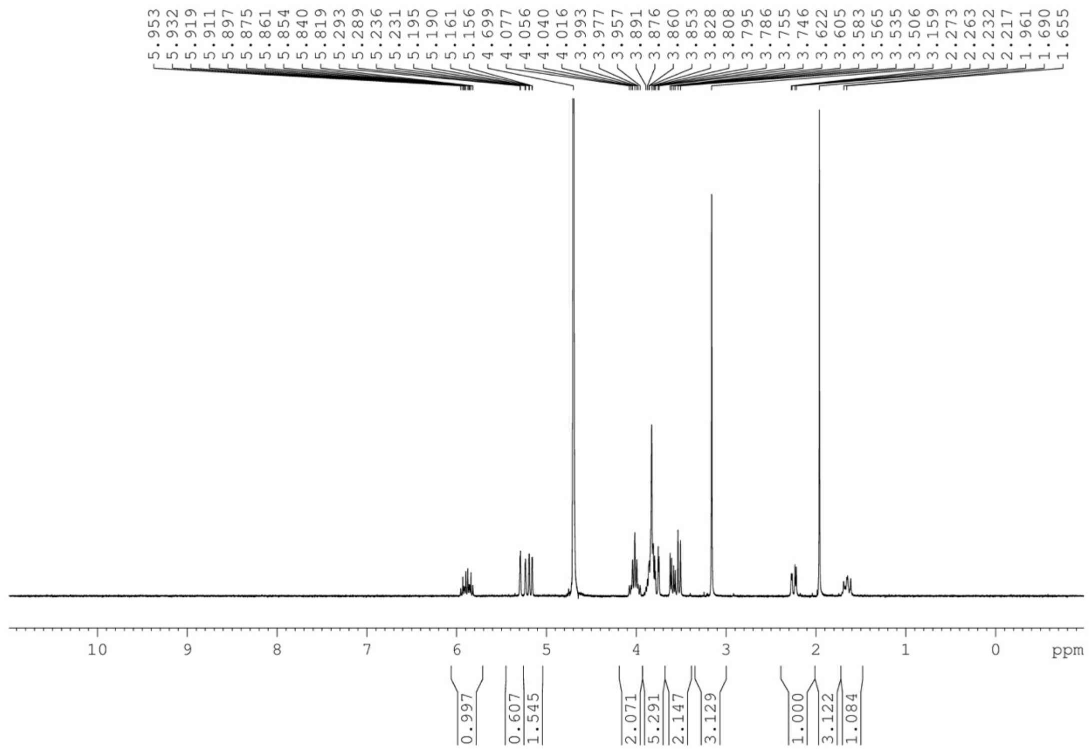

^{13}C NMR (D_2O): Methyl 5-acetamido-3,5-dideoxy-7-*O*-(prop-2-enyl)-D-*glycero*-β-D-*galacto*-non-2-ulopyranosidonic acid (**14b**)

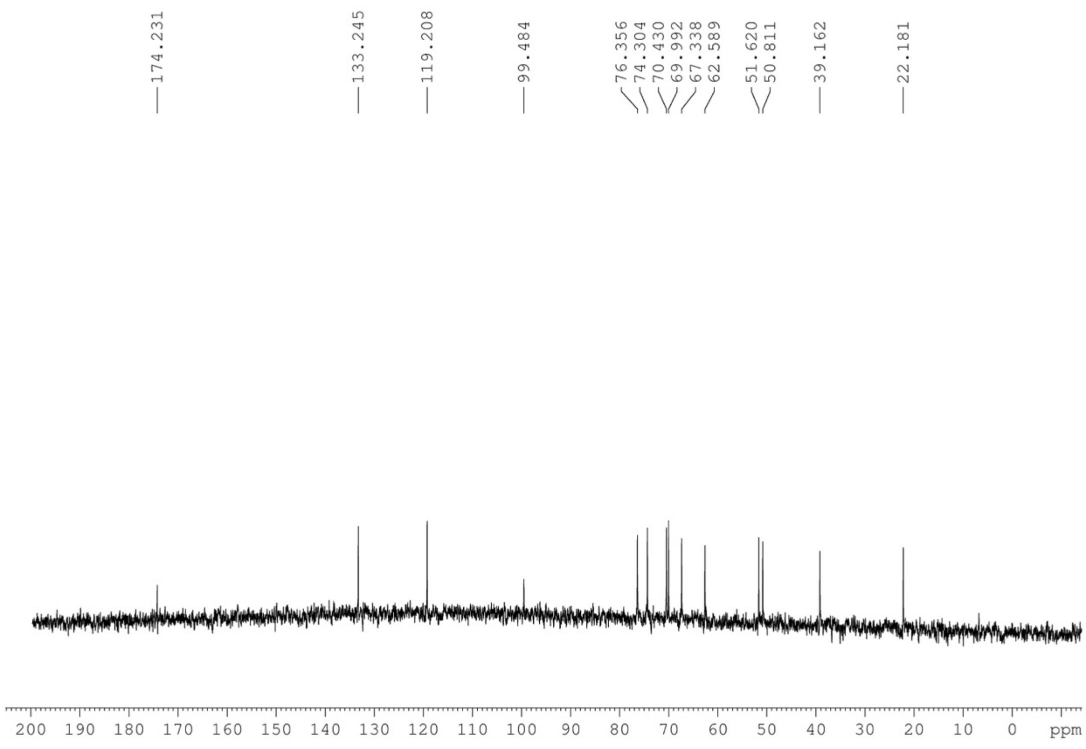

^{1}H NMR (D_2O): Methyl 5-acetamido-3,5-dideoxy-7-*O*-propyl-D-*glycero*-β-D-*galacto*-non-2-ulopyranosidonic acid, sodium salt (**14c**)

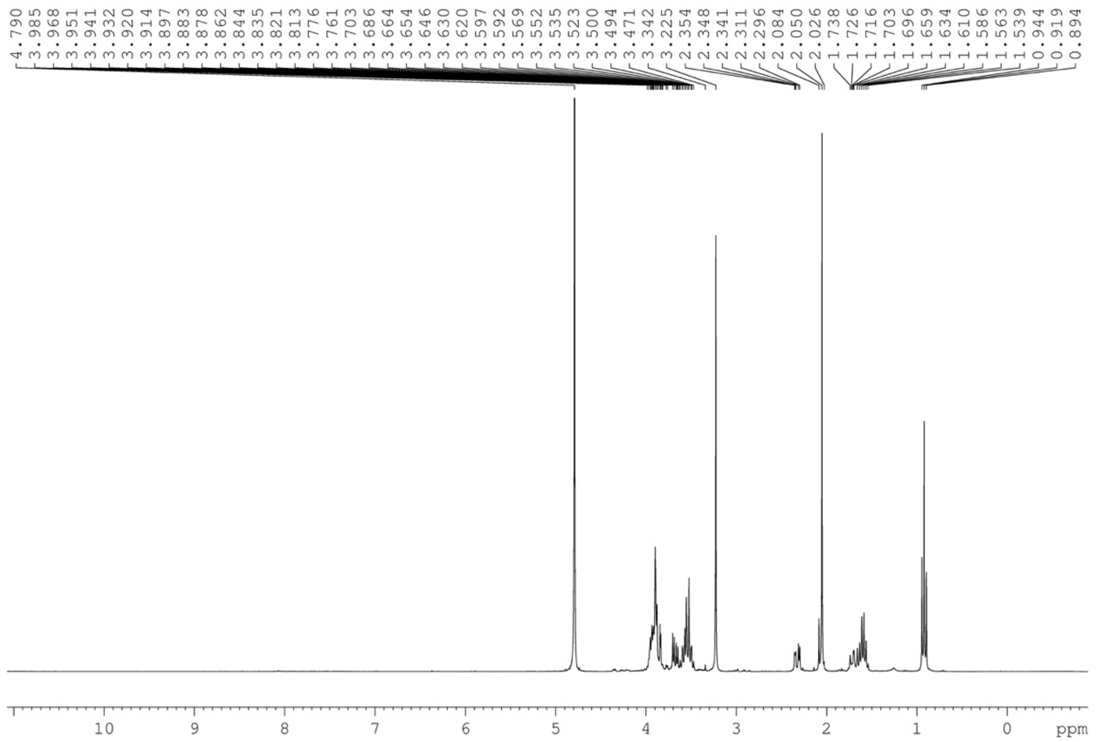

^{13}C NMR (D_2O): Methyl 5-acetamido-3,5-dideoxy-7-*O*-propyl-D-*glycero*-β-D-*galacto*-non-2-ulopyranosidonic acid, sodium salt (**14c**)

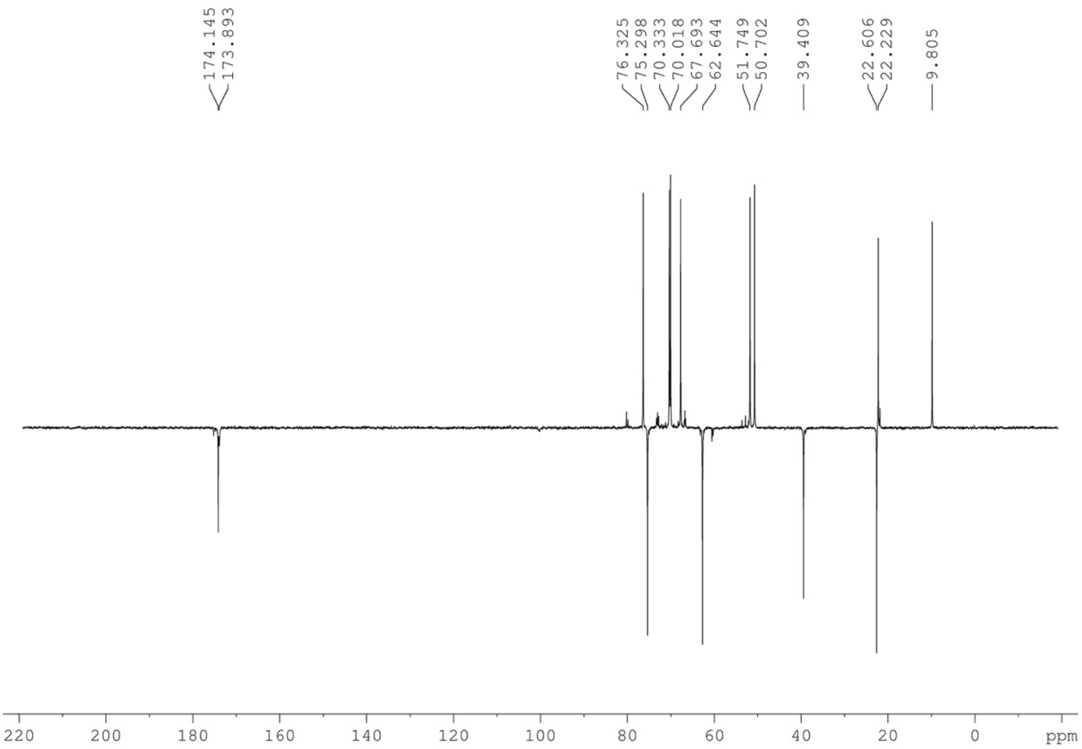

^{1}H NMR (D_2O): Methyl 5-acetamido-3,5-dideoxy-7-*O*-(prop-2-ynyl)-D-*glycero*-β-D-*galacto*-non-2-ulopyranosidonic acid, sodium salt (**14d**)

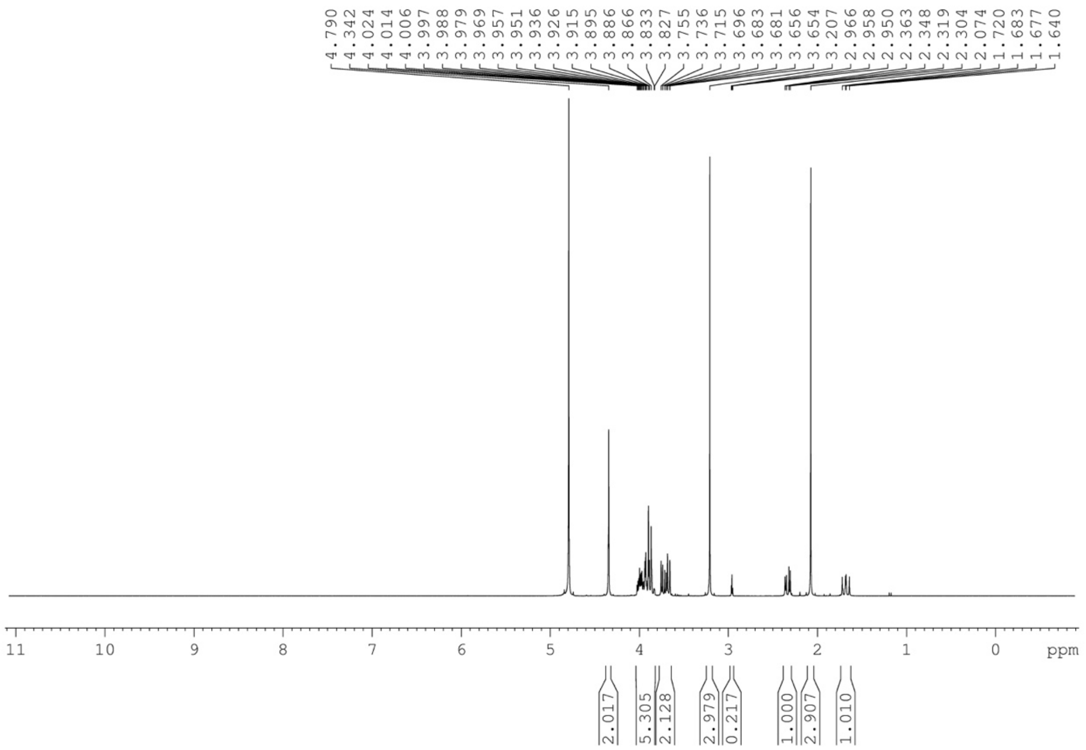

^{13}C NMR (D_2O): Methyl 5-acetamido-3,5-dideoxy-7-*O*-(prop-2-ynyl)-D-*glycero*-β-D-*galacto*-non-2-ulopyranosidonic acid, sodium salt (**14d**)

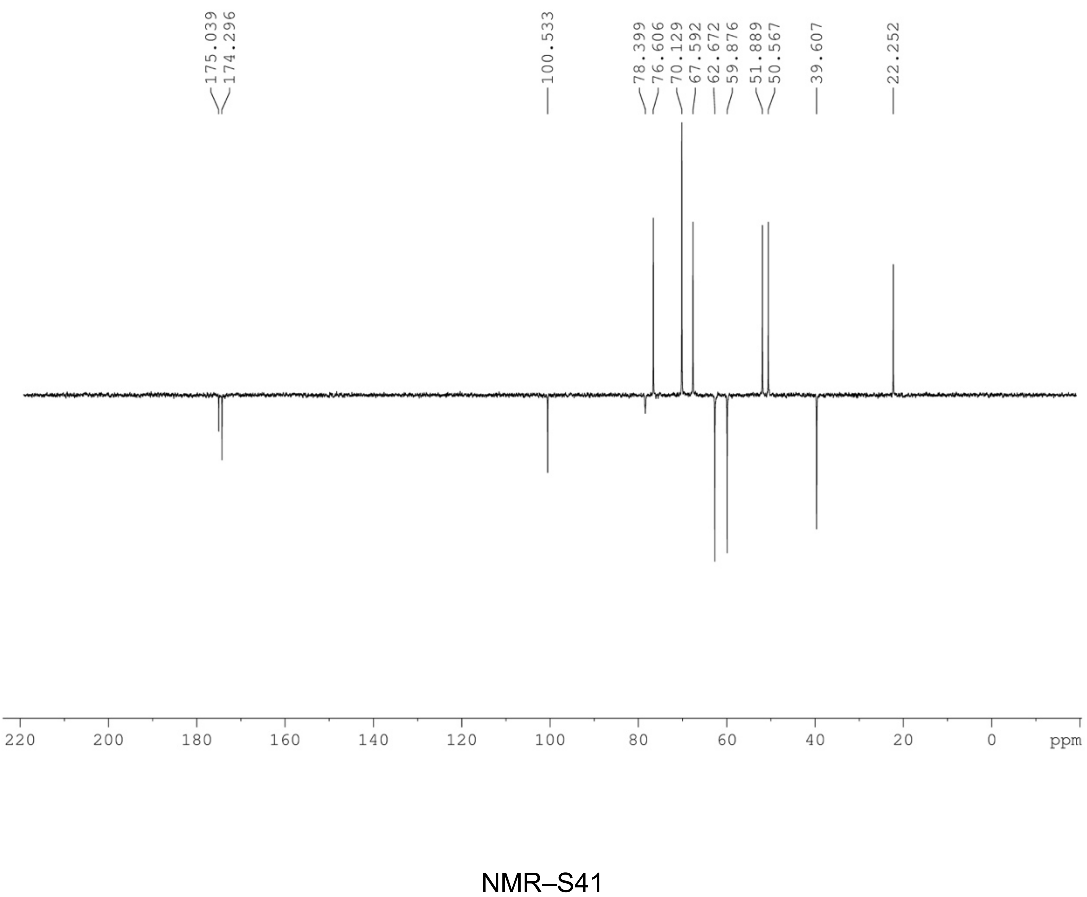

^{1}H NMR (D_2O): Methyl 5-acetamido-7-*O*-benzyl-3,5-dideoxy-D-*glycero*-β-D-*galacto*-non-2-ulopyranosidonic acid (**14e**)

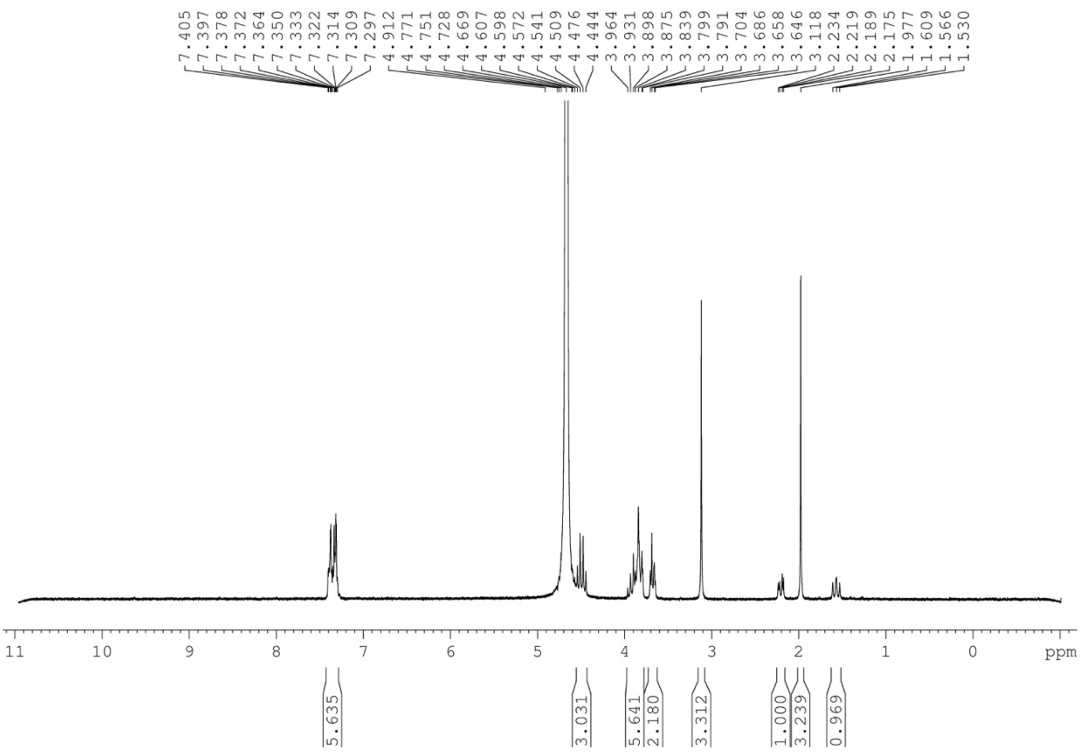

^{13}C NMR (D_2O): Methyl 5-acetamido-7-*O*-benzyl-3,5-dideoxy-D-*glycero*-β-D-*galacto*-non-2-ulopyranosidonic acid (**14e**)

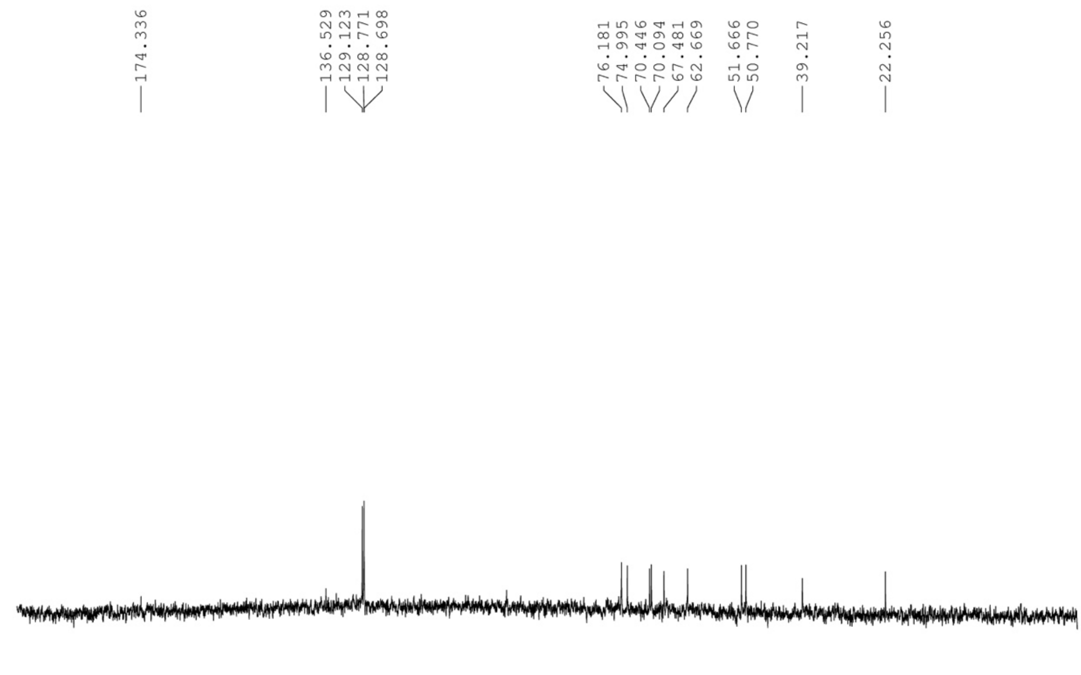

^{1}H NMR ($CDCl_3$): Methyl [methyl 5-acetamido-4,8,9-tri-*O*-acetyl-3,5-dideoxy-7-*O*-(prop-2-ynyl)-D-*glycero*-β-D-*galacto*-non-2-ulopyranosid]onate (**15**)

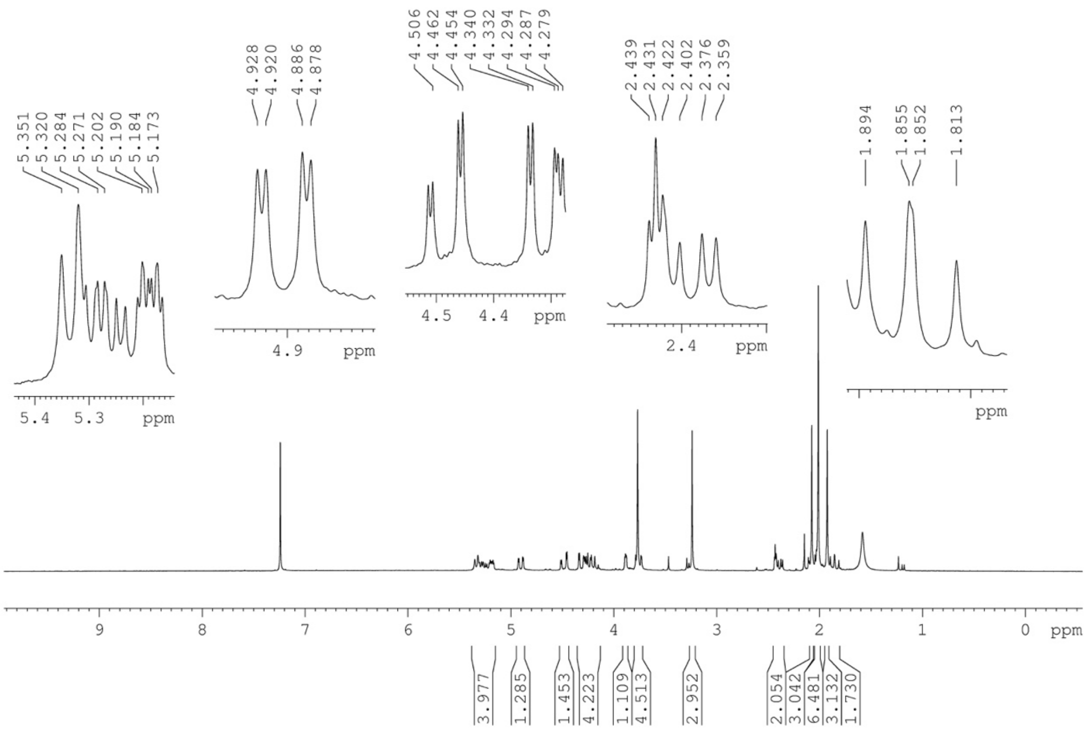

^{13}C NMR ($CDCl_3$): Methyl [methyl 5-acetamido-4,8,9-tri-*O*-acetyl-3,5-dideoxy-7-*O*-(prop-2-ynyl)-D-*glycero*-β-D-*galacto*-non-2-ulopyranosid]onate (**15**)

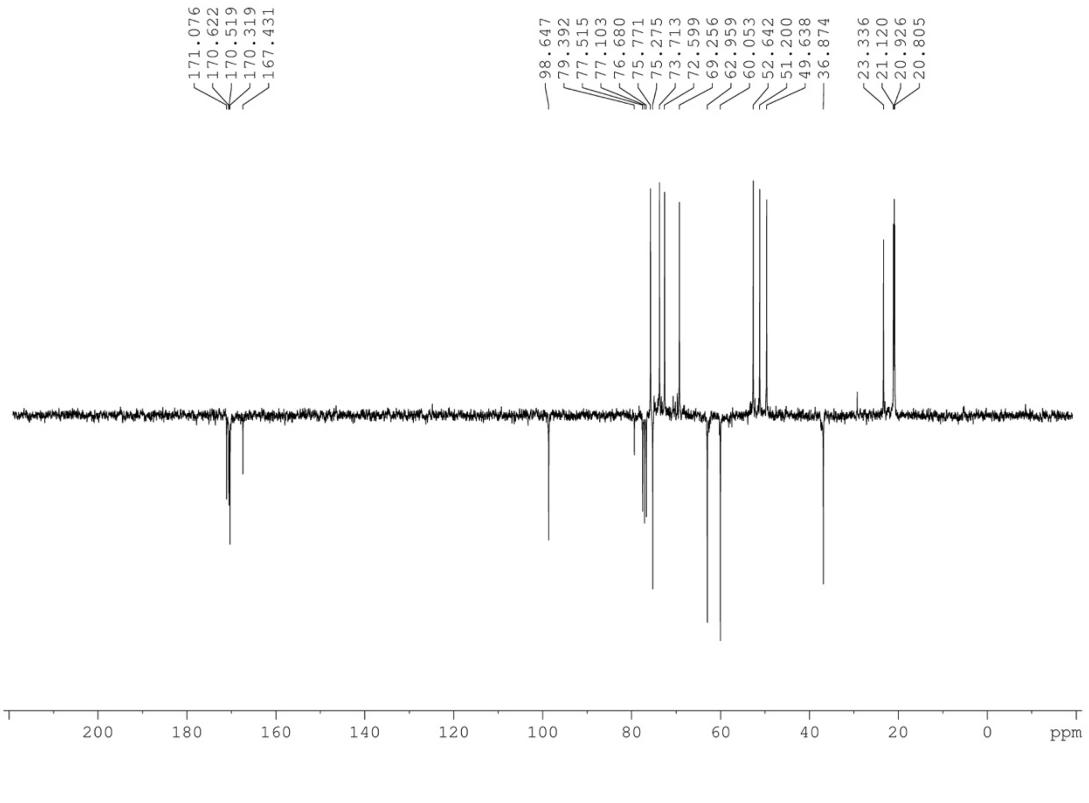

^{1}H NMR ($CDCl_3$): Methyl {methyl 5-acetamido-4,8,9-tri-*O*-acetyl-3,5-dideoxy-7-*O*-(3-phenyl-prop-2-ynyl)-D-*glycero*-β-D-*galacto*-2-nonulopyranosid}onate (**16a**)

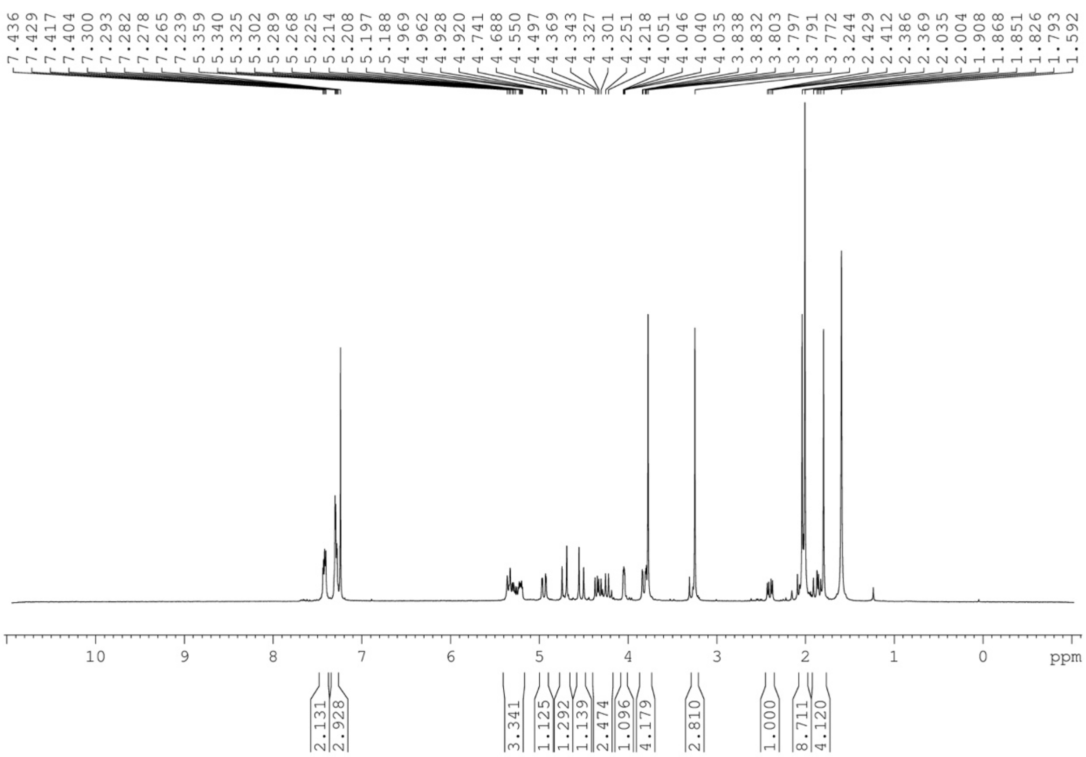

^{13}C NMR ($CDCl_3$): Methyl [methyl 5-acetamido-4,8,9-tri-*O*-acetyl-3,5-dideoxy-7-*O*-(3-phenyl-prop-2-ynyl)-D-*glycero*-β-D-*galacto*-2-nonulopyranosid]onate (**16a**)

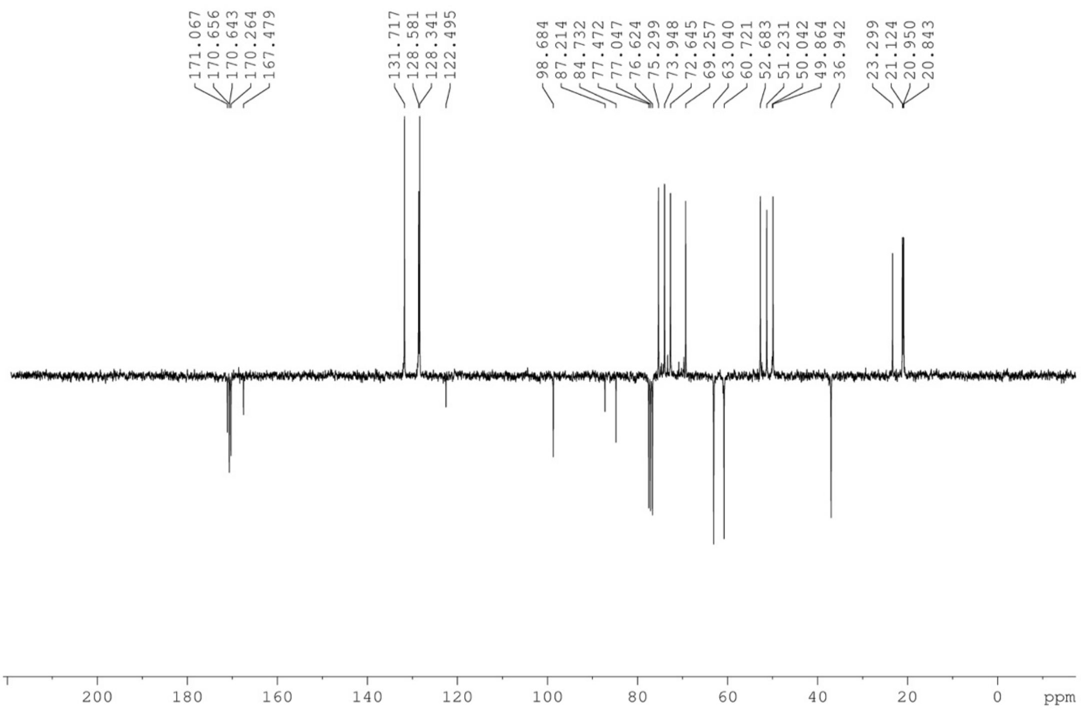

^{1}H NMR ($CDCl_3$): Methyl {methyl 5-acetamido-4,8,9-tri-*O*-acetyl-3,5-dideoxy-7-*O*-[3-(4-methoxyphenyl)-prop-2-ynyl]-D-*glycero*-β-D-*galacto*-2-nonulopyranosid}onate (**16b**)

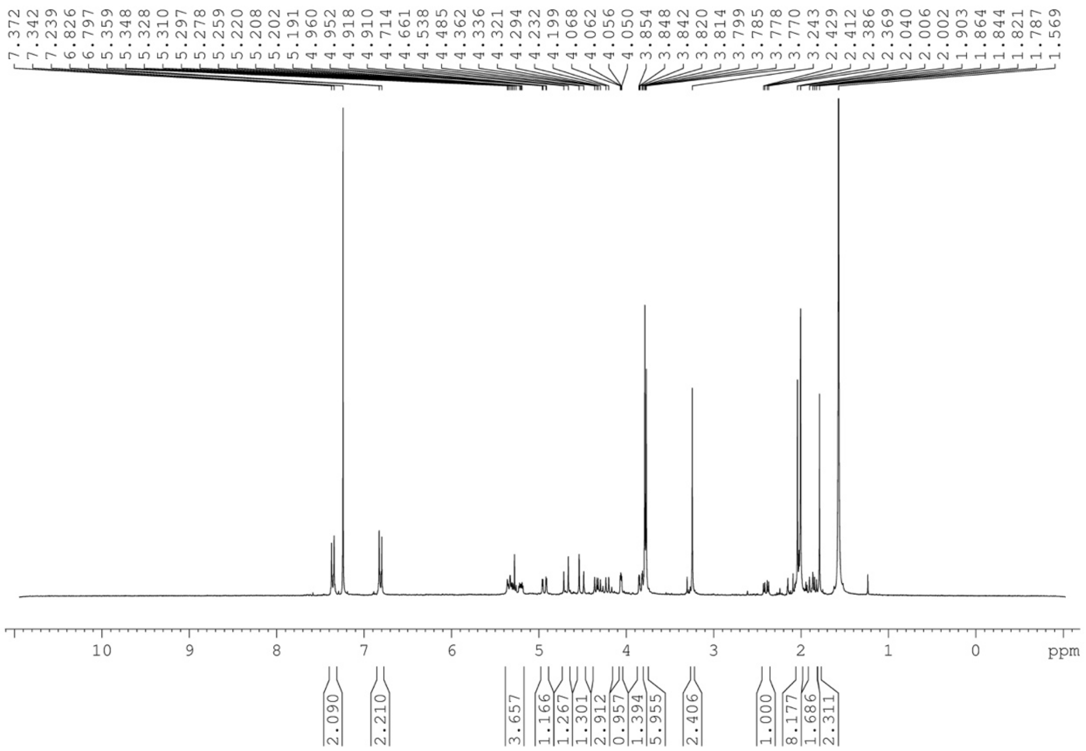

^{13}C NMR ($CDCl_3$): Methyl {methyl 5-acetamido-4,8,9-tri-*O*-acetyl-3,5-dideoxy-7-*O*-[3-(4-methoxyphenyl)-prop-2-ynyl]-D-*glycero*-β-D-*galacto*-2-nonulopyranosid}onate (**16b**)

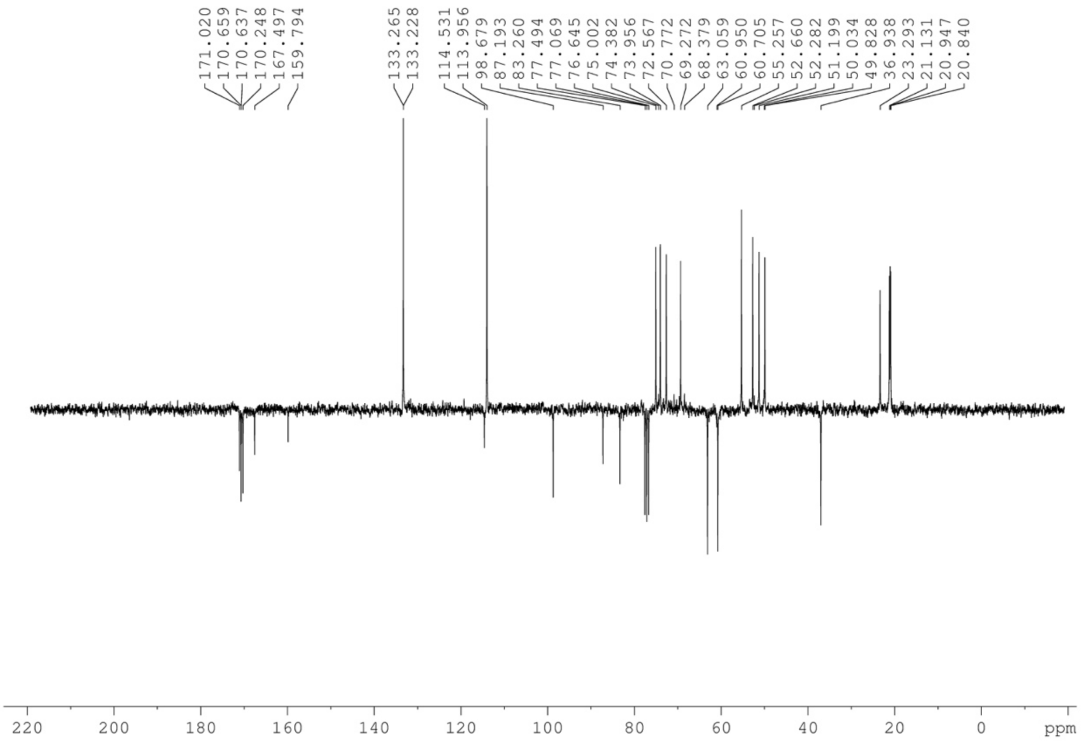

^{1}H NMR ($CDCl_3$): Methyl {methyl 5-acetamido-4,8,9-tri-*O*-acetyl-3,5-dideoxy-7-*O*-[3-(2-thiofuranyl)-prop-2-ynyl]-D-*glycero*-β-D-*galacto*-2-nonulopyranosid}onate (**16c**)

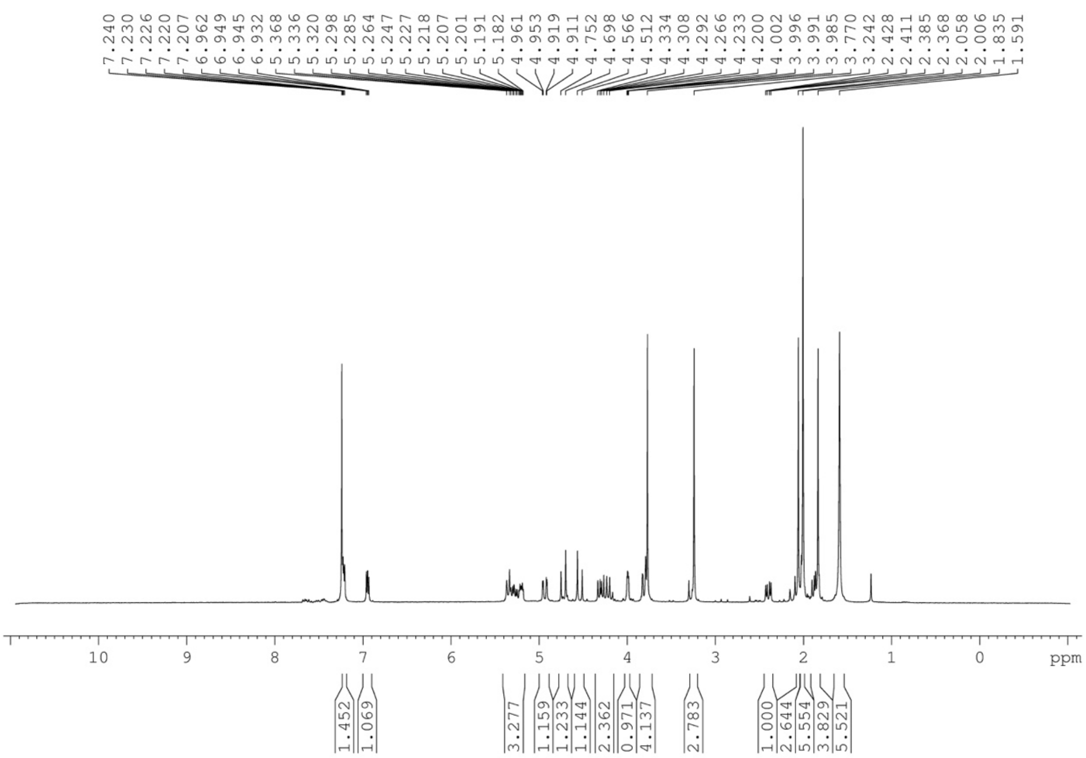

^{13}C NMR ($CDCl_3$): Methyl {methyl 5-acetamido-4,8,9-tri-*O*-acetyl-3,5-dideoxy-7-*O*-[3-(2-thiofuranyl)-prop-2-ynyl]-D-*glycero*-β-D-*galacto*-2-nonulopyranosid}onate (**16c**)

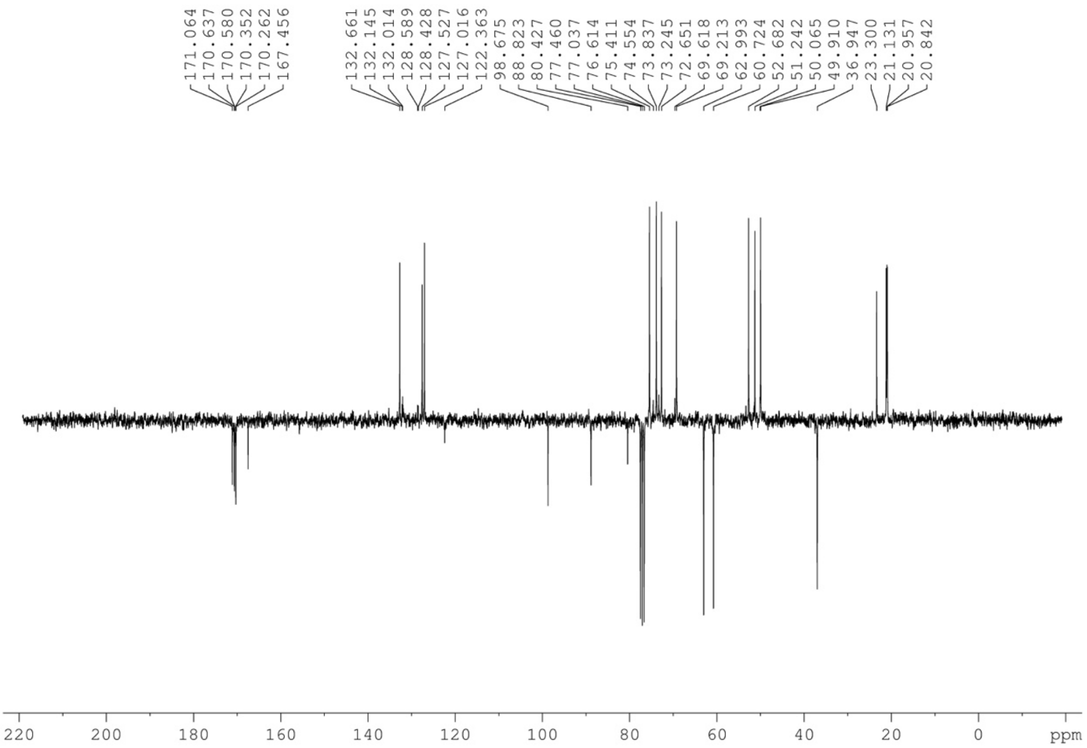

^{1}H NMR (D_2O): Methyl 5-acetamido-3,5-dideoxy-7-*O*-(3-phenyl-prop-2-ynyl)-D-*glycero*-β-D-*galacto*-2-nonulopyranosidonic acid, sodium salt (**17a**)

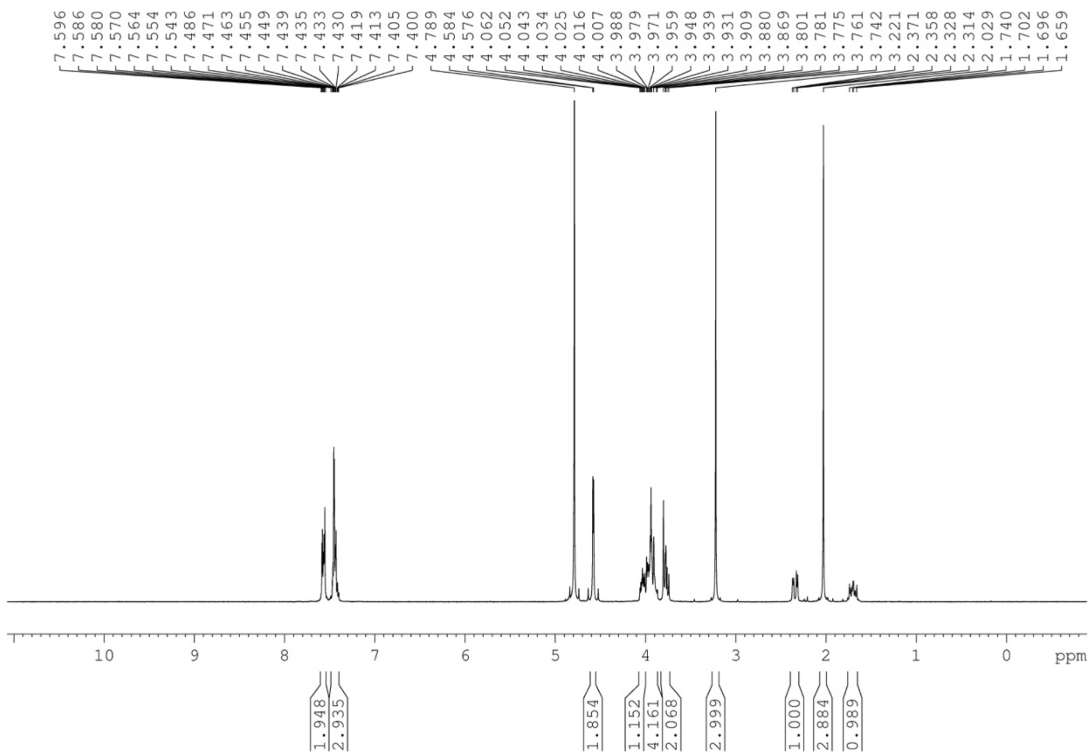

^{13}C NMR (D_2O): Methyl 5-acetamido-3,5-dideoxy-7-*O*-(3-phenyl-prop-2-ynyl)-D-*glycero*-β-D-*galacto*-2-nonulopyranosidonic acid, sodium salt (**17a**)

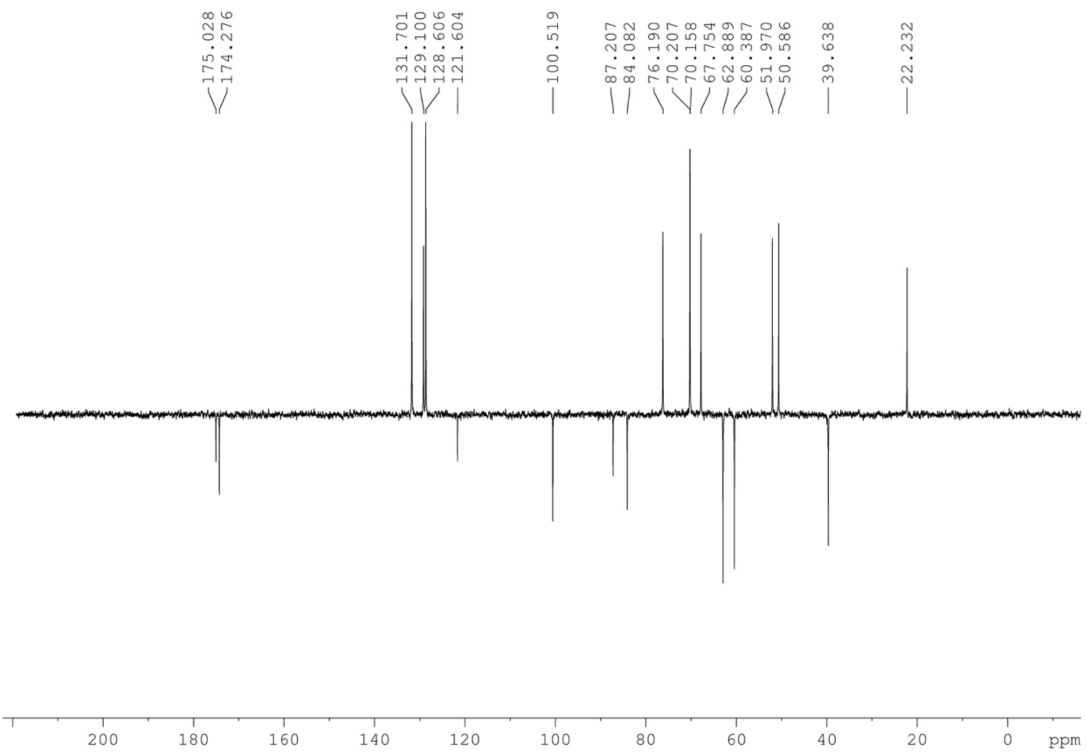

^{1}H NMR (D_2O): Methyl 5-acetamido-3,5-dideoxy-7-*O*-[3-(4-methoxyphenyl)-prop-2-ynyl]-D-*glycero*-β-D-*galacto*-2-nonulopyranosidonic acid, sodium salt (**17b**)

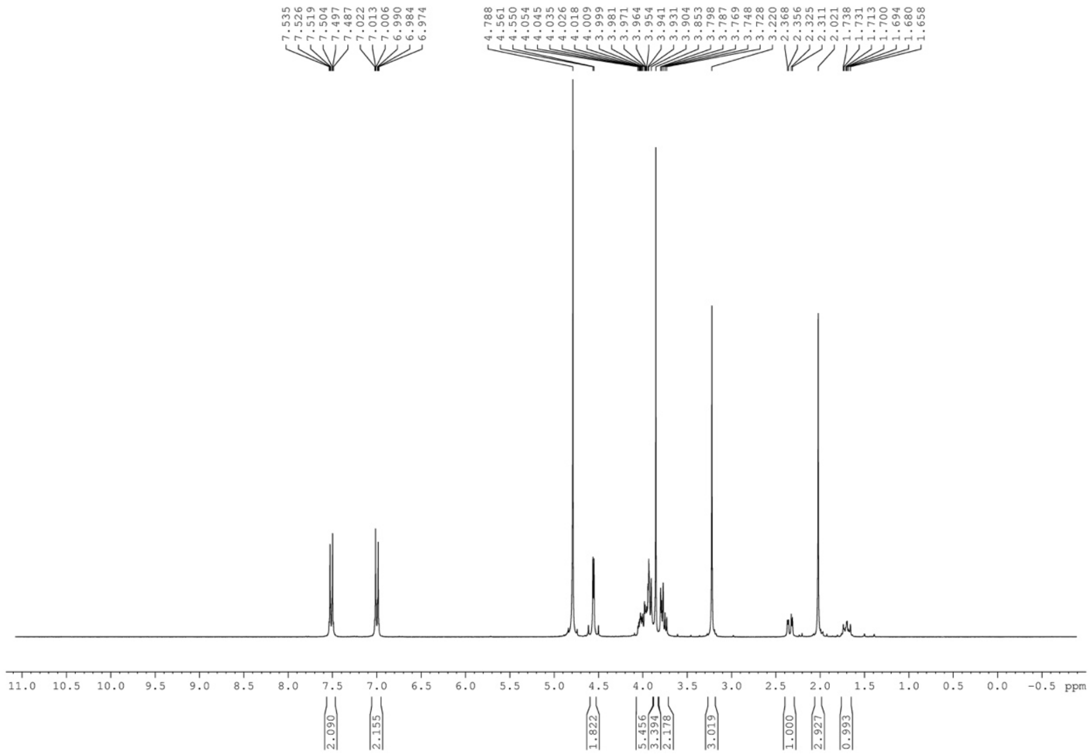

^{13}C NMR (D_2O): Methyl 5-acetamido-3,5-dideoxy-7-*O*-[3-(4-methoxyphenyl)-prop-2-ynyl]-D-*glycero*-β-D-*galacto*-2-nonulopyranosidonic acid, sodium salt (**17b**)

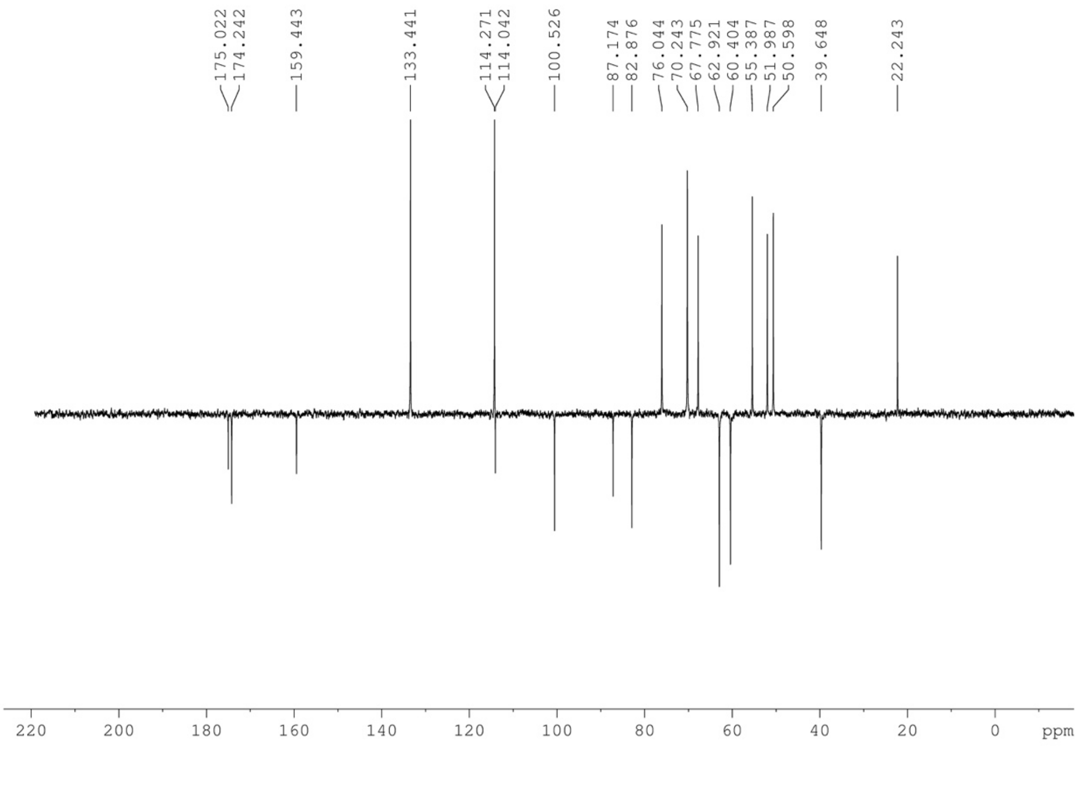

^{1}H NMR (D_2O): Methyl 5-acetamido-3,5-dideoxy-7-*O*-[3-(2-thiofuranyl)-prop-2-ynyl]-D-*glycero*-β-D-*galacto*-2-nonulopyranosidonic acid, sodium salt (**17c**)

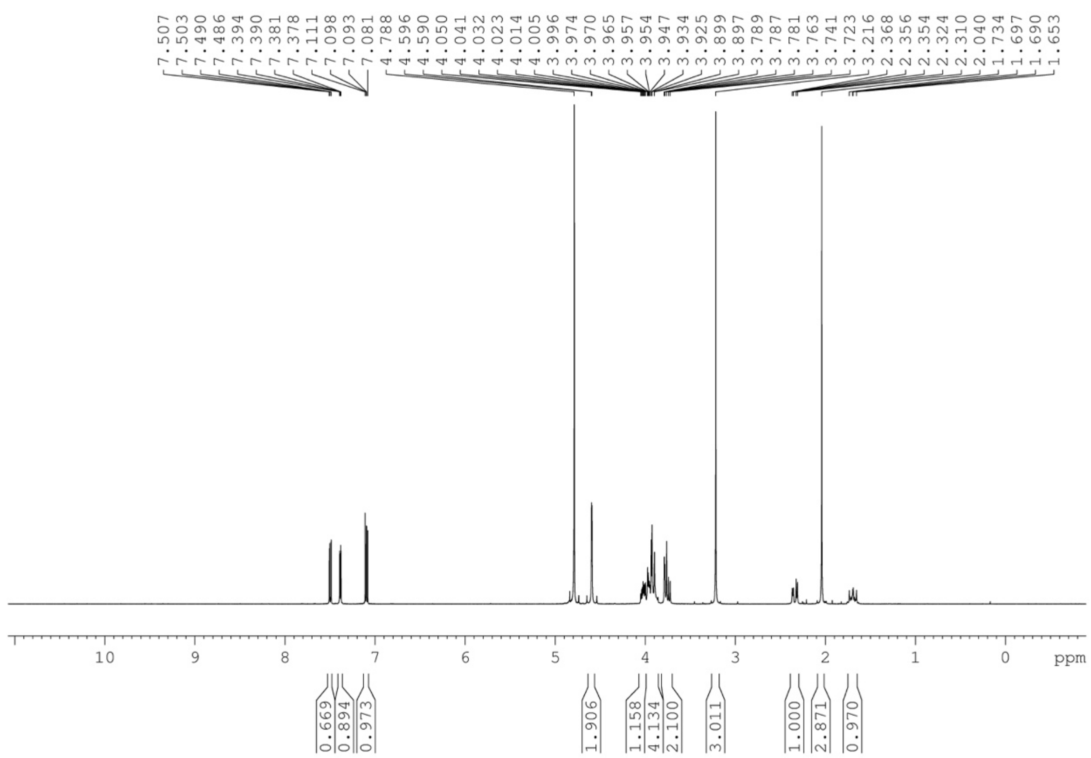

^{13}C NMR (D_2O): Methyl 5-acetamido-3,5-dideoxy-7-*O*-[3-(2-thiofuranyl)-prop-2-ynyl]-D-*glycero*-β-D-*galacto*-2-nonulopyranosidonic acid, sodium salt (**17c**)

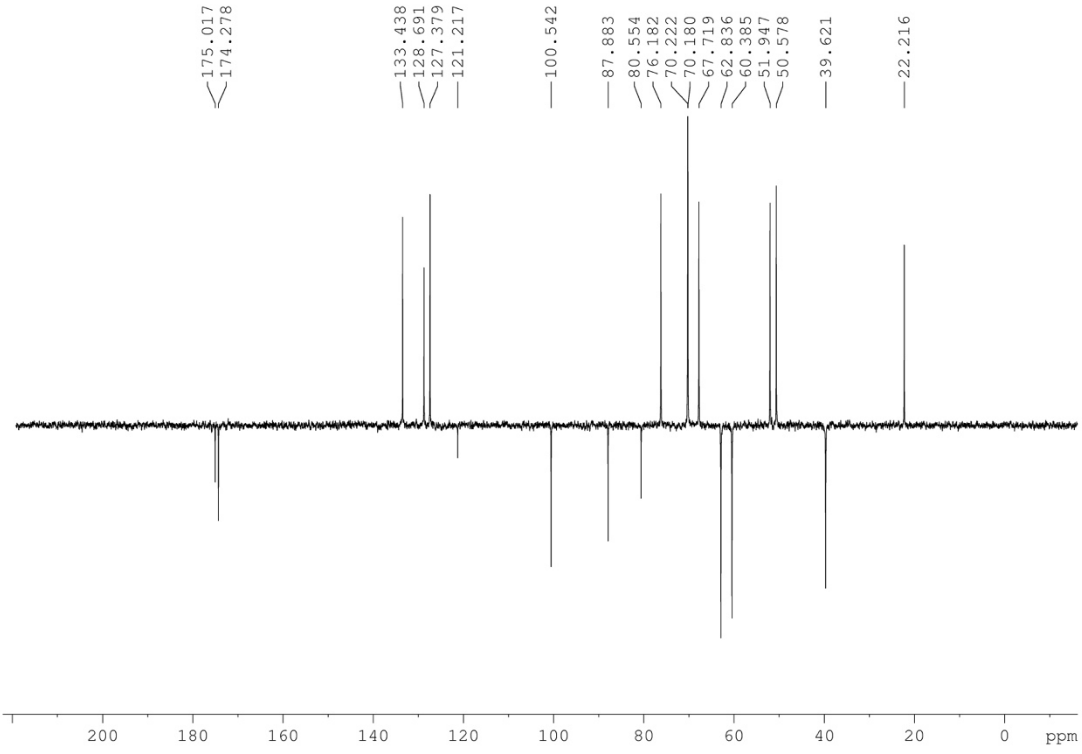

^{1}H NMR (D_2O): Methyl 5-amino-3,5-dideoxy-D-*glycero*-β-D-*galacto*-non-2-ulopyranosidonic acid, ammonium salt (**18**)

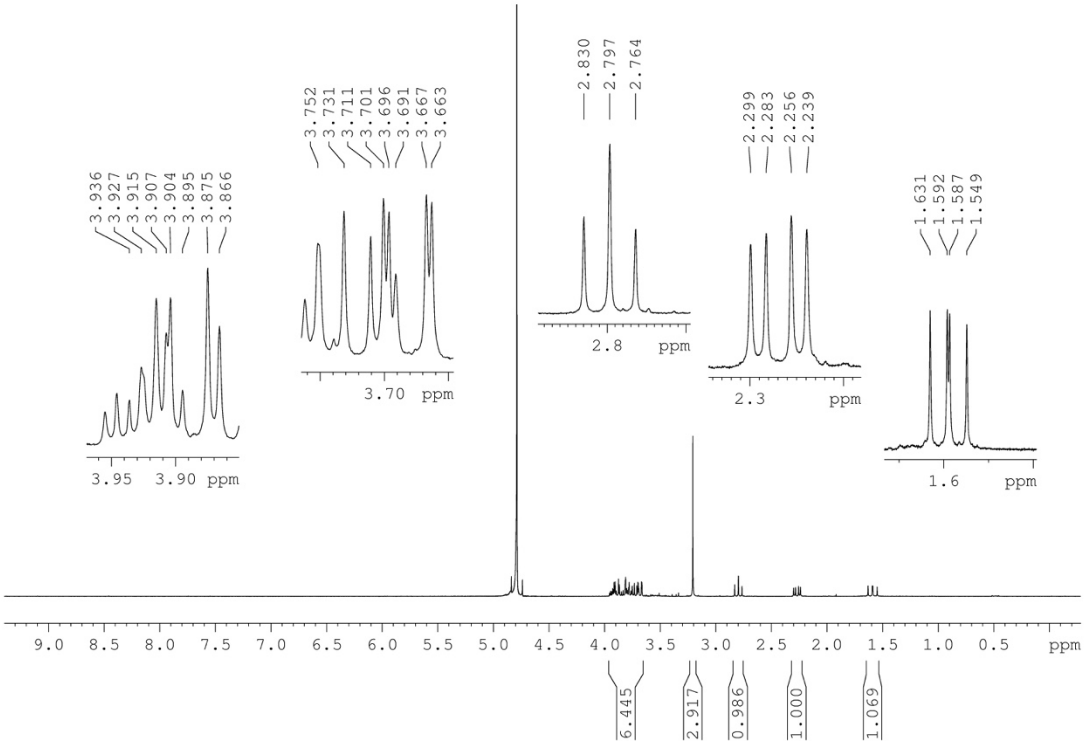

^{13}C NMR (D_2O): Methyl 5-amino-3,5-dideoxy-D-*glycero*-β-D-*galacto*-non-2-ulopyranosidonic acid, ammonium salt (**18**)

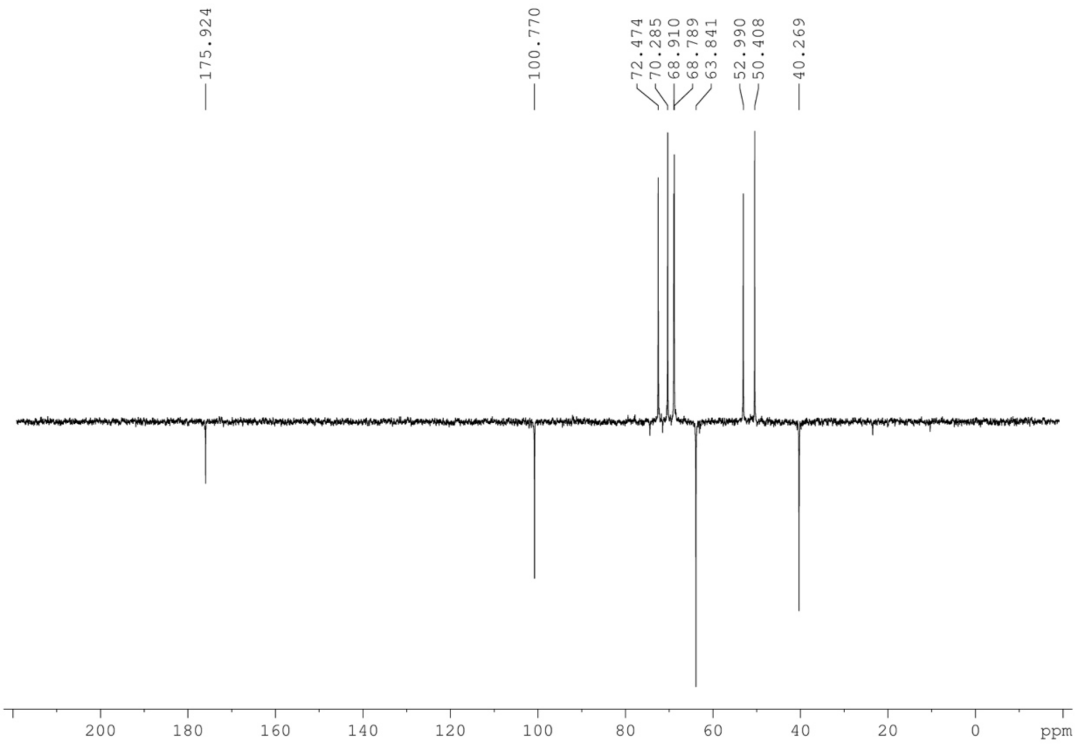

^{1}H NMR (CD_3OD): Methyl (methyl 5-amino-3,5-dideoxy-D-*glycero*-β-D-*galacto*-non-2-ulopyranosid)onate (**19**)

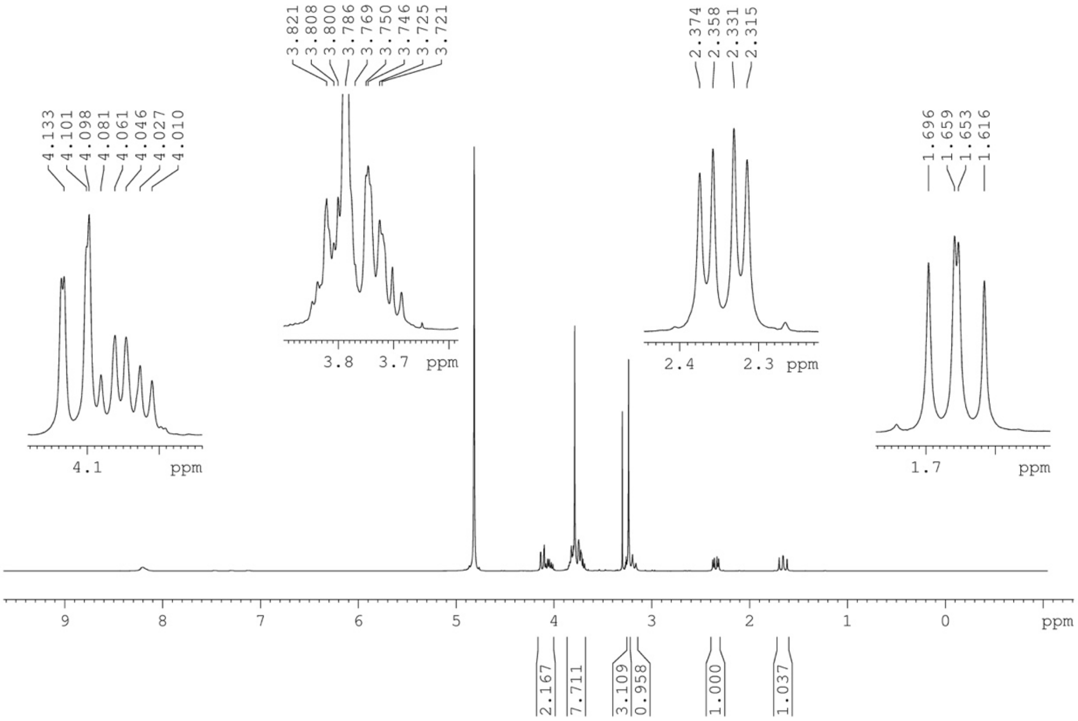

^{13}C NMR (CD_3OD): Methyl (methyl 5-amino-3,5-dideoxy-D-*glycero*-β-D-*galacto*-non-2-ulopyranosid)onate (**19**)

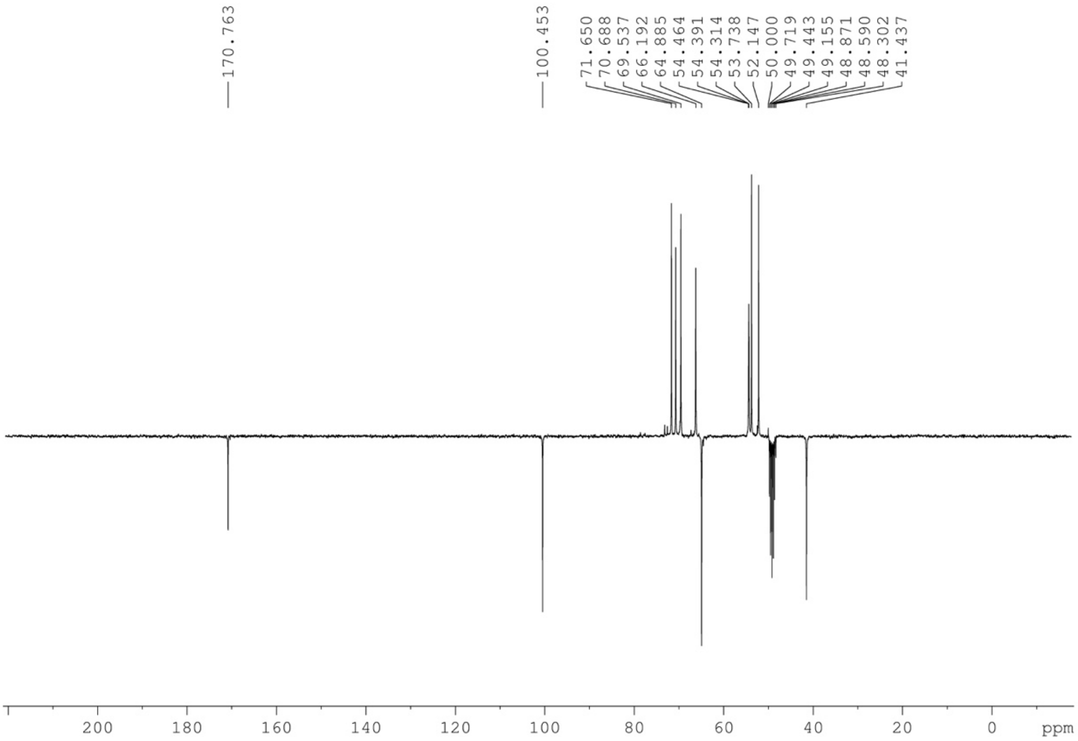

^{1}H NMR (CD_3OD): Methyl (methyl 5-butanamido-3,5-dideoxy-D-*glycero*-β-D-*galacto*-non-2-ulopyranosid)onate (**20a**)

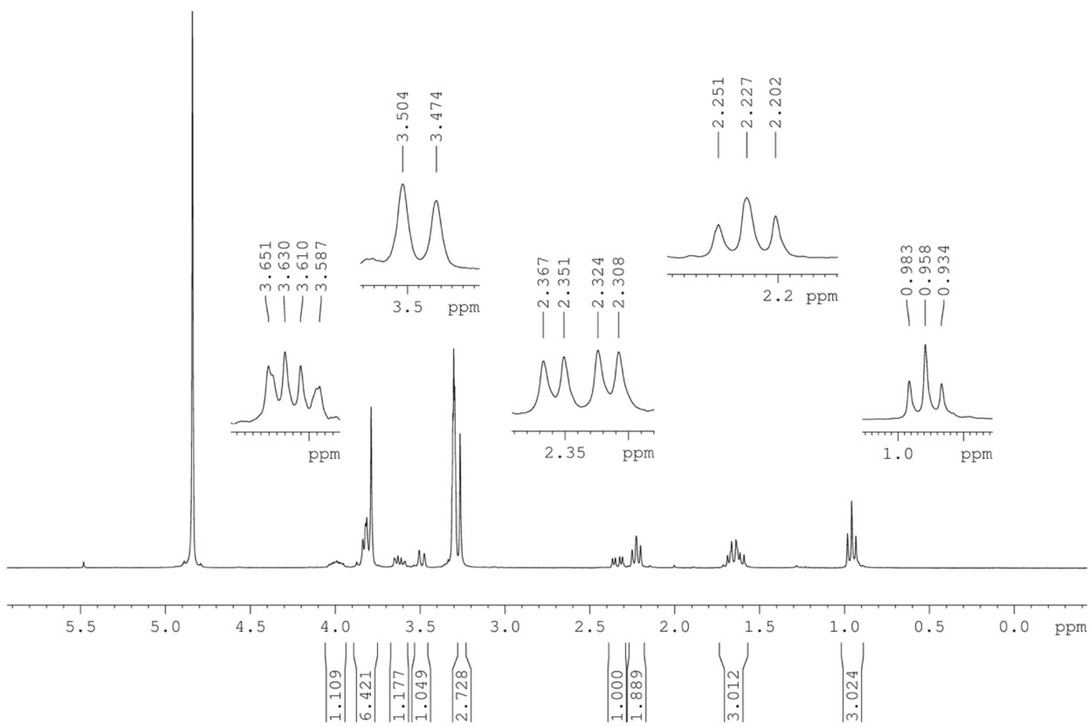

^{13}C NMR (CD_3OD): Methyl (methyl 5-butanamido-3,5-dideoxy-D-*glycero*-β-D-*galacto*-non-2-ulopyranosid)onate (**20a**)

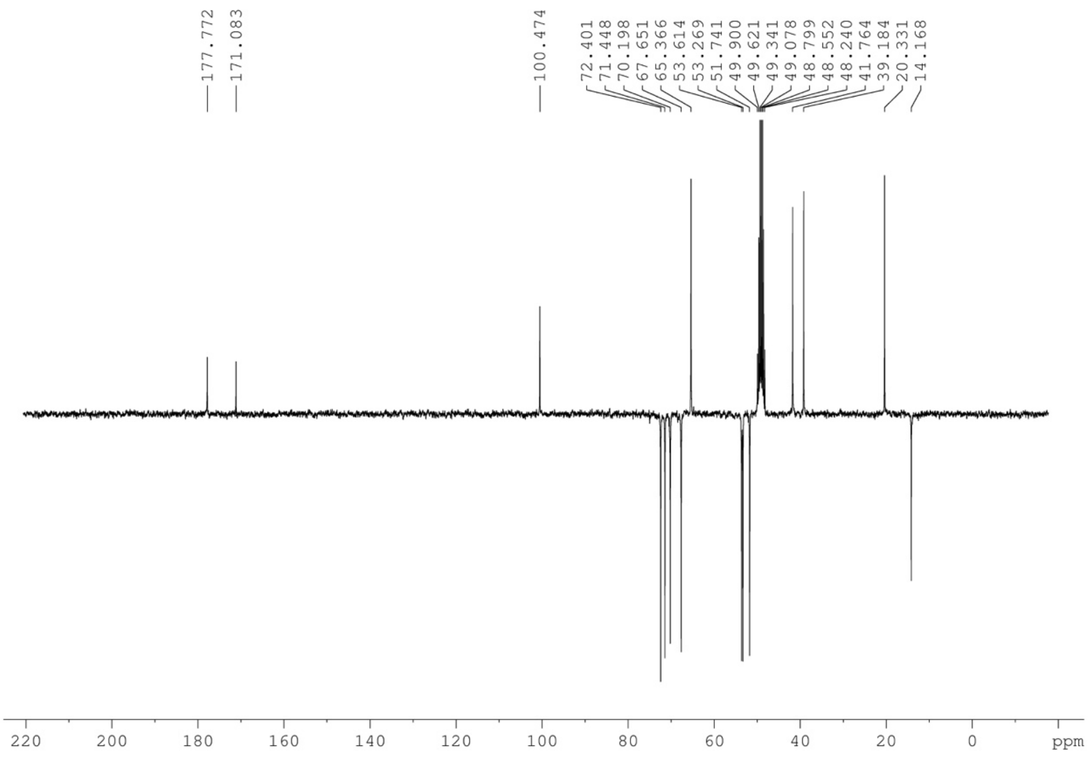

^{1}H NMR (CD_3OD): Methyl (methyl 5-cyclopentylcarboxamido-3,5-dideoxy-D-*glycero*-β-D-*galacto*-non-2-ulopyranosid)onate (**20b**)

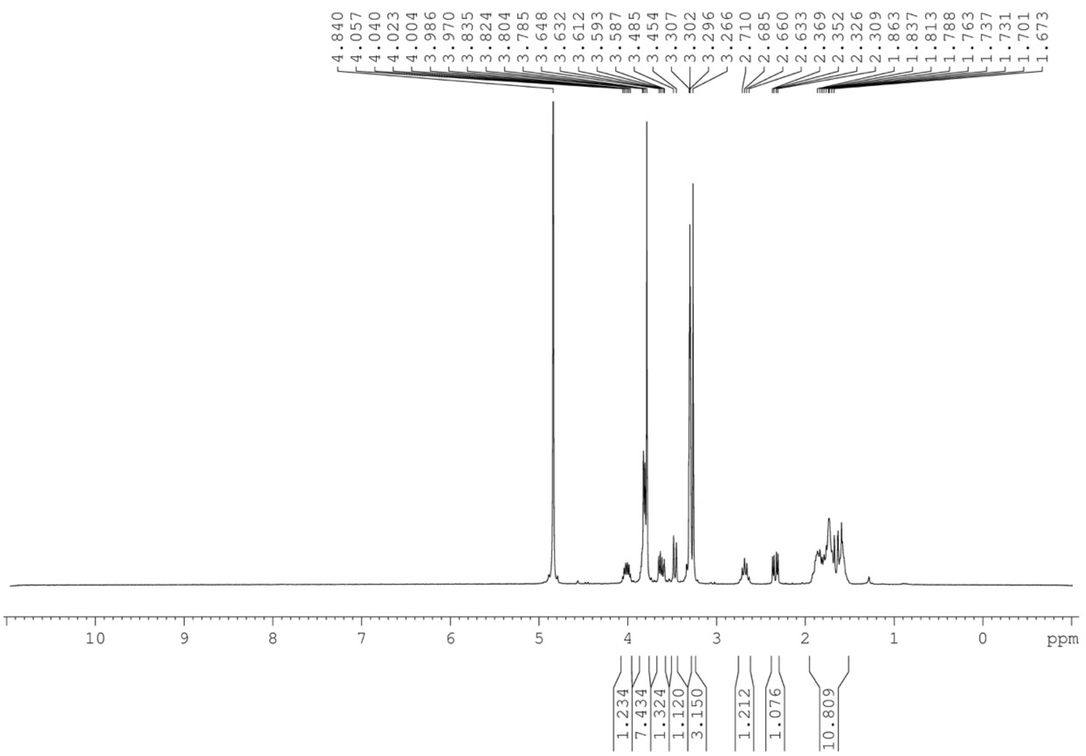

^{13}C NMR (CD_3OD): Methyl (methyl 5-cyclopentylcarboxamido-3,5-dideoxy-D-*glycero*-β-D-*galacto*-non-2-ulopyranosid)onate (**20b**)

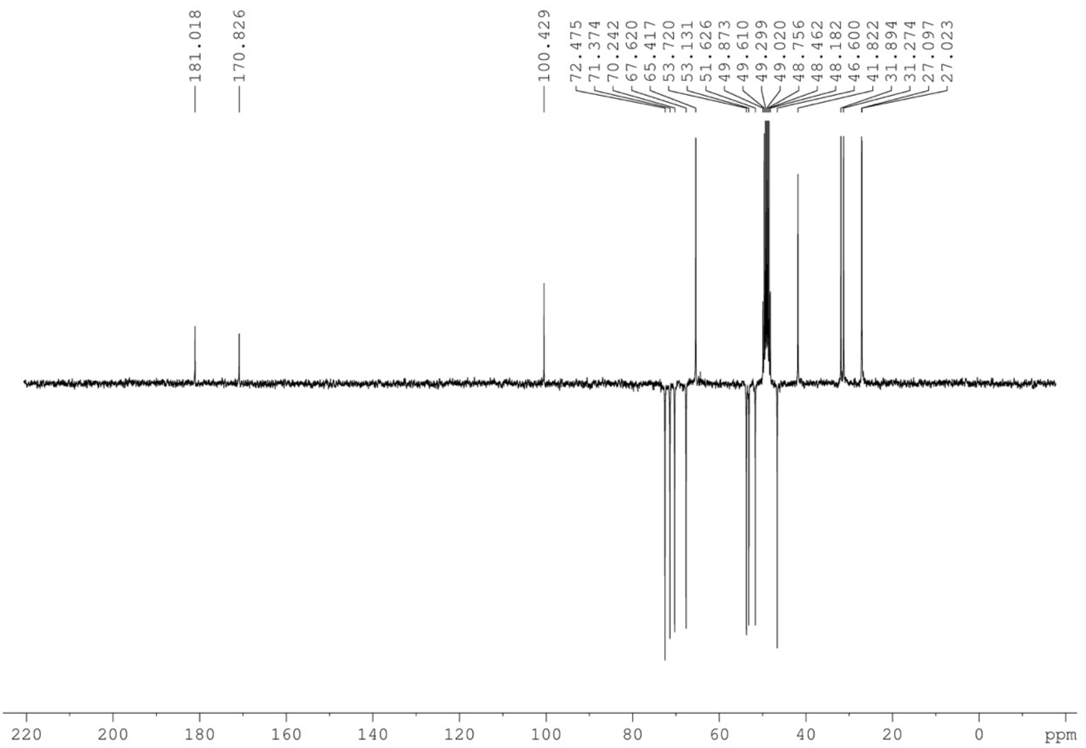

^{1}H NMR (CD_3OD): Methyl (methyl 5-cyclohexylcarboxamido-3,5-dideoxy-D-*glycero*-β-D-*galacto*-non-2-ulopyranosid)onate (**20c**)

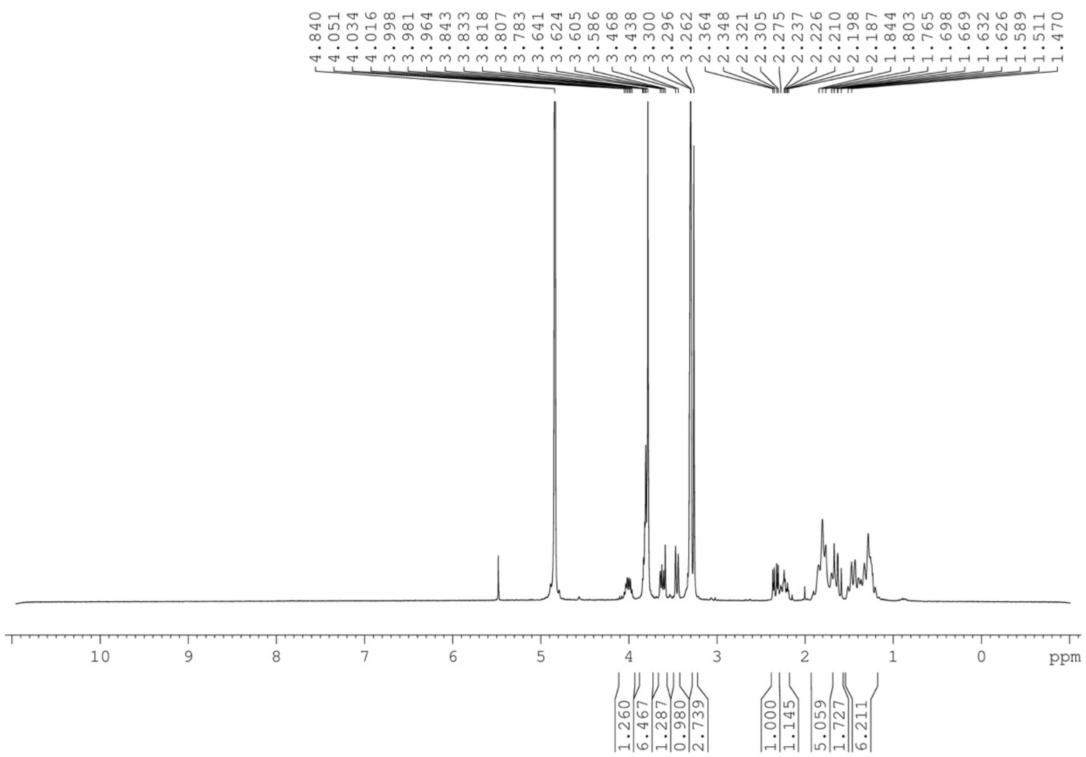

^{13}C NMR (CD_3OD): Methyl (methyl 5-cyclohexylcarboxamido-3,5-dideoxy-D-*glycero*-β-D-*galacto*-non-2-ulopyranosid)onate (**20c**)

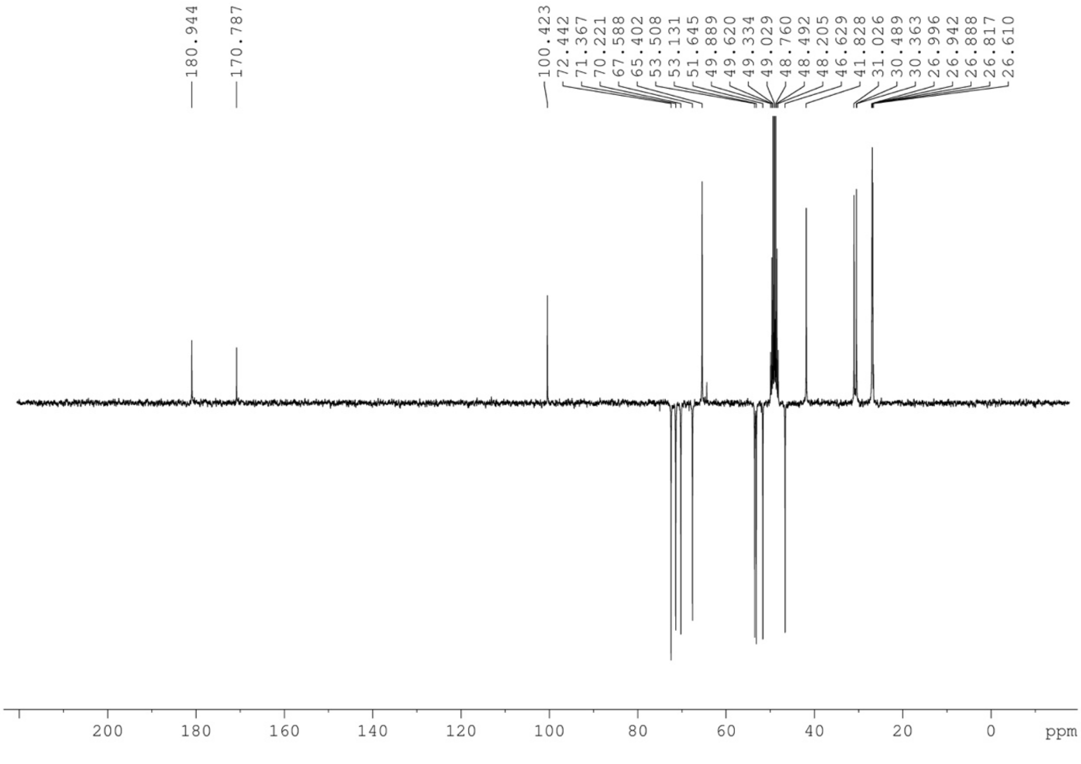

^{1}H NMR (CD_3OD): Methyl (methyl 5-acetoxyacetamido-3,5-dideoxy-D-*glycero*-β-D-*galacto*-non-2-ulopyranosid)onate (**20d**)

[Note: An inseparable impurity of acetoxyglycolic acid was present in the isolated product.]

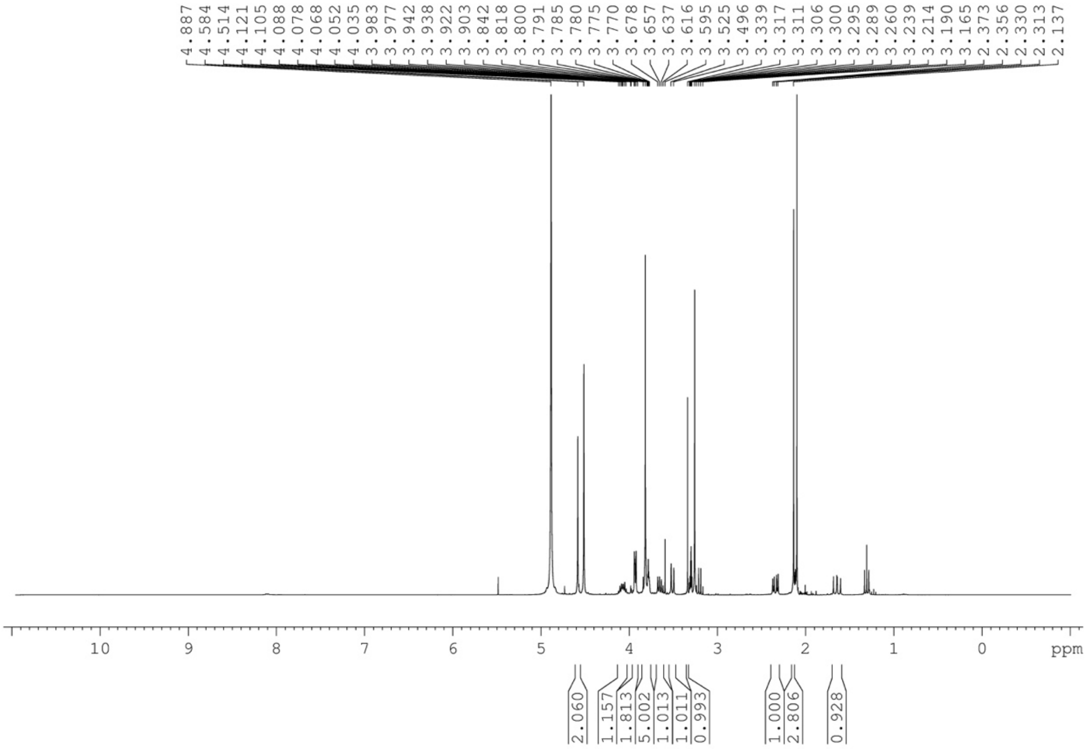

^{13}C NMR (CD_3OD): Methyl (methyl 5-acetoxyacetamido-3,5-dideoxy-D-*glycero*-β-D-*galacto*-non-2-ulopyranosid)onate (**20d**)

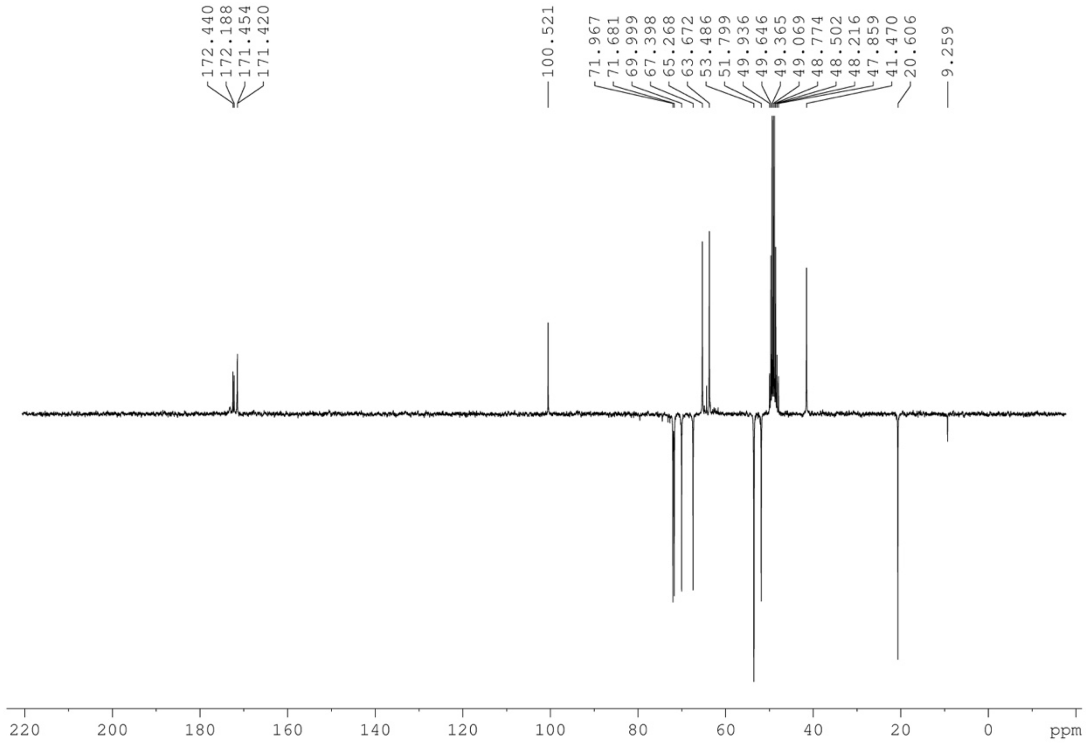

^{1}H NMR (CD_3OD): Methyl [methyl 3,5-dideoxy-5-(5-methoxycarbonyl-pentanamido)-D-*glycero*-β-D-*galacto*-non-2-ulopyranosid]onate (**20e**)

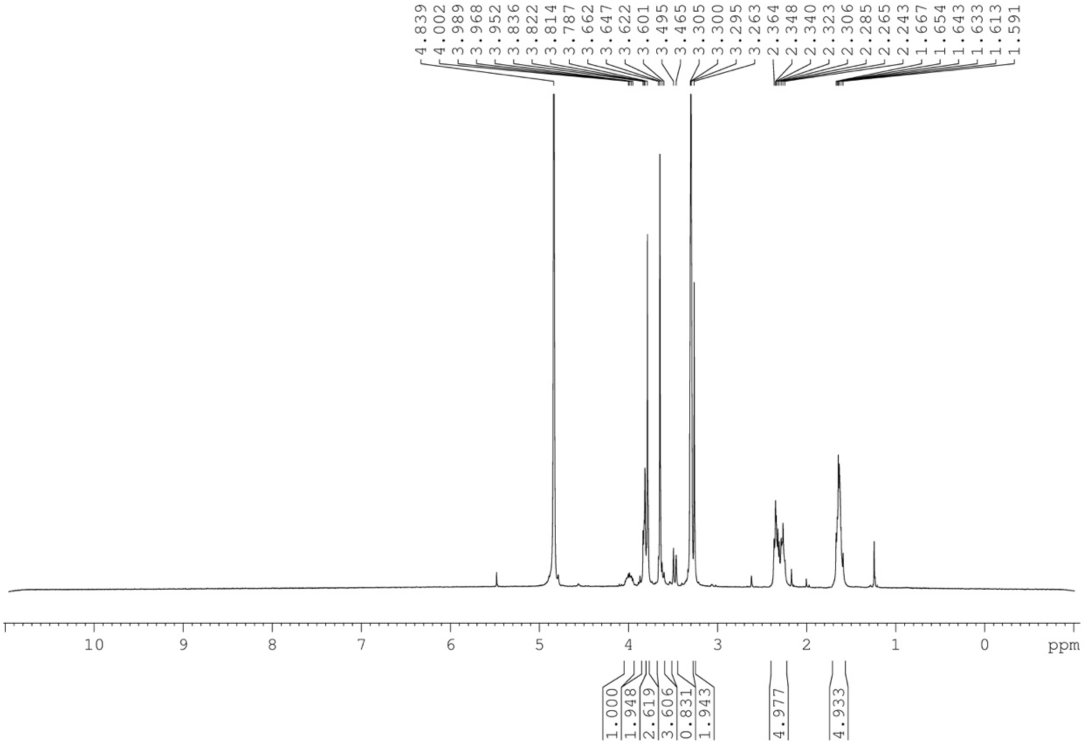

^{13}C NMR (CD_3OD): Methyl [methyl 3,5-dideoxy-5-(5-methoxycarbonyl-pentanamido)-D-*glycero*-β-D-*galacto*-non-2-ulopyranosid]onate (**20e**)

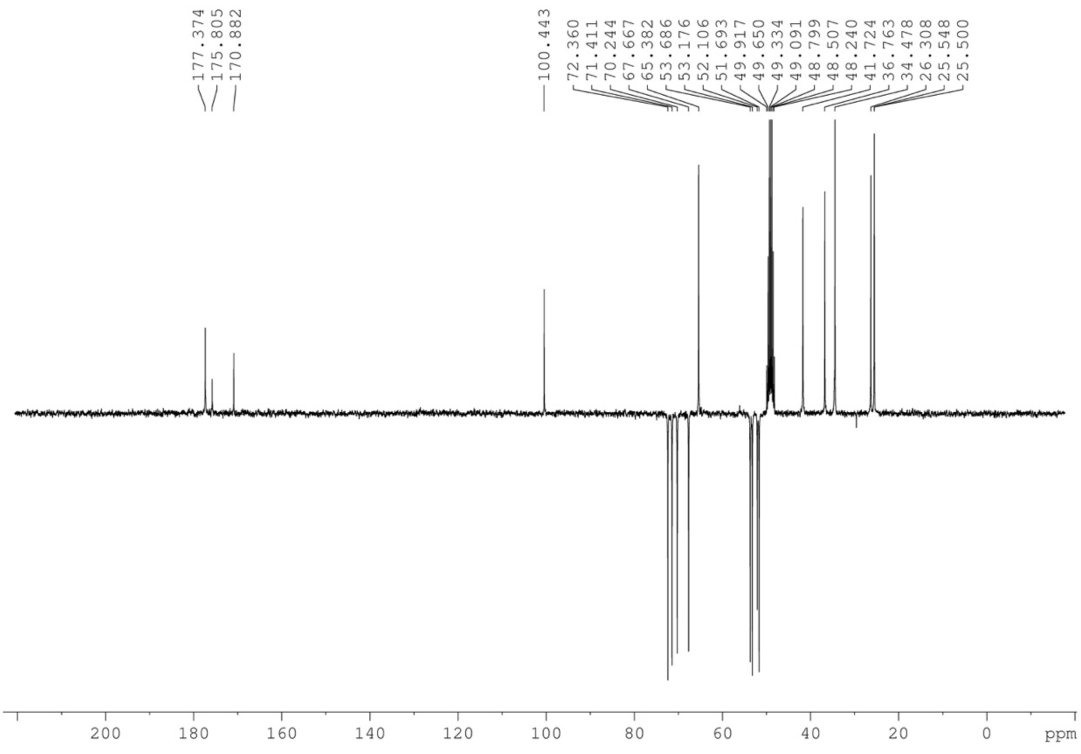

^{1}H NMR (CD_3OD): Methyl [methyl 3,5-dideoxy-5-(3-phenyl-propanamido)-D-*glycero*-β-D-*galacto*-non-2-ulopyranosid]onate (**20f**)

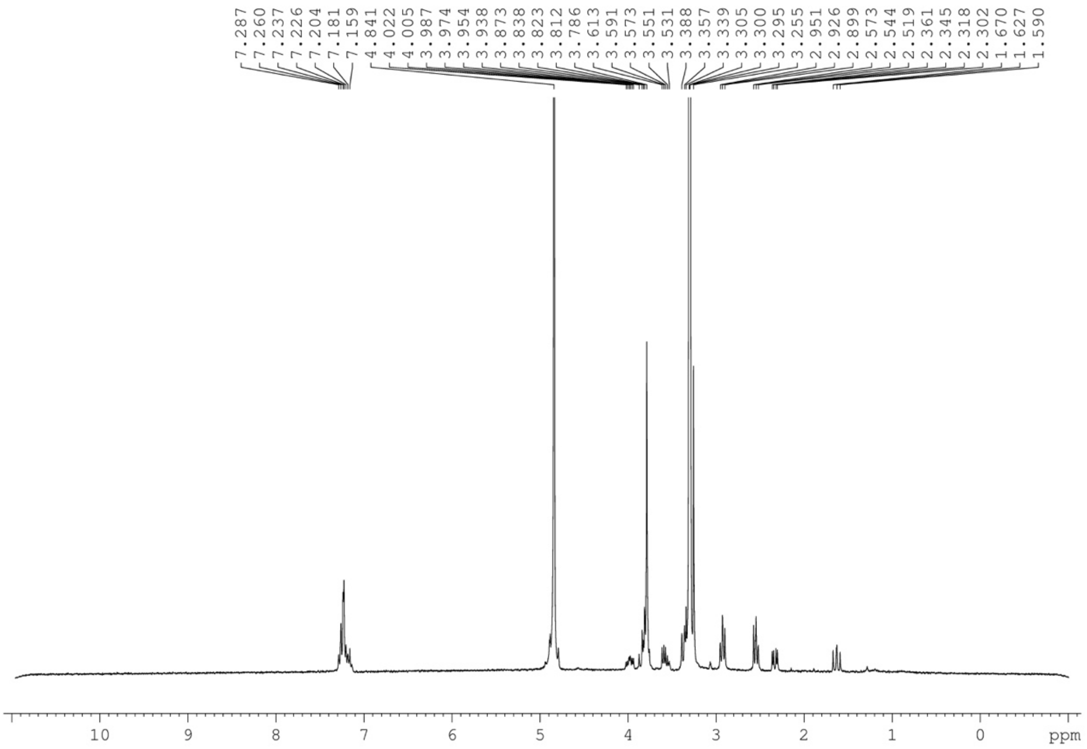

^{13}C NMR (CD_3OD): Methyl [methyl 3,5-dideoxy-5-(3-phenyl-propanamido)-D-*glycero*-β-D-*galacto*-non-2-ulopyranosid]onate (**20f**)

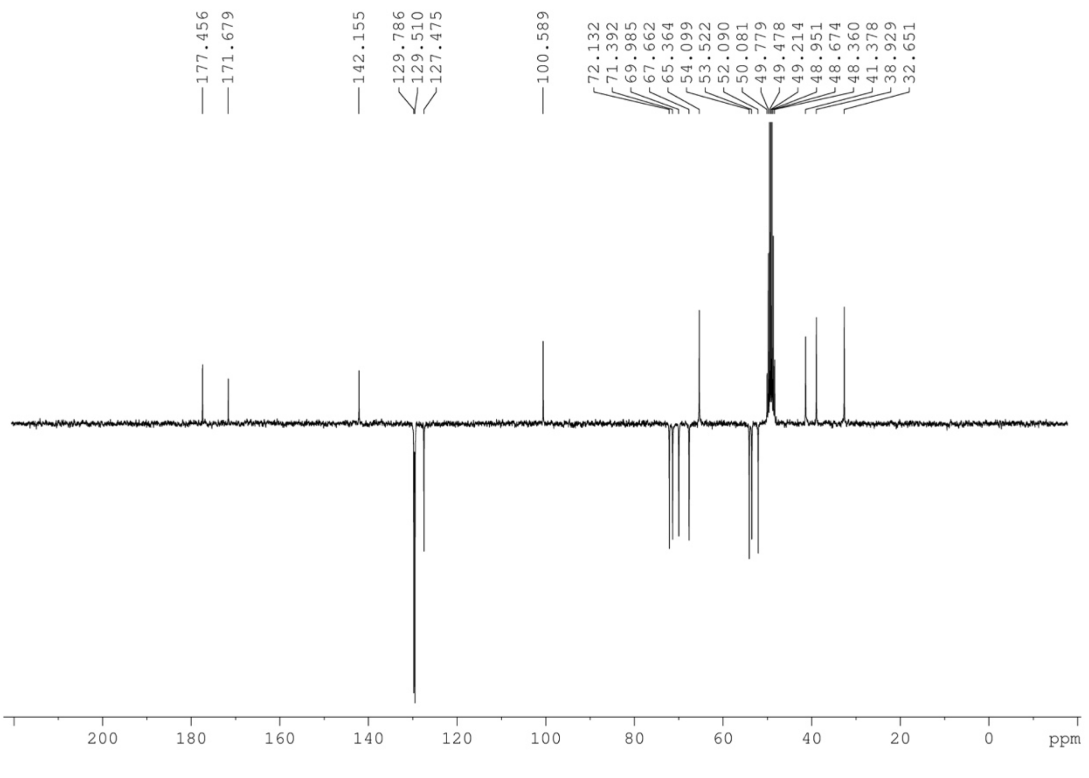

^{1}H NMR (CD_3OD): Methyl [methyl 3,5-dideoxy-5-(4-phenoxy-butanamido)-D-*glycero*-β-D-*galacto*-non-2-ulopyranosid]onate (**20g**)

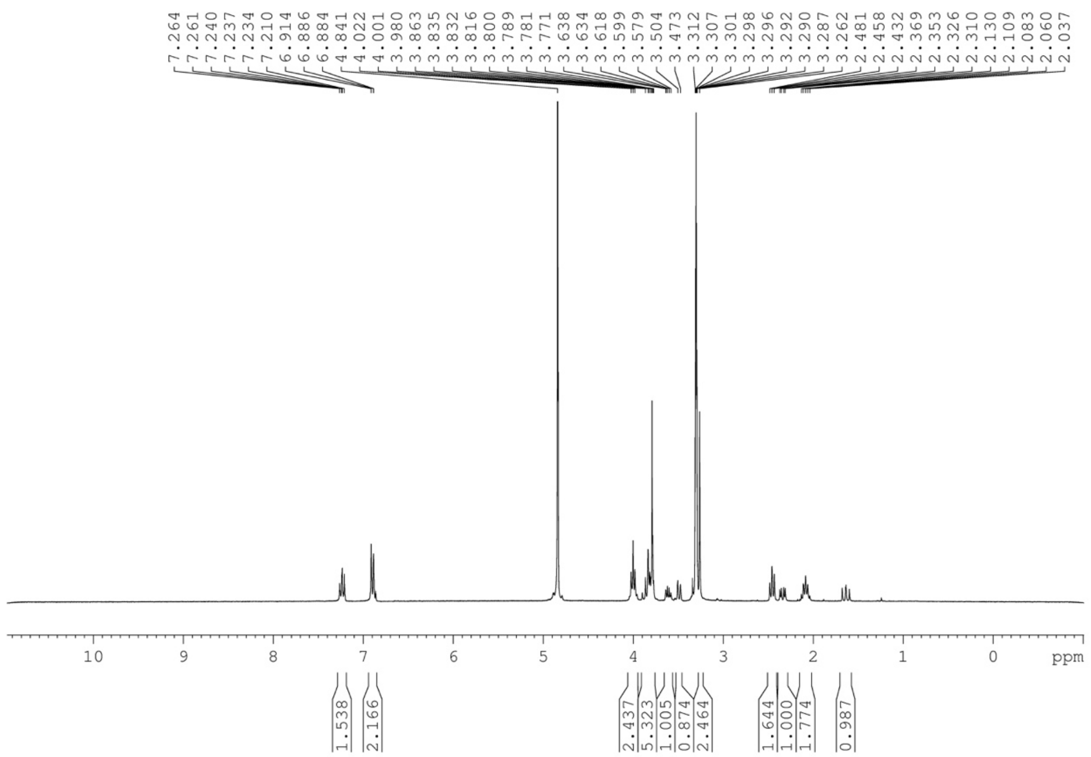

^{13}C NMR (CD_3OD): Methyl [methyl 3,5-dideoxy-5-(4-phenoxy-butanamido)-D-*glycero*-β-D-*galacto*-non-2-ulopyranosid]onate (**20g**)

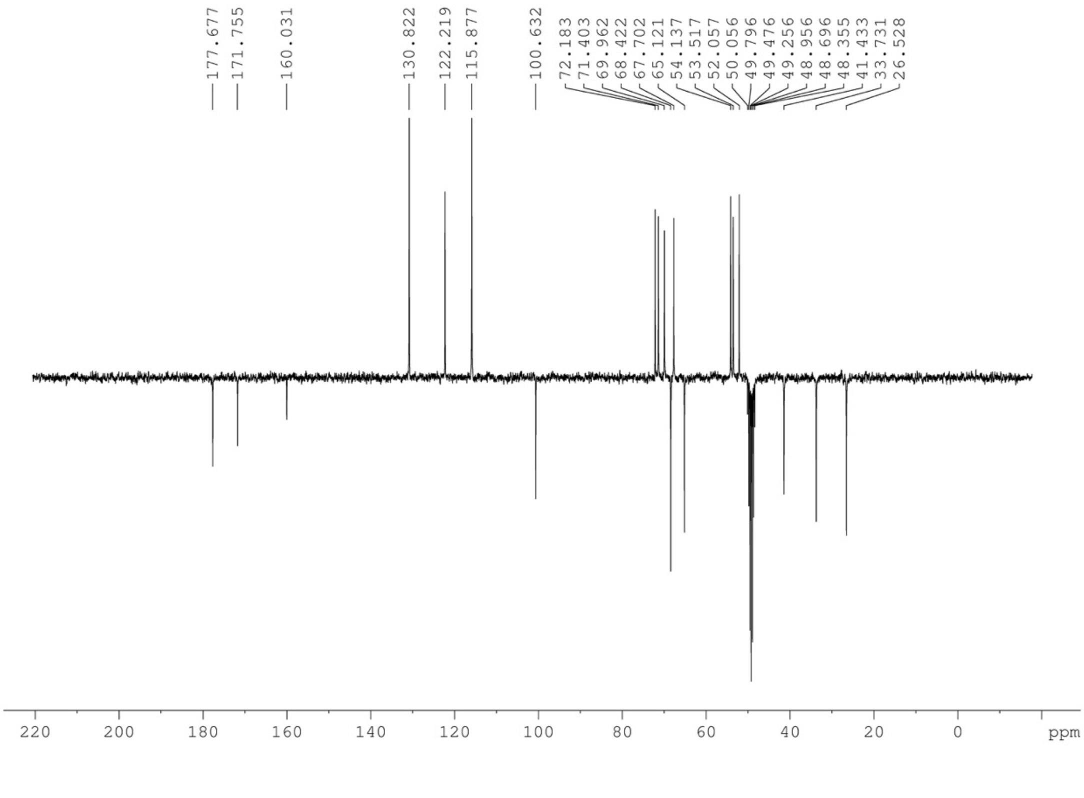

^{1}H NMR (CD_3OD): Methyl [methyl 3,5-dideoxy-5-(4-phenyl-benzamido)-D-*glycero*-β-D-*galacto*-non-2-ulopyranosid]onate (**20h**)

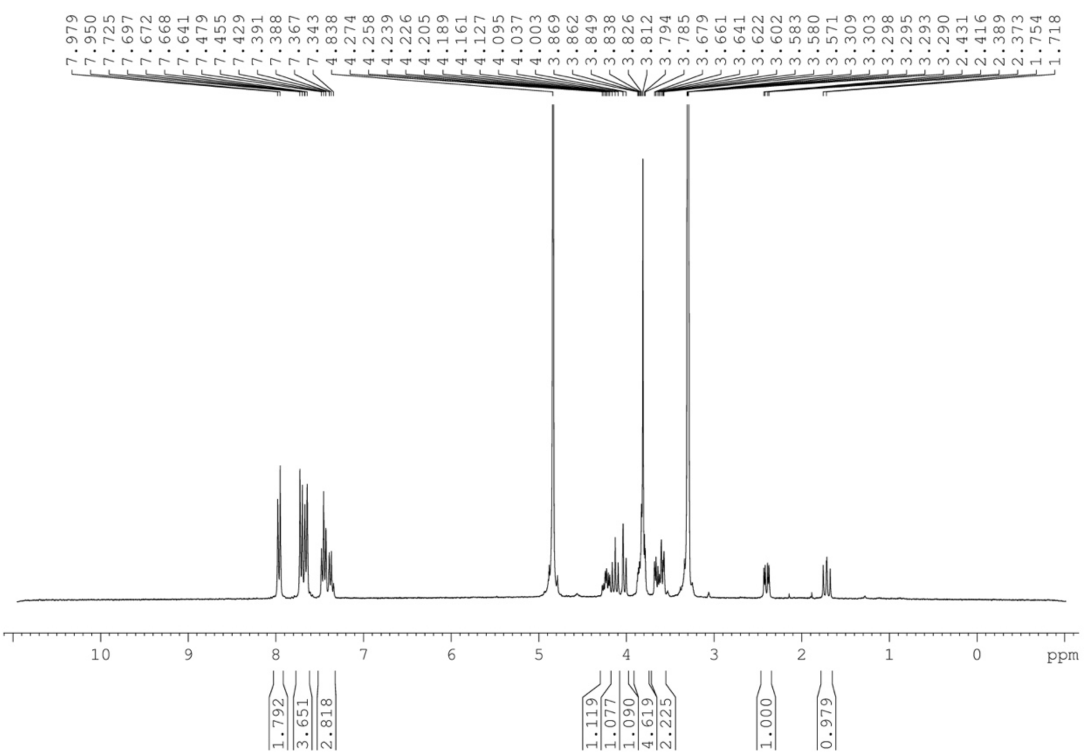

^{13}C NMR (CD_3OD): Methyl [methyl 3,5-dideoxy-5-(4-phenyl-benzamido)-D-*glycero*-β-D-*galacto*-non-2-ulopyranosid]onate (**20h**)

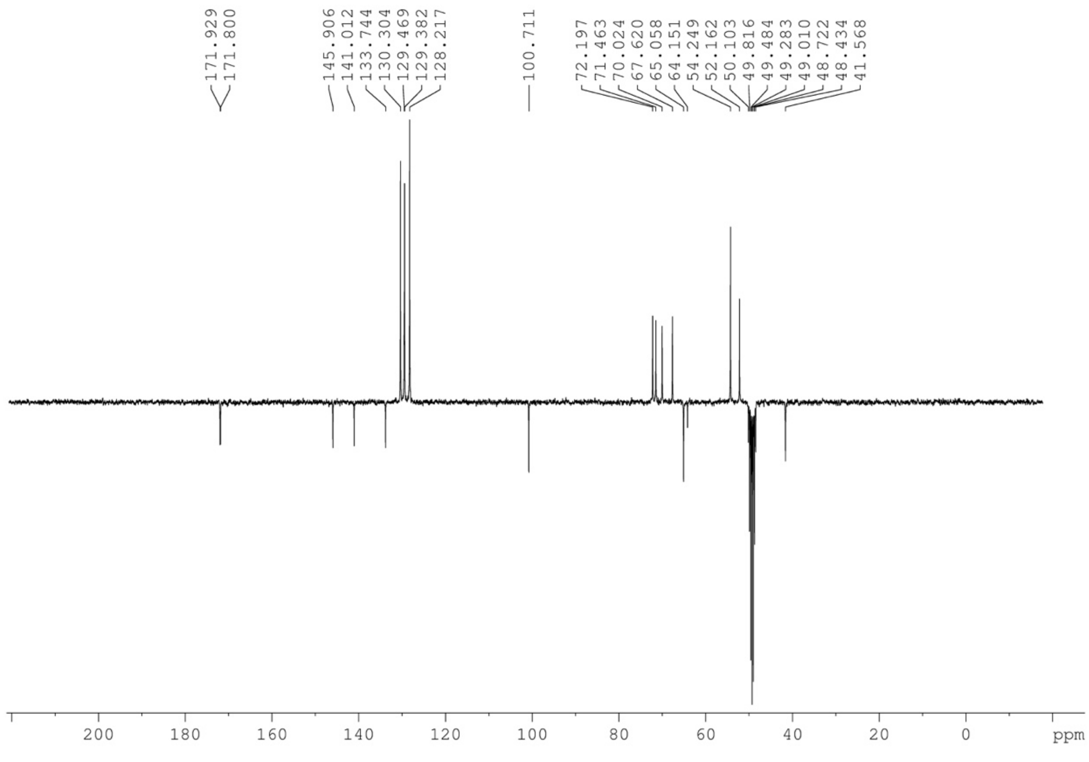

^{1}H NMR (CD_3OD): Methyl [methyl 3,5-dideoxy-5-(1-naphthylcarboxamido)-D-*glycero*-β-D-*galacto*-non-2-ulopyranosid]onate (**20i**)

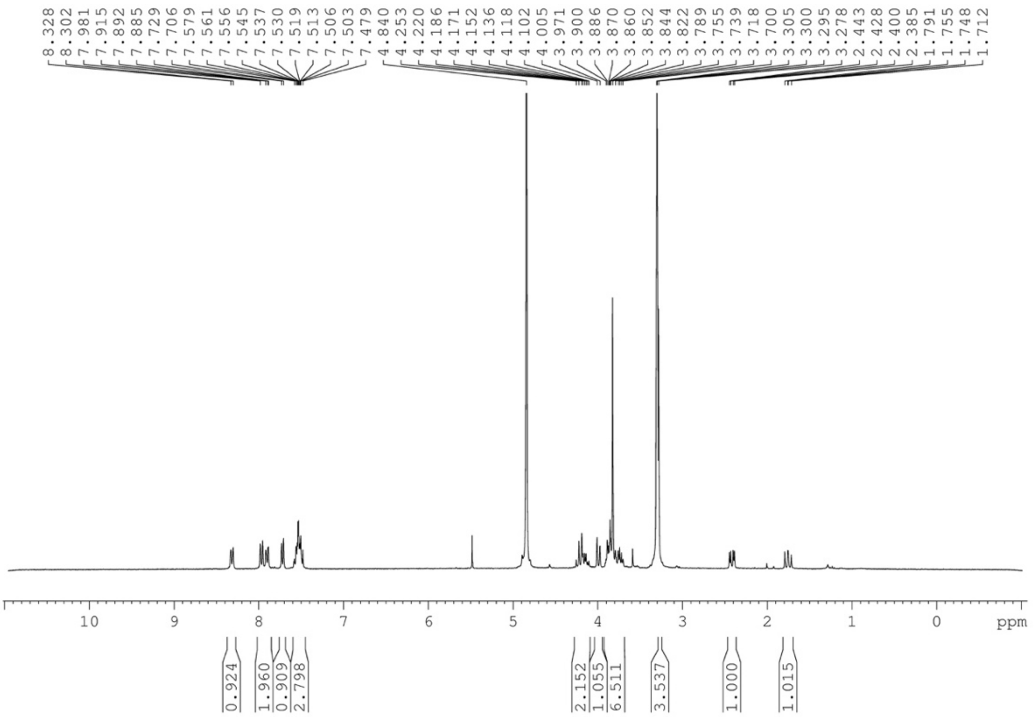

^{13}C NMR (CD_3OD): Methyl [methyl 3,5-dideoxy-5-(1-naphthylcarboxamido)-D-*glycero*-β-D-*galacto*-non-2-ulopyranosid]onate (**20i**)

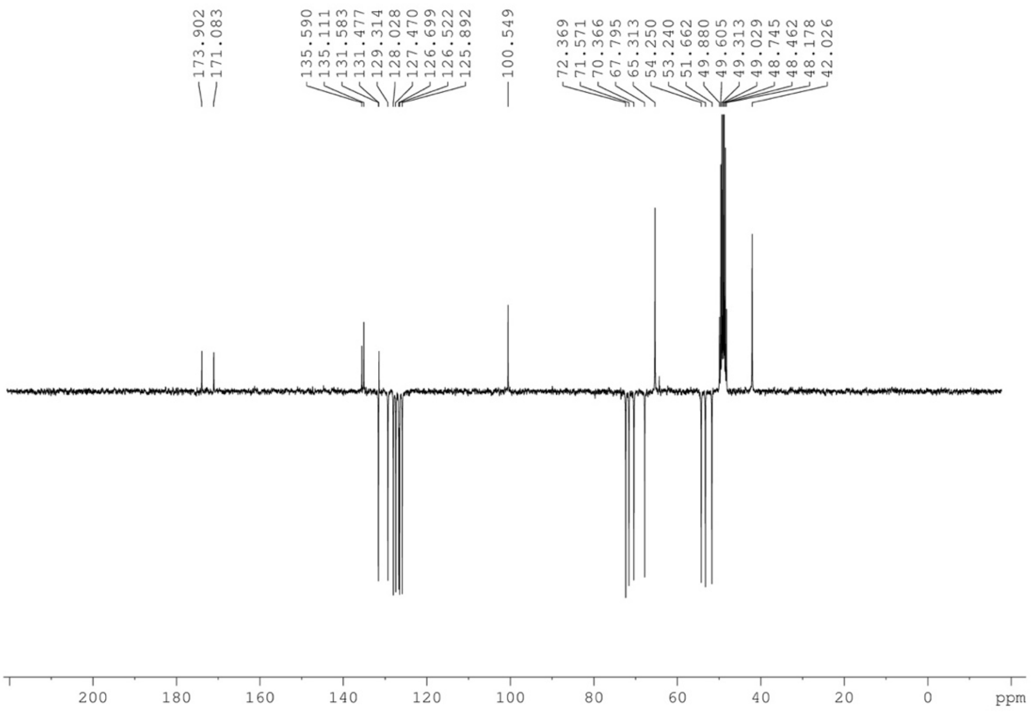

^{1}H NMR (D_2O): Methyl 5-butanamido-3,5-dideoxy-D-*glycero*-β-D-*galacto*-non-2-ulopyranosidonic acid, sodium salt (**21a**)

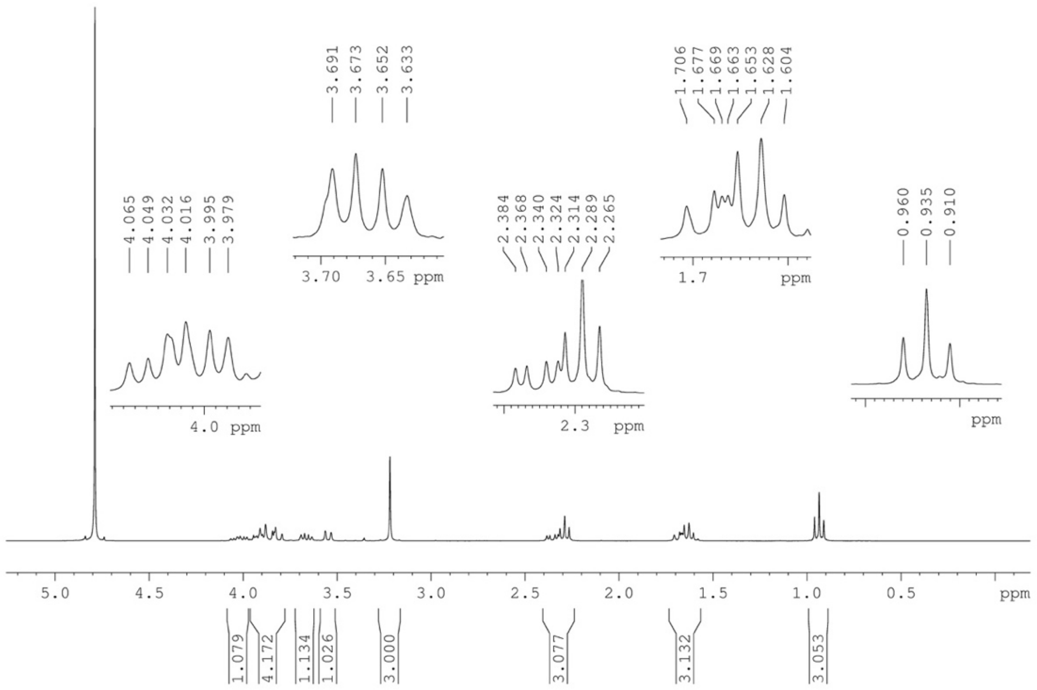

^{13}C NMR (D_2O): Methyl 5-butanamido-3,5-dideoxy-D-*glycero*-β-D-*galacto*-non-2-ulopyranosidonic acid, sodium salt (**21a**)

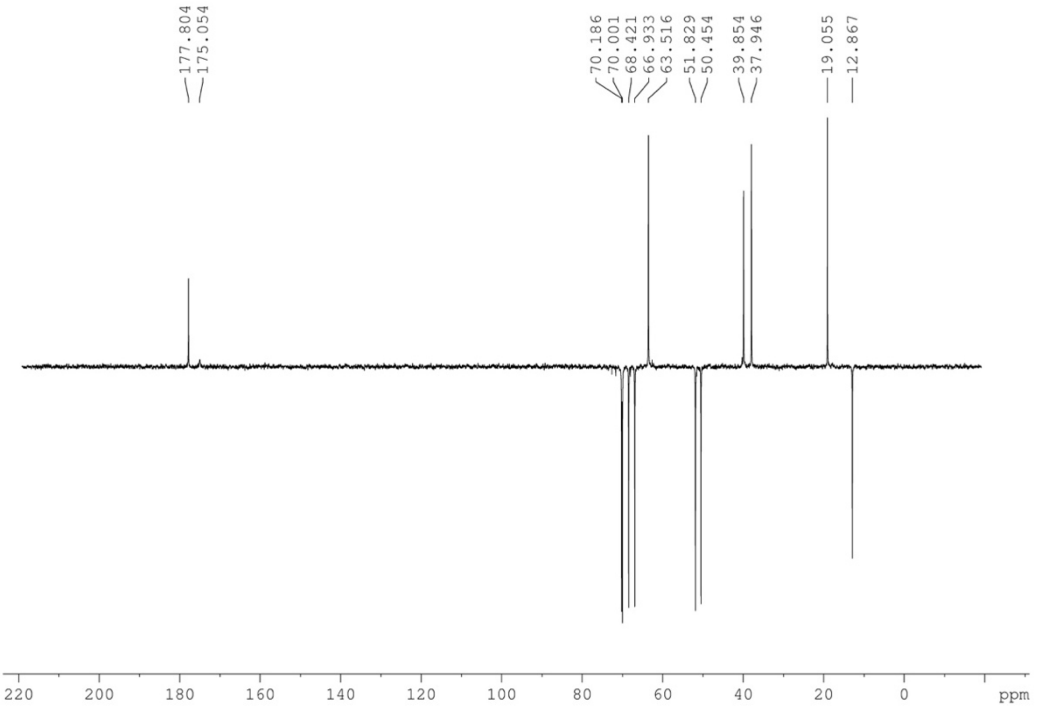

^{1}H NMR (D_2O): Methyl 5-cyclopentylcarboxamido-3,5-dideoxy-D-*glycero*-β-D-*galacto*-non-2-ulopyranosidonic acid, sodium salt (**21b**)

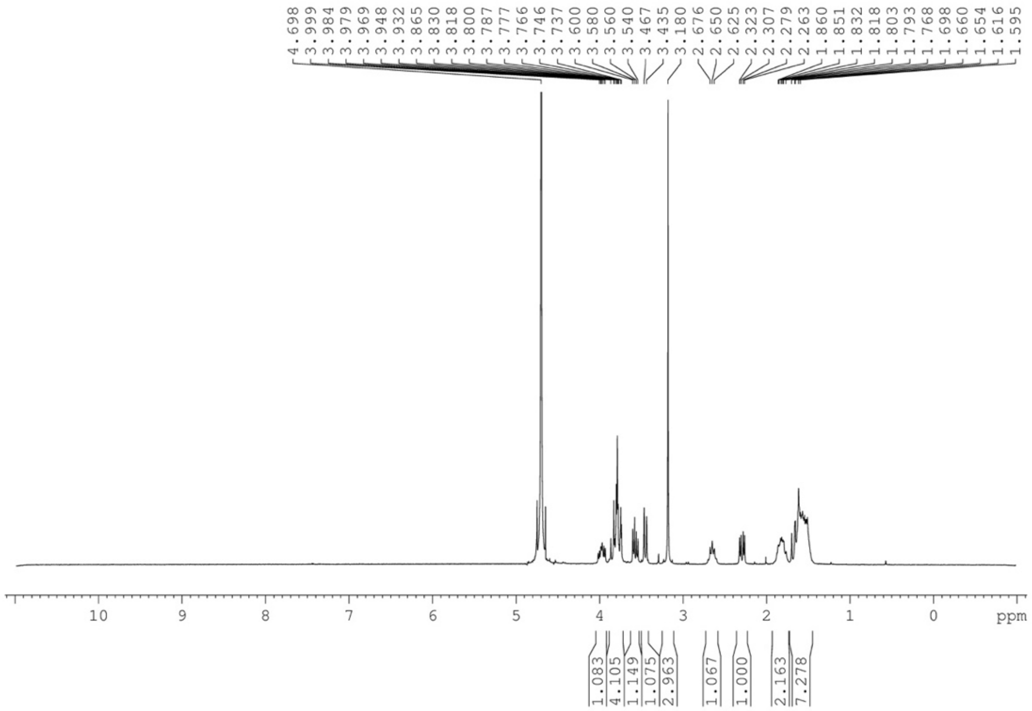

^{13}C NMR (D_2O): Methyl 5-cyclopentylcarboxamido-3,5-dideoxy-D-*glycero*-β-D-*galacto*-non-2-ulopyranosidonic acid, sodium salt (**21b**)

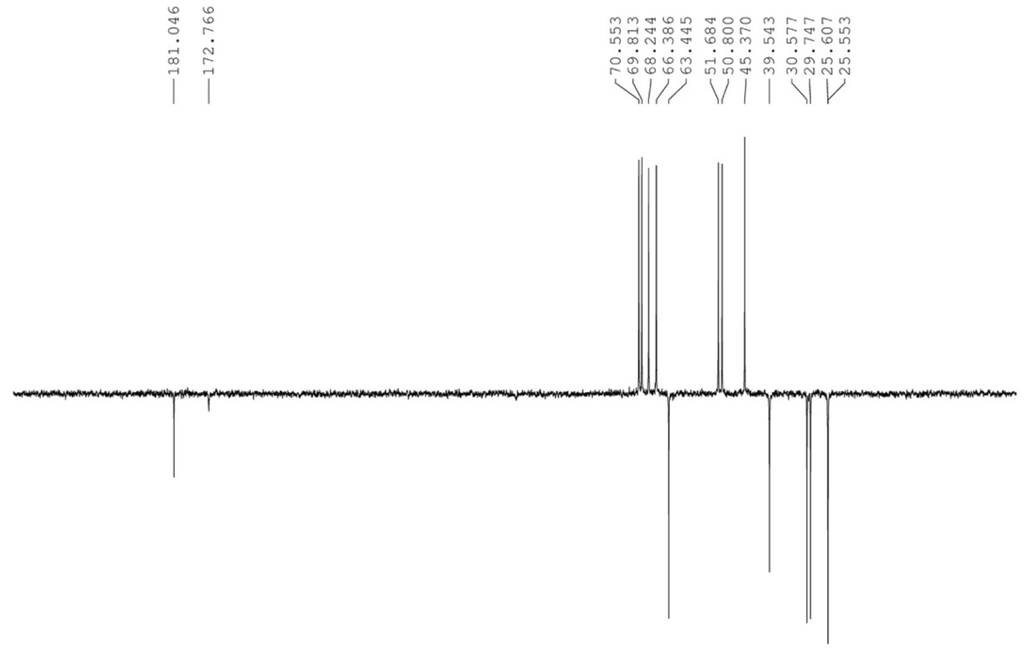

^{1}H NMR (D_2O): Methyl 5-cyclohexylcarboxamido-3,5-dideoxy-D-*glycero*-β-D-*galacto*-non-2-ulopyranosidonic acid, sodium salt (**21c**)

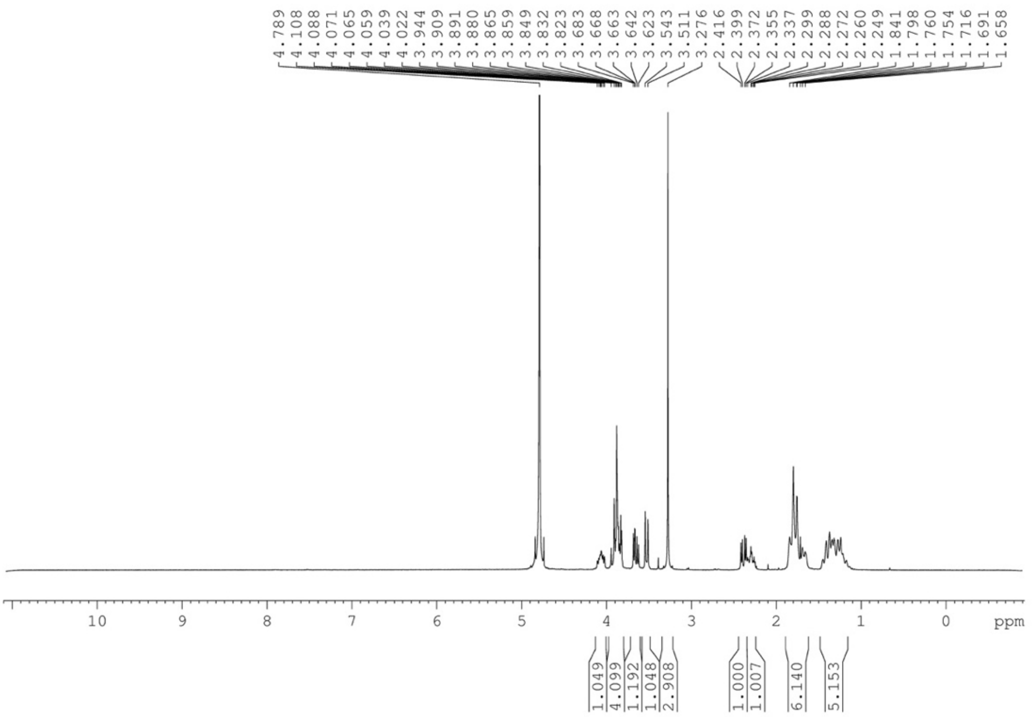

^{13}C NMR (D_2O): Methyl 5-cyclohexylcarboxamido-3,5-dideoxy-D-*glycero*-β-D-*galacto*-non-2-ulopyranosidonic acid, sodium salt (**21c**)

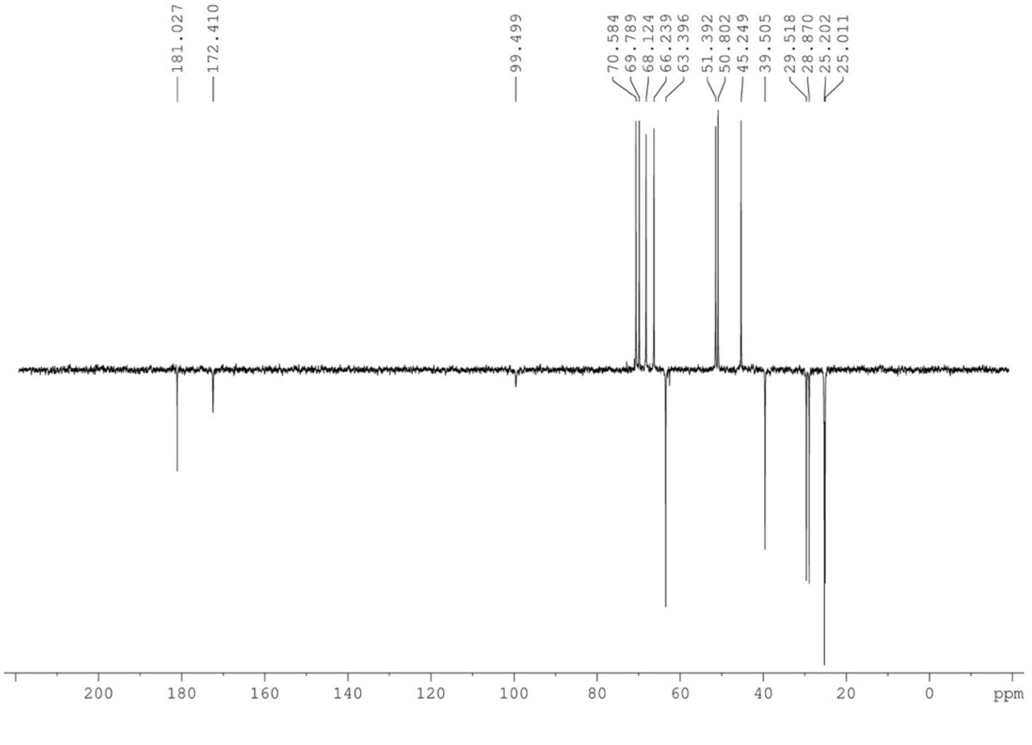

^{1}H NMR (D_2O): Methyl 3,5-dideoxy-5-(2-hydroxyacetamido)-D-*glycero*-β-D-*galacto*-non-2-ulopyranosidonic acid (**21d**)

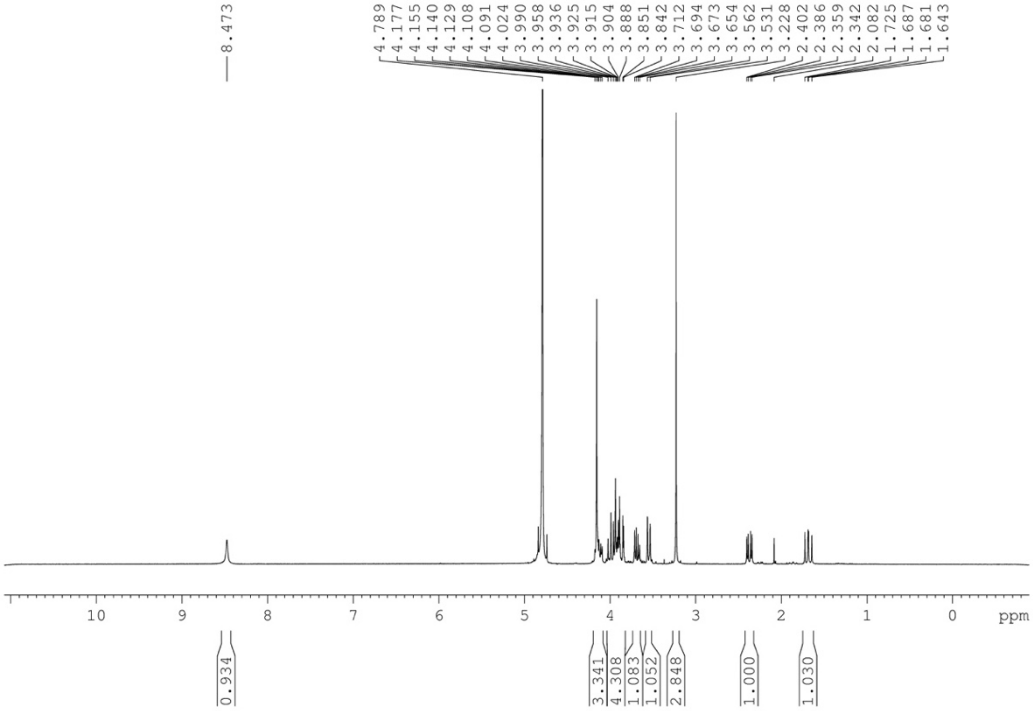

^{13}C NMR (D_2O): Methyl 3,5-dideoxy-5-(2-hydroxyacetamido)-D-*glycero*-β-D-*galacto*-non-2-ulopyranosidonic acid (**21d**)

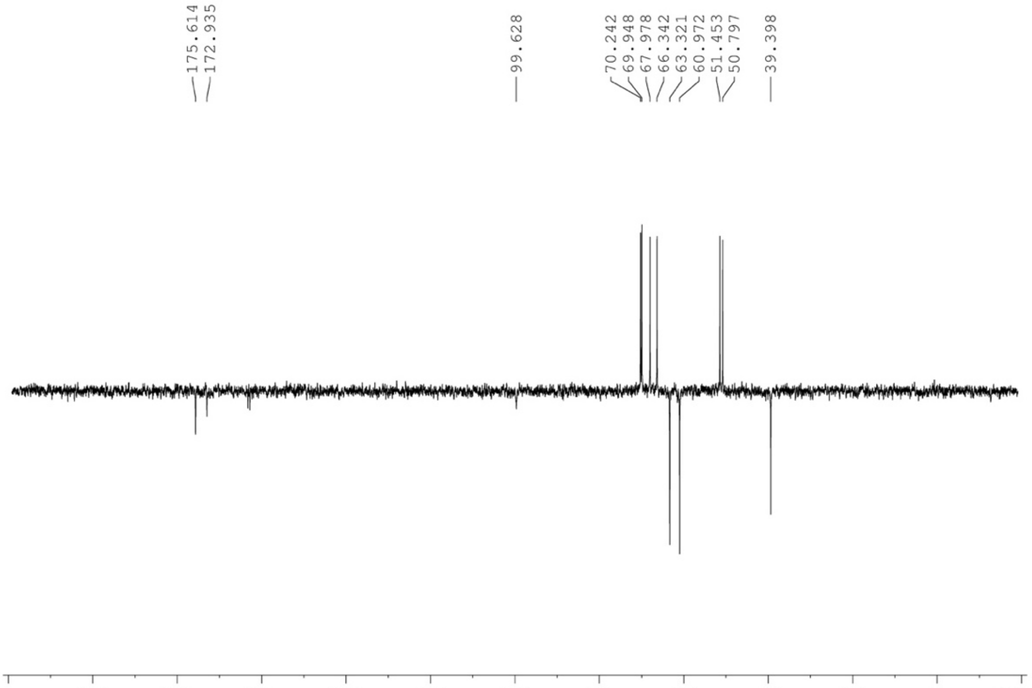

^{1}H NMR (D_2O): Methyl 5-(5-carboxy-pentamido)-3,5-dideoxy-D-*glycero*-β-D-*galacto*-non-2-ulopyranosidonic acid, sodium salt (**21e**)

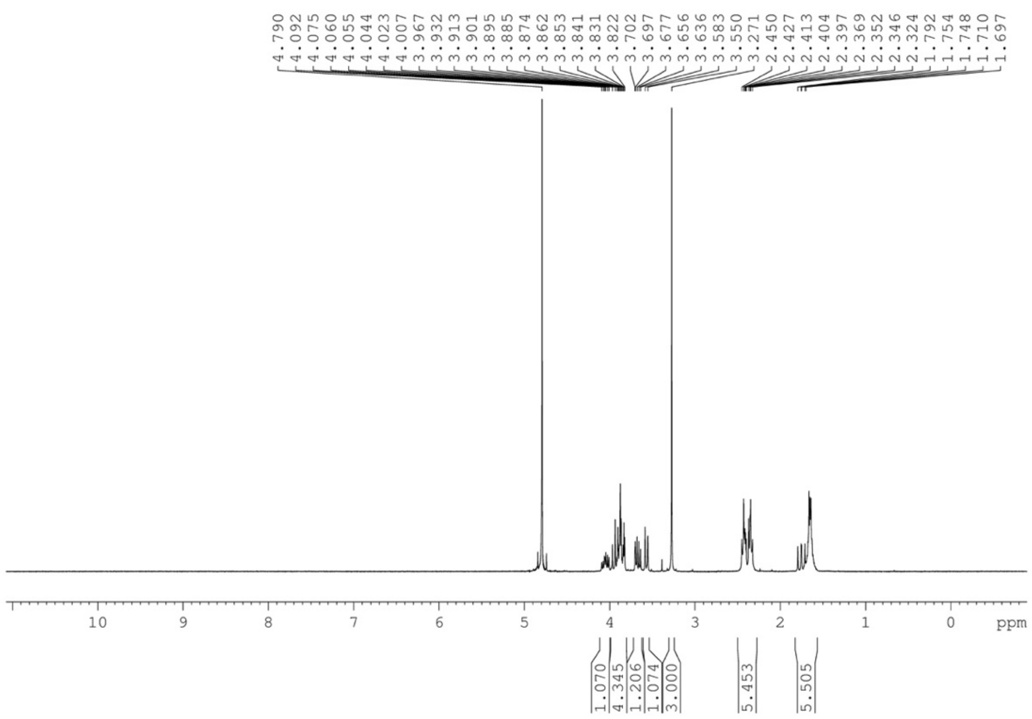

^{13}C NMR (D_2O): Methyl 5-(5-carboxy-pentamido)-3,5-dideoxy-D-*glycero*-β-D-*galacto*-non-2-ulopyranosidonic acid, sodium salt (**21e**)

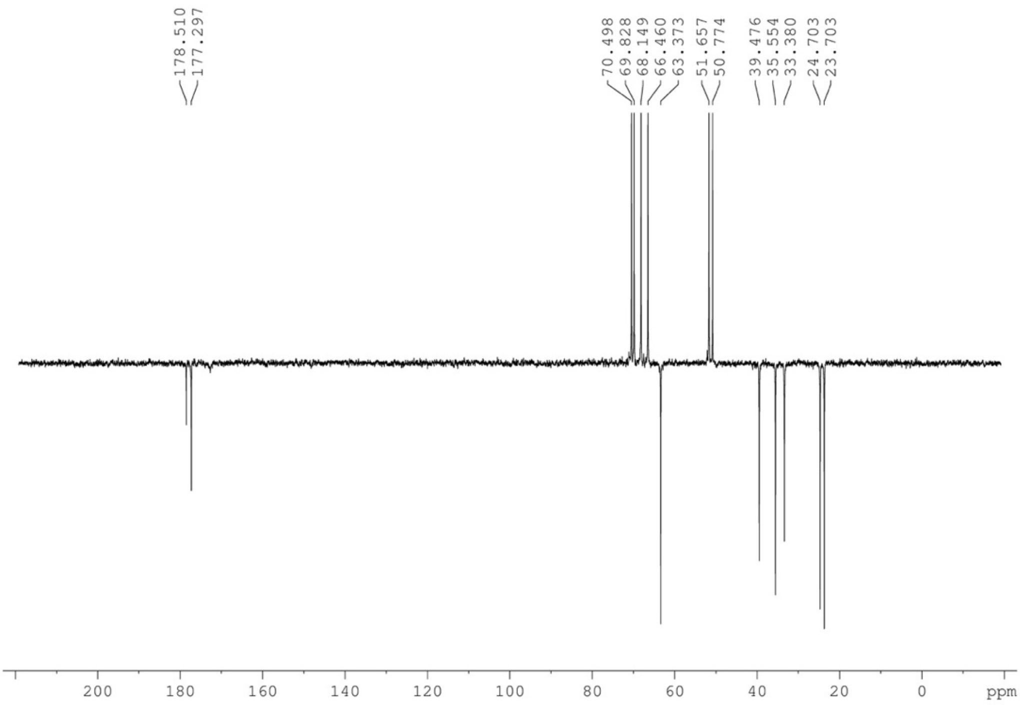

^{1}H NMR (D_2O): Methyl 3,5-dideoxy-5-(3-phenyl-propanamido)-D-*glycero*-β-D-*galacto*-non-2-ulopyranosidonic acid, sodium salt (**21f**)

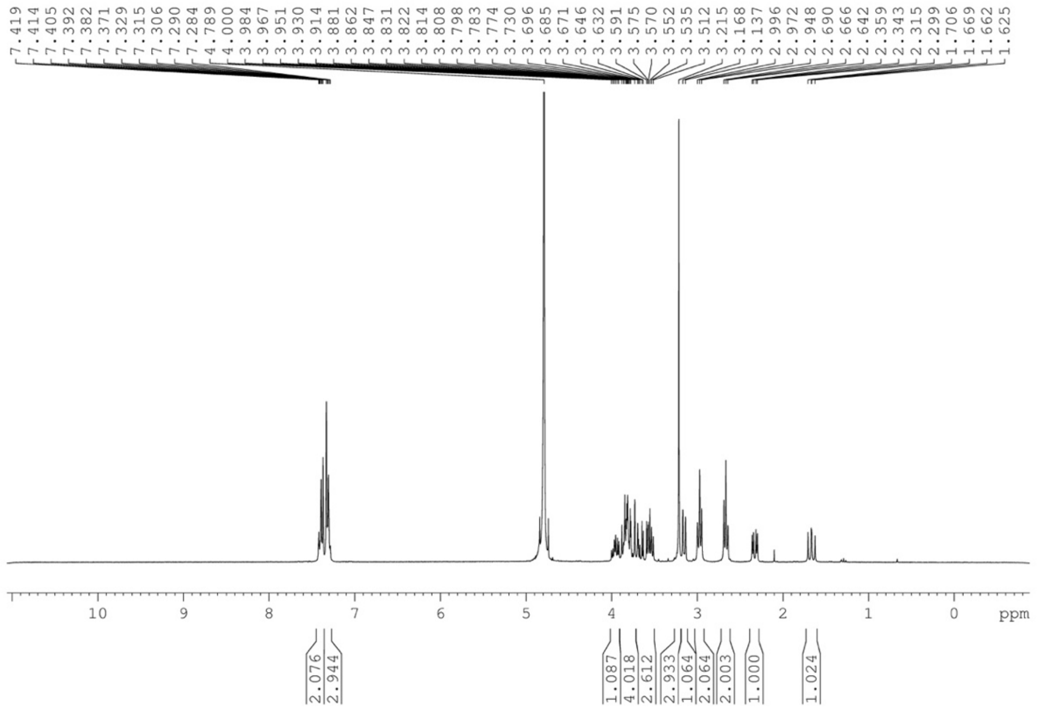

^{13}C NMR (D_2O): Methyl 3,5-dideoxy-5-(3-phenyl-propanamido)-D-*glycero*-β-D-*galacto*-non-2-ulopyranosidonic acid, sodium salt (**21f**)

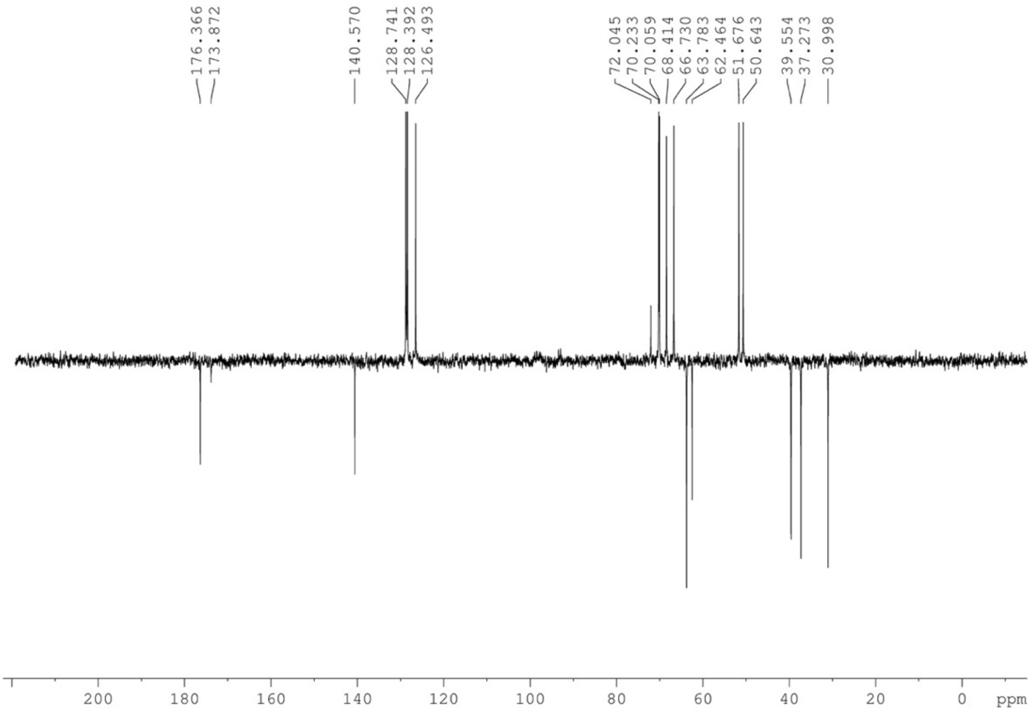

^{1}H NMR (D_2O): Methyl 3,5-dideoxy-5-(4-phenoxy-butanamido)-D-*glycero*-β-D-*galacto*-non-2-ulopyranosidonic acid, sodium salt (**21g**)

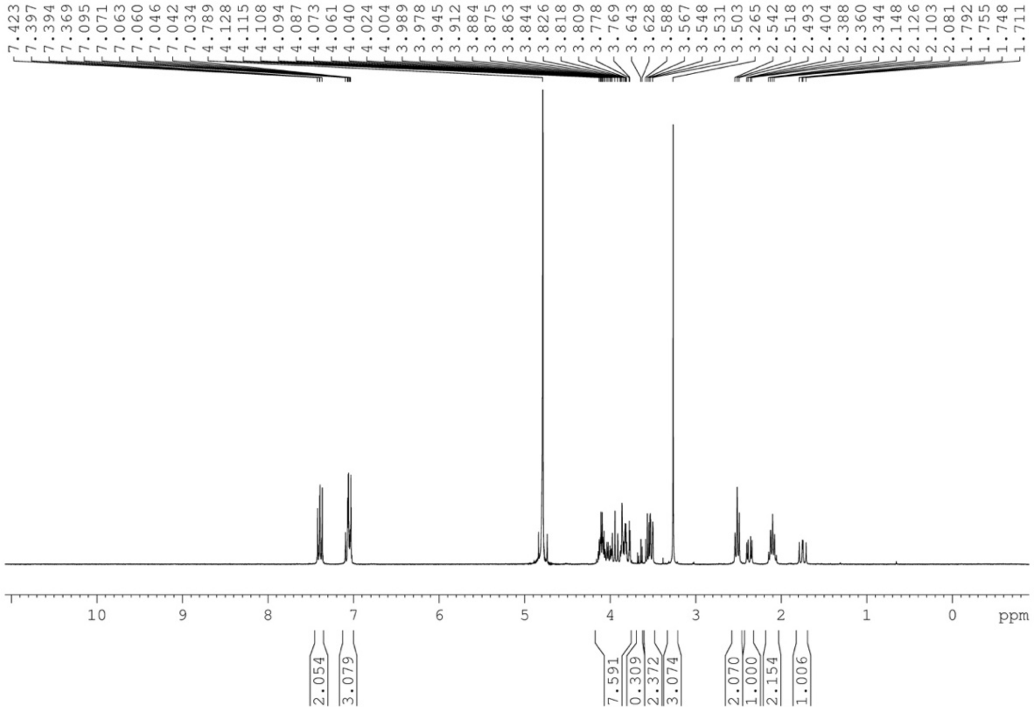

^{13}C NMR (D_2O): Methyl 3,5-dideoxy-5-(4-phenoxy-butanamido)-D-*glycero*-β-D-*galacto*-non-2-ulopyranosidonic acid, sodium salt (**21g**)

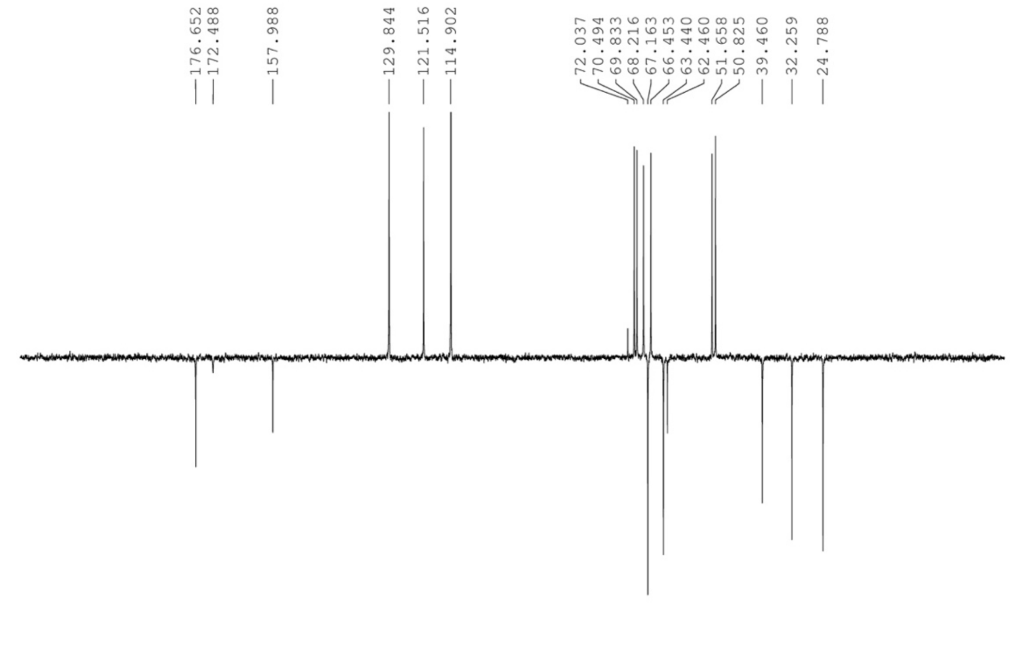

^{1}H NMR (CD_3OD): Methyl 3,5-dideoxy-5-(4-phenyl-benzamido)-D-*glycero*-β-D-*galacto*-non-2-ulopyranosidonic acid, sodium salt (**21h**)

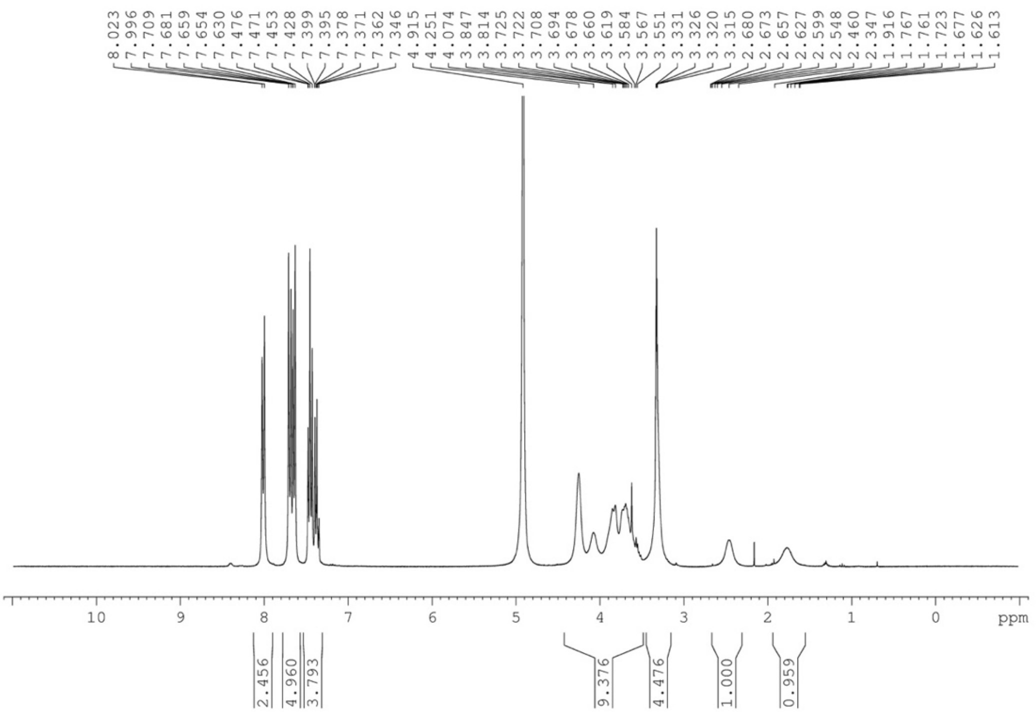

^{13}C NMR (CD_3OD): Methyl 3,5-dideoxy-5-(4-phenyl-benzamido)-D-*glycero*-β-D-*galacto*-non-2-ulopyranosidonic acid, sodium salt (**21h**)

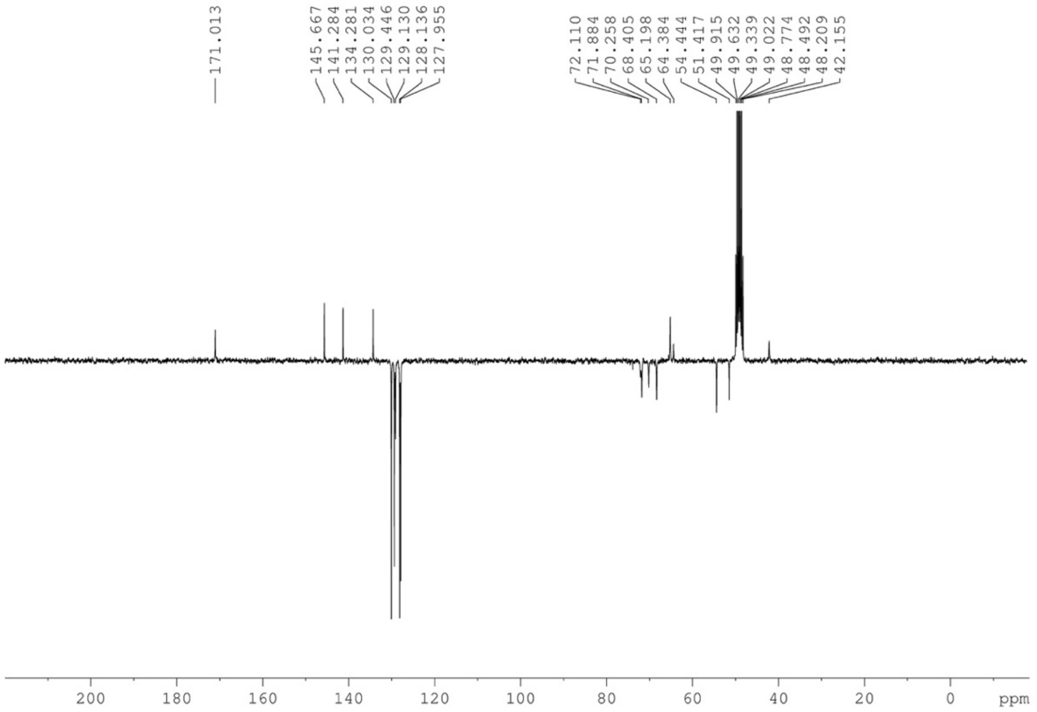

^{1}H NMR (D_2O): Methyl 3,5-dideoxy-5-(1-naphthylcarboxamido)-D-*glycero*-β-D-*galacto*-non-2-ulopyranosidonic acid, partial triethylammonium salt (**21i**)

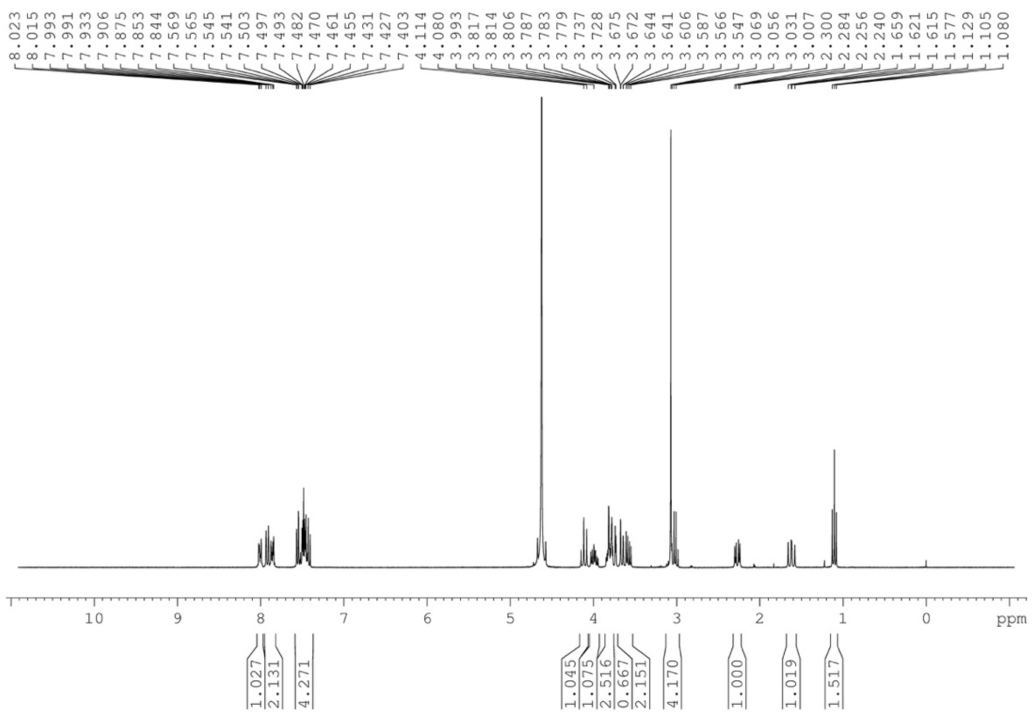

^{13}C NMR (D_2O): Methyl 3,5-dideoxy-5-(1-naphthylcarboxamido)-D-*glycero*-β-D-*galacto*-non-2-ulopyranosidonic acid, partial triethylammonium salt (**21i**)

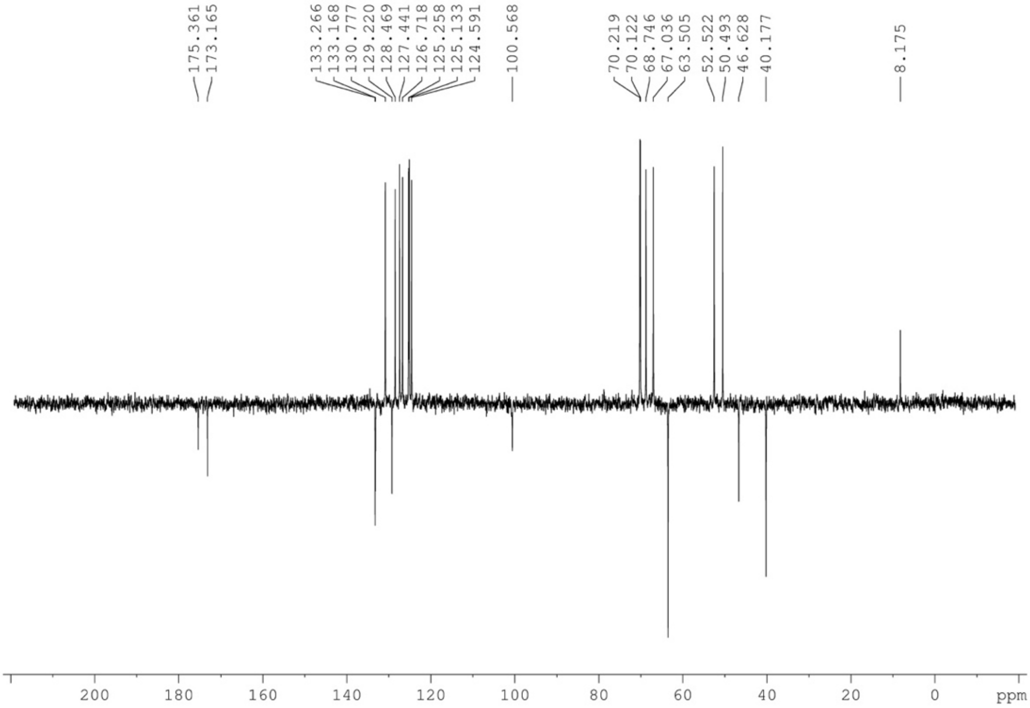

^{1}H NMR ($CDCl_3$): Methyl (methyl 5-acetamido-7,8,9-tri-*O*-acetyl-4-amino-3,4,5-trideoxy-D-*glycero*-β-D-*galacto*-non-2-ulopyranosid)onate (**25**)

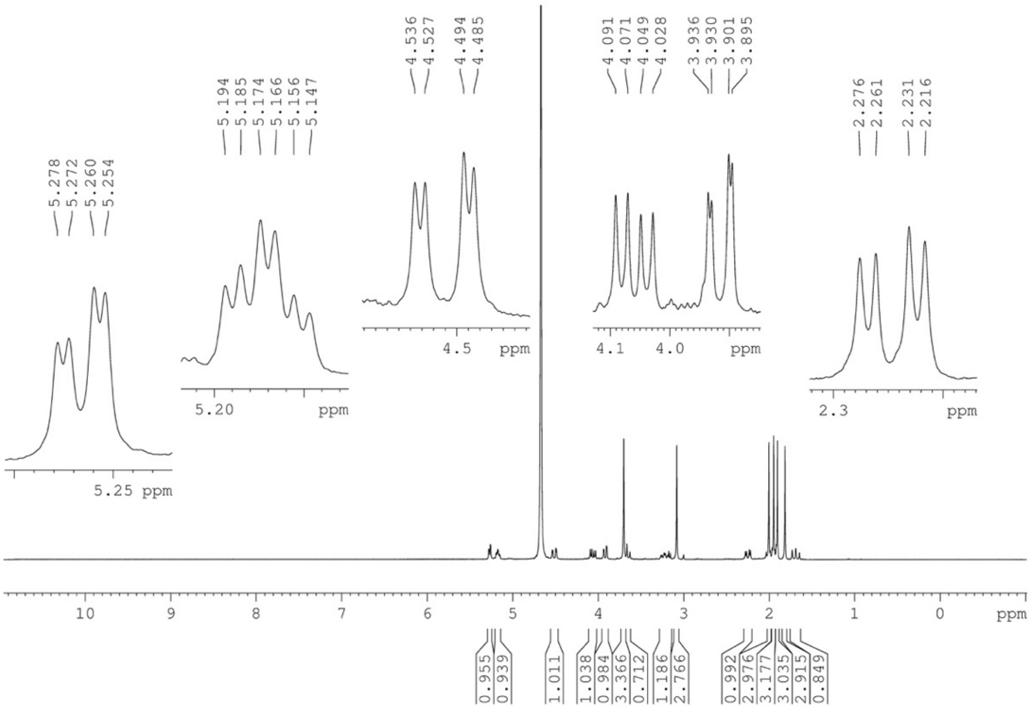

^{1}H NMR ($CDCl_3$): Methyl [methyl 5-acetamido-7,8,9-tri-*O*-acetyl-4-butanamido-3,4,5-trideoxy-D-*glycero*-β-D-*galacto*-non-2-ulopyranosid]onate (**26a**)

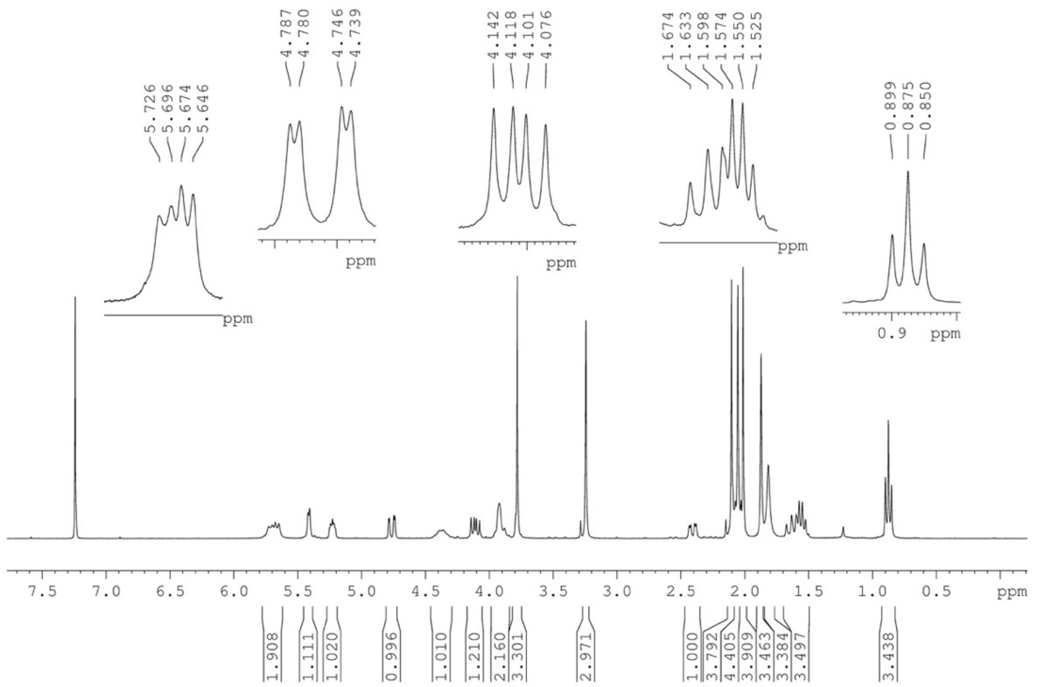

^{13}C NMR ($CDCl_3$): Methyl [methyl 5-acetamido-7,8,9-tri-*O*-acetyl-4-butanamido-3,4,5-trideoxy-D-*glycero*-β-D-*galacto*-non-2-ulopyranosid]onate (**26a**)

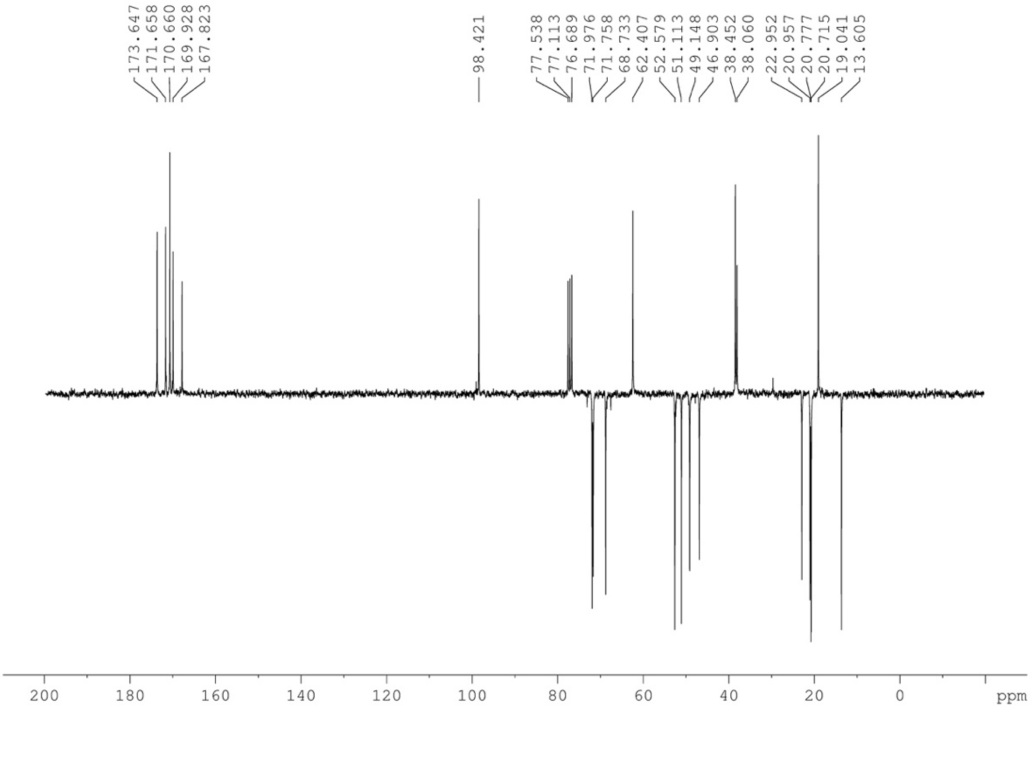

^{1}H NMR ($CDCl_3$): Methyl [methyl 5-acetamido-7,8,9-tri-*O*-acetyl-3,4,5-trideoxy-4-hexanamido-D-*glycero*-β-D-*galacto*-non-2-ulopyranosid]onate (**26b**)

[Note: there are impurities present in the provided spectrum; it is included for reference only]

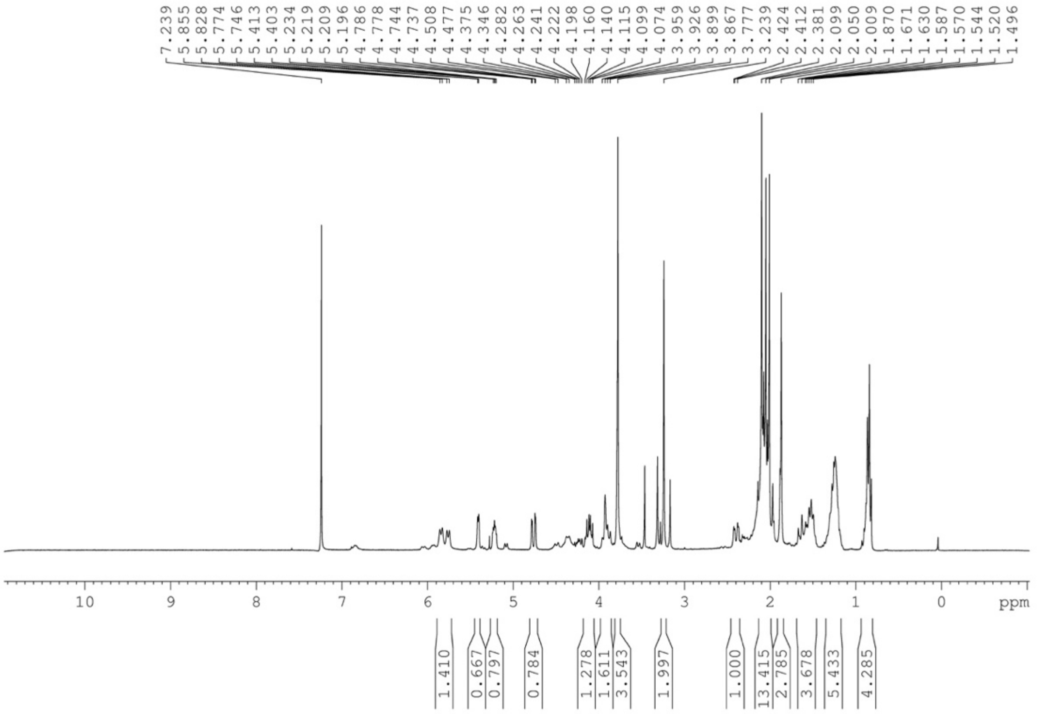

^{13}C NMR ($CDCl_3$): Methyl [methyl 5-acetamido-7,8,9-tri-*O*-acetyl-3,4,5-trideoxy-4-hexanamido-D-*glycero*-β-D-*galacto*-non-2-ulopyranosid]onate (**26b**)

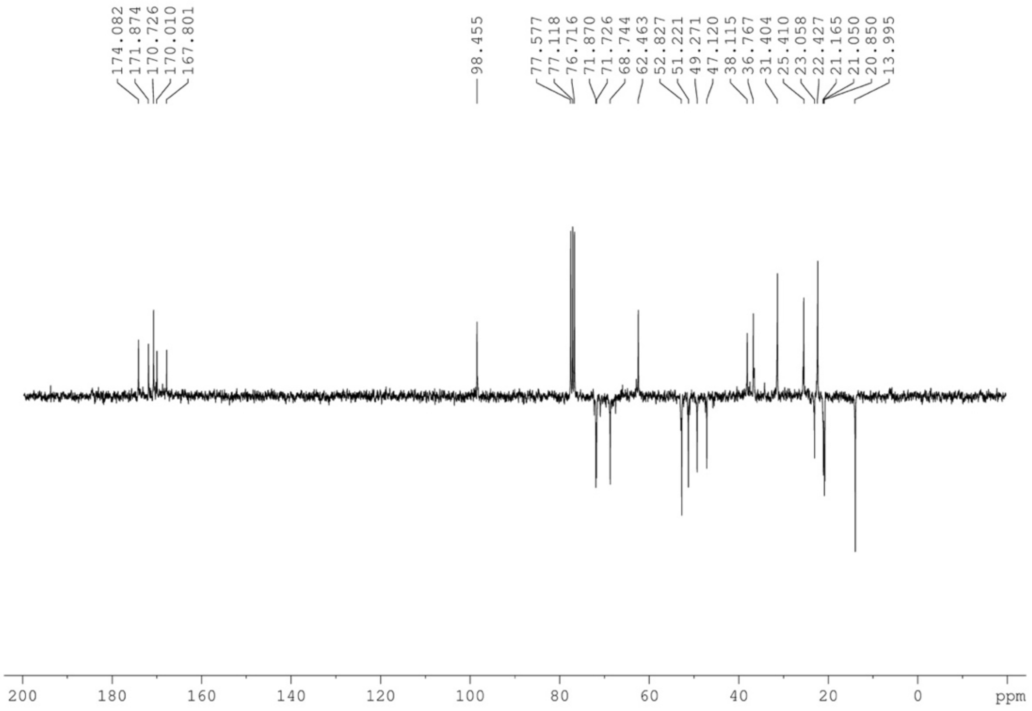

^{1}H NMR ($CDCl_3$): Methyl [methyl 5-acetamido-7,8,9-tri-*O*-acetyl-3,4,5-trideoxy-4-(2-ethyl-butanamido)-D-*glycero*-β-D-*galacto*-non-2-ulopyranosid]onate (**26c**)

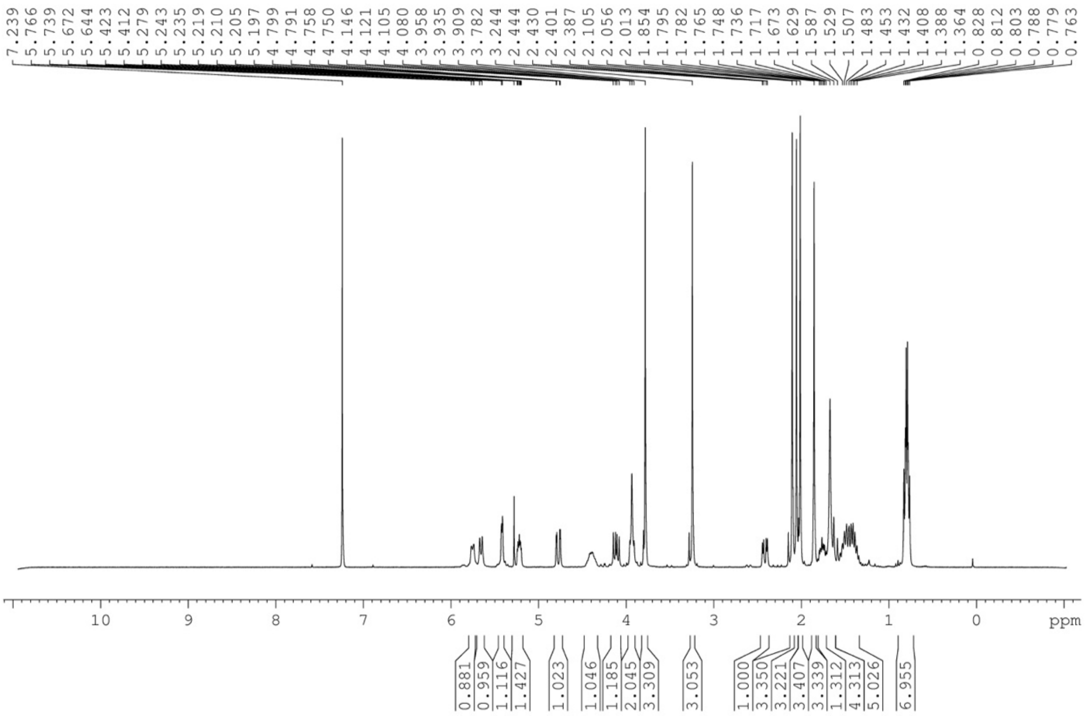

^{13}C NMR ($CDCl_3$): Methyl [methyl 5-acetamido-7,8,9-tri-*O*-acetyl-3,4,5-trideoxy-4-(2-ethyl-butanamido)-D-*glycero*-β-D-*galacto*-non-2-ulopyranosid]onate (**26c**)

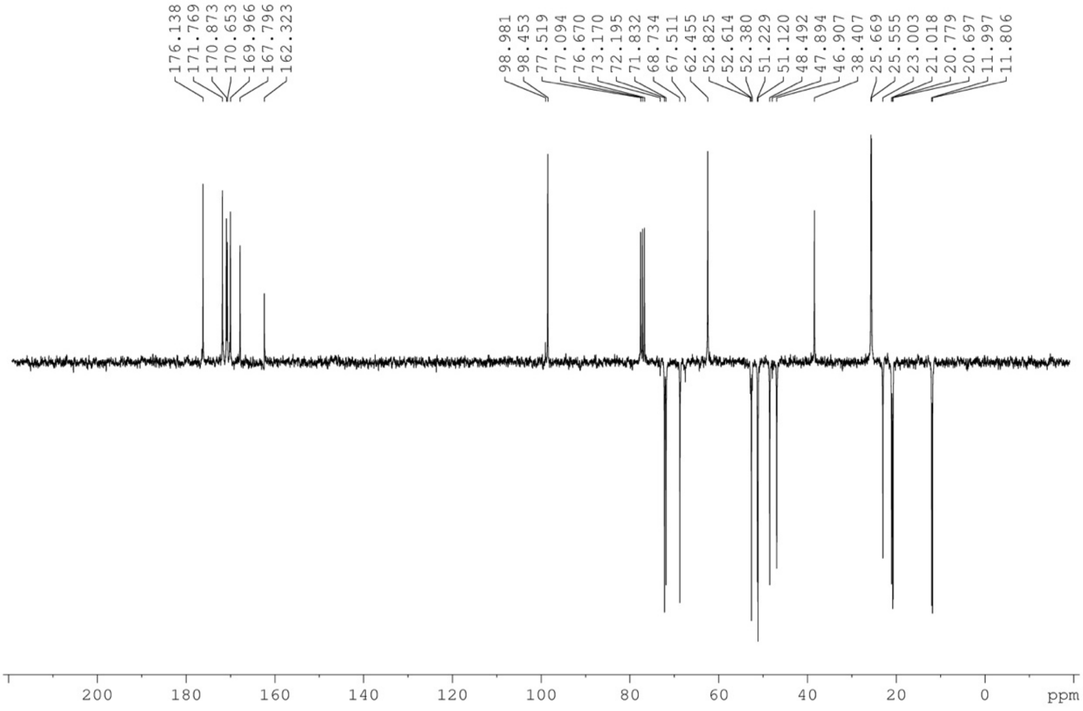

^{1}H NMR ($CDCl_3$): Methyl [methyl 5-acetamido-7,8,9-tri-*O*-acetyl-3,4,5-trideoxy-4-(2-ethyl-hexanamido)-D-*glycero*-β-D-*galacto*-non-2-ulopyranosid]onate (**26d**)

[Note: impurity is present at 1.6 ppm in the provided spectrum]

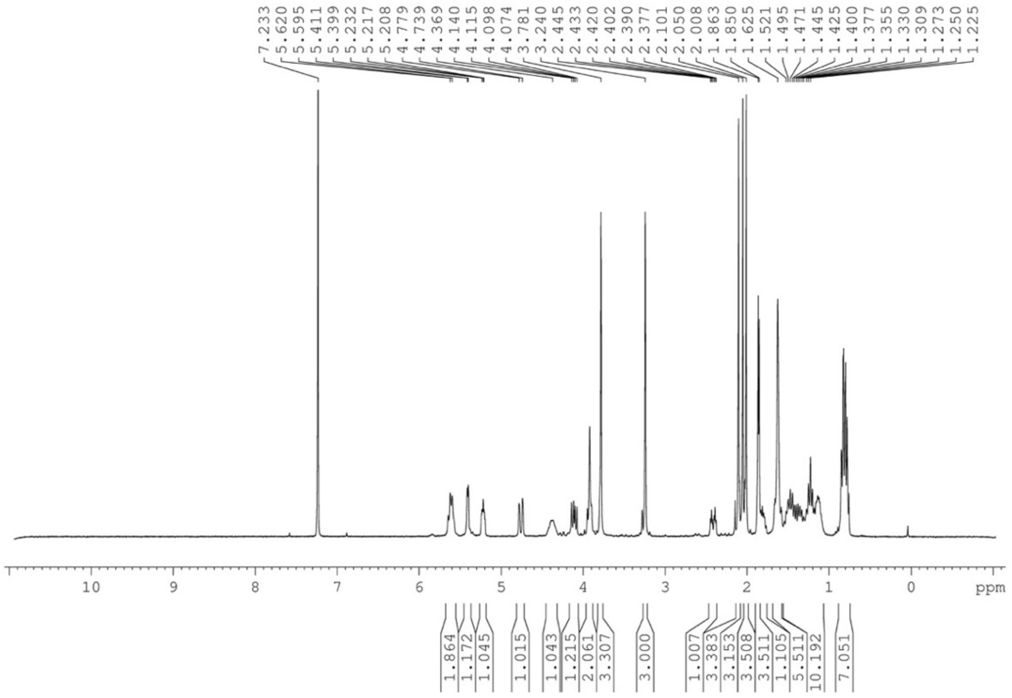

^{13}C NMR ($CDCl_3$): Methyl [methyl 5-acetamido-7,8,9-tri-*O*-acetyl-3,4,5-trideoxy-4-(2-ethyl-hexanamido)-D-*glycero*-β-D-*galacto*-non-2-ulopyranosid]onate (**26d**)

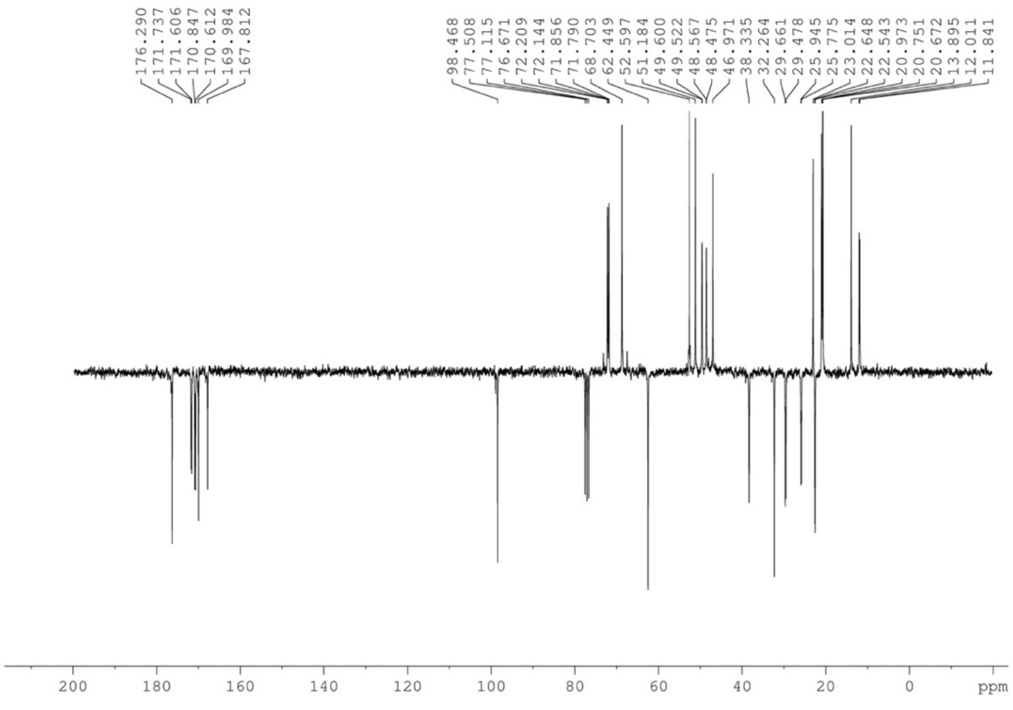

^{1}H NMR ($CDCl_3$): Methyl [methyl 5-acetamido-7,8,9-tri-*O*-acetyl-3,4,5-trideoxy-4-(3-nitrobenzamido)-D-*glycero*-β-D-*galacto*-non-2-ulopyranosid]onate (**26e**)

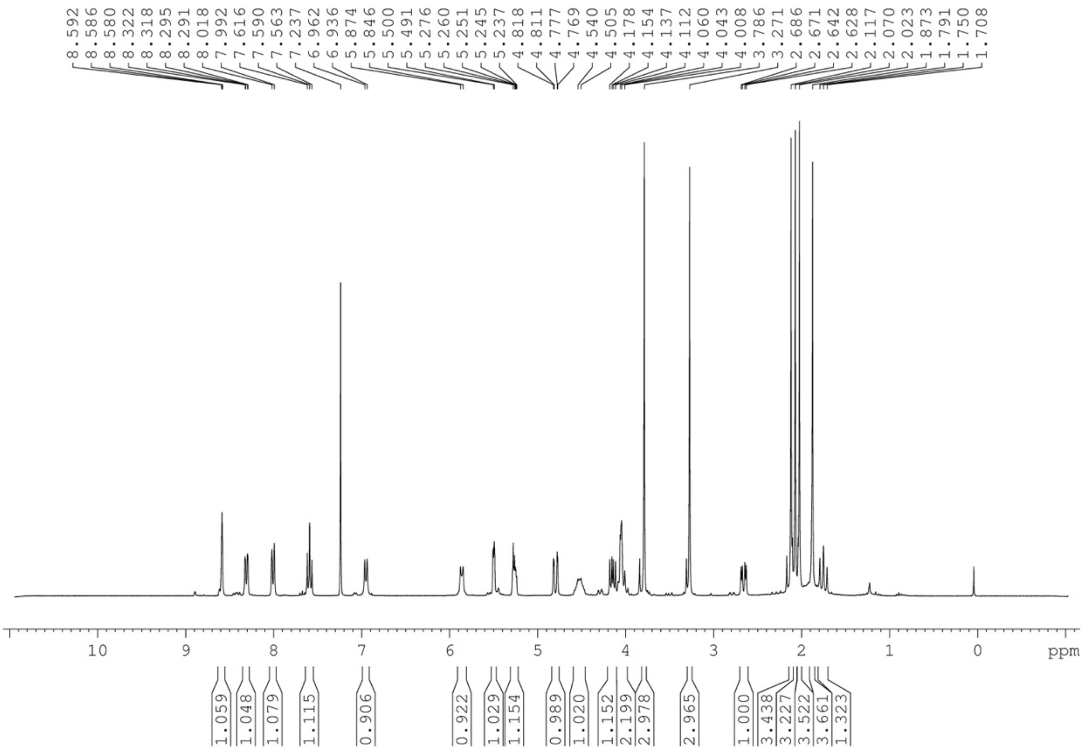

^{13}C NMR ($CDCl_3$): Methyl [methyl 5-acetamido-7,8,9-tri-*O*-acetyl-3,4,5-trideoxy-4-(3-nitrobenzamido)-D-*glycero*-β-D-*galacto*-non-2-ulopyranosid]onate (**26e**)

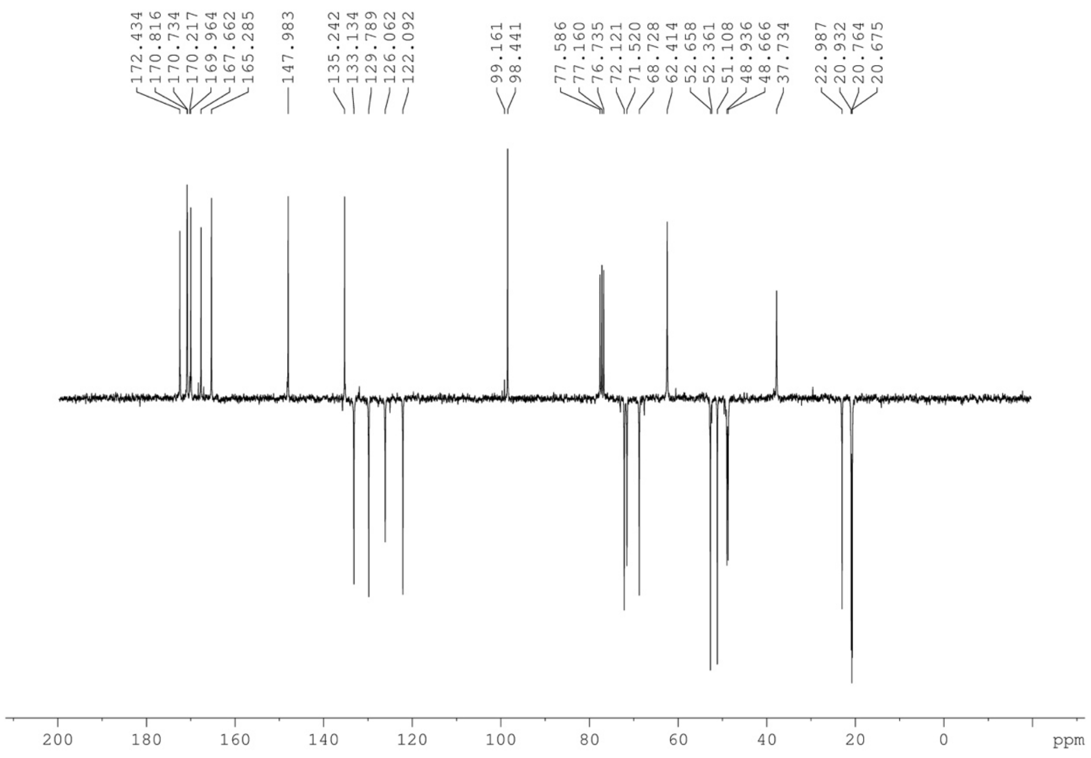

^{1}H NMR ($CDCl_3$): Methyl [methyl 5-acetamido-7,8,9-tri-*O*-acetyl-3,4,5-trideoxy-4-(3-phenyl-propanamido)-D-*glycero*-β-D-*galacto*-non-2-ulopyranosid]onate (**26f**)

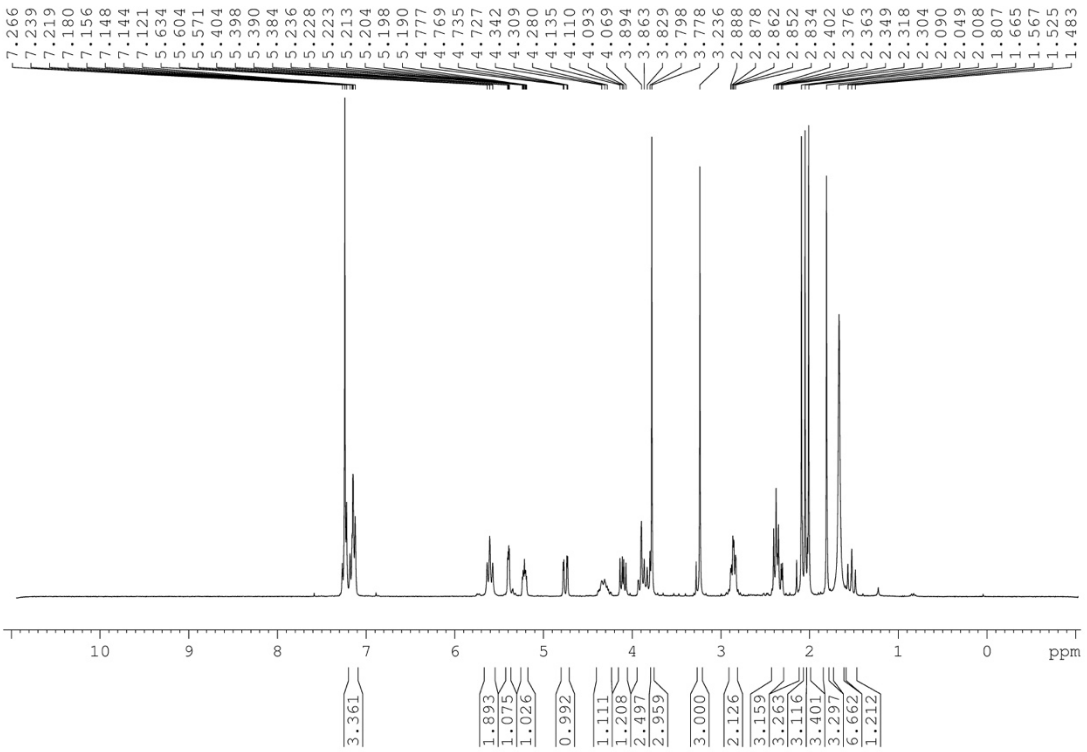

^{13}C NMR ($CDCl_3$): Methyl [methyl 5-acetamido-7,8,9-tri-*O*-acetyl-3,4,5-trideoxy-4-(3-phenyl-propanamido)-D-*glycero*-β-D-*galacto*-non-2-ulopyranosid]onate (**26f**)

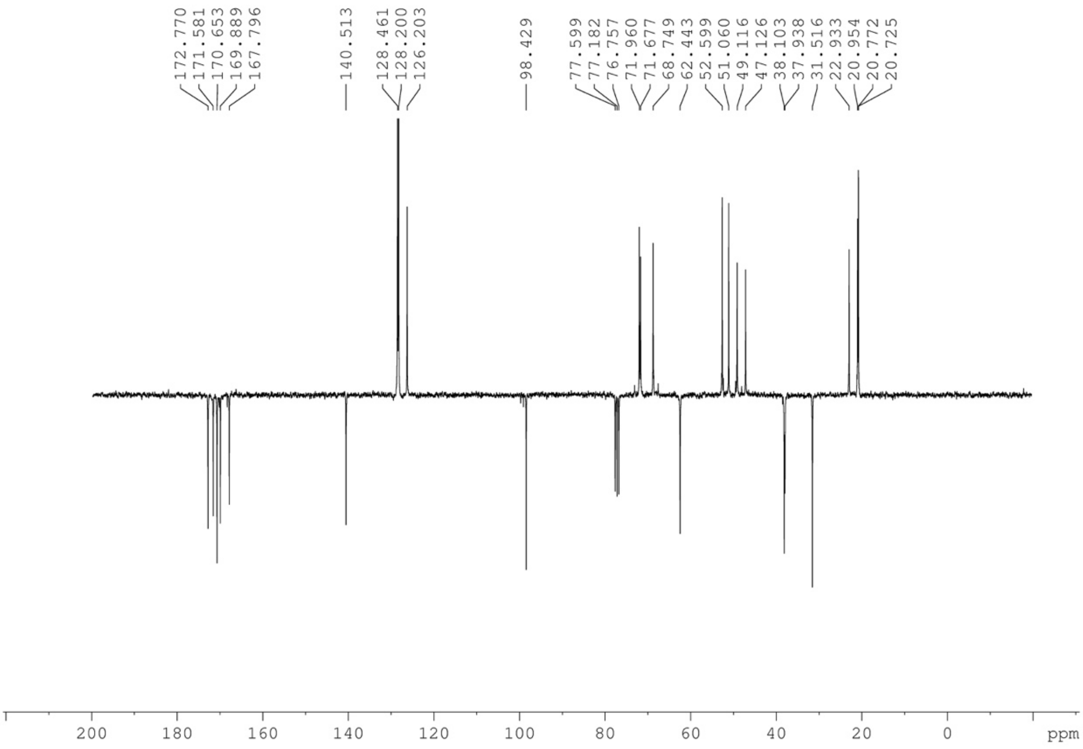

^{1}H NMR ($CDCl_3$): Methyl [methyl 5-acetamido-7,8,9-tri-*O*-acetyl-3,4,5-trideoxy-4-(4-phenoxy-butanamido)-D-*glycero*-β-D-*galacto*-non-2-ulopyranosid]onate (**26g**)

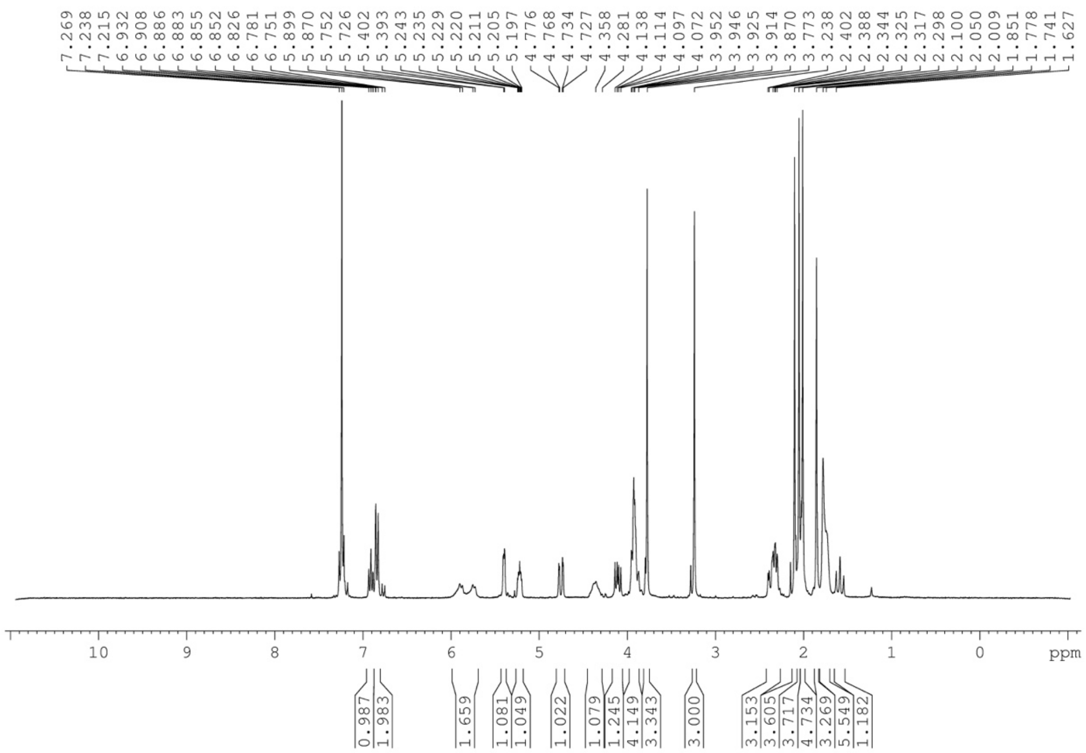

^{13}C NMR ($CDCl_3$): Methyl [methyl 5-acetamido-7,8,9-tri-*O*-acetyl-3,4,5-trideoxy-4-(4-phenoxy-butanamido)-D-*glycero*-β-D-*galacto*-non-2-ulopyranosid]onate (**26g**)

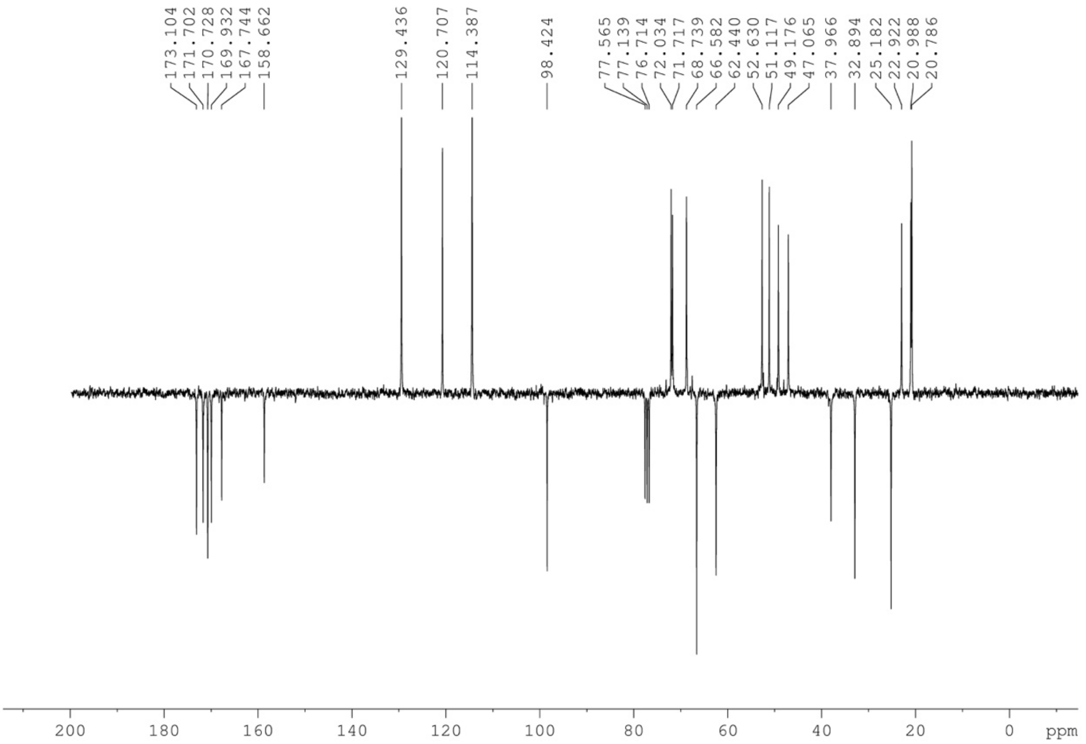

^{1}H NMR ($CDCl_3$): Methyl [methyl 5-acetamido-7,8,9-tri-*O*-acetyl-3,4,5-trideoxy-4-(4-phenyl-benzamido)-D-*glycero*-β-D-*galacto*-non-2-ulopyranosid]onate (**26h**)

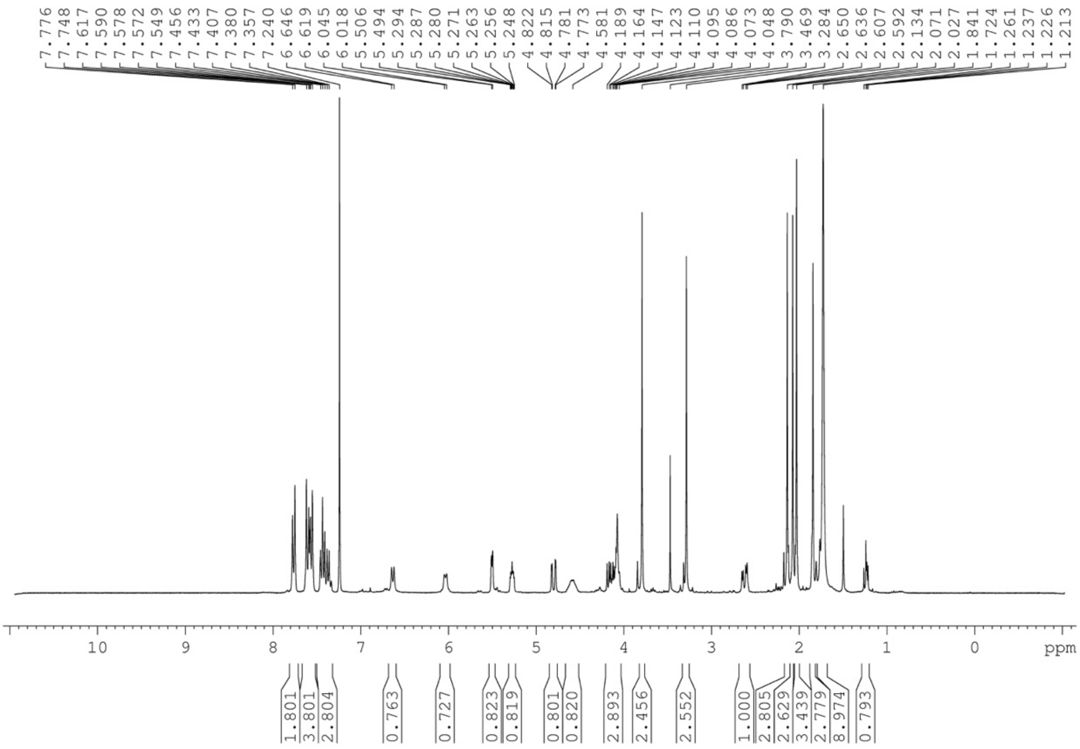

^{13}C NMR (CD_3OD): Methyl [methyl 5-acetamido-7,8,9-tri-*O*-acetyl-3,4,5-trideoxy-4-(4-phenyl-benzamido)-D-*glycero*-β-D-*galacto*-non-2-ulopyranosid]onate (**26h**)

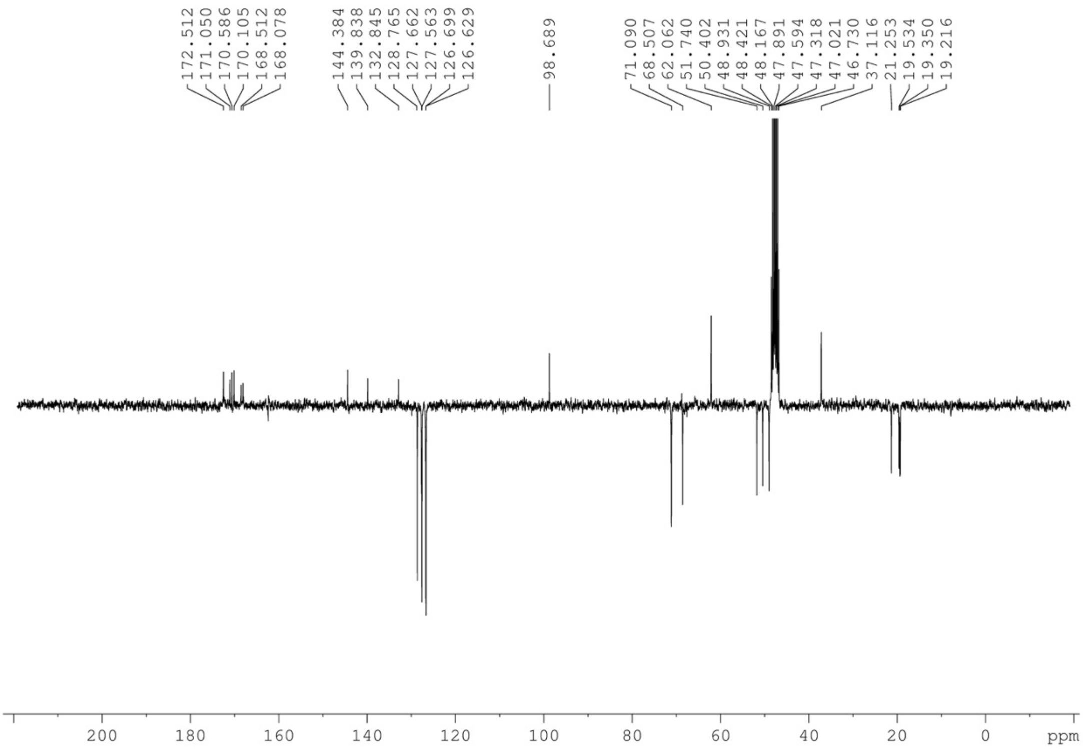

^{1}H NMR ($CDCl_3$): Methyl [methyl 5-acetamido-7,8,9-tri-*O*-acetyl-3,4,5-trideoxy-4-(1-naphthylcarboxamido)-D-*glycero*-β-D-*galacto*-non-2-ulopyranosid]onate (**26i**)

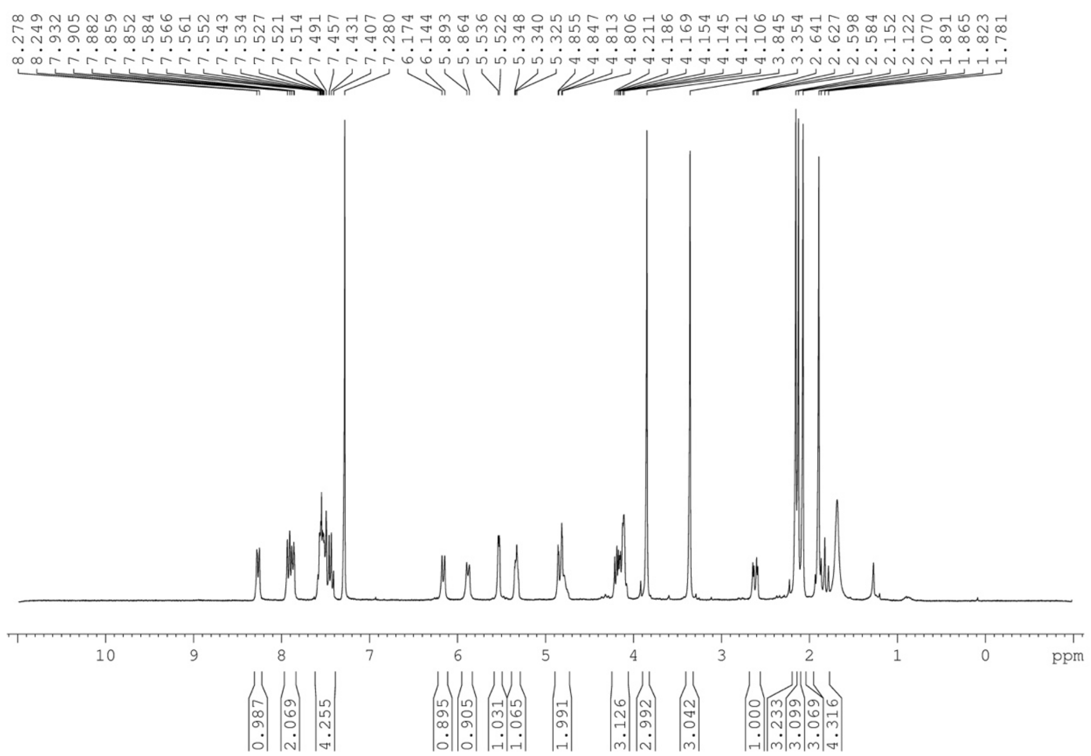

^{13}C NMR ($CDCl_3$): Methyl [methyl 5-acetamido-7,8,9-tri-*O*-acetyl-3,4,5-trideoxy-4-(1-naphthylcarboxamido)-D-*glycero*-β-D-*galacto*-non-2-ulopyranosid]onate (**26i**)

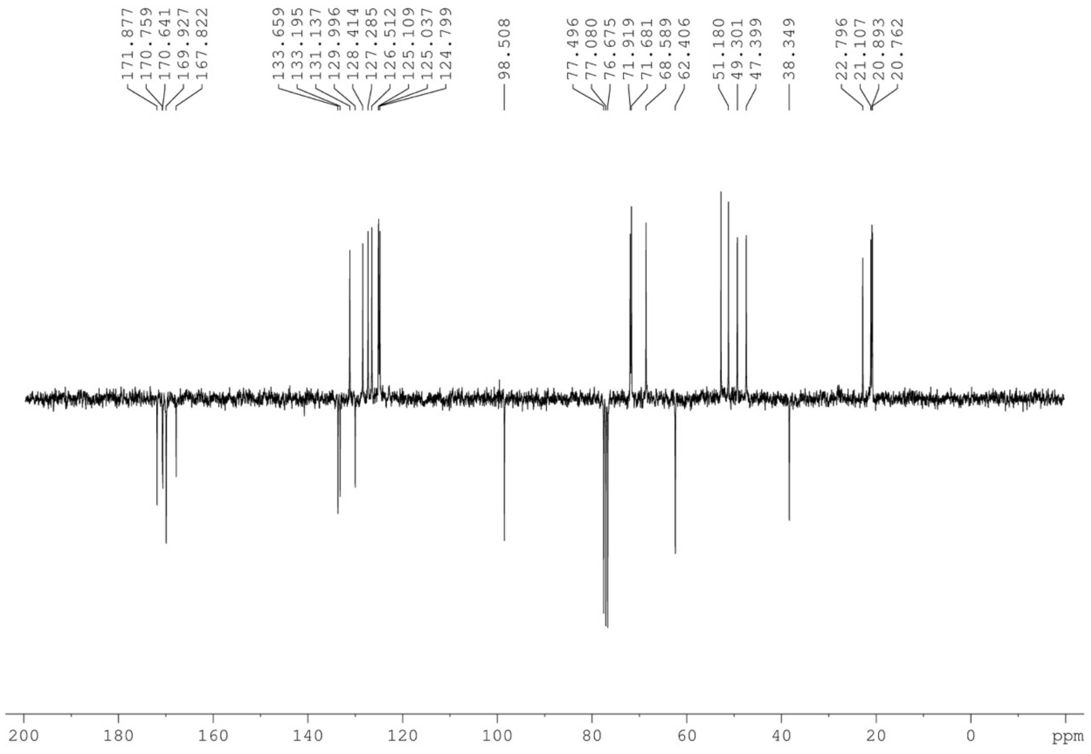

^{1}H NMR (D_2O): Methyl 5-acetamido-4-butanamido-3,4,5-trideoxy-D-*glycero*-β-D-*galacto*-non-2-ulopyranosidonic acid, lithium salt (**27a**)

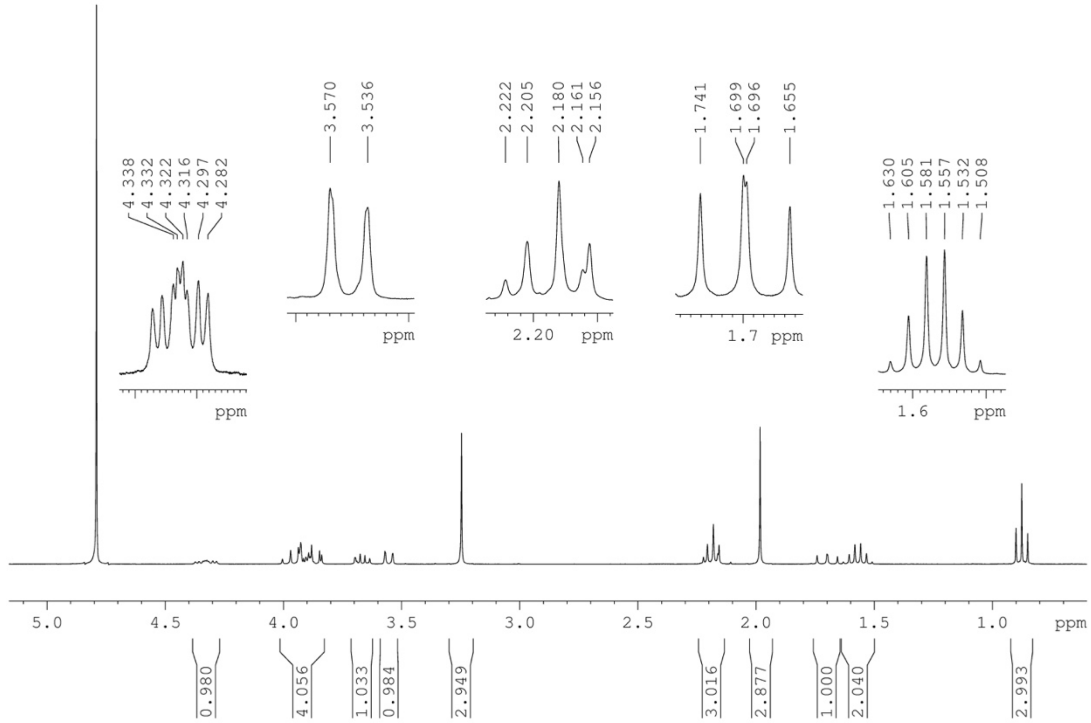

^{13}C NMR (D_2O): Methyl 5-acetamido-4-butanamido-3,4,5-trideoxy-D-*glycero*-β-D-*galacto*-non-2-ulopyranosidonic acid, lithium salt (**27a**)

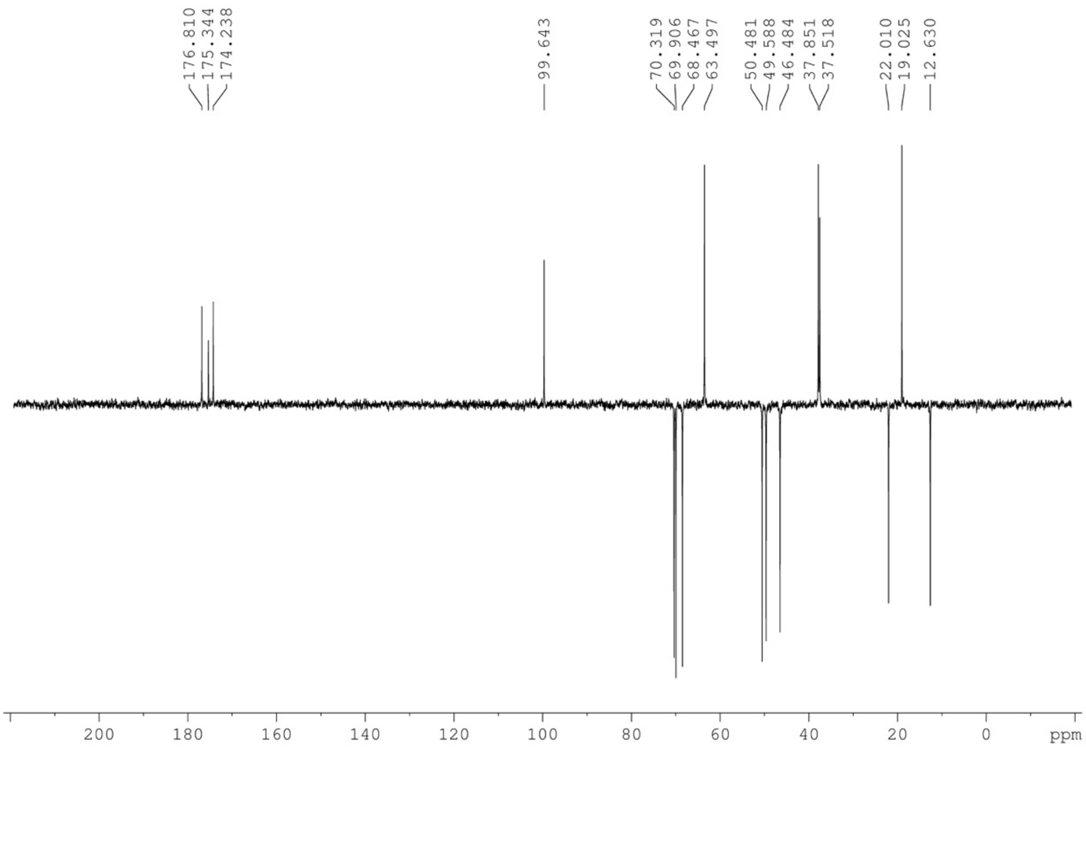

^{1}H NMR (D_2O): Methyl 5-acetamido-3,4,5-trideoxy-4-hexanamido-D-*glycero*-β-D-*galacto*-non-2-ulopyranosidonic acid, lithium salt (**27b**)

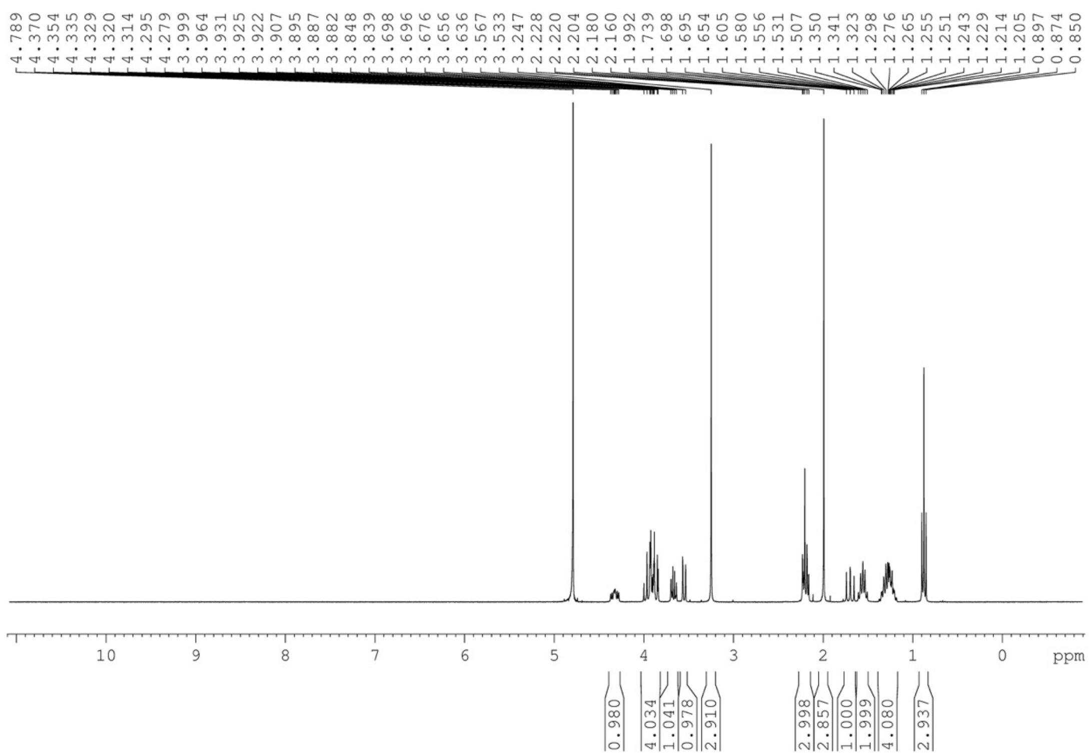

^{13}C NMR (D_2O): Methyl 5-acetamido-3,4,5-trideoxy-4-hexanamido-D-*glycero*-β-D-*galacto*-non-2-ulopyranosidonic acid, lithium salt (**27b**)

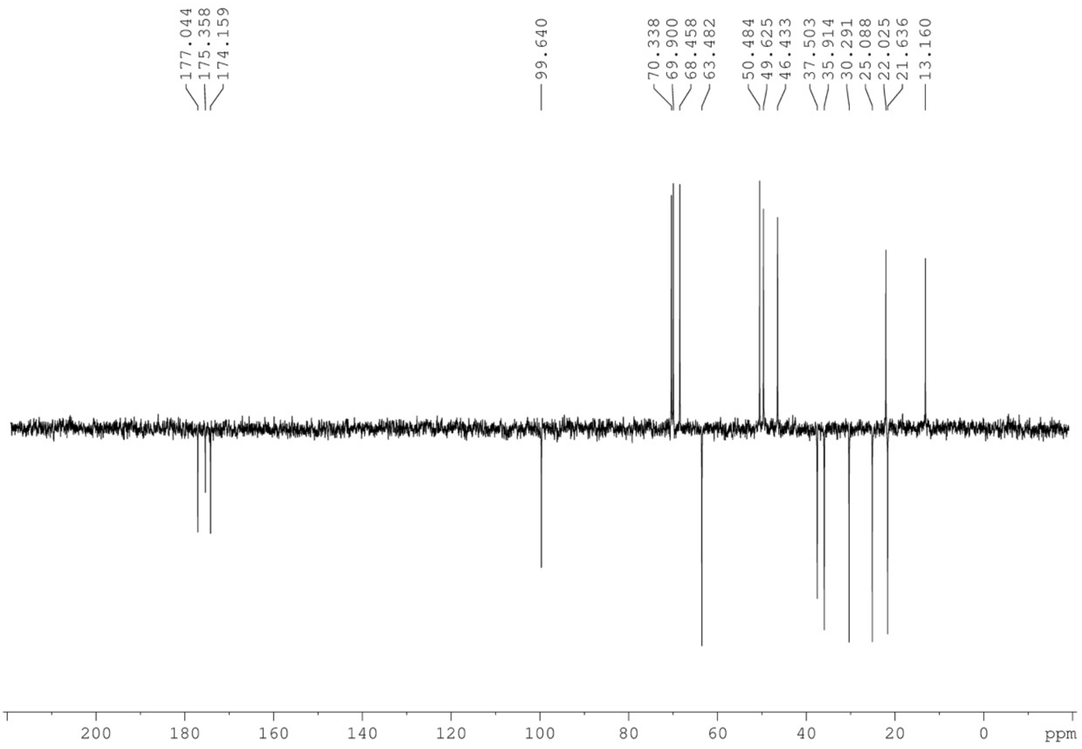

^{1}H NMR (D_2O): Methyl 5-acetamido-3,4,5-trideoxy-4-(2-ethyl-butanamido)-D-*glycero*-β-D-*galacto*-non-2-ulopyranosidonic acid, lithium salt (**27c**)

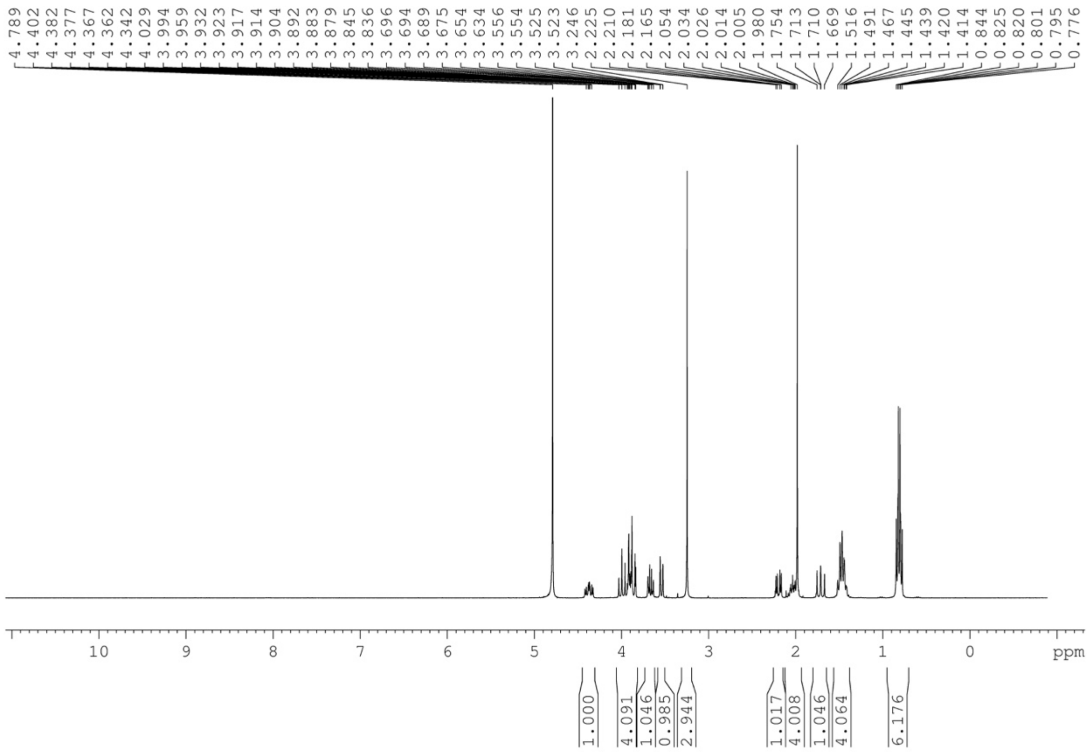

^{13}C NMR (D_2O): Methyl 5-acetamido-3,4,5-trideoxy-4-(2-ethyl-butanamido)-D-*glycero*-β-D-*galacto*-non-2-ulopyranosidonic acid, lithium salt (**27c**)

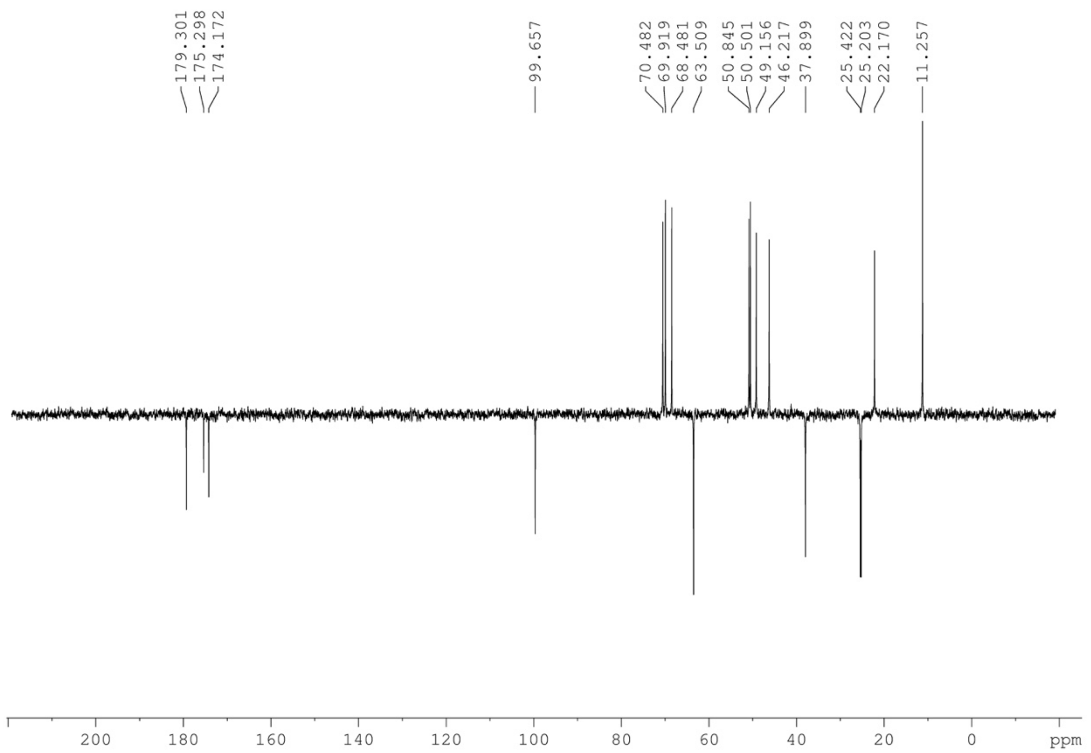

^{1}H NMR (D_2O): Methyl 5-acetamido-3,4,5-trideoxy-4-(2-ethyl-hexanamido)-D-*glycero*-β-D-*galacto*-non-2-ulopyranosidonic acid, lithium salt (**27d**)

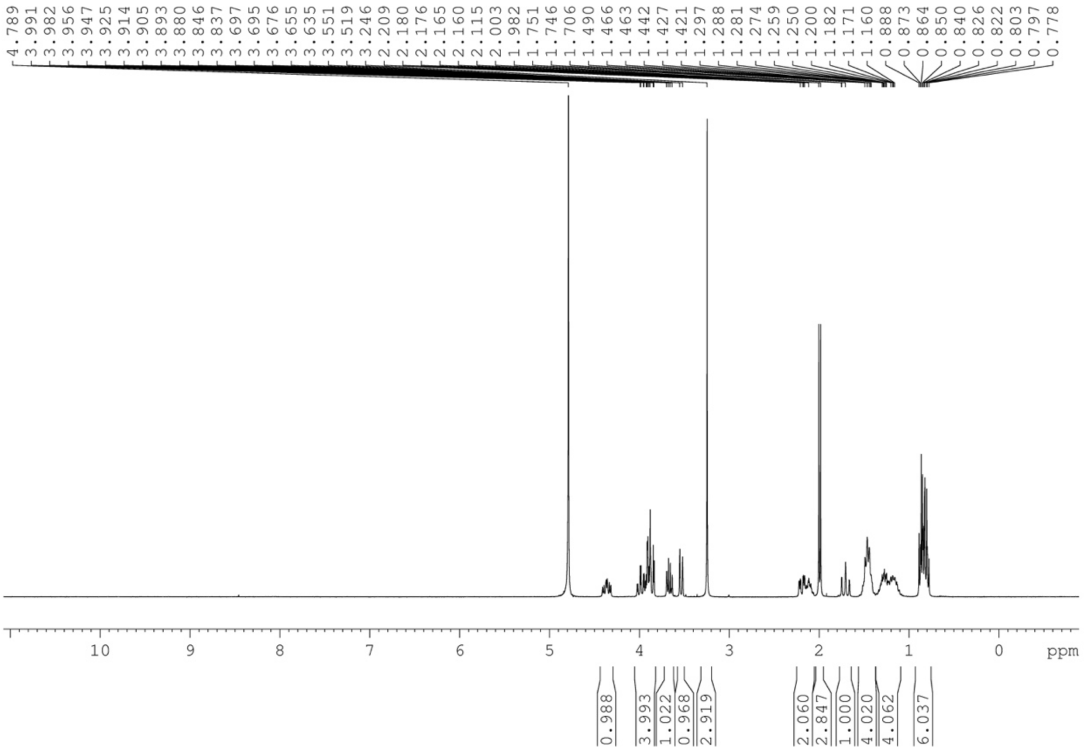

^{13}C NMR (D_2O): Methyl 5-acetamido-3,4,5-trideoxy-4-(2-ethyl-hexanamido)-D-*glycero*-β-D-*galacto*-non-2-ulopyranosidonic acid, lithium salt (**27d**)

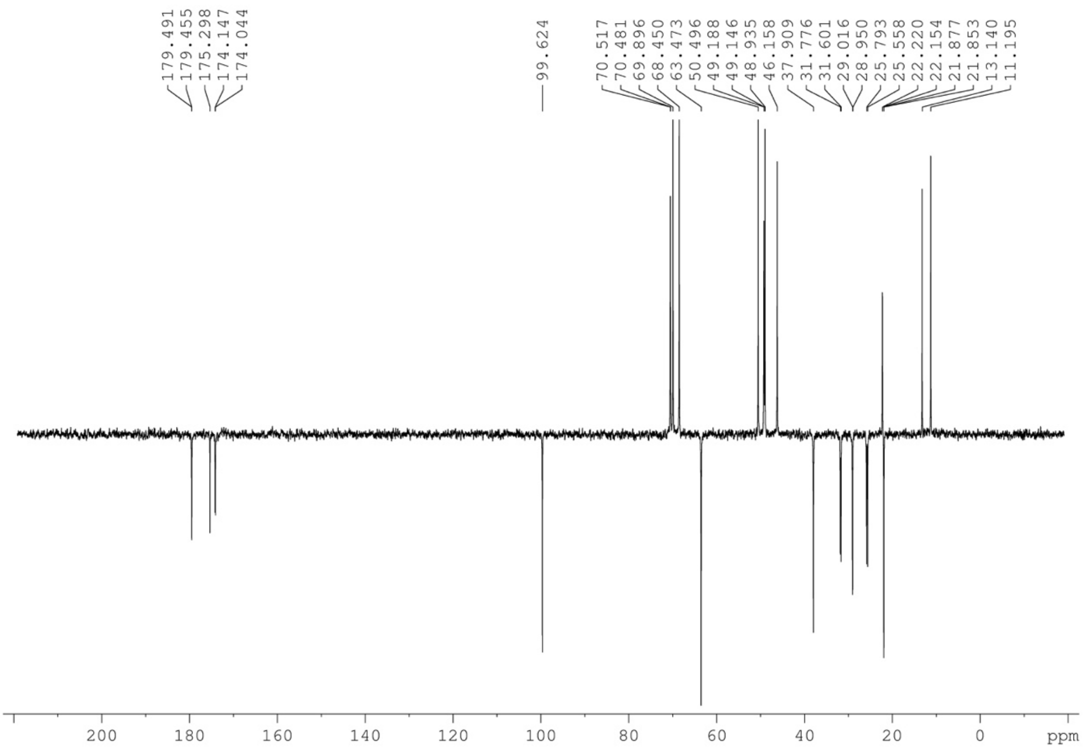

^{1}H NMR (D_2O): Methyl 5-acetamido-3,4,5-trideoxy-4-(3-nitro-benzamido)-D-*glycero*-β-D-*galacto*-non-2-ulopyranosidonic acid, lithium salt (**27e**)

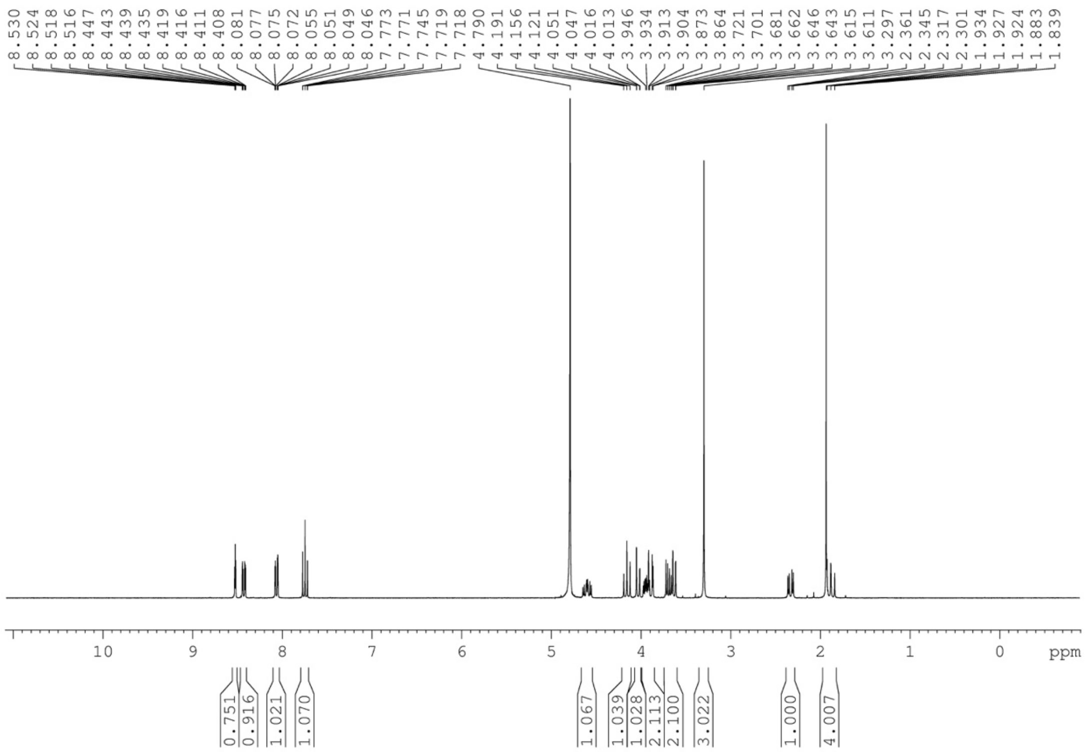

^{13}C NMR (D_2O): Methyl 5-acetamido-3,4,5-trideoxy-4-(3-nitro-benzamido)-D-*glycero*-β-D-*galacto*-non-2-ulopyranosidonic acid, lithium salt (**27e**)

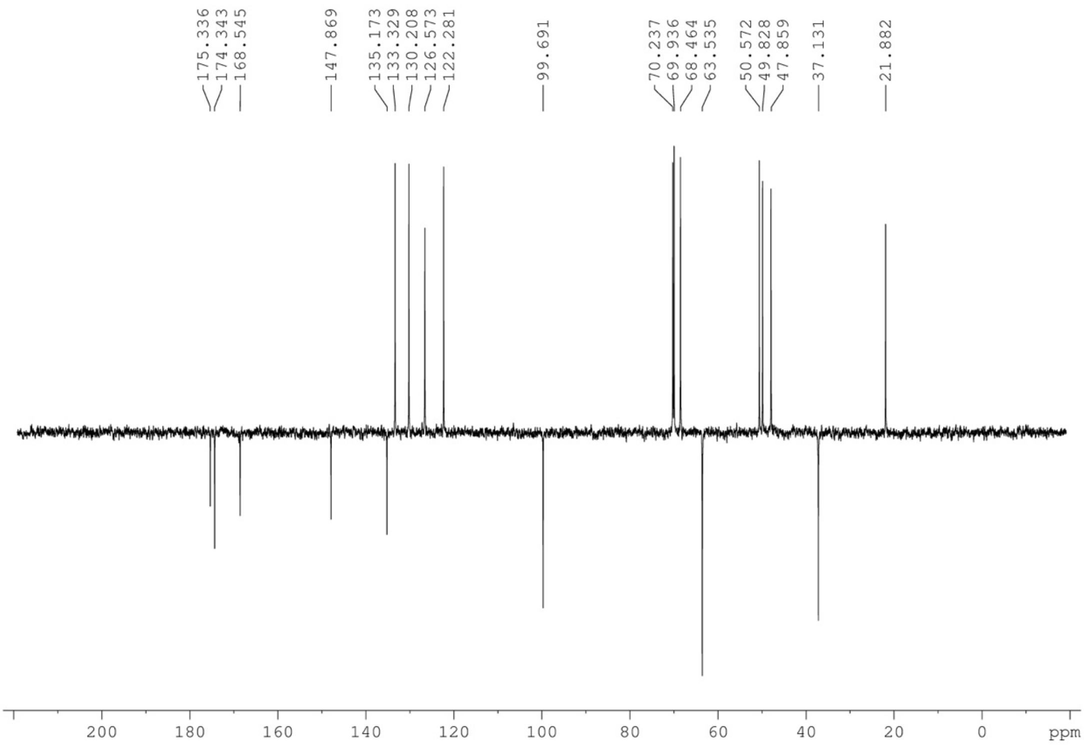

^{1}H NMR (D_2O): Methyl 5-acetamido-3,4,5-trideoxy-4-(3-phenyl-propanamido)-D-*glycero*-β-D-*galacto*-non-2-ulopyranosidonic acid, lithium salt (**27f**)

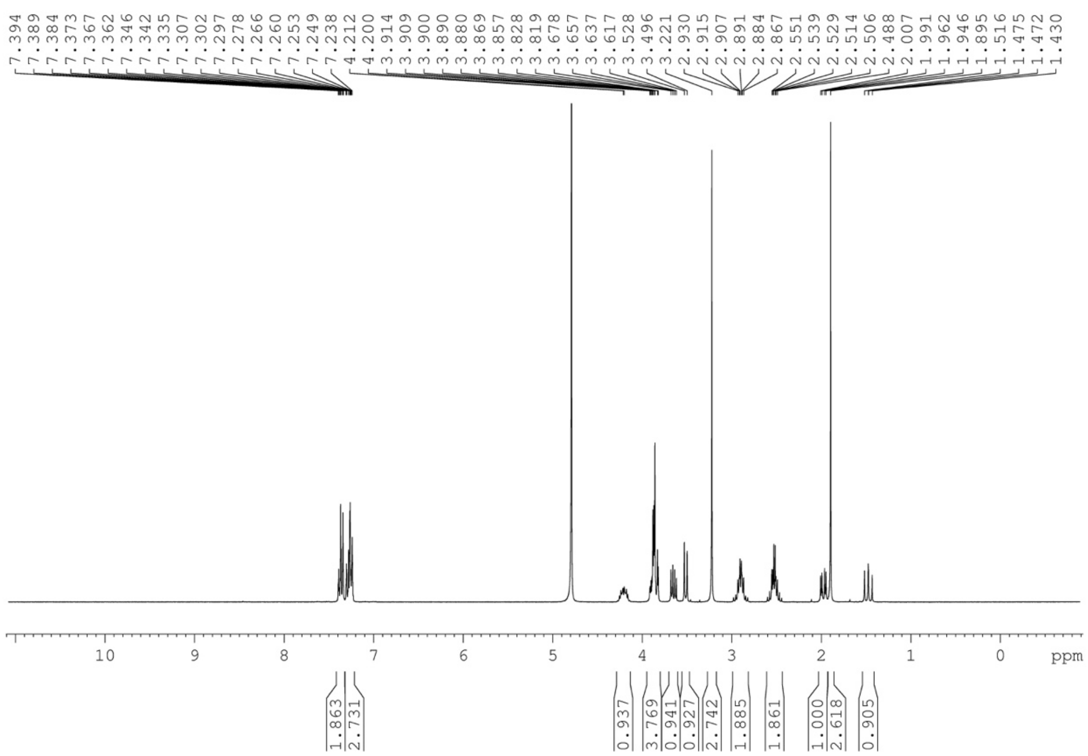

^{13}C NMR (D_2O): Methyl 5-acetamido-3,4,5-trideoxy-4-(3-phenyl-propanamido)-D-*glycero*-β-D-*galacto*-non-2-ulopyranosidonic acid, lithium salt (**27f**)

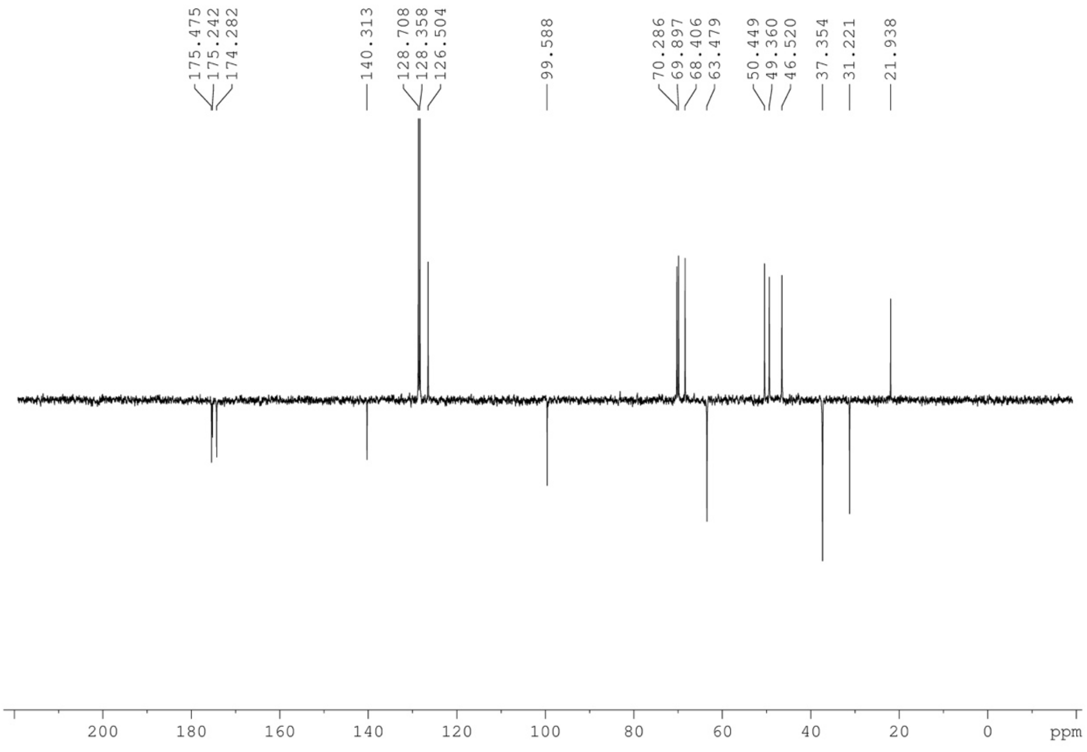

^{1}H NMR (D_2O): Methyl 5-acetamido-3,4,5-trideoxy-4-(4-phenoxy-butanamido)-D-*glycero*-β-D-*galacto*-non-2-ulopyranosidonic acid, lithium salt (**27g**)

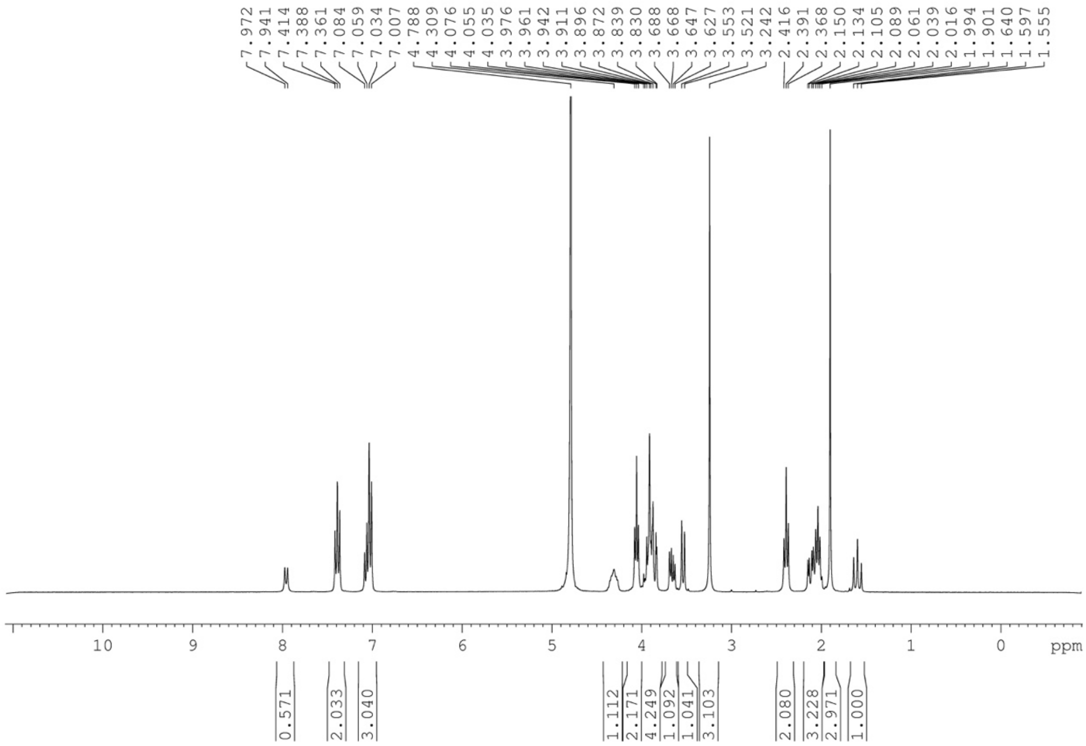

^{13}C NMR (D_2O): Methyl 5-acetamido-3,4,5-trideoxy-4-(4-phenoxy-butanamido)-D-*glycero*-β-D-*galacto*-non-2-ulopyranosidonic acid, lithium salt (**27g**)

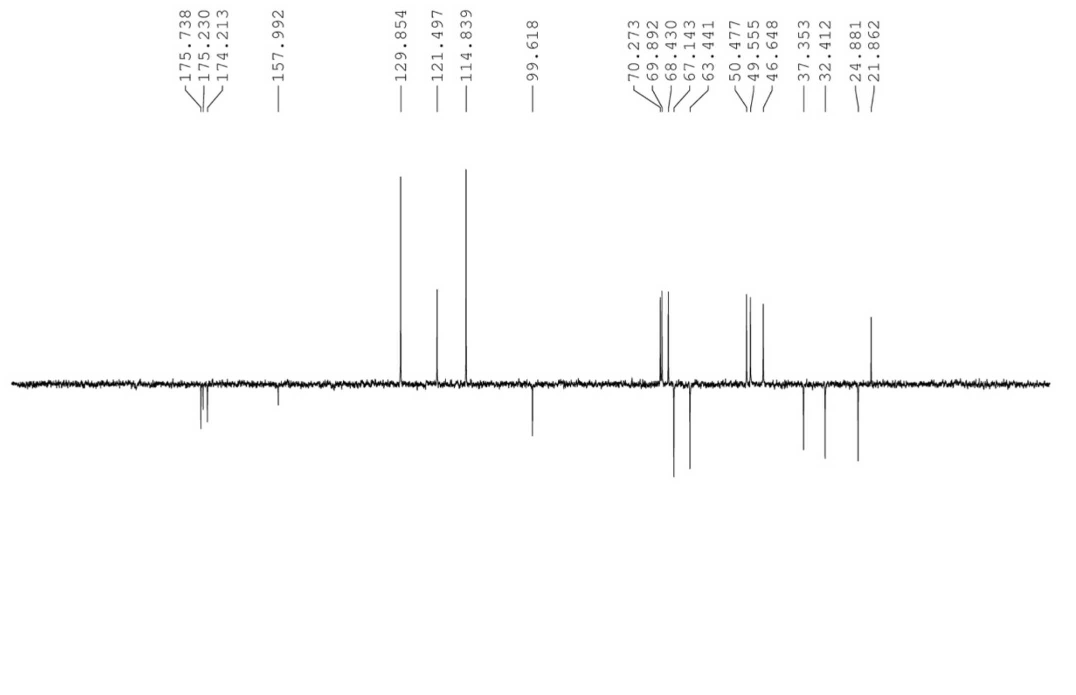

^{1}H NMR (D_2O): Methyl 5-acetamido-3,4,5-trideoxy-4-(4-phenyl-benzamido)-D-*glycero*-β-D-*galacto*-non-2-ulopyranosidonic acid, lithium salt (**27h**)

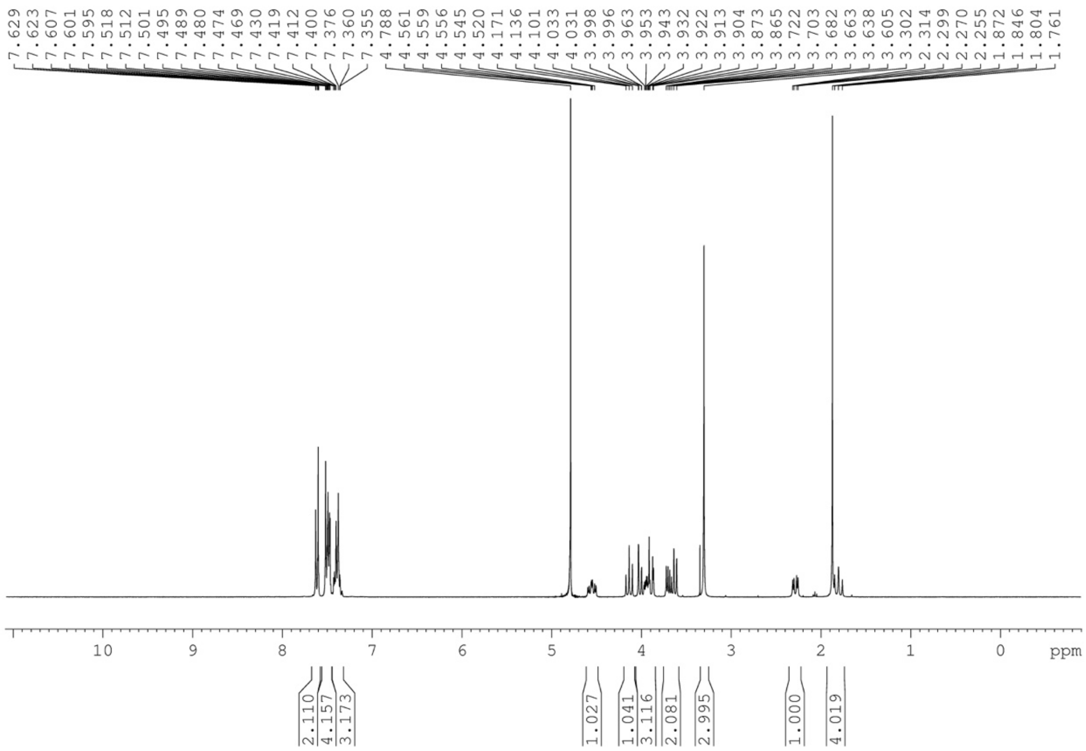

^{13}C NMR (D_2O): Methyl 5-acetamido-3,4,5-trideoxy-4-(4-phenyl-benzamido)-D-*glycero*-β-D-*galacto*-non-2-ulopyranosidonic acid, lithium salt (**27h**)

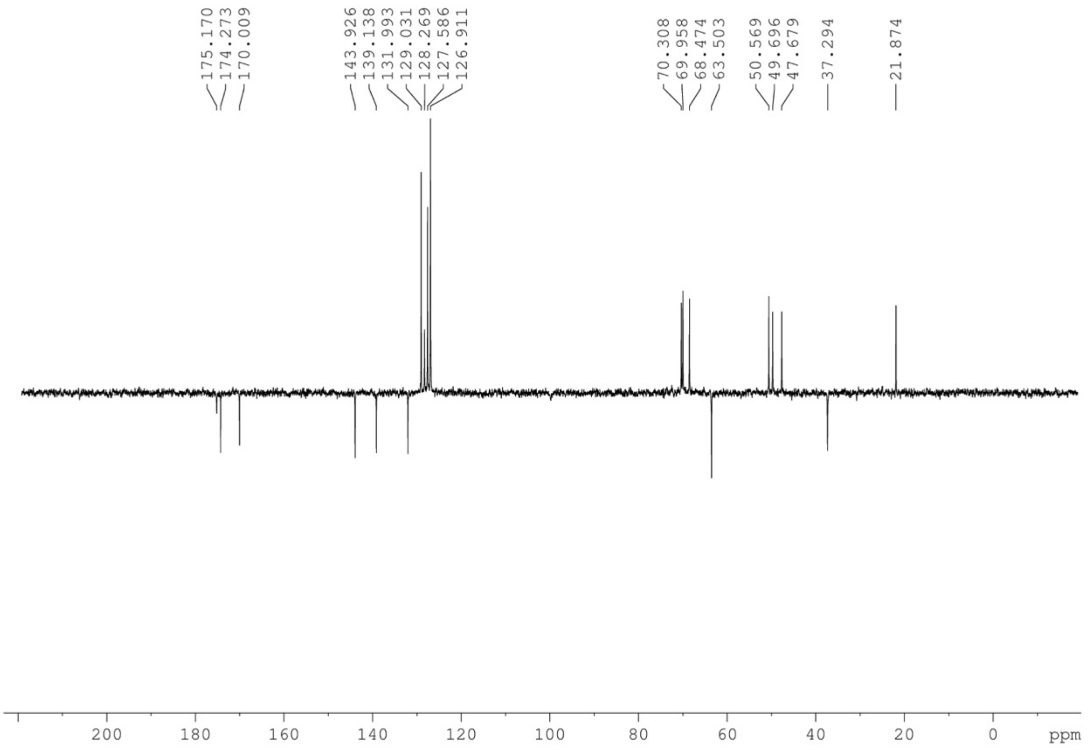

^{1}H NMR (D_2O): Methyl 5-acetamido-3,4,5-trideoxy-4-(1-naphthylcarboxamido)-D-*glycero*-β-D-*galacto*-non-2-ulopyranosidonic acid, lithium salt (**27i**)

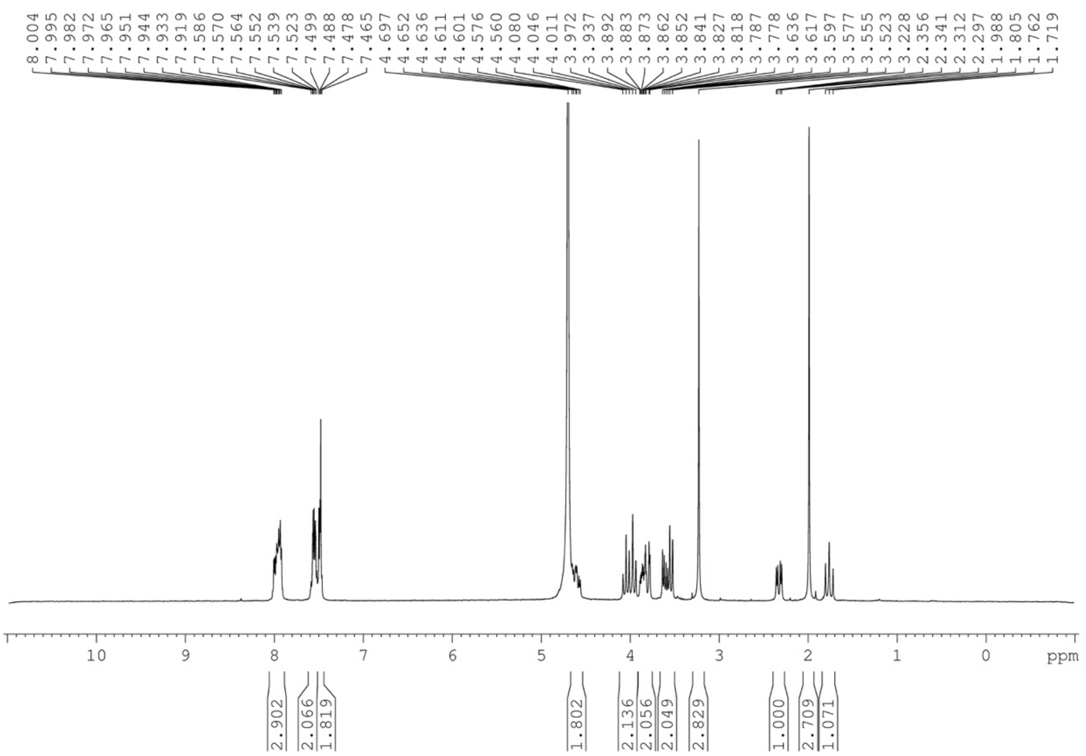

^{13}C NMR (D_2O): Methyl 5-acetamido-3,4,5-trideoxy-4-(1-naphthylcarboxamido)-D-*glycero*-β-D-*galacto*-non-2-ulopyranosidonic acid, lithium salt (**27i**)

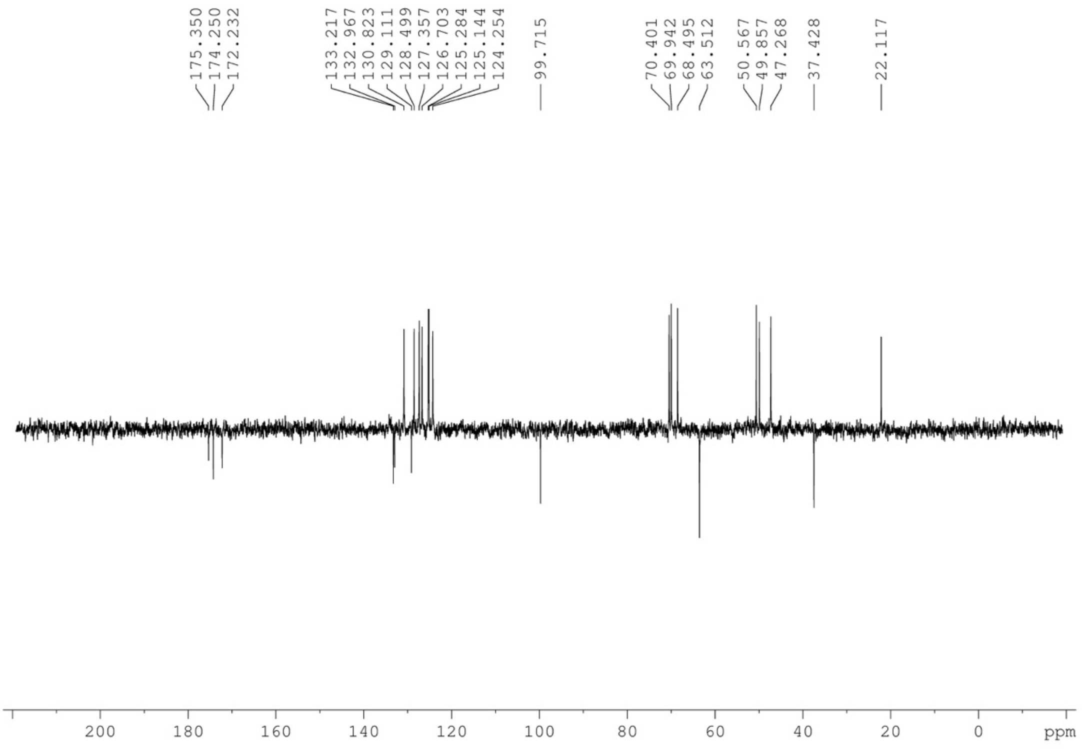

Supplement: Supplementary file 1 [file molecules-30-04329-s001.zip › Supporting information-2.pdf]
